# Supplementary material for: Chiral donor–acceptor azetines as powerful reactants for synthesis of amino acid derivatives
Source: Nat Commun. 2019 Nov 22;10:5328. doi: 10.1038/s41467-019-13326-8 (PMC6874555; doi:10.1038/s41467-019-13326-8)
Supplement: Supplementary file 2 — Supplementary Information [file 41467_2019_13326_MOESM2_ESM.pdf]

Supplementary Information

Chiral donor–acceptor azetines as powerful reactants for synthesis of amino acid derivatives

Marichev et al.

Table of Contents

Supplementary Methods 2
General information 2
General procedure for the synthesis of *N*-arylsulfilimines **2** 3
Procedure for the copper-catalyzed [3+1]-cycloaddition of enoldiazoacetate **1a** with *N*-arylsulfilimine **2a** 4
Optimized procedure for chiral copper-catalyzed [3+1]-cycloaddition of enoldiazoacetates with *N*-arylsulfilimine **2a** 4
Characterization of donor–acceptor azetines **3** 5
Procedure for the 2 mmol scale synthesis of methyl (*R*)-1-(4-chlorophenyl)-4-ethyl-3-[(triisopropylsilyl)oxy]-1,4-dihydroazete-2-carboxylate (**3c**) 10
Procedures for ring opening reactions of donor–acceptor azetines **3** 10
Characterization of ring opened products 12
Procedure for preparative deuterium incorporation 22
Procedure for proton–deuterium exchange with CD_3OD 23
Procedure for Suzuki-Miyaura cross-coupling of a ring opened product 25
Procedure for the ring opening of azetine **3c** with 9-aminoacridine 26
Fluorescence analysis for **34** 26
Procedure for esterification of cholesterol and ergocalciferol (vitamin D_2) 27
X-ray crystallographic report for **35** 29
Supplementary Figures 31
NMR spectra of new compounds 31
HPLC traces of racemic and chiral azetines **3** 99
HPLC traces of racemic and chiral ring opened products (selected examples) 114
Supplementary References 120

Supplementary Methods

General information

Unless otherwise noted, all reactions were carried out in oven-dried (120 °C) glassware with magnetic stirring under an atmosphere of dry nitrogen. Tetrahydrofuran, dichloromethane, chloroform, and toluene were purified using a *JC*-Meyer solvent purification system. All other solvents were purified and dried using standard methods. Thin layer chromatography (TLC) was carried out using Dynamic Adsorbents precoated (0.25 mm, F254) silica gel plates. Column chromatography was performed on CombiFlash® Rf200 and Rf+ purification systems using normal phase disposable columns. Melting points were measured uncorrected on an Electro Thermo Mel-Temp DLX 104 device. High-resolution mass spectra (HRMS) were performed on a Bruker MicroTOF-ESI mass spectrometer with an ESI resource using CsI or LTQ ESI positive ion calibration solution as the standard. Accurate masses were reported for the molecular ions $[M+H]^+$, $[M+Na]^+$ or $[M+K]^+$. Enantioselectivities were determined by HPLC analysis at 25°C using an Agilent 1260 Infinity HPLC System equipped with a G1311B quaternary pump, G1315D diode array detector, G1329B auto-sampler, G1316A thermostated column compartment and G1170A valve drive. For instrument control and data processing, the Agilent OpenLAB CDS ChemStation Edition for LC & LC/MS Systems (Rev. C.01.07[26]) software was used. Chiralpak AD-H (0.46 mm x 250 mm) and OD-H (0.46 mm x 250 mm) columns were obtained from Daicel Chiral Technologies. UV-Visible spectra were recorded using a Shimadzu UV-2600 spectrophotometer. Corrected steady-state fluorescence spectra were obtained with a FLS 1000 Edinburgh fluorometer. A 1 cm^2 square quartz cuvette was used for solution spectra, and emission was collected at 90° relative to the excitation beam. ^1H NMR spectra were recorded on a Bruker spectrometer (300 or 500 MHz) in $CDCl_3$ with residual $CHCl_3$ (δ 7.26 ppm) and H_2O (δ 1.56 ppm); CD_3OD [δ 3.31 ppm (CH_3OH), δ 4.87 ppm (H_2O)]; DMSO-d_6 [δ 2.50 ppm (DMSO), δ 3.33 ppm (H_2O)]. Chemical shifts (δ values) are reported in ppm downfield from the internal standard tetramethylsilane (TMS, δ 0.00 ppm). Multiplicities are reported as: s (singlet); br (broad singlet); d (doublet); t (triplet); q (quartet); dd (doublet of doublets); m (multiplet); comp (composite of magnetically non-equivalent protons). The number of protons (*n*) for a given resonance is reported as *n*H. Coupling constants (*J*) are given in Hertz (Hz). ^{13}C NMR spectra were recorded in $CDCl_3$, CD_3OD and DMSO-d_6 on a Bruker spectrometer at 75 or 126 MHz with the central resonance for $CDCl_3$ of δ 77.16, for CD_3OD of δ 49.00, and for DMSO-d_6 of δ 39.52 ppm. ^{19}F NMR spectra for fluorine containing compounds were recorded on a Bruker spectrometer at 471 MHz.

Materials. Enoldiazoacetates **1** were synthesized according to the published procedures.[1,2] $Cu(MeCN)_4PF_6$ was purchased from Sigma–Aldrich. Chiral sabox ligand **L1** was purchased from Strem and used without further purification, or synthesized using literature procedures.[3,4] (2'-Amino-1,1'-biphenyl-2-yl)methanesulfonatopalladium(II) dimer and 2-(dicyclohexylphosphino)-1,1'-biphenyl (CyJohnPhos) for Suzuki coupling were purchased from Strem. Racemic samples of azetines **3** for HPLC analysis were prepared *via* the [3+1]-cycloaddition reaction using $Cu(MeCN)_4PF_6$ and racemic sabox ligand **L2** following the general procedure. All other chemicals were obtained from commercial sources and used as received.

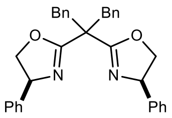

Racemic sabox ligand L2

General procedure for the synthesis of *N*-arylsulfilimines 2

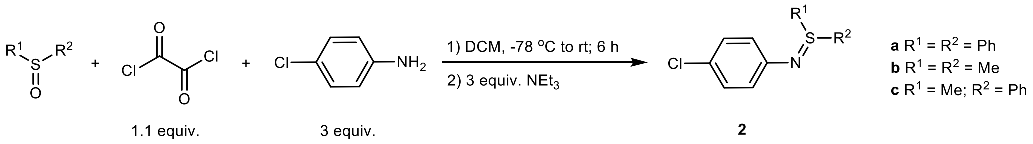

To a 250 mL oven-dried round-bottom flask equipped with a magnetic stirring bar were sequentially introduced the sulfoxide (5.00 mmol) and dry DCM (30.0 mL) under a flow of dry nitrogen. The resulting solution was cooled to −78 °C. Oxalyl chloride 470 µL (5.50 mmol) was introduced into the system, and the solution was stirred for 2 min at −78 °C. 4-Chloroaniline (1.90 g, 15.0 mmol) in dry DCM (10.0 mL) was then added dropwise over 3 min. The stirred suspension was slowly warmed to room temperature within 1 h, and stirring was continued at room temperature for 5 h. Subsequently, the reaction suspension was quenched with triethylamine (2.10 mL, 15.0 mmol); the white precipitate of triethylammonium hydrochloride was filtered, and the resulting solution was concentrated in vacuo. The residue was purified by flash chromatography on silica gel using a gradient hexane/ethyl acetate 9:1 to 4:1 (v/v) as eluent to afford *N*-arylsulfilimine **2**.

N-(4-Chlorophenyl)-1,1-diphenyl-λ_4-sulfanimine (2a)

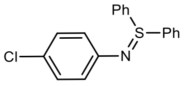

2a

1.06 g, 68% yield. Light to dark brown solid, mp 117–118 °C. ^{1}H NMR (300 MHz, $CDCl_3$) δ 7.80 – 7.70 (comp, 4H, Ph), 7.52 – 7.48 (comp, 6H, Ph), 7.10 (d, *J* = 8.8 Hz, 2H, Ar), 6.92 (d, *J* = 8.8 Hz, 2H, Ar) ppm. ^{13}C NMR (126 MHz, $CDCl_3$) δ 153.3, 139.3, 131.5, 129.7, 128.8, 127.0, 124.8, 120.2 ppm. HRMS (ESI) *m/z* calcd for $C_{18}H_{15}ClNS$ $[M+H]^+$ 312.0608; found: 312.0613.

N-(4-Chlorophenyl)-1,1-dimethyl-λ_4-sulfanimine (2b)

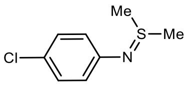

2b

673 mg, 72% yield. Pale yellow solid, mp 61–62 °C. ^{1}H NMR (300 MHz, $CDCl_3$) δ 7.09 (d, *J* = 8.8 Hz, 2H, Ar), 6.78 (d, *J* = 8.8 Hz, 2H, Ar), 2.65 (s, 6H, Me) ppm. ^{13}C NMR (75 MHz, $CDCl_3$) δ 153.5, 128.8, 121.4, 119.0, 36.3 ppm. HRMS (ESI) *m/z* calcd for $C_8H_{11}ClNS$ $[M+H]^+$ 188.0295; found: 188.0296.

N-(4-Chlorophenyl)-1-methyl-1-phenyl-λ_4-sulfanimine (2c)

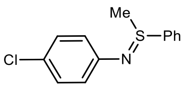

2c

710 mg, 57% yield. Light to dark brown solid, mp 73–74 °C. ^{1}H NMR (300 MHz, $CDCl_3$) δ 7.73 – 7.62 (comp, 2H, Ph), 7.58 – 7.49 (comp, 3H, Ph), 7.06 (d, *J* = 8.7 Hz, 2H, Ar), 6.74 (d, *J* = 8.7 Hz, 2H, Ar), 2.91 (s, 3H, Me) ppm. ^{13}C NMR (75 MHz, $CDCl_3$) δ 153.2, 140.1, 131.4, 129.8, 128.7, 124.9, 122.0, 119.7, 38.4 ppm. HRMS (ESI) *m/z* calcd for $C_{13}H_{13}ClNS$ $[M+H]^+$ 250.0452; found: 250.0455.

Procedure for the copper-catalyzed [3+1]-cycloaddition of enoldiazoacetate 1a with *N*-arylsulfilimine 2a

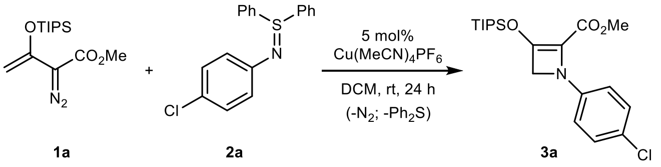

To a 8-mL oven-dried screw-capped vial equipped with a magnetic stirring bar were sequentially introduced $Cu(MeCN)_4PF_6$ (3.72 mg, 0.0100 mmol, 5 mol%), *N*-arylsulfilimine **2a** (62.2 mg, 0.200 mmol), and dry DCM (2.00 mL) under a flow of dry nitrogen. Then enoldiazoacetate **1a** (71.6 mg, 0.24 mmol) in DCM (2.00 mL) was added dropwise within 1 min, the vial was sealed, and stirring was continued at room temperature for 24 h. Subsequently, the reaction mixture was concentrated in vacuo, and the residue was purified by flash chromatography on silica gel using a gradient of hexane/ethyl acetate [49:1 to 19:1 (v/v)] as eluent to afford **methyl 1-(4-chlorophenyl)-3-[(triisopropylsilyl)oxy]-1,4-dihydroazete-2-carboxylate** (**3a**) as a pale yellow oil (63.2 mg, 80% yield). ^1H NMR (500 MHz, $CDCl_3$) δ 7.20 (d, *J* = 8.8 Hz, 2H, Ar), 6.92 (d, *J* = 8.8 Hz, 2H, Ar), 4.28 (s, 2H, CH_2), 3.82 (s, 3H, CO_2Me), 1.27 – 1.19 (m, 3H, CH in TIPS), 1.13 (d, *J* = 7.0 Hz, 18H, Me in TIPS) ppm. ^{13}C NMR (126 MHz, $CDCl_3$) δ 159.5, 152.9, 149.8, 128.8, 121.1, 119.3, 117.6, 67.1, 51.3, 17.5, 12.3 ppm. HRMS (ESI) *m/z* calcd for $C_{20}H_{31}ClNO_3Si$ $[M+H]^+$ 396.1756; found: 396.1754.

Optimized procedure for chiral copper-catalyzed [3+1]-cycloaddition of enoldiazoacetates with *N*-arylsulfilimine 2a

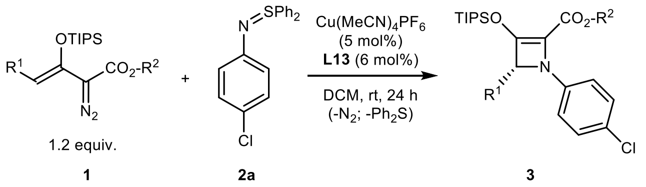

To an 8-mL oven-dried screw-capped vial equipped with a magnetic stirring bar were sequentially added $Cu(MeCN)_4PF_6$ (3.72 mg, 0.0100 mmol, 5 mol%), sabox ligand **L1** (8.81 mg, 0.012 mmol, 6 mol%), and 2.00 mL of dry DCM under a nitrogen atmosphere. The resulting solution was stirred at room temperature for 1 h. *N*-Arylsulfilimine **2a** (62.2 mg, 0.200 mmol) was then introduced to the reaction solution under a flow of nitrogen, followed by dropwise addition (over 1 min) of enoldiazoacetate **1** (0.24 mmol) in dry DCM (2.00 mL). The vial was capped, and stirring was continued at room temperature for 24–72 h. Subsequently, the reaction mixture was concentrated under reduced pressure, and the residue was purified by flash chromatography on silica gel using a gradient of hexane/ethyl acetate 49:1 to 4:1 (v/v) as eluent to afford donor-acceptor azetine **3** as a pale yellow oil.

Supplementary Table 1. *N*-Arylsulfilimine screening in copper(I)-catalyzed enantioselective [3+1]-cycloaddition with enoldiazoacetate 1b[a-c]

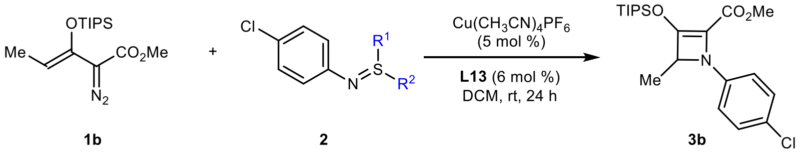

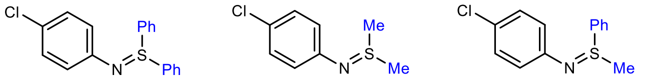

**2a**	**2b**	**2c**
75% ee, 71% yield	64% ee, 17% yield	61% ee, 66% yield

[a]Reactions were carried out on a 0.20 mmol scale in 4.0 mL DCM: **2** (0.20 mmol), **1b** (0.30 mmol). [b]Isolated yields following chromatography are reported. [c]Enantiomeric excess (ee) values were obtained by chiral HPLC analysis using a Chiralpak AD-H column.

Characterization of donor–acceptor azetines 3

Methyl (*R*)-1-(4-chlorophenyl)-4-methyl-3-[(triisopropylsilyl)oxy]-1,4-dihydroazete-2-carboxylate (3b)

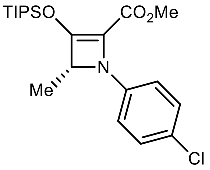

3b

67 mg, 82% yield. Pale yellow oil. 75% ee [HPLC conditions: Chiralpak AD-H column, hexanes/*i*PrOH = 95:5, flow rate = 0.7 mL/min, wavelength = 254 nm, t_R = 5.4 min for major isomer, t_R = 8.4 min for minor isomer]. ^{1}H NMR (300 MHz, $CDCl_3$) δ 7.21 (d, *J* = 8.7 Hz, 2H, Ar), 6.90 (d, *J* = 8.7 Hz, 2H, Ar), 4.02 (q, *J* = 6.8 Hz, 1H, CH), 3.79 (s, 3H, CO_2Me), 1.59 (d, *J* = 6.8 Hz, 3H, <u>Me</u>CH), 1.38 – 1.22 (m, 3H, CH in TIPS), 1.14 (d, *J* = 5.9 Hz, 18H, Me in TIPS) ppm. ^{13}C NMR (126 MHz, $CDCl_3$) δ 159.6, 153.9, 148.5, 128.8, 127.2, 120.1, 119.8, 75.0, 51.1, 17.6, 16.6, 12.7 ppm. HRMS (ESI) *m/z* calcd for $C_{21}H_{33}ClNO_3Si$ $[M+H]^+$ 410.1918; found: 410.1925.

Methyl (*R*)-1-(4-chlorophenyl)-4-ethyl-3-[(triisopropylsilyl)oxy]-1,4-dihydroazete-2-carboxylate (3c)

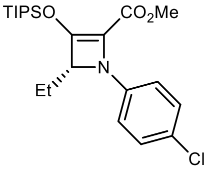

3c

78 mg, 92% yield. Pale yellow oil. 90% ee [HPLC conditions: Chiralpak AD-H column, hexanes/*i*PrOH = 98:2, flow rate = 0.7 mL/min, wavelength = 254 nm, t_R = 5.5 min for major isomer, t_R = 7.8 min for minor isomer]. ^{1}H NMR (500 MHz, $CDCl_3$) δ 7.20 (d, *J* = 8.7 Hz, 2H, Ar), 6.93 (d, *J* = 8.7 Hz, 2H, Ar), 3.88 (dd, *J* = 6.3, 5.0 Hz, 1H, CH), 3.78 (s, 3H, CO_2Me), 2.09 – 2.00 (m, 1H, CH_2 in Et), 1.97 – 1.87 (m, 1H, CH_2 in Et), 1.36 – 1.26 (m, 3H, CH in TIPS), 1.18 (t, *J* = 7.4 Hz, 3H, CH_3 in Et), 1.14 (d, *J* = 7.4 Hz, 18H, Me in TIPS) ppm. ^{13}C NMR (126 MHz, $CDCl_3$) δ 159.5, 153.1, 149.2, 128.7, 127.3, 120.4, 120.3, 80.7, 51.1, 24.2, 17.6, 12.8, 9.8 ppm. HRMS (ESI) *m/z* calcd for $C_{22}H_{35}ClNO_3Si$ $[M+H]^+$ 424.2069; found: 424.2073.

Methyl (*R*)-4-benzyl-1-(4-chlorophenyl)-3-[(triisopropylsilyl)oxy]-1,4-dihydroazete-2-carboxylate (3d)

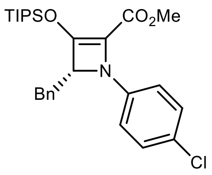

3d

71 mg, 73% yield. Pale yellow oil. 90% ee [HPLC conditions: Chiralpak AD-H column, hexanes/*i*PrOH = 98:2, flow rate = 0.7 mL/min, wavelength = 254 nm, t_R = 5.6 min for major isomer, t_R = 7.5 min for minor isomer]. ^{1}H NMR (500 MHz, $CDCl_3$) δ 7.40 – 7.29 (comp, 5H, Ph), 7.01 (d, *J* = 8.7 Hz, 2H, Ar), 6.40 (d, *J* = 8.7 Hz, 2H, Ar), 4.04 (dd, *J* = 9.5, 4.2 Hz, 1H, CH), 3.79 (s, 3H, CO_2Me), 3.24 (dd, *J* = 14.0, 4.2 Hz, 1H, CH_2), 3.13 (dd, *J* = 14.0, 9.5 Hz, 1H, CH_2), 1.35 – 1.27 (m, 3H, CH in TIPS), 1.14 (d, *J* = 7.3 Hz, 18H, Me in TIPS) ppm. ^{13}C NMR (126 MHz, $CDCl_3$) δ 159.6, 153.0, 148.7, 137.8, 129.5, 128.7, 128.5, 127.2, 126.9, 120.7, 120.4, 81.2, 51.2, 38.6, 17.7, 12.8 ppm. HRMS (ESI) *m/z* calcd for $C_{27}H_{37}ClNO_3Si$ $[M+H]^+$ 486.2226; found: 486.2224.

Methyl (*R*)-1-(4-chlorophenyl)-4-isopropyl-3-[(triisopropylsilyl)oxy]-1,4-dihydroazete-2-carboxylate (3e)

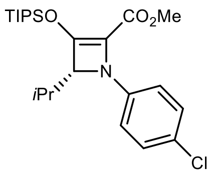

3e

61 mg, 70% yield. Pale yellow oil. 92% ee [HPLC conditions: Chiralpak AD-H column, hexanes/*i*PrOH = 98:2, flow rate = 0.7 mL/min, wavelength = 254 nm, t_R = 4.6 min for major isomer, t_R = 6.1 min for minor isomer]. ^{1}H NMR (300 MHz, $CDCl_3$) δ 7.20 (d, *J* = 8.8 Hz, 2H, Ar), 6.96 (d, *J* = 8.8 Hz, 2H, Ar), 3.77 (s, 3H, CO_2Me), 3.74 (d, *J* = 4.5 Hz, 1H, C$\underline{H}$-iPr), 2.29 – 2.18 (m, 1H, C$\underline{H}Me_2$), 1.40 – 1.28 (m, 3H, CH in TIPS), 1.21 (d, *J* = 6.8 Hz, 6H, CH$\underline{Me_2}$), 1.14 (d, *J* = 7.2 Hz, 18H, Me in TIPS) ppm. ^{13}C NMR (75 MHz, $CDCl_3$) δ 159.4, 152.8, 149.6, 128.7, 127.5, 120.8, 120.6, 84.8, 51.1, 29.7, 19.1, 18.2, 17.7, 12.9 ppm. HRMS (ESI) *m/z* calcd for $C_{23}H_{37}ClNO_3Si$ $[M+H]^+$ 438.2226; found: 438.2229.

Methyl (*R*)-1-(4-chlorophenyl)-4-octyl-3-[(triisopropylsilyl)oxy]-1,4-dihydroazete-2-carboxylate (3f)

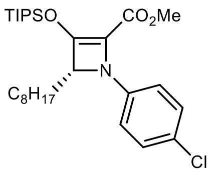

3f

64 mg, 63% yield. Pale yellow oil. 97% ee [HPLC conditions: Chiralpak AD-H column, hexanes/*i*PrOH = 99:1, flow rate = 0.5 mL/min, wavelength = 254 nm, t_R = 8.0 min for minor isomer, t_R = 8.6 min for major isomer]. ^{1}H NMR (500 MHz, $CDCl_3$) δ 7.21 (d, *J* = 8.7 Hz, 2H, Ar), 6.93 (d, *J* = 8.7 Hz, 2H, Ar), 3.90 (t, *J* = 5.9 Hz, 1H, CH), 3.78 (s, 3H, CO_2Me), 1.98 – 1.86 (comp, 2H, C$\underline{H}_2$CH), 1.39 – 1.26 (comp, 12H of CH_2 in C_8H_{17}, and 3H of CH in TIPS), 1.14 (d, *J* = 7.5 Hz, 18H, Me in TIPS), 0.90 (t, *J* = 6.8 Hz, 3H, CH_3 in C_8H_{17}) ppm. ^{13}C NMR (126 MHz, $CDCl_3$) δ 159.5, 153.4, 149.2, 128.7, 127.3, 120.4, 120.3, 79.7, 51.1, 31.8, 31.4, 29.7, 29.4, 29.2, 25.6, 22.7, 17.6, 14.1, 12.8 ppm. HRMS (ESI) *m/z* calcd for $C_{28}H_{47}ClNO_3Si$ $[M+H]^+$ 508.3008; found: 508.3015.

Isopropyl (*R*)-1-(4-chlorophenyl)-4-ethyl-3-[(triisopropylsilyl)oxy]-1,4-dihydroazete-2-carboxylate (3g)

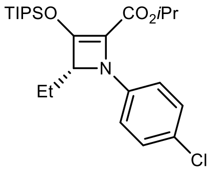

3g

74 mg, 82% yield. Pale yellow oil. 89% ee [HPLC conditions: Chiralpak AD-H column, hexanes/*i*PrOH = 98:2, flow rate = 0.7 mL/min, wavelength = 254 nm, t_R = 5.5 min for major isomer, t_R = 6.8 min for minor isomer]. ^{1}H NMR

(500 MHz, $CDCl_3$) δ 7.20 (d, *J* = 8.8 Hz, 2H, Ar), 6.93 (d, *J* = 8.8 Hz, 2H, Ar), 5.19 (sep, *J* = 6.5 Hz, 1H, CH in iPr), 3.87 (dd, *J* = 6.5, 4.8 Hz, 1H, CH), 2.09 – 2.00 (m, 1H, CH_2 in Et), 1.98 – 1.85 (m, 1H, CH_2 in Et), 1.37 – 1.29 (comp, 6H, Me in iPr, CH in TIPS), 1.27 (d, *J* = 6.5 Hz, 3H, Me in iPr), 1.18 (t, *J* = 7.4 Hz, 3H, CH_3 in Et), 1.14 (dd, *J* = 7.4, 3.3 Hz, 18H, Me in TIPS) ppm. ^{13}C NMR (126 MHz, $CDCl_3$) δ 158.9, 152.2, 149.3, 128.6, 127.1, 121.1, 120.4, 80.4, 67.8, 24.2, 22.0, 21.9, 17.7, 12.7, 9.9 ppm. HRMS (ESI) *m/z* calcd for $C_{24}H_{39}ClNO_3Si$ $[M+H]^+$ 452.2382; found: 452.2397.

Benzyl (*R*)-1-(4-chlorophenyl)-4-ethyl-3-[(triisopropylsilyl)oxy]-1,4-dihydroazete-2-carboxylate (3h)

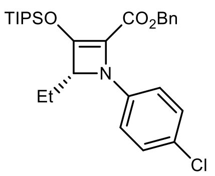

3h

87 mg, 87% yield. Pale yellow oil. 92% ee [HPLC conditions: Chiralpak AD-H column, hexanes/*i*PrOH = 98:2, flow rate = 0.7 mL/min, wavelength = 254 nm, t_R = 6.3 min for major isomer, t_R = 11.2 min for minor isomer]. ^{1}H NMR (500 MHz, $CDCl_3$) δ 7.38 – 7.29 (comp, 5H, Ar in Bn), 7.18 (d, *J* = 8.8 Hz, 2H, Ar), 6.93 (d, *J* = 8.8 Hz, 2H, Ar), 5.31 (d, *J* = 12.2 Hz, 1H, CH_2 in Bn), 5.20 (d, *J* = 12.2 Hz, 1H, CH_2 in Bn), 3.89 (dd, *J* = 6.5, 4.8 Hz, 1H, CH), 2.09 – 2.00 (m, 1H, CH_2 in Et), 1.96 – 1.88 (m, 1H, CH_2 in Et), 1.26 – 1.13 (comp, 6H, CH_3 in Et, CH in TIPS), 1.07 (dd, *J* = 7.4, 4.0 Hz, 18H, Me in TIPS) ppm. ^{13}C NMR (126 MHz, $CDCl_3$) δ 158.9, 153.1, 149.1, 135.7, 128.7, 128.6, 128.4, 128.2, 127.3, 120.5, 120.3, 80.6, 66.0, 24.2, 17.6, 12.7, 9.9 ppm. HRMS (ESI) *m/z* calcd for $C_{28}H_{39}ClNO_3Si$ $[M+H]^+$ 500.2382; found: 500.2386.

4-Bromobenzyl (*R*)-1-(4-chlorophenyl)-4-ethyl-3-[(triisopropylsilyl)oxy]-1,4-dihydroazete-2-carboxylate (3i)

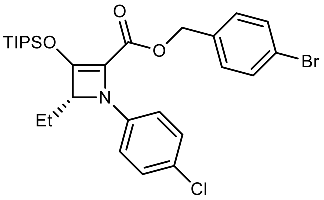

3i

87 mg, 90% yield. Pale yellow oil. 90% ee [HPLC conditions: Chiralpak AD-H column, hexanes/*i*PrOH = 98:2, flow rate = 0.7 mL/min, wavelength = 254 nm, t_R = 6.6 min for major isomer, t_R = 12.6 min for minor isomer]. ^{1}H NMR (500 MHz, $CDCl_3$) δ 7.47 (d, *J* = 8.5 Hz, 2H, Ar), 7.21 – 7.16 (comp, 4H, Ar), 6.92 (d, *J* = 8.5 Hz, 2H, Ar), 5.25 (d, *J* = 12.4 Hz, 1H, CH_2 in Bn), 5.12 (d, *J* = 12.4 Hz, 1H, CH_2 in Bn), 3.89 (t, *J* = 5.5 Hz, 1H, CH), 2.09 – 1.99 (m, 1H, CH_2 in Et), 1.96 – 1.86 (m, 1H, CH_2 in Et), 1.27 – 1.18 (m, 3H, CH in TIPS), 1.17 (t, *J* = 7.3 Hz, 3H, CH_3 in Et), 1.08 (dd, *J* = 7.3, 3.9 Hz, 18H, Me in TIPS) ppm. ^{13}C NMR (126 MHz, $CDCl_3$) δ 158.7, 153.5, 149.1, 134.8, 131.6, 130.2, 128.7, 127.4, 122.3, 120.5, 120.3, 80.7, 65.1, 24.2, 17.6, 12.7, 9.9 ppm. HRMS (ESI) *m/z* calcd for $C_{28}H_{38}BrClNO_3Si$ $[M+H]^+$ 578.1487; found: 578.1502.

4-Methoxybenzyl (*R*)-1-(4-chlorophenyl)-4-ethyl-3-[(triisopropylsilyl)oxy]-1,4-dihydroazete-2-carboxylate (3j)

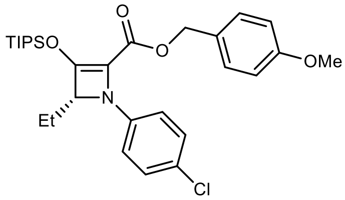

3j

100 mg, 95% yield. Pale yellow oil. >99% ee [HPLC conditions: Chiralpak OD-H column, hexanes/*i*PrOH = 98:2, flow rate = 0.7 mL/min, wavelength = 254 nm, t_R = 7.4 min for major isomer, t_R = 8.6 min for minor isomer]. ^{1}H NMR (500 MHz, $CDCl_3$) δ 7.28 (d, *J* = 8.6 Hz, 2H, Ar), 7.16 (d, *J* = 8.8 Hz, 2H, Ar), 6.91 (d, *J* = 8.8 Hz, 2H, Ar), 6.87 (d, *J* = 8.6 Hz, 2H, Ar), 5.25 (d, *J* = 11.9 Hz, 1H, CH_2 in Bn),

5.13 (d, *J* = 11.9 Hz, 1H, CH_2 in Bn), 3.87 (dd, *J* = 6.3, 5.0 Hz, 1H, CH), 3.83 (s, 3H, OMe), 2.07 – 1.99 (m, 1H, CH_2 in Et), 1.96 – 1.86 (m, 1H, CH_2 in Et), 1.27 – 1.16 (m, 3H, CH in TIPS), 1.17 (t, *J* = 7.4 Hz, 3H, CH_3 in Et), 1.07 (d, *J* = 7.4 Hz, 18H, Me in TIPS) ppm. ^{13}C NMR (126 MHz, $CDCl_3$) δ 159.6, 159.0, 152.9, 149.1, 130.5, 128.7, 127.9, 127.2, 120.6, 120.4, 113.74, 80.5, 65.8, 55.3, 24.2, 17.6, 12.7, 9.9 ppm. HRMS (ESI) *m/z* calcd for $C_{29}H_{41}ClNO_4Si$ $[M+H]^+$ 530.2488; found: 530.2485.

3,4,5-Trimethoxybenzyl (*R*)-1-(4-chlorophenyl)-4-ethyl-3-[(triisopropylsilyl)oxy]-1,4-dihydroazete-2-carboxylate (3k)

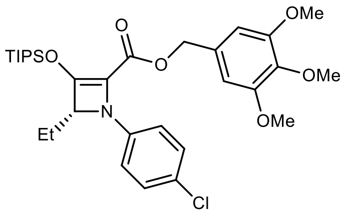

3k

82 mg, 70% yield. Pale yellow oil. 98% ee [HPLC conditions: Chiralpak AD-H column, hexanes/*i*PrOH = 95:5, flow rate = 1.0 mL/min, wavelength = 254 nm, t_R = 6.6 min for major isomer, t_R = 9.8 min for minor isomer]. ^{1}H NMR (500 MHz, $CDCl_3$) δ 7.18 (d, *J* = 8.6 Hz, 2H, Ar), 6.93 (d, *J* = 8.6 Hz, 2H, Ar), 6.54 (s, 2H, Ar), 5.25 (d, *J* = 12.1 Hz, 1H, CH_2 in Bn), 5.08 (d, *J* = 12.1 Hz, 1H, CH_2 in Bn), 3.90 – 3.87 (m, 1H, CH), 3.86 (s, 3H, *p*-OMe), 3.80 (s, 6H, *m*-OMe), 2.08 – 1.98 (m, 1H, CH_2 in Et), 1.97 – 1.87 (m, 1H, CH_2 in Et), 1.32 – 1.21 (m, 3H, CH in TIPS), 1.18 (t, *J* = 7.3 Hz, 3H, CH_3 in Et), 1.09 (dd, *J* = 7.4, 3.9 Hz, 18H, Me in TIPS) ppm. ^{13}C NMR (126 MHz, $CDCl_3$) δ 158.8, 153.4, 153.2, 150.1, 149.1, 137.9, 131.3, 128.7, 124.3, 120.5, 105.7, 80.7, 66.2, 60.8, 56.0, 31.6, 24.2, 17.6, 12.8, 9.9 ppm. HRMS (ESI) *m/z* calcd for $C_{31}H_{45}ClNO_6Si$ $[M+H]^+$ 590.2699; found: 590.2706.

4-(Trifluoromethyl)benzyl (*R*)-1-(4-chlorophenyl)-4-ethyl-3-[(triisopropylsilyl)oxy]-1,4-dihydroazete-2-carboxylate (3l)

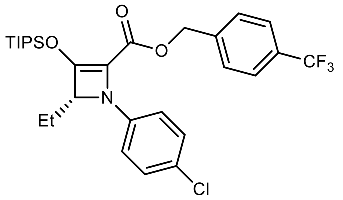

3l

83 mg, 73% yield. Pale yellow oil. 87% ee [HPLC conditions: Chiralpak AD-H column, hexanes/*i*PrOH = 98:2, flow rate = 0.7 mL/min, wavelength = 254 nm, t_R = 6.2 min for major isomer, t_R = 9.6 min for minor isomer]. ^{1}H NMR (300 MHz, $CDCl_3$) δ 7.60 (d, *J* = 8.1 Hz, 2H, Ar), 7.42 (d, *J* = 8.1 Hz, 2H, Ar), 7.19 (d, *J* = 8.7 Hz, 2H, Ar), 6.94 (d, *J* = 8.7 Hz, 2H, Ar), 5.36 (d, *J* = 12.8 Hz, 1H, CH_2 in Bn), 5.22 (d, *J* = 12.8 Hz, 1H, CH_2 in Bn), 3.90 (dd, *J* = 6.5, 4.8 Hz, 1H, CH), 2.14 – 1.81 (comp, 2H, CH_2 in Et), 1.34 – 1.22 (m, 3H, CH in TIPS), 1.18 (t, *J* = 7.5 Hz, 3H, CH_3 in Et), 1.09 (d, *J* = 7.4 Hz, 18H, Me in TIPS) ppm. ^{13}C NMR (75 MHz, $CDCl_3$) δ 158.6, 153.8, 149.0, 139.8, 129.1, 128.8, 128.5, 127.6, 125.4 (q, *J* = 3.6 Hz), 120.6, 120.1, 114.5, 80.8, 64.9, 24.2, 17.5, 12.7, 9.8 ppm. HRMS (ESI) *m/z* calcd for $C_{29}H_{38}ClF_3NO_3Si$ $[M+H]^+$ 568.2256; found: 568.2256.

4-Methoxybenzyl (*R*)-1-(4-chlorophenyl)-4-methyl-3-[(triisopropylsilyl)oxy]-1,4-dihydroazete-2-carboxylate (3m)

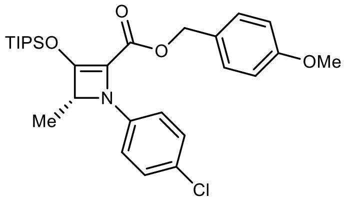

3m

79 mg, 77% yield. Pale yellow oil. 88% ee [HPLC conditions: Chiralpak AD-H column, hexanes/*i*PrOH = 90:10, flow rate = 0.7 mL/min, wavelength = 250 nm, t_R = 5.9 min for major isomer, t_R = 12.4 min for minor isomer]. ^{1}H NMR (500 MHz, $CDCl_3$) δ 7.29 (d, *J* = 8.4 Hz, 2H, Ar), 7.17 (d, *J* = 8.4 Hz, 2H, Ar), 6.90 – 6.86 (comp, 4H,

Ar), 5.24 (d, J = 11.9 Hz, 1H, CH_2 in Bn), 5.15 (d, J = 11.9 Hz, 1H, CH_2 in Bn), 4.01 (q, J = 6.7 Hz, 3H, CH), 3.83 (s, 3H, OMe), 1.57 (d, J = 6.8 Hz, 3H, MeCH), 1.25 – 1.16 (m, 3H, CH in TIPS), 1.07 (d, J = 7.0 Hz, 18H, Me in TIPS) ppm. ^{13}C NMR (126 MHz, $CDCl_3$) δ 159.7, 159.1, 153.8, 148.4, 130.6, 128.7, 127.9, 127.1, 120.2, 119.9, 113.8, 74.9, 65.8, 55.3, 17.6, 16.6, 12.7 ppm. HRMS (ESI) *m/z* calcd for $C_{28}H_{39}ClNO_4Si$ $[M+H]^+$ 516.2331; found: 516.2349.

Methyl (*R*)-4-ethyl-1-[4-(trifluoromethyl)phenyl]-3-[(triisopropylsilyl)oxy]-1,4-dihydroazete-2-carboxylate (3n)

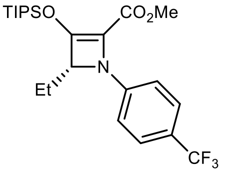

3n

80 mg, 87% yield. Pale yellow oil. 91% ee [HPLC conditions: Chiralcel OD-H column, hexanes/*i*PrOH = 99:1, flow rate = 0.7 mL/min, wavelength = 230 nm, t_R = 7.7 min for major isomer, t_R = 8.3 min for minor isomer]. ^1H NMR (500 MHz, $CDCl_3$) δ 7.46 (d, J = 8.5 Hz, 2H, Ar), 7.02 (d, J = 8.5 Hz, 2H, Ar), 4.04 (dd, J = 5.9, 5.1 Hz, 1H, CHEt), 3.80 (s, 3H, CO_2Me), 2.13 – 2.03 (m, 1H, CH_2 in Et), 1.98 – 1.90 (m, 1H, CH_2 in Et), 1.29 – 1.26 (m, 3H, CH in TIPS), 1.17 (t, J = 7.4 Hz, 3H, CH_3 in Et), 1.11 (d, J = 7.4 Hz, 18H, Me in TIPS) ppm. ^{13}C NMR (126 MHz, $CDCl_3$) δ 159.5, 153.6, 153.5, 126.0 (d, J = 3.6 Hz), 123.3 (q, J = 32.5 Hz), 120.5, 117.9, 113.0, 79.9, 51.2, 24.1, 17.6, 12.8, 9.6 ppm. ^{19}F NMR (471 MHz, $CDCl_3$) δ -61.7 ppm. HRMS (ESI) *m/z* calcd for $C_{23}H_{35}F_3NO_3Si$ $[M+H]^+$ 458.2338; found: 458.2344.

Methyl (*R*)-1-(3-fluorophenyl)-4-ethyl-3-[(triisopropylsilyl)oxy]-1,4-dihydroazete-2-carboxylate (3o)

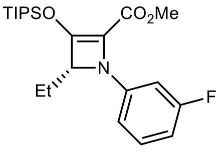

3o

71 mg, 87% yield. Pale yellow oil. 92% ee [HPLC conditions: Chiralcel OD-H column, hexanes/*i*PrOH = 99:1, flow rate = 0.7 mL/min, wavelength = 250 nm, t_R = 5.2 min for major isomer, t_R = 5.9 min for minor isomer]. ^1H NMR (500 MHz, $CDCl_3$) δ 7.19 (dd, J = 14.8, 8.0 Hz, 1H, Ar), 6.77 (dd, J = 8.0, 1.2 Hz, 1H, Ar), 6.74 – 6.64 (comp, 2H, Ar), 4.00 – 3.95 (m, 1H, CHEt), 3.80 (s, 3H, CO_2Me), 2.11 – 2.03 (m, 1H, CH_2 in Et), 1.98 – 1.89 (m, 1H, CH_2 in Et), 1.34 – 1.29 (m, 3H, CH in TIPS), 1.19 (t, J = 7.4 Hz, 3H, CH_3 in Et), 1.14 (d, J = 7.4 Hz, 18H, Me in TIPS) ppm. ^{13}C NMR (126 MHz, $CDCl_3$) δ 163.2 (d, J = 244.4 Hz), 159.5, 153.3, 152.5 (d, J = 9.1 Hz), 129.8 (d, J = 9.5 Hz), 120.5, 114.3, 108.6 (d, J = 21.5 Hz), 105.9 (d, J = 23.9 Hz), 80.3, 51.1, 24.2, 17.6, 12.8, 9.7 ppm. ^{19}F NMR (471 MHz, $CDCl_3$) δ -112.7 – -112.8 (m) ppm. HRMS (ESI) *m/z* calcd for $C_{22}H_{35}FNO_3Si$ $[M+H]^+$ 408.2370; found: 408.2370.

Methyl (*R*)-4-ethyl-1-(3-methylphenyl)-3-[(triisopropylsilyl)oxy]-1,4-dihydroazete-2-carboxylate (3p)

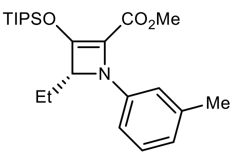

3p

69 mg, 85% yield. Pale yellow oil. 74% ee [HPLC conditions: Chiralcel OD-H column, hexanes/*i*PrOH = 99:1, flow rate = 0.7 mL/min, wavelength = 250 nm, t_R = 5.7 min for major isomer, t_R = 6.8 min for minor isomer]. ^1H NMR (500 MHz, $CDCl_3$) δ 7.14 (t, J = 7.9 Hz, 1H, Ar), 6.84 – 6.79 (comp, 3H, Ar), 3.90 (t, J = 5.6 Hz, 1H, CHEt), 3.78 (s, 3H, CO_2Me), 2.32 (s, 3H, Me–Ar), 2.11 – 2.02 (m, 1H, CH_2 in Et), 1.98 – 1.89 (m, 1H, CH_2 in Et), 1.36 – 1.28 (m, 3H, CH in TIPS), 1.19 (t, J = 7.4 Hz, 3H, CH_3 in Et), 1.14 (d, J = 7.3 Hz, 18H, Me in TIPS) ppm. ^{13}C NMR (126 MHz, $CDCl_3$) δ

159.7, 152.7, 150.6, 138.5, 128.6, 123.2, 120.6, 119.9, 116.0, 80.6, 51.0, 24.3, 21.6, 17.6, 12.8, 9.8 ppm. HRMS (ESI) *m/z* calcd for $C_{23}H_{38}NO_3Si$ $[M+H]^+$ 404.2615; found: 404.2616.

Procedure for the 2 mmol scale synthesis of methyl (*R*)-1-(4-chlorophenyl)-4-ethyl-3-[(triisopropylsilyl)oxy]-1,4-dihydroazete-2-carboxylate (3c)

To an 100-mL oven-dried round-bottom flask equipped with a magnetic stirring bar $Cu(MeCN)_4PF_6$ (37.2 mg, 0.100 mmol, 5 mol%), sabox ligand **L1** (88.1 mg, 0.120 mmol, 6 mol%), and 10.0 mL of dry DCM were sequentially added under a nitrogen atmosphere. The flask was sealed, and the resulting solution was stirred at room temperature for 1 h. *N*-arylsulfilimine **2a** (622 mg, 2.00 mmol) in dry DCM (10.0 mL) was then introduced to the reaction solution at room temperature, followed by a dropwise addition (over 5 min) of enoldiazoacetate **1c** (783 mg, 2.40 mmol) in dry DCM (20.0 mL). Stirring was continued at room temperature for 24 h. Subsequently, the reaction mixture was concentrated under reduced pressure, and the residue was purified by flash chromatography on silica gel (ca. 60 g) using a gradient of hexane/ethyl acetate [49:1 to 19:1 (v/v)] as eluent to afford **3c** as a pale yellow oil (710 mg, 84% yield, 90% ee).

Procedures for ring opening reactions of donor–acceptor azetines 3

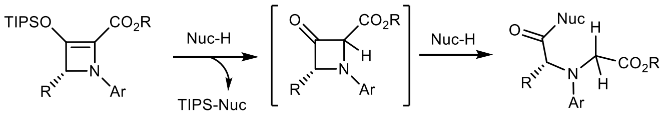

Procedure A for the synthesis of chiral carboxylato-amides 4, 5, 7−12, 14−16

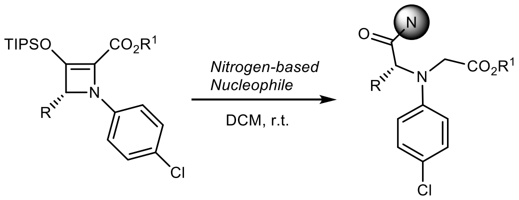

To a 8-mL screw-capped vial equipped with a magnetic stirring bar an azetine **3** (0.20 mmol), an amine (0.50 mmol), and DCM (4.0 mL) were sequentially introduced. The vial was capped, and stirring was continued at room temperature for 48 h [for **4**, **5**, **7**, **8**, **9**, **12**, **15**, **16**] or 96 h [for **10**, **11**, **14**]. After completion of the reaction (monitored by TLC), DCM was evaporated, and the residue was purified by flash chromatography on silica gel using a gradient hexane/ethyl acetate 2:1 (v/v) to pure ethyl acetate [for **4**, **5**, **8**−**12**, **14**] or a mixture DCM/methanol 9:1 (v/v) [for **7**, **15**, **16**] as eluents to afford a ring opening product.

Procedure B for the synthesis of chiral diamide 6, amido-carboxylic acids 17, 18, 19, and zwitterion 31

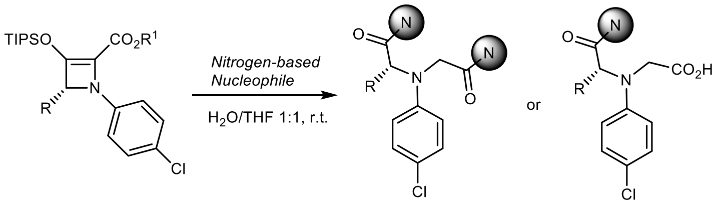

To a 8-mL screw-capped vial equipped with a magnetic stirring bar an azetine **3** (0.20 mmol), an amine (0.80 mmol), THF (2.0 mL), and water (2.0 mL) were sequentially introduced. The vial was capped, and stirring was continued at room temperature for 12 h. Solvents were then evaporated, and the residue was purified by flash chromatography on silica gel using a gradient of DCM/methanol [9:1 to 4:1 (v/v)] as eluent to afford a ring opening product.

Procedure C for the synthesis of chiral carboxylato-amide 13a and -hydrazide 27a

To a 8-mL screw-capped vial equipped with a magnetic stirring bar an azetine **3** (0.20 mmol), aniline or phenylhydrazine (0.60 mmol), and 1,2-dichloroethane or nitromethane (4.0 mL) were sequentially introduced. The vial was capped, and stirring was continued at 50 °C for 12 h [for **27a**] or at 65 °C for 24 h [for **13a**]. DCE was then evaporated, and the residue was purified by flash chromatography on silica gel using a gradient of hexane/ethyl acetate [2:1 (v/v) to pure ethyl acetate] to afford ring opening product **13a** or **27a**. Reactions in nitromethane were carried out at room temperature for 12 h (**13b**) or 24 h (**27b**).

Procedure D for the synthesis of chiral dihydroxamic acid 28, dihydrazide 29, and diamide 30

To a 8-mL screw-capped vial equipped with a magnetic stirring bar an azetine **3** (0.20 mmol), a nucleophile (1.0 mmol), and THF (4.0 mL) were sequentially introduced. The vial was capped, and stirring was continued at room temperature for 12 h. THF was then evaporated, and the residue was purified by flash chromatography on silica gel using a gradient of DCM/methanol [9:1 to 4:1 (v/v)] as eluent to afford a ring opening product.

General procedure for the synthesis of chiral diesters 20−25

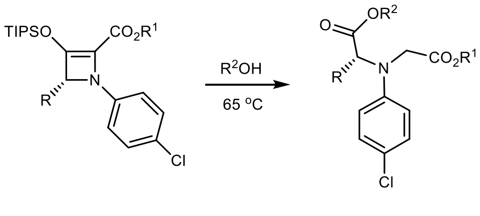

To a 8-mL screw-capped vial equipped with a magnetic stirring bar an azetine **3** (0.200 mmol) and an alcohol [5.00 mL of methanol (for **20**−**22**); 5.00 mL of ethanol (for **23**); 123 mg, 0.800 mmol of geraniol in 3.00 mL of THF (for **24**); 50.0 mg, 0.800 mmol of ethylene glycol in 3.00 mL of 1,4-dioxane/water 2:1 (for **25**)] were sequentially introduced. The vial was capped, and stirring was continued at 65 °C for 2 h (for **20**−**22**) or for 24 h (for **23**−**25**). Solvents were then

evaporated, and the residue was purified by flash chromatography on silica gel using a gradient of hexane/ethyl acetate [2:1 (v/v) to pure ethyl acetate] to afford a chiral diester.

Procedure for the silyl deprotection – ring opening of azetines 3 using TBAF

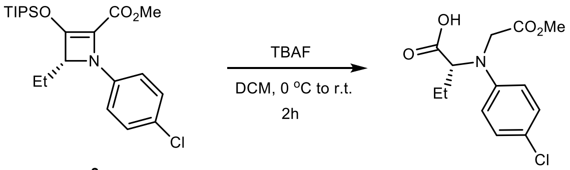

3c **26**

To an 8-mL oven-dried screw-capped vial equipped with a magnetic stirring bar azetine **3c** (84.6 mg, 0.200 mmol), and 4.00 mL of dry DCM were sequentially added under a nitrogen atmosphere. The reaction solution was cooled to 0 °C, then a 1*M* solution of tetrabutylammonium fluoride, TBAF, in THF (0.300 mL, 0.300 mmol) was added dropwise over 1 min under the flow of nitrogen. The vial was sealed, and stirring was continued at room temperature for 2 h. Subsequently, the reaction mixture was concentrated under reduced pressure, and the residue was purified by flash chromatography on silica gel using a gradient of DCM/methanol [9:1 to 4:1 (v/v)] as eluent to afford carboxylic acid monomethyl ester **26** as a colorless oil.

Characterization of ring opened products

Methyl (*R*)-*N*-[1-(benzylamino)-1-oxobutan-2-yl]-*N*-(4-chlorophenyl)glycinate (4a)

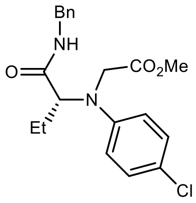

4a

71 mg, 95% yield. Colorless oil. 90% ee [HPLC conditions: Chiralpak AD-H column, hexanes/*i*PrOH = 90:10, flow rate = 1.5 mL/min, wavelength = 250 nm, t_R = 7.2 min for major isomer, t_R = 8.6 min for minor isomer]. ^{1}H NMR (300 MHz, $CDCl_3$) δ 8.51 (br, 1H, NH), 7.29 – 7.09 (comp, 7H, Ar), 6.53 (d, *J* = 9.1 Hz, 2H, Ar), 4.50 – 4.33 (comp, 2H, C<u>H</u>$_2$Ph), 4.22 (d, *J* = 18.5 Hz, 1H, C<u>H</u>$_2$CO$_2$Me), 4.07 (d, *J* = 18.5 Hz, 1H, C<u>H</u>$_2$CO$_2$Me), 4.00 (dd, *J* = 10.0, 4.9 Hz, 1H, CH), 3.70 (s, 3H, CO_2Me), 2.40 – 2.23 (m, 1H, CH_2 in Et), 1.99 – 1.82 (m, 1H, CH_2 in Et), 0.95 (t, *J* = 7.4 Hz, 3H, CH_3 in Et) ppm. ^{13}C NMR (75 MHz, $CDCl_3$) δ 172.9, 172.0, 145.2, 138.5, 129.4, 128.6, 127.8, 127.2, 123.9, 114.4, 67.9, 52.9, 51.1, 43.5, 22.7, 11.8 ppm. HRMS (ESI) *m/z* calcd for $C_{20}H_{24}ClN_2O_3$ [M+H]$^+$ 375.1470; found: 375.1472.

Methyl (*R*)-*N*-[1-(benzylamino)-1-oxobutan-2-yl]-N-[4-(trifluoromethyl)phenyl]glycinate (4b)

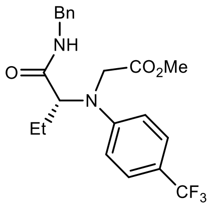

4b

72 mg, 93% yield. Colorless oil. 91% ee [based on the ee of **3n**]. ^{1}H NMR (500 MHz, $CDCl_3$) δ 8.52 (br, 1H, NH), 7.44 (d, *J* = 8.7 Hz, 2H, Ar), 7.24 – 7.20 (comp, 3H, Ar), 7.12 – 7.08 (comp, 2H), 6.62 (d, *J* = 8.7 Hz, 2H, Ar), 4.46 (dd, *J* = 14.8, 5.9 Hz, 1H, C<u>H</u>$_2$Ph), 4.46 (dd, *J* = 14.8, 5.9 Hz, 1H, C<u>H</u>$_2$Ph), 4.31 (d, *J* = 18.4 Hz, 1H, C<u>H</u>$_2$CO$_2$Me), 4.13 (d, *J* = 18.4 Hz, 1H, C<u>H</u>$_2$CO$_2$Me), 4.06 (dd, *J* = 10.0, 4.9 Hz, 1H, CH), 3.74 (s, 3H, CO_2Me), 2.40 – 2.30 (m, 1H, CH_2 in Et), 2.02 – 1.90 (m, 1H, CH_2 in Et), 0.97 (t, *J* = 7.4 Hz, 3H, CH_3 in Et) ppm. ^{13}C NMR (126 MHz, $CDCl_3$) δ 172.6, 171.3, 148.8, 138.3, 128.4, 127.6, 127.12, 126.6 (q, *J* = 3.6 Hz), 125.7, 123.6, 120.4 (q, *J* =

32.9 Hz), 112.4, 67.7, 52.9, 51.2, 43.4, 22.5, 11.7 ppm. ^{19}F NMR (471 MHz, $CDCl_3$) δ -61.38 (s) ppm. HRMS (ESI) *m/z* calcd for $C_{21}H_{23}F_3N_2O_3$ $[M+Na]^+$ 431.1553; found: 431.1544.

Methyl (*R*)-*N*-[1-(benzylamino)-1-oxobutan-2-yl]-*N*-(3-fluorophenyl)glycinate (4c)

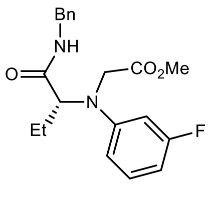

4c

65 mg, 90% yield. Colorless oil. 92% ee [based on the ee of **3o**]. ^{1}H NMR (500 MHz, $CDCl_3$) δ 8.48 (br, 1H, NH), 7.25 – 7.10 (comp, 6H, Ar), 6.50 (td, *J* = 8.3, 1.9 Hz, 1H, Ar), 6.36 (dd, *J* = 8.3, 2.3 Hz, 1H, Ar), 6.29 (dt, *J* = 12.3, 2.3 Hz, 1H, Ar), 4.41 (qd, *J* = 14.8, 5.9 Hz, 2H, C$\underline{H}_2$Ph), 4.18 (d, *J* = 18.4 Hz, 1H, C$\underline{H}_2CO_2$Me), 4.05 (d, *J* = 18.4 Hz, 1H, C$\underline{H}_2CO_2$Me), 4.04 – 3.99 (m, 1H, CH), 3.69 (s, 3H, CO_2Me), 2.40 – 2.24 (m, 1H, CH_2 in Et), 1.96 – 1.83 (m, 1H, CH_2 in Et), 0.94 (t, *J* = 7.4 Hz, 3H, CH_3 in Et) ppm. ^{13}C NMR (126 MHz, $CDCl_3$) δ 172.7, 171.7, 163.9 (d, *J* = 243.6 Hz), 148.35 (d, *J* = 10.3 Hz), 138.40, 130.5 (d, *J* = 10.1 Hz), 128.4, 127.6, 127.1, 108.7, 105.3 (d, *J* = 21.4 Hz), 100.4 (d, *J* = 26.5 Hz), 67.6, 52.8, 50.7, 43.4, 22.6, 11.7 ppm. ^{19}F NMR (471 MHz, $CDCl_3$) δ -111.40 (ddd, *J* = 12.3, 8.0, 7.2 Hz) ppm. HRMS (ESI) *m/z* calcd for $C_{20}H_{23}FN_2O_3$ $[M+K]^+$ 397.1324; found: 397.1323.

Methyl (*R*)-*N*-[1-(benzylamino)-1-oxobutan-2-yl]-*N*-(*m*-tolyl)glycinate (4d)

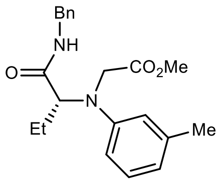

4d

65 mg, 92% yield. Colorless oil. 74% ee [based on the ee of **3o**]. ^{1}H NMR (500 MHz, $CDCl_3$) δ 8.57 (br, 1H, NH), 7.27 – 7.19 (comp, 3H, Ar), 7.18 – 7.15 (comp, 2H, Ar), 7.14 – 7.09 (m, 1H, Ar), 6.66 (d, *J* = 7.4 Hz, 1H, Ar), 6.46 – 6.42 (comp, 2H, Ar), 4.49 – 4.40 (comp, 2H, C$\underline{H}_2$Ph), 4.19 (d, *J* = 18.1 Hz, 1H, C$\underline{H}_2CO_2$Me), 4.11 (d, *J* = 18.1 Hz, 1H, C$\underline{H}_2CO_2$Me), 4.09 – 4.04 (m, 1H, CH), 3.68 (s, 3H, CO_2Me), 2.40 – 2.31 (m, 1H, CH_2 in Et), 2.28 (s, 3H, $\underline{Me}$–Ar), 1.96 – 1.86 (m, 1H, CH_2 in Et), 0.95 (t, *J* = 7.4 Hz, 3H, CH_3 in Et) ppm. ^{13}C NMR (126 MHz, $CDCl_3$) δ 173.1, 172.4, 146.7, 139.2, 138.6, 129.2, 128.4, 127.6, 127.0, 119.8, 114.0, 110.2, 67.5, 52.6, 50.4, 43.3, 22.7, 21.9, 11.8 ppm. HRMS (ESI) *m/z* calcd for $C_{21}H_{26}N_2O_3$ $[M+K]^+$ 393.1575; found: 393.1565.

4-Methoxybenzyl (*R*)-*N*-[1-(benzylamino)-1-oxobutan-2-yl]-*N*-(4-chlorophenyl)glycinate (5)

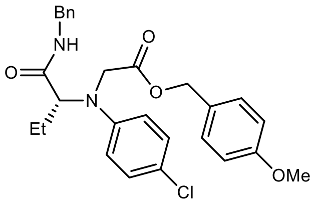

5

92 mg, 96% yield. White solid, mp 111–113 °C. >99% ee [based on the ee of **3j**]. ^{1}H NMR (500 MHz, $CDCl_3$) δ 8.56 (br, 1H, NH), 7.29 – 7.23 (comp, 3H, Ar), 7.22 (d, *J* = 8.4 Hz, 2H, Ar), 7.17 – 7.09 (comp, 4H, Ar), 6.89 (d, *J* = 8.4 Hz, 2H, Ar), 6.49 (d, *J* = 8.9 Hz, 2H, Ar), 5.07 (s, 2H, C$\underline{H}_2C_6H_4$OMe), 4.44 – 4.34 (comp, 2H, C$\underline{H}_2$Ph), 4.21 (d, *J* = 18.2 Hz, 1H, C$\underline{H}_2CO_2$PMB), 4.08 (d, *J* = 18.2 Hz, 1H, C$\underline{H}_2CO_2$PMB), 4.00 (dd, *J* = 10.0, 4.7 Hz, 1H, CH), 3.84 (s, 3H, OMe), 2.38 – 2.24 (m, 1H, CH_2 in Et), 1.98 – 1.82 (m, 1H, CH_2 in Et), 0.93 (t, *J* = 7.4 Hz, 3H, CH_3 in Et) ppm. ^{13}C NMR (126 MHz, $CDCl_3$) δ 172.2, 171.9, 160.0, 145.1, 138.5, 130.5, 129.2, 128.4, 127.6, 127.1, 126.9, 123.7, 114.3, 114.1, 67.8, 67.6, 55.3, 51.2, 43.3, 22.6, 11.7 ppm. HRMS (ESI) *m/z* calcd for $C_{27}H_{30}ClN_2O_4$ $[M+H]^+$ 481.1889; found: 481.1890.

(*R*)-*N*-Benzyl-2-([2-(benzylamino)-2-oxoethyl](4-chlorophenyl)amino)butanamide (6a)

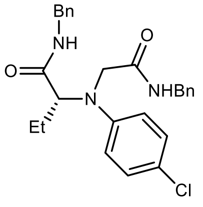

6a

84 mg, 93% yield. White solid, mp 107–108 °C. 90% ee [HPLC conditions: Chiralpak AD-H column, hexanes/*i*PrOH = 78:22, flow rate = 0.75 mL/min, wavelength = 254 nm, t_R = 6.8 min for major isomer, t_R = 11.8 min for minor isomer]. ^{1}H NMR (500 MHz, $CDCl_3$) δ 8.61 (br, 1H, NH), 7.62 (br, 1H, NH), 7.34 – 7.23 (comp, 6H, Ar), 7.19 – 7.14 (comp, 4H, Ar), 7.12 (d, *J* = 8.8 Hz, 2H, Ar), 6.56 (d, *J* = 8.8 Hz, 2H, Ar), 4.51 – 4.35 (comp, 4H, C$\underline{H}_2$Ph), 4.13 (d, *J* = 15.7 Hz, 1H, C$\underline{H}_2$CONHBn), 4.03 – 3.97 (m, 1H, CH), 3.95 (d, *J* = 15.7 Hz, 1H, C$\underline{H}_2$CONHBn), 2.17 – 2.07 (m, 1H, CH_2 in Et), 1.95 – 1.84 (m, 1H, CH_2 in Et), 0.93 (t, *J* = 7.3 Hz, 3H, CH_3 in Et) ppm. ^{13}C NMR (126 MHz, $CDCl_3$) δ 172.7, 171.0, 145.6, 138.3, 137.9, 129.2, 128.7, 128.5, 127.6, 127.5, 127.2, 123.6, 114.5, 66.9, 52.1, 43.5, 43.5, 23.3, 11.6 ppm. HRMS (ESI) *m/z* calcd for $C_{26}H_{29}ClN_3O_2$ $[M+H]^+$ 450.1943; found: 450.1949.

(*R*)-*N*-(4-bromobenzyl)-2-[(2-[(4-bromobenzyl)amino]-2-oxoethyl)(4-chlorophenyl)amino]-butanamide (6b)

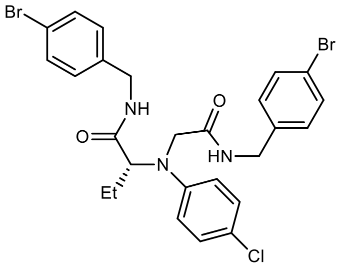

6b

103 mg, 85% yield. White solid, mp 149–150 °C. 90% ee [based on the ee of **3c**]. ^{1}H NMR (500 MHz, $CDCl_3$) δ 8.40 (br, 1H, NH), 7.43 (d, *J* = 8.3 Hz, 2H, Ar), 7.39 (d, *J* = 8.3 Hz, 2H, Ar), 7.25 (br, 1H, NH), 7.15 (d, *J* = 9.0 Hz, 2H, Ar), 7.04 (d, *J* = 8.3 Hz, 4H, Ar), 6.53 (d, *J* = 9.0 Hz, 2H, Ar), 4.47 – 4.31 (comp, 4H, C$\underline{H}_2$Ph), 4.14 (d, *J* = 17.3 Hz, 1H, C$\underline{H}_2$CONH), 4.03 – 3.91 (comp, 2H, CH and C$\underline{H}_2$CONH), 2.20 – 2.10 (m, 1H, CH_2 in Et), 2.00 – 1.86 (m, 1H, CH_2 in Et), 0.96 (t, *J* = 7.3 Hz, 3H, CH_3 in Et) ppm. ^{13}C NMR (126 MHz, $CDCl_3$) δ 172.6, 170.8, 145.4, 137.3, 136.8, 131.8, 131.6, 129.4, 129.3, 129.2, 123.8, 121.5, 121.2, 114.3, 67.2, 52.3, 43.0, 42.9, 23.2, 11.6 ppm. HRMS (ESI) *m/z* calcd for $C_{26}H_{27}Br_2ClN_3O_2$ $[M+H]^+$ 606.0153; found: 606.0143.

Methyl (*R*)-*N*-(4-chlorophenyl)-*N*-(1-[(3-[cyclohexylamino]propyl)amino]-1-oxobutan-2-yl)glycinate (7)

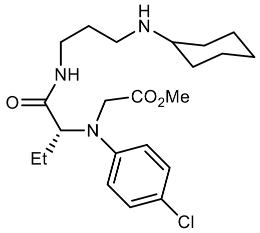

7

78 mg, 92% yield. White solid, mp 118–120 °C. 90% ee [based on the ee of **3c**]. ^{1}H NMR (500 MHz, $CDCl_3$) δ 8.86 (br, 1H, NH), 7.20 (d, *J* = 8.9 Hz, 2H, Ar), 6.58 (d, *J* = 8.9 Hz, 2H, Ar), 4.40 (d, *J* = 18.3 Hz, 1H, C$\underline{H}_2$$CO_2$Me), 4.12 (d, *J* = 18.3 Hz, 1H, C$\underline{H}_2$$CO_2$Me), 4.14 – 4.08 (m, 1H, CH), 3.83 (s, 3H, CO_2Me) 3.46 – 3.38 (m, 1H, C$\underline{H}_2$NHCO), 3.38 – 3.27 (m, 1H, C$\underline{H}_2$NHCO), 2.88 – 2.78 (comp, 2H, CyN$\underline{H}$, C$\underline{H}$NH), 2.19 – 2.00 (comp, 5H, CH_2), 1.92 – 1.76 (comp, 3H, CH_2), 1.69 – 1.63 (m, 1H, CH_2), 1.54 – 1.39 (comp, 2H, CH_2), 1.26 – 1.17 (comp, 5H, CH_2), 0.94 (t, *J* = 7.3 Hz, 3H, CH_3 in Et) ppm. ^{13}C NMR (126 MHz, $CDCl_3$) δ 173.8, 173.4, 145.2, 129.4, 123.6, 113.9, 65.8, 57.9, 53.0, 49.5, 42.5, 36.7, 28.9, 26.3, 24.8, 24.5, 22.6, 11.3 ppm. HRMS (ESI) *m/z* calcd for $C_{22}H_{35}ClN_3O_3$ $[M+H]^+$ 424.2361; found: 424.2364.

Methyl (*R*)-*N*-(1-([2-(1*H*-indol-3-yl)ethyl]amino)-1-oxobutan-2-yl)-*N*-(4-chlorophenyl)glycinate (8)

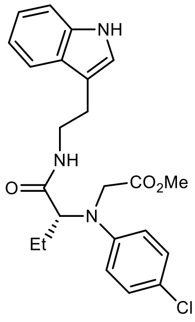

8

72 mg, 84% yield. White solid, mp 67–69 °C. 90% ee [HPLC conditions: Chiralpak AD-H column, hexanes/*i*PrOH = 80:20, flow rate = 1.0 mL/min, wavelength = 254 nm, t_R = 8.4 min for minor isomer, t_R = 10.7 min for major isomer]. ^{1}H NMR (500 MHz, $CDCl_3$) δ 8.16 (br, 1H, NHCO), 7.97 (br, 1H, NH), 7.59 (d, *J* = 8.0 Hz, 1H, Ar), 7.35 (d, *J* = 8.0 Hz, 1H, Ar), 7.19 (t, *J* = 7.5 Hz, 1H, Ar), 7.15 – 7.06 (comp, 3H, Ar), 6.81 (s, 1H, C2-$\underline{H}$), 6.46 (d, *J* = 9.0 Hz, 2H, Ar), 4.08 (d, *J* = 18.2 Hz, 1H, $C\underline{H}_2CO_2Me$), 3.99 (d, *J* = 18.2 Hz, 1H, $C\underline{H}_2CO_2Me$), 3.91 (dd, *J* = 10.2, 4.6 Hz, 1H, CH), 3.67 (s, 3H, CO_2Me), 3.66 – 3.53 (comp, 2H, $C\underline{H}_2Ar$), 2.93 (t, *J* = 7.0 Hz, 2H, $C\underline{H}_2NH$), 2.36 – 2.22 (m, 1H, CH_2 in Et), 1.88 – 1.76 (m, 1H, CH_2 in Et), 0.90 (t, *J* = 7.4 Hz, 3H, CH_3 in Et) ppm. ^{13}C NMR (126 MHz, $CDCl_3$) δ 172.6, 171.8, 145.3, 136.2, 129.1, 127.5, 123.5, 121.9, 119.2, 118.7, 114.2, 113.1, 111.1, 67.7, 52.7, 50.3, 40.0, 24.9, 22.46, 11.7 ppm. HRMS (ESI) *m/z* calcd for $C_{23}H_{27}ClN_3O_3$ $[M+H]^+$ 428.1735; found: 428.1746.

Methyl (*R*)-*N*-(1-[(2-(1H-indol-3-yl)ethyl]amino)-1-oxodecan-2-yl)-*N*-(4-chlorophenyl)glycinate (9)

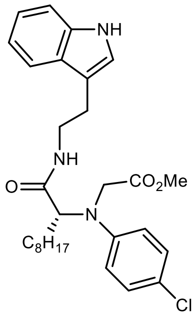

9

90 mg, 88% yield. White solid, mp 108–109 °C. 97% ee [HPLC conditions: Chiralpak AD-H column, hexanes/*i*PrOH = 80:20, flow rate = 1.0 mL/min, wavelength = 254 nm, t_R = 5.3 min for minor isomer, t_R = 7.5 min for major isomer]. ^{1}H NMR (300 MHz, $CDCl_3$) δ 8.18 (br, 1H, N$\underline{H}$CO), 7.99 (br, 1H, NH), 7.59 (d, *J* = 8.0 Hz, 1H, Ar), 7.35 (d, *J* = 8.0 Hz, 1H, Ar), 7.23 – 7.16 (m, 1H, Ar), 7.11 (d, *J* = 9.0 Hz, 2H), 7.14 – 7.06 (m, 1H, Ar), 6.81 (s, 1H, C2-$\underline{H}$), 6.45 (d, *J* = 9.0 Hz, 2H), 4.01 (dd, *J* = 8.6, 1.0 Hz, 2H, $C\underline{H}_2CO_2Me$), 3.98 – 3.91 (m, 1H, CH), 3.68 (s, 3H, CO_2Me), 3.66 – 3.50 (comp, 2H, $C\underline{H}_2Ar$), 2.93 (t, *J* = 7.0 Hz, 2H, $C\underline{H}_2NH$), 2.31 – 2.17 (m, 1H, $C\underline{H}_2CH$), 1.83 – 1.67 (m, 1H, $C\underline{H}_2CH$), 1.32 – 1.16 (comp, 12H, CH_2 in C_8H_{17}), 0.89 (t, *J* = 6.7 Hz, 3H, CH_3 in C_8H_{17}) ppm. ^{13}C NMR (126 MHz, $CDCl_3$) δ 172.6, 172.0, 145.3, 136.2, 129.2, 127.5, 123.5, 121.9, 119.2, 118.8, 114.2, 113.1, 111.1, 66.2, 52.7, 50.2, 40.1, 31.8, 29.7, 29.5, 29.3, 29.2, 27.1, 24.9, 22.6, 14.1 ppm. HRMS (ESI) *m/z* calcd for $C_{29}H_{39}ClN_3O_3$ $[M+H]^+$ 512.2674; found: 512.2696.

Benzyl (*R*)-(2-[(4-chlorophenyl)(2-methoxy-2-oxoethyl)amino]butanoyl)-*L*-prolinate (10)

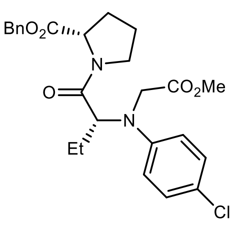

10

83 mg, 88% yield. Colorless oil. Single enantiomer (>99% ee). ^{1}H NMR (500 MHz, $CDCl_3$) δ 7.44 – 7.30 (comp, 5H, Ar in Bn), 7.20 (d, *J* = 8.9 Hz, 2H, Ar), 6.59 (d, *J* = 8.9 Hz, 2H, Ar), 5.24 (d, *J* = 12.3 Hz, 1H, CH_2 in Bn), 5.13 (d, *J* = 12.3 Hz, 1H, CH_2 in Bn), 4.47 – 4.39 (comp, 2H, C$\underline{H}$N, C$\underline{H}$Et), 4.25 (d, *J* = 18.3 Hz, 1H, $C\underline{H}_2CO_2Me$), 3.90 (d, *J* = 18.3 Hz, 1H, $C\underline{H}_2CO_2Me$), 3.71 (s, 3H, CO_2Me), 3.66 – 3.60 (m, 1H, $C\underline{H}_2N$), 3.59 – 3.54 (m, 1H, $C\underline{H}_2N$), 2.29 – 2.15 (m, 1H, CH_2), 2.05 – 1.90 (comp, 4H, CH_2), 1.71 – 1.63 (m, 1H, CH_2), 0.94 (t, *J* = 7.4 Hz, 3H, CH_3 in Et) ppm. ^{13}C NMR (126 MHz, $CDCl_3$) δ 172.0, 171.3, 168.9, 146.8, 135.7, 129.4, 128.5, 128.2,

128.1, 122.7, 113.2, 66.8, 59.9, 59.4, 52.0, 47.4, 46.9, 29.2, 24.8, 21.7, 10.5 ppm. HRMS (ESI) *m/z* calcd for $C_{25}H_{30}ClN_2O_5$ $[M+H]^+$ 473.1838; found: 473.1851.

Methyl (*R*)-*N*-(4-chlorophenyl)-*N*-[1-([(*R*)-2-hydroxy-1-phenylethyl]amino)-1-oxobutan-2-yl]glycinate (11)

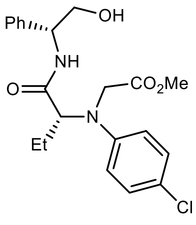

11

73 mg, 90% yield. Colorless oil. Single enantiomer (>99% ee). ^{1}H NMR (500 MHz, $CDCl_3$) δ 8.80 (br, 1H, NH), 7.26 – 7.18 (comp, 3H, Ph), 7.12 (d, *J* = 8.6 Hz, 2H, Ar), 7.09 – 7.01 (comp, 2H, Ph), 6.48 (d, *J* = 8.6 Hz, 2H, Ar), 5.06 (dd, *J* = 11.7, 6.6 Hz, 1H, C$\underline{H}$NH), 4.32 (d, *J* = 18.4 Hz, 1H, C$\underline{H}_2$CO$_2$Me), 4.11 (d, *J* = 18.4 Hz, 1H, C$\underline{H}_2$CO$_2$Me), 3.98 (dd, *J* = 9.7, 4.6 Hz, 1H, CH), 3.81 (s, 3H), 3.80 – 3.75 (comp, 2H, C$\underline{H}_2$OH), 2.69 (br, 1H, OH), 2.32 – 2.19 (m, 1H, CH_2 in Et), 1.98 – 1.85 (m, 1H, CH_2 in Et), 0.94 (t, *J* = 7.2 Hz, 3H, CH_3 in Et) ppm. ^{13}C NMR (126 MHz, $CDCl_3$) δ 173.4, 172.1, 144.3, 138.9, 129.2, 128.4, 127.5, 126.9, 123.8, 114.3, 68.4, 66.4, 56.2, 53.0, 52.5, 22.0, 11.6 ppm. HRMS (ESI) *m/z* calcd for $C_{21}H_{26}ClN_2O_4$ $[M+H]^+$ 405.1576; found: 405.1585.

Methyl (*R*)-*N*-[1-(*tert*-butylamino)-1-oxobutan-2-yl]-*N*-(4-chlorophenyl)glycinate (12)

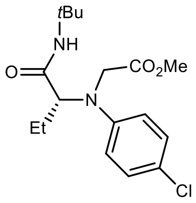

12

63 mg, 92% yield. White solid, mp 82–83 °C. 90% ee [based on the ee of **3c**]. ^{1}H NMR (500 MHz, $CDCl_3$) δ 7.85 (br, 1H, NH), 7.18 (d, *J* = 9.0 Hz, 2H, Ar), 6.53 (d, *J* = 9.0 Hz, 2H, Ar), 4.22 (d, *J* = 18.3 Hz, 1H, C$\underline{H}_2$CO$_2$Me), 4.04 (d, *J* = 18.3 Hz, 1H, C$\underline{H}_2$CO$_2$Me), 3.85 – 3.80 (m, 1H, CH), 3.82 (s, 3H, CO_2Me), 2.31 – 2.18 (m, 1H, CH_2 in Et), 1.93 – 1.82 (m, 1H, CH_2 in Et), 1.29 (s, 9H, *t*Bu), 0.91 (t, *J* = 7.4 Hz, 3H, CH_3 in Et). ^{13}C NMR (126 MHz, $CDCl_3$) δ 172.6, 170.8, 144.8, 129.1, 123.5, 114.3, 68.6, 52.7, 51.6, 50.9, 28.4, 21.9, 11.7. HRMS (ESI) *m/z* calcd for $C_{17}H_{26}ClN_2O_3$ $[M+H]^+$ 341.1626; found: 341.1633.

Methyl (*R*)-*N*-(4-chlorophenyl)-*N*-[1-oxo-1-(phenylamino)butan-2-yl]glycinate (13a)

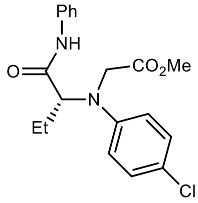

13a

47 mg, 65% yield. White solid, mp 117–118 °C. 90% ee [based on the ee of **3c**]. ^{1}H NMR (500 MHz, $CDCl_3$) δ 10.21 (s, 1H, NH), 7.66 (d, *J* = 7.8 Hz, 2H, Ar), 7.31 (t, *J* = 7.8 Hz, 2H, Ar), 7.21 (d, *J* = 8.9 Hz, 2H, Ar), 7.09 (t, *J* = 7.8 Hz, 1H, Ar), 6.62 (d, *J* = 8.9 Hz, 2H, Ar), 4.33 (d, *J* = 18.3 Hz, 1H, C$\underline{H}_2$CO$_2$Me), 4.17 (d, *J* = 18.3 Hz, 1H, C$\underline{H}_2$CO$_2$Me), 4.08 (dd, *J* = 10.1, 4.6 Hz, 1H, CH), 3.90 (s, 3H, CO_2Me), 2.47 – 2.30 (m, 1H, CH_2 in Et), 2.04 – 1.91 (m, 1H, CH_2 in Et), 0.99 (t, *J* = 7.3 Hz, 3H, CH_3 in Et) ppm. ^{13}C NMR (126 MHz, $CDCl_3$) δ 173.4, 170.4, 144.8, 138.3, 129.4, 128.9, 124.1, 124.0, 119.6, 114.2, 68.7, 53.1, 50.9, 22.3, 11.7 ppm. HRMS (ESI) *m/z* calcd for $C_{19}H_{22}ClN_2O_3$ $[M+H]^+$ 361.1313; found: 361.1328.

Methyl (*R*)-*N*-(4-chlorophenyl)-N-(1-[(4-(dimethylamino)phenyl)amino]-1-oxobutan-2-yl)glycinate (13b)

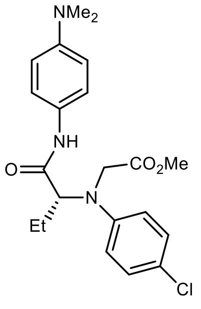

13b

Reaction was performed in nitromethane. 75 mg, 93% yield. Brown solid, mp 145–146 °C. 90% ee [based on the ee of **3c**]. ^{1}H NMR (500 MHz, $CDCl_3$) δ 9.91 (br, 1H, NH), 7.48 (d, *J* = 9.1 Hz, 2H, Ar), 7.18 (d, *J* = 9.1 Hz, 2H, Ar), 6.69 (d, *J* = 9.1 Hz, 2H, Ar), 6.59 (d, *J* = 9.1 Hz, 2H, Ar), 4.28 (d, *J* = 18.3 Hz, 1H, $C\underline{H}_2CO_2Me$), 4.13 (d, *J* = 18.3 Hz, 1H, $C\underline{H}_2CO_2Me$), 4.05 (dd, *J* = 10.3, 4.7 Hz, 1H, CH), 3.85 (s, 3H, CO_2Me), 2.89 (s, 6H, NMe_2), 2.45 – 2.33 (m, 1H, CH_2 in Et), 1.98 – 1.88 (m, 1H, CH_2 in Et), 0.95 (t, *J* = 7.4 Hz, 3H, CH_3 in Et) ppm. ^{13}C NMR (126 MHz, $CDCl_3$) δ 173.2, 169.6, 147.8, 145.0, 129.4, 128.5, 123.9, 121.0, 114.3, 113.2, 68.5, 53.0, 50.7, 41.0, 22.4, 11.7 ppm. HRMS (ESI) *m/z* calcd for $C_{21}H_{27}ClN_3O_3$ $[M+H]^+$ 404.1735; found: 404.1742.

Diethyl (*R*)-(2-[(4-chlorophenyl)(2-methoxy-2-oxoethyl)amino]butanoyl)-*L*-glutamate (14)

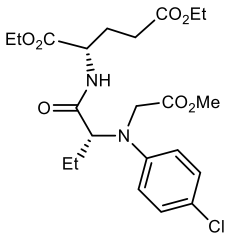

14

79 mg, 84% yield. Colorless oil. Single enantiomer (>99% ee). ^{1}H NMR (500 MHz, $CDCl_3$) δ 8.52 (d, *J* = 7.9 Hz, 1H, $N\underline{H}CO$), 7.18 (d, *J* = 9.1 Hz, 2H, Ar), 6.55 (d, *J* = 9.1 Hz, 2H, Ar), 4.56 – 4.50 (m, 1H, $C\underline{H}NH$), 4.25 (d, *J* = 18.3 Hz, 1H, $C\underline{H}_2CO_2Me$), 4.18 – 4.07 (comp, 5H, $CO_2C\underline{H}_2Me$, $C\underline{H}_2CO_2Me$), 3.96 (dd, *J* = 10.0, 4.9 Hz, 1H, CH), 3.84 (s, 3H, CO_2Me), 2.32 – 2.23 (m, 1H, CH_2), 2.15 – 2.07 (comp, 3H, CH_2), 1.97 – 1.87 (comp, 2H, CH_2), 1.25 (t, *J* = 7.1 Hz, 6H, $CO_2CH_2\underline{Me}$), 0.94 (t, *J* = 7.4 Hz, 3H, CH_3 in Et) ppm. ^{13}C NMR (126 MHz, $CDCl_3$) δ 172.8, 172.5, 171.9, 171.5, 144.8, 129.2, 123.7, 114.3, 68.0, 61.3, 60.5, 52.9, 52.0, 51.8, 30.0, 26.8, 22.2, 14.2, 14.1, 11.6 ppm. HRMS (ESI) *m/z* calcd for $C_{22}H_{32}ClN_2O_7$ $[M+H]^+$ 471.1893; found: 471.1903.

***(R)-N*-(3-[(4-[(3-aminopropyl)amino]butyl)amino]propyl)-2-((2-((3-((4-((3-aminopropyl)-amino)butyl)amino)propyl)amino)-2-oxoethyl)(4-chlorophenyl)amino)butanamide (15)**

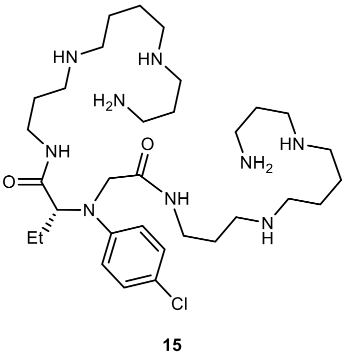

15

80 mg, 63% yield. White solid, mp 33–34 °C. 90% ee [based on the ee of **3c**]. ^{1}H NMR (500 MHz, $CDCl_3$) δ 9.07 (br, 1H, NHCO), 8.59 (br, 1H, NHCO), 7.10 (d, *J* = 8.9 Hz, 2H, Ar), 6.52 (d, *J* = 8.9 Hz, 2H, Ar), 4.05 (d, *J* = 17.3 Hz, 1H, $C\underline{H}_2CO_2Me$), 3.98 – 3.81 (comp, 2H, $C\underline{H}Et$, $C\underline{H}_2CO_2Me$), 3.37 – 3.22 (comp, 4H, alkyl), 2.74 (t, *J* = 6.5 Hz, 4H, alkyl), 2.71 – 2.27 (comp, 23H, alkyl, NH), 2.23 – 2.17 (m, 1H, alkyl), 2.16 – 2.01 (m, 1H, $C\underline{H}_2$ in Et), 1.91 – 1.79 (m, 1H, $C\underline{H}_2$ in Et), 1.70 – 1.57 (comp, 8H, alkyl, NH), 1.54 – 1.40 (comp, 8H, alkyl, NH), 0.91 (t, *J* = 7.1 Hz, 3H, CH_3 in Et) ppm. ^{13}C NMR (126 MHz, $CDCl_3$) δ 172.5, 170.8, 145.8, 128.9, 122.8, 114.1, 70.0, 66.9, 55.4, 53.4, 53.1, 51.3, 49.7, 49.6, 49.5, 47.8, 47.6, 47.5, 45.2, 40.5, 38.4, 38.2, 33.1, 33.0, 28.8, 28.5, 27.7, 24.7, 23.1, 11.7 ppm. HRMS (ESI) *m/z* calcd for $C_{32}H_{63}ClN_9O_2$ $[M+H]^+$ 640.4788; found: 640.4779.

Methyl (*R*)-*N*6-(2-[(4-chlorophenyl)(2-methoxy-2-oxoethyl)amino]butanoyl)-*L*-lysinate (16)

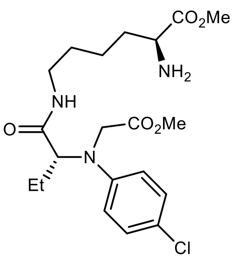

16

77 mg, 90% yield. Colorless oil. Single enantiomer (>99% ee). ^{1}H NMR (500 MHz, $CDCl_3$) δ 8.23 (br, 1H, $N\underline{H}CO$), 7.18 (d, *J* = 9.0 Hz, 2H, Ar), 6.52 (d, *J* = 9.0 Hz, 2H, Ar), 4.23 (d, *J* = 18.3 Hz, 1H, $C\underline{H}_2CO_2Me$), 4.08 (d, *J* = 18.3 Hz, 1H, $C\underline{H}_2CO_2Me$), 3.93 (dd, *J* = 10.2, 4.7 Hz, 1H, CH), 3.83 (s, 3H, CO_2Me), 3.71 (s, 3H, CO_2Me), 3.42 – 3.36 (m, 1H, $C\underline{H}NH_2$) 3.22 (dd, *J* = 12.8, 6.7 Hz, 2H, $C\underline{H}_2NH$), 2.35 – 2.22 (m, 1H, CH_2), 2.02 – 1.81 (comp, 3H, NH_2, CH_2), 1.58 – 1.39 (comp, 3H), 1.37 – 1.23 (comp, 3H), 0.92 (t, *J* = 7.4 Hz, 3H, CH_3 in Et) ppm. ^{13}C NMR (126 MHz, $CDCl_3$) δ 176.1, 173.2, 171.8, 145.0, 129.2, 123.6, 114.0, 67.7, 54.2, 52.9, 52.0, 50.6, 39.1, 34.3, 28.9, 22.8, 17.8, 11.7 ppm. HRMS (ESI) *m/z* calcd for $C_{20}H_{31}ClN_3O_5$ $[M+H]^+$ 428.1947; found: 428.1955.

(*S*)-2-Ammonio-6-[(*R*)-2-[(carboxymethyl)(4-chlorophenyl)amino]butanamido]hexanoate (17)

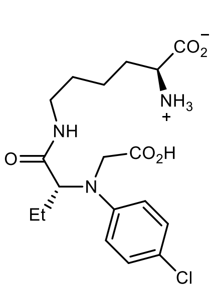

17

60 mg, 75% yield. White solid, mp 183–184 °C. Single enantiomer (>99% ee). ^{1}H NMR (300 MHz, CD_3OD) δ 7.13 (d, *J* = 8.7 Hz, 2H, Ar), 6.57 (dd, *J* = 8.7, 4.8 Hz, 2H, Ar), 4.18 (d, *J* = 17.6 Hz, 1H, $C\underline{H}_2CO_2H$), 4.08 – 3.95 (m, 1H, CH), 3.90 (d, *J* = 17.6 Hz, 1H, $C\underline{H}_2CO_2H$), 3.46 (dd, *J* = 7.8, 1.9 Hz, 1H, $CHNH_3^+$), 3.31 – 3.20 (m, 1H, $C\underline{H}_2NH$), 3.19 – 3.02 (m, 1H, $C\underline{H}_2NH$), 2.22 – 1.85 (comp, 3H, CH_2), 1.80 – 1.65 (m, 1H, CH_2), 1.56 – 1.42 (comp, 2H, CH_2), 1.41 – 1.31 (comp, 2H, CH_2), 0.97 (t, *J* = 7.4 Hz, 3H, CH_3 in Et) ppm. ^{13}C NMR (126 MHz, CD_3OD) δ 174.4, 172.9, 146.3, 145.8, 128.2, 121.4, 113.3, 68.2, 66.9, 54.1, 38.1, 37.9, 27.4, 22.9, 22.1, 10.8 ppm. HRMS (ESI) *m/z* calcd for $C_{18}H_{27}ClN_3O_5$ $[M+H]^+$ 400.1634; found: 400.1640.

(*R*)-*N*-[1-(*tert*-Butylamino)-1-oxobutan-2-yl]-*N*-(4-chlorophenyl)glycine (18)

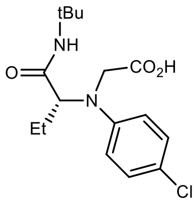

18

52 mg, 80% yield. White solid, mp 206–207 °C. 90% ee [based on the ee of **3c**]. ^{1}H NMR (500 MHz, $CDCl_3$) δ 7.23 (br, 1H, NH), 7.16 (d, *J* = 8.0 Hz, 2H, Ar), 6.57 (d, *J* = 8.0 Hz, 2H, Ar), 4.21 (d, *J* = 17.8 Hz, 1H, $C\underline{H}_2CO_2H$), 4.07 (d, *J* = 17.8 Hz, 1H, $C\underline{H}_2CO_2H$), 3.95 (dd, *J* = 9.1, 4.5 Hz, 1H, CH), 2.15 – 2.00 (m, 1H, CH_2 in Et), 1.99 – 1.86 (m, 1H, CH_2 in Et), 1.33 (s, 9H, *t*Bu), 0.96 (t, *J* = 7.0 Hz, 3H, CH_3 in Et) ppm. ^{13}C NMR (126 MHz, $CDCl_3$) δ 174.6, 173.4, 145.1, 129.2, 123.7, 114.8, 113.8, 66.5, 52.1, 28.4, 23.4, 11.5 ppm; IR (neat) 3295 (NH), 2967 (OH), 1720 ($\underline{CO}OH$), 1598 ($\underline{CO}NH$), 1496, 1220, 1187, 1094, 812, 737 cm^{-1}; HRMS (ESI) *m/z* calcd for $C_{16}H_{24}ClN_2O_3$ $[M+H]^+$ 327.1470; found: 327.1482.

(*R*)-*N*-(4-Chlorophenyl)-*N*-[1-oxo-1-(pyrrolidin-1-yl)butan-2-yl]glycine (19)

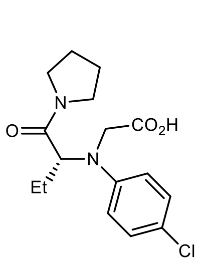

19

47 mg, 72% yield. White solid, mp 235–237 °C. 90% ee [based on the ee of **3c**]. ^{1}H NMR (500 MHz, $CDCl_3$) δ 7.21 (d, *J* = 8.9 Hz, 2H, Ar), 6.52 (d, *J* = 8.9 Hz, 2H, Ar), 4.28 (dd, *J* = 9.8, 4.8 Hz, 1H, CH), 4.22 (d, *J* = 17.6 Hz, 1H, $C\underline{H}_2CO_2H$), 4.13 (d, *J* = 17.6 Hz, 1H, $C\underline{H}_2CO_2H$), 3.76 (dt, *J* = 10.0, 6.7 Hz, 1H, $C\underline{H}_2NCO$), 3.68 – 3.57 (comp, 2H, $C\underline{H}_2NCO$), 3.51 (dt, *J* = 10.0, 6.7 Hz, 1H, $C\underline{H}_2NCO$), 2.20 – 2.09 (comp, 2H, CH_2), 2.02 – 1.90 (comp, 4H, CH_2), 1.05 (t, *J* = 7.3 Hz, 3H, CH_3 in Et)

ppm. ^{13}C NMR (126 MHz, $CDCl_3$) δ 173.6, 173.5, 145.8, 129.6, 123.9, 113.0, 61.8, 48.5, 47.0, 46.6, 26.4, 23.8, 22.9, 11.5 ppm; IR (neat) 2970 (OH), 1730 (<u>CO</u>OH), 1591 (<u>CO</u>N), 1496, 1380, 1188, 952, 810, 735 cm^{-1}; HRMS (ESI) *m/z* calcd for $C_{16}H_{22}ClN_2O_3$ $[M+H]^+$ 325.1313; found: 325.1324.

Methyl (*R*)-2-[(4-chlorophenyl)(2-methoxy-2-oxoethyl)amino]butanoate (20)

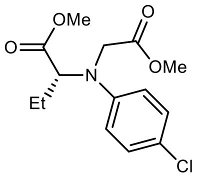

20

54 mg, 91% yield. Colorless oil. 90% ee [HPLC conditions: Chiralpak AD-H column, hexanes/*i*PrOH = 97:3, flow rate = 1.0 mL/min, wavelength = 254 nm, t_R = 9.0 min for major isomer, t_R = 10.1 min for minor isomer]. ^1H NMR (500 MHz, $CDCl_3$) δ 7.19 (d, *J* = 9.0 Hz, 2H, Ar), 6.70 (d, *J* = 9.0 Hz, 2H, Ar), 4.26 (t, *J* = 7.5 Hz, 1H, CH), 4.22 (d, *J* = 18.5 Hz, 1H, C<u>H</u>$_2CO_2$Me), 4.07 (d, *J* = 18.5 Hz, 1H, C<u>H</u>$_2CO_2$Me), 3.76 (s, 3H, CO_2Me), 3.73 (s, 3H, CO_2Me), 2.01 – 1.92 (m, 1H, CH_2 in Et), 1.91 – 1.82 (m, 1H, CH_2 in Et), 1.03 (t, *J* = 7.4 Hz, 3H, CH_3 in Et) ppm. ^{13}C NMR (126 MHz, $CDCl_3$) δ 172.8, 171.6, 147.3, 129.1, 123.6, 114.9, 62.7, 52.2, 52.0, 49.0, 23.8, 10.9 ppm. HRMS (ESI) *m/z* calcd for $C_{14}H_{19}ClNO_4$ $[M+H]^+$ 300.0997; found: 300.0999.

Methyl (*R*)-2-[(4-chlorophenyl)(2-[(4-methoxybenzyl)oxy]-2-oxoethyl)amino]butanoate (21)

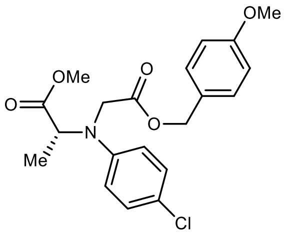

21

72 mg, 92% yield. White solid, mp 154–156 °C. 88% ee [based on the ee of **3m**]. ^1H NMR (500 MHz, $CDCl_3$) δ 7.29 (d, *J* = 8.5 Hz, 2H, Ar), 7.16 (d, *J* = 8.9 Hz, 2H), 6.91 (d, *J* = 8.5 Hz, 2H), 6.59 (d, *J* = 8.9 Hz, 2H), 5.15 (s, 2H, C<u>H</u>$_2$Ar), 4.47 (dd, *J* = 9.8, 7.2 Hz, 1H, CH), 4.21 (d, *J* = 18.5 Hz, 1H, C<u>H</u>$_2CO_2$Me), 4.07 (d, *J* = 18.5 Hz, 1H, C<u>H</u>$_2CO_2$PMB), 3.84 (s, 3H, OMe), 3.71 (s, 3H, OMe), 1.51 (d, *J* = 7.2 Hz, 3H, <u>Me</u>CH) ppm. ^{13}C NMR (126 MHz, $CDCl_3$) δ 173.6, 171.4, 159.8, 146.9, 130.2, 129.1, 127.6, 123.5, 114.6, 114.0, 66.8, 56.3, 55.3, 52.2, 49.7, 16.2 ppm. HRMS (ESI) *m/z* calcd for $C_{20}H_{23}ClNO_5$ $[M+H]^+$ 392.1259; found: 392.1268.

Methyl (*R*)-2-[(4-chlorophenyl)(2-oxo-2-[(3,4,5-trimethoxybenzyl)oxy]ethyl)amino]butanoate (22)

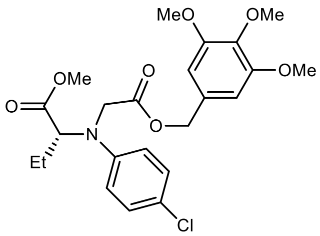

22

82 mg, 88% yield. White solid, mp 188–190 °C. 98% ee [based on the ee of **3k**]. ^1H NMR (500 MHz, $CDCl_3$) δ 7.16 (d, *J* = 9.0 Hz, 2H, Ar), 6.69 (d, *J* = 9.0 Hz, 2H, Ar), 6.55 (s, 2H, Ar), 5.12 (s, 2H, C<u>H</u>$_2$Ar), 4.29 – 4.22 (comp, 2H, C<u>H</u>$_2CO_2$R, CH), 4.13 (d, *J* = 18.4 Hz, 1H, C<u>H</u>$_2CO_2$R), 3.87 (s, 3H, OMe), 3.85 (s, 6H, OMe), 3.67 (s, 3H, OMe), 2.01 – 1.91 (m, 1H, CH_2 in Et), 1.90 – 1.80 (m, 1H, CH_2 in Et), 1.00 (t, *J* = 7.4 Hz, 3H, CH_3 in Et) ppm. ^{13}C NMR (126 MHz, $CDCl_3$) δ 172.7, 170.9, 153.3, 147.2, 138.0, 131.0, 129.1, 123.6, 114.8, 105.4, 67.1, 62.6, 60.9, 56.1, 51.9, 49.0, 23.8, 10.9 ppm. HRMS (ESI) *m/z* calcd for $C_{23}H_{29}ClNO_7$ $[M+H]^+$ 466.1627; found: 466.1635.

Ethyl (*R*)-2-[(4-chlorophenyl)(2-methoxy-2-oxoethyl)amino]butanoate (23)

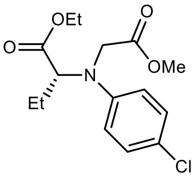

23

42 mg, 66% yield. Colorless oil. 90% ee [HPLC conditions: Chiralpak AD-H column, hexanes/*i*PrOH = 97:3, flow rate = 1.0 mL/min, wavelength = 254 nm, t_R = 7.7 min for major isomer, t_R = 8.9 min for minor isomer]. ^{1}H NMR (500 MHz, $CDCl_3$) δ 7.19 (d, *J* = 9.1 Hz, 2H, Ar), 6.70 (d, *J* = 9.1 Hz, 2H, Ar), 4.27 – 4.21 (comp, 2H, CH, C<u>H</u>$_2$CO$_2$Me), 4.18 (q, *J* = 7.1 Hz, 2H, CO$_2$C<u>H</u>$_2$Me), 4.07 (d, *J* = 18.4 Hz, 1H, C<u>H</u>$_2$CO$_2$Me), 3.76 (s, 3H, CO$_2$Me), 2.02 – 1.92 (m, 1H, CH$_2$ in EtCH), 1.92 – 1.81 (m, 1H, CH$_2$ in EtCH), 1.27 (t, *J* = 7.1 Hz, 3H, CO$_2$CH$_2$<u>Me</u>), 1.04 (t, *J* = 7.4 Hz, 3H, CH$_3$ in EtCH) ppm. ^{13}C NMR (126 MHz, $CDCl_3$) δ 172.4, 171.7, 147.4, 129.0, 123.5, 114.9, 62.9, 61.0, 52.1, 49.1, 23.9, 14.2, 11.0 ppm. HRMS (ESI) *m/z* calcd for $C_{15}H_{21}ClNO_4$ [M+H]$^+$ 314.1154; found: 314.1160.

(*R*)-3,7-Dimethylocta-2,6-dien-1-yl 2-[(4-chlorophenyl)(2-methoxy-2-oxoethyl)amino]-butanoate (24)

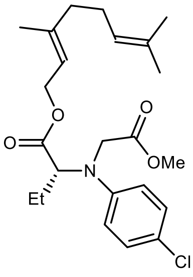

24

52 mg, 62% yield. Colorless oil with lime odor. 90% ee [based on the ee of **3c**]. ^{1}H NMR (500 MHz, $CDCl_3$) δ 7.18 (d, *J* = 8.8 Hz, 2H, Ar), 6.70 (d, *J* = 8.8 Hz, 2H, Ar), 5.33 (t, *J* = 6.4 Hz, 1H, =CH), 5.09 (t, *J* = 5.9 Hz, 1H, =CH), 4.69 – 4.58 (comp, 2H, C<u>H</u>$_2$O), 4.28 – 4.20 (comp, 2H, CH, C<u>H</u>$_2$CO$_2$Me), 4.06 (d, *J* = 18.4 Hz, 1H, C<u>H</u>$_2$CO$_2$Me), 3.76 (s, 3H, CO$_2$Me), 2.14 – 2.02 (comp, 4H, CH$_2$), 2.00 – 1.93 (m, 1H, CH$_2$), 1.91 – 1.83 (m, 1H, CH$_2$), 1.70 (s, 3H, <u>Me</u>–C=, terminal), 1.69 (s, 3H, <u>Me</u>–C=, terminal), 1.62 (s, 3H, <u>Me</u>–C=, internal), 1.04 (t, *J* = 7.3 Hz, 3H, CH$_3$ in Et) ppm. ^{13}C NMR (126 MHz, $CDCl_3$) δ 172.5, 171.7, 147.5, 143.0, 131.9, 129.0, 123.6, 123.5, 117.8, 115.0, 62.9, 61.8, 52.1, 49.2, 39.5, 26.3, 25.7, 24.0, 17.7, 16.5, 11.0 ppm. HRMS (ESI) *m/z* calcd for $C_{23}H_{33}ClNO_4$ [M+H]$^+$ 422.2093; found: 422.2099.

(*R*)-2-Hydroxyethyl 2-[(4-chlorophenyl)(2-methoxy-2-oxoethyl)amino]butanoate (25)

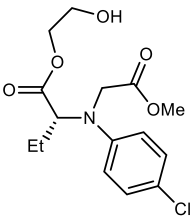

25

32 mg, 48% yield. Colorless oil. 90% ee [based on the ee of **3c**]. ^{1}H NMR (500 MHz, $CDCl_3$) δ 7.20 (d, *J* = 9.0 Hz, 2H, Ar), 6.72 (d, *J* = 9.0 Hz, 2H, Ar), 4.35 – 4.27 (comp, 2H, C<u>H</u>$_2$O), 4.24 – 4.20 (m, 1H, CH), 4.18 (d, *J* = 18.4 Hz, 1H, C<u>H</u>$_2$CO$_2$Me), 4.12 (d, *J* = 18.4 Hz, 1H, C<u>H</u>$_2$CO$_2$Me), 3.79 (t, *J* = 4.5 Hz, 2H, C<u>H</u>$_2$OH), 3.76 (s, 3H, CO$_2$Me), 2.09 – 1.95 (comp, 2H, OH, CH$_2$ in Et), 1.93 – 1.82 (m, 1H, CH$_2$ in Et), 1.02 (t, *J* = 7.4 Hz, 3H, CH$_3$ in Et) ppm. ^{13}C NMR (126 MHz, $CDCl_3$) δ 172.3, 171.6, 147.0, 129.2, 123.7, 114.8, 66.7, 62.9, 60.9, 52.3, 48.6, 23.5, 10.8 ppm. HRMS (ESI) *m/z* calcd for $C_{15}H_{21}ClNO_5$ [M+H]$^+$ 330.1103; found: 330.1106.

(*R*)-2-[(4-Chlorophenyl)(2-methoxy-2-oxoethyl)amino]butanoic acid (26)

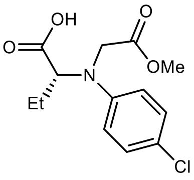

26

40 mg, 70% yield. Colorless oil. 90% ee [based on the ee of **3c**]. ^{1}H NMR (500 MHz, $CDCl_3$) δ 7.22 (d, *J* = 8.9 Hz, 2H, Ar), 6.61 (d, *J* = 8.9 Hz, 2H, Ar), 4.30 (d, *J* = 18.5 Hz, 1H, C<u>H</u>$_2$CO$_2$Me), 4.14 (d, *J* = 18.5 Hz, 1H, C<u>H</u>$_2$CO$_2$Me), 4.07 (dd, *J* = 8.0, 6.6 Hz, 1H, CH), 3.87 (s, 3H, CO$_2$Me), 2.20 – 2.10 (m, 1H, CH$_2$ in Et), 2.04 – 1.94 (m, 1H, CH$_2$ in Et), 1.04 (t, *J* = 7.4 Hz, 3H, CH$_3$ in Et) ppm. ^{13}C NMR (126 MHz,

$CDCl_3$) δ 174.4, 174.2, 144.7, 129.5, 124.4, 114.4, 66.0, 53.3, 51.1, 23.0, 11.3 ppm. HRMS (ESI) *m/z* calcd for $C_{13}H_{17}ClNO_4$ $[M+H]^+$ 286.0841; found: 286.0846.

Methyl (*R*)-*N*-(4-chlorophenyl)-*N*-[1-oxo-1-(2-phenylhydrazineyl)butan-2-yl]glycinate (27a)

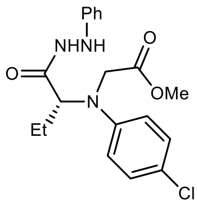

27a

65 mg, 87% yield. White solid, mp 178–180 °C. 90% ee [based on the ee of **3c**]. ^1H NMR (500 MHz, $CDCl_3$) δ 10.18 (s, 1H, N$\underline{H}$CO), 7.23 (d, *J* = 9.0 Hz, 2H, Ar), 7.14 (t, *J* = 7.9 Hz, 2H, Ar), 6.87 (t, *J* = 7.3 Hz, 1H, Ar), 6.64 – 6.59 (comp, 4H, Ar), 6.15 (br, 1H, N$\underline{H}$Ph), 4.33 (d, *J* = 18.4 Hz, 1H, C$\underline{H}_2$CO$_2$Me), 4.16 (d, *J* = 18.4 Hz, 1H, C$\underline{H}_2$CO$_2$Me), 4.09 (dd, *J* = 9.9, 5.1 Hz, 1H, CH), 3.87 (s, 3H, CO$_2$Me), 2.34 – 2.24 (m, 1H, CH_2 in Et), 2.03 – 1.93 (m, 1H, CH_2 in Et), 1.00 (t, *J* = 7.4 Hz, 3H, CH_3 in Et) ppm. ^{13}C NMR (126 MHz, $CDCl_3$) δ 173.7, 171.5, 148.1, 144.8, 129.4, 129.0, 124.1, 121.1, 114.3, 113.9, 66.5, 53.2, 50.9, 22.7, 11.5 ppm. HRMS (ESI) *m/z* calcd for $C_{19}H_{23}ClN_3O_3$ $[M+H]^+$ 376.1422; found: 376.1424.

Methyl *(R)*-*N*-(4-chlorophenyl)-*N*-(1-[2-(4-nitrophenyl)hydrazineyl]-1-oxobutan-2-yl)glycinate (27b)

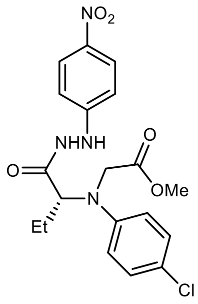

27b

Reaction was performed in nitromethane. 74 mg, 88% yield. Yellow solid, mp 205–206 °C. 90% ee [based on the ee of **3c**]. ^1H NMR (500 MHz, $CDCl_3$) δ 10.45 (d, *J* = 3.1 Hz, 1H, NHCO), 7.98 (d, *J* = 9.1 Hz, 2H, Ar), 7.25 (d, *J* = 9.1 Hz, 2H, Ar), 6.71 (br, 1H, NHAr), 6.59 (d, *J* = 9.1 Hz, 2H, Ar), 6.51 (d, *J* = 9.1 Hz, 2H, Ar), 4.38 (d, *J* = 18.6 Hz, 1H, C$\underline{H}_2$CO$_2$Me), 4.18 (d, *J* = 18.6 Hz, 1H, C$\underline{H}_2$CO$_2$Me), 4.10 (dd, *J* = 9.7, 5.4 Hz, 1H, CH), 3.88 (s, 3H, CO$_2$Me), 2.32 – 2.21 (m, 1H, CH_2 in Et), 2.09 – 1.99 (m, 1H, CH_2 in Et), 1.02 (t, *J* = 7.4 Hz, 3H, CH_3 in Et) ppm. ^{13}C NMR (126 MHz, $CDCl_3$) δ 174.3, 172.2, 153.5, 144.5, 140.7, 129.6, 125.7, 124.3, 114.1, 112.0, 66.5, 53.3, 51.4, 22.7, 11.5 ppm. HRMS (ESI) *m/z* calcd for $C_{19}H_{22}ClN_4O_5$ $[M+H]^+$ 421.1273; found: 421.1273.

(*R*)-2-[(4-Chlorophenyl)(2-hydroxyamino-2-oxoethyl)amino]-*N*-hydroxybutanamide (28)

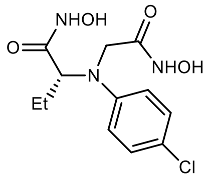

28

56 mg, 93% yield. White solid, mp 86–88 °C. 90% ee [based on the ee of **3c**]. ^1H NMR (500 MHz, CD_3OD) δ 7.18 (d, *J* = 8.8 Hz, 2H, Ar), 6.70 (d, *J* = 8.8 Hz, 2H, Ar), 4.21 – 4.07 (comp, 3H, C$\underline{H}$Et, C$\underline{H}_2$CONH), 2.12 – 2.02 (m, 1H, CH_2 in Et), 1.98 – 1.91 (m, 1H, CH_2 in Et), 0.95 (t, *J* = 7.1 Hz, 3H, CH_3 in Et) ppm. ^{13}C NMR (126 MHz, CD_3OD) δ 170.2, 169.2, 146.1, 129.0, 128.6, 114.5, 63.9, 48.3, 22.8, 10.1 ppm. HRMS (ESI) *m/z* calcd for $C_{12}H_{17}ClN_3O_4$ $[M+H]^+$ 324.0722; found: 324.0731.

(*R*)-2-[(4-Chlorophenyl)(2-hydrazineyl-2-oxoethyl)amino]butanehydrazide (29)

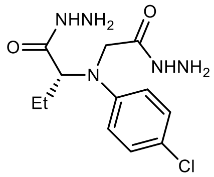

29

56 mg, 94% yield. White solid, mp 202–203 °C. 90% ee [based on the ee of **3c**]. ^1H NMR (500 MHz, $CDCl_3$) δ 9.66 (br, 1H, NH), 8.76 (br, 1H, NH), 7.15 (d, *J* = 9.0 Hz, 2H, Ar), 6.56 (d, *J* = 9.0 Hz, 2H, Ar), 4.12 (d, *J* = 17.4 Hz, 1H, C$\underline{H}_2$CONH), 4.04 (dd, *J* = 9.3, 5.7 Hz, 1H, CH), 3.95 (d, *J* = 17.4 Hz, 1H, C$\underline{H}_2$CONH), 4.10 – 3.75 (br, 4H, NH_2), 2.17 – 2.05 (m, 1H, CH_2 in Et), 1.89 –

1.78 (m, 1H, CH_2 in Et), 0.92 (t, *J* = 7.3 Hz, 3H, CH_3 in Et) ppm. ^{13}C NMR (126 MHz, $CDCl_3$) δ 172.5, 171.5, 145.5, 129.28, 123.8, 114.3, 65.4, 50.1, 22.9, 11.4 ppm. HRMS (ESI) *m/z* calcd for $C_{12}H_{19}ClN_5O_2$ $[M+H]^+$ 300.1222; found: 300.1225.

(*R*)-2-[(2-Amino-2-oxoethyl)(4-chlorophenyl)amino]butanamide (30)

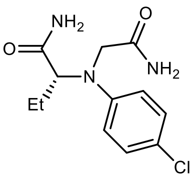

30

52 mg, 96% yield. White solid, mp 290–292 °C. 90% ee [based on the ee of **3c**]. ^1H NMR (300 MHz, CD_3OD) δ 7.18 (d, *J* = 9.1 Hz, 2H, Ar), 6.66 (d, *J* = 9.1 Hz, 2H, Ar), 4.21 (d, *J* = 17.7 Hz, 1H, $C\underline{H}_2CONH_2$), 4.15 – 4.03 (comp, 2H, $C\underline{H}Et$, $C\underline{H}_2CONH_2$), 2.18 – 2.03 (m, 1H, CH_2 in Et), 2.03 – 1.87 (m, 1H, CH_2 in Et), 0.97 (t, *J* = 7.4 Hz, 3H, CH_3 in Et) ppm. ^{13}C NMR (126 MHz, CD_3OD) δ 176.9, 175.2, 146.2, 128.5, 122.6, 114.0, 65.9, 50.8, 22.6, 10.3 ppm. HRMS (ESI) *m/z* calcd for $C_{12}H_{17}ClN_3O_2$ $[M+H]^+$ 270.1004; found: 270.1004.

(*R*)-*N*-(1-[(Amino(iminio)methyl)amino]-1-oxobutan-2-yl)-*N*-(4-chlorophenyl)glycinate (31)

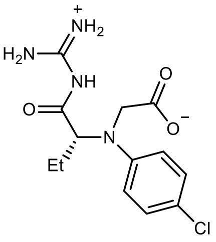

31

49 mg, 78% yield. White solid, mp 211–212 °C. 90% ee [based on the ee of **3c**]. ^1H NMR (300 MHz, DMSO-*d*$_6$) δ 7.15 (d, *J* = 8.7 Hz, 2H, Ar), 6.50 (d, *J* = 8.7 Hz, 2H, Ar), 4.04 – 3.69 (comp, 2H, $C\underline{H}Et$, $C\underline{H}_2COO$), 3.37 (br, 4H, $C\underline{H}_2COO$, NH_2), 2.05 – 1.82 (comp, 2H, CH_2 in Et), 0.86 (t, *J* = 7.2 Hz, 3H, CH_3 in Et) ppm. ^{13}C NMR (75 MHz, DMSO-*d*$_6$) δ 175.5, 174.9, 129.1, 128.9, 120.7, 113.7, 49.0, 23.0, 11.9, 11.3 ppm. HRMS (ESI) *m/z* calcd for $C_{13}H_{18}ClN_4O_3$ $[M+H]^+$ 313.1062; found: 313.1069.

Procedure for preparative deuterium incorporation

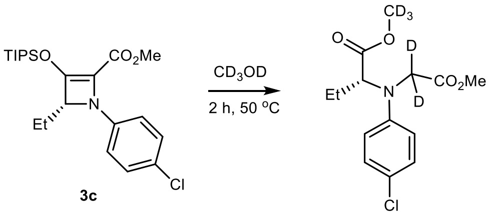

To an 8-mL screw-capped vial equipped with a magnetic stirring bar azetine **3c** (84.6 mg, 0.200 mmol) and 4.00 mL of methanol-*d*$_4$ were sequentially introduced at room temperature. The vial was sealed, and the reaction solution was stirred at 65 °C for 2 h. Methanol-*d*$_4$ was then evaporated, and the residue was purified by flash chromatography on silica gel using a gradient hexane/ethyl acetate 9:1 to 4:1 (v/v) to afford **methyl-*d*$_3$ (*R*)-2-[(4-chlorophenyl)(2-methoxy-2-oxoethyl-1,1-*d*$_2$)amino]butanoate** (**32**) as a colorless oil (56.0 mg, 92% yield), 90% ee [based on the ee of **3c**]. ^1H NMR (500 MHz, $CDCl_3$) δ 7.19 (d, *J* = 9.0 Hz, 2H, Ar), 6.69 (d, *J* = 19.0 Hz, 2H, Ar), 4.26 (t, *J* = 7.5 Hz, 1H, CH), 3.76 (s, 3H, CO_2Me), 2.02 – 1.91 (m, 1H, CH_2 in Et), 1.90 – 1.81 (m, 1H, CH_2 in Et), 1.03 (t, *J* = 7.4 Hz, 3H, CH_3 in Et) ppm. ^{13}C NMR (126 MHz, $CDCl_3$) δ 172.8, 171.6, 147.3, 129.1, 123.6, 114.8, 62.6, 52.1, 23.8, 10.9 ppm. ^2H NMR (77 MHz, $CHCl_3/CDCl_3$ 4:1) δ 4.13 (br, 1H, $C\underline{D}_2CO_2Me$), 3.98 (br, 1H, $C\underline{D}_2CO_2Me$), 3.63 (br, 3H, CD_3) ppm. HRMS (ESI) *m/z* calcd for $C_{14}H_{14}D_5ClNO_4$ $[M+H]^+$ 305.1311; found: 305.1319.

A ^{1}H NMR experiment with CD_3OD at 50 °C showed clean conversion of **3c** to **32** over time (Fig. S1). The reaction reached near complete conversion in 60 min without detection of intermediates **I**–**L** by the NMR method. Clean conversion of **3c** to **32** was easily confirmed by following the change of absorptions of the aromatic protons from 7.21 and 6.96 ppm (**3c**) to 7.16 and 6.76 ppm (**32**), or the (Et)C–**H** proton from 3.96 ppm (**3c**) to 4.35 ppm (**32**) (Fig. S1).

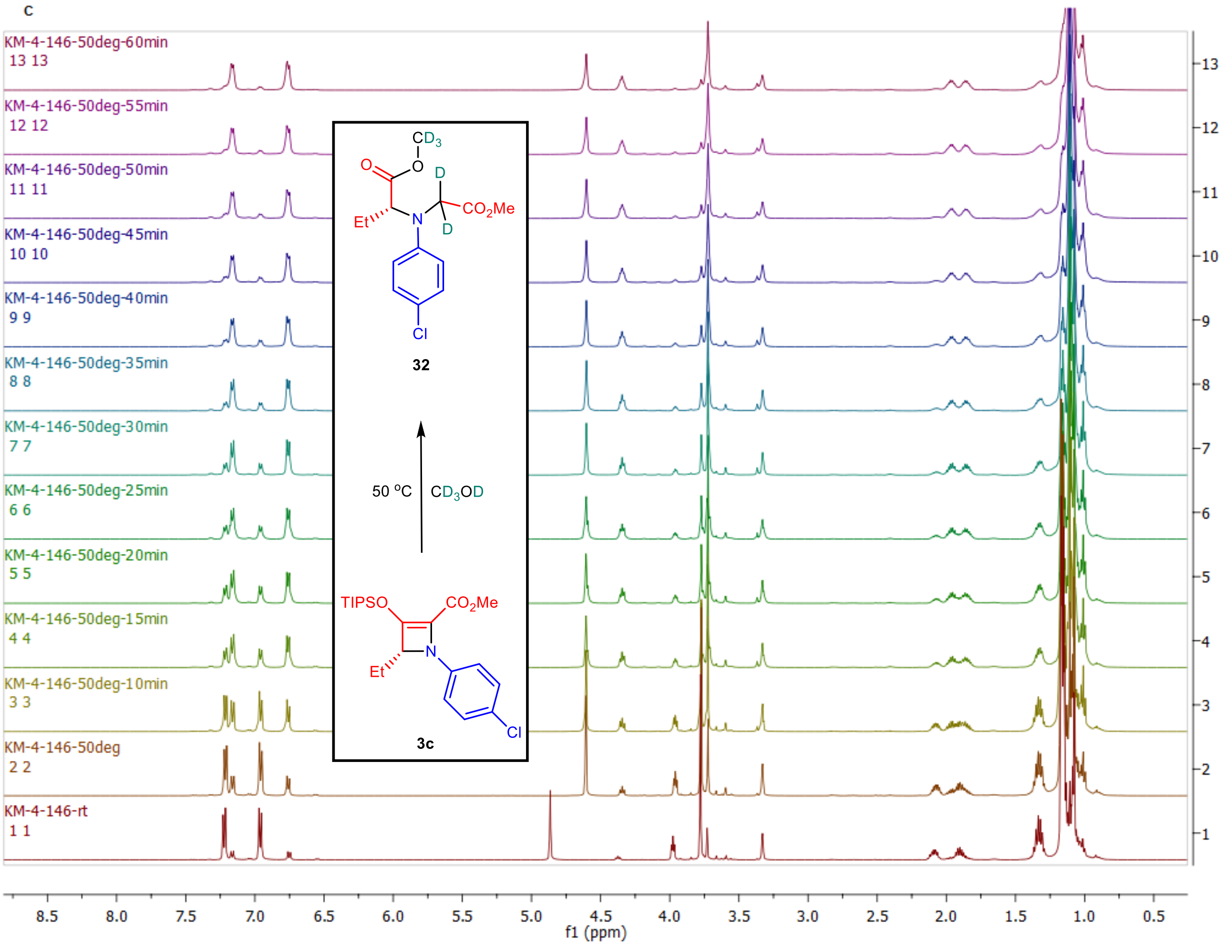

Supplementary Figure 1. ^{1}H NMR kinetics for the ring opening of 3c by CD_3OD used as a solvent. NMR scans were performed every 5 minutes at 50 °C (first scan was done at 23 °C)

Procedure for proton–deuterium exchange with CD_3OD

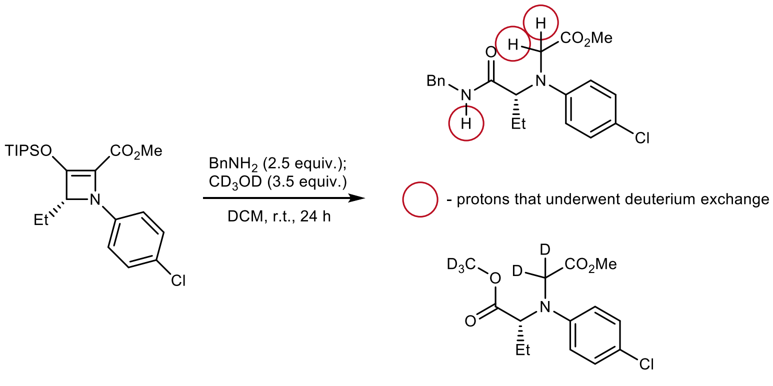

To a 8-mL screw-capped vial equipped with a magnetic stirring bar azetine **3c** (42 mg, 0.10 mmol) was introduced, followed by the addition of a mixture of benzylamine (27 mg, 0.25 mmol) and MeOD (13 mg, 0.35 mmol) in 2.0 mL of DCM. The vial was sealed, and the reaction solution was stirred at room temperature for 24 h. All volatiles were then evaporated in vacuum, and the resulting reaction mixture was subjected to the ^{1}H NMR analysis. Deuterium exchange partially occurred, as evidenced by integration of the circled positions: ~17% at the amine nitrogen; ~26% at one methylene position and ~40% at the other methylene position (~33% average) alpha to the CO_2Me group. One interpretation of deuterium capture from MeOD is that deuterium exchange occurred with the ammonium ion after initial nucleophilic addition to the carbonyl group of the β-ketoester so that a fraction of the deuterium exchange was directed from the ammonium group; but even without this interpretation, the majority of the reaction involved proton transfer from the ammonium group.

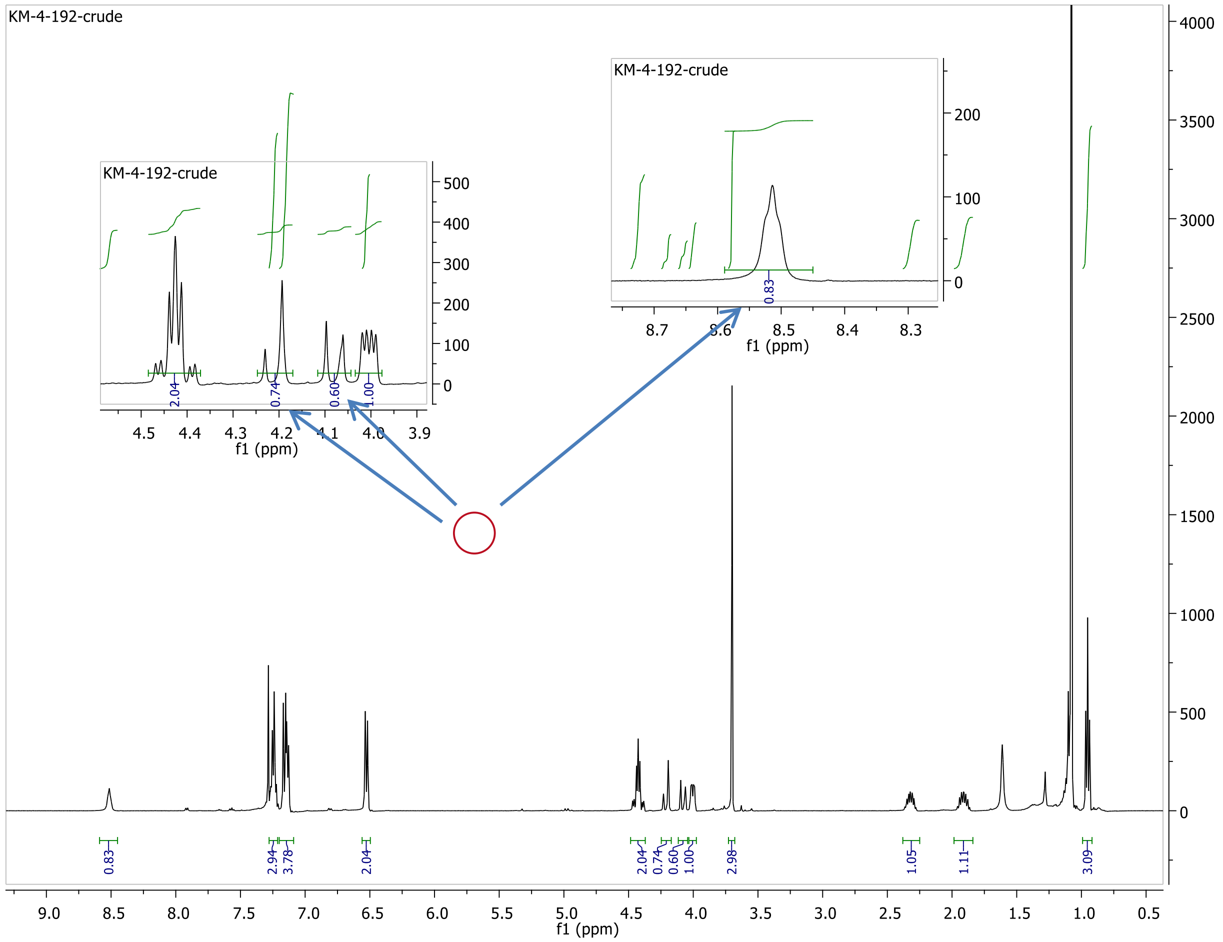

Supplementary Figure 2. ^{1}H NMR spectrum of the reaction mixture for proton−deuterium exchange experiment

Procedure for Suzuki-Miyaura cross-coupling of a ring opened product

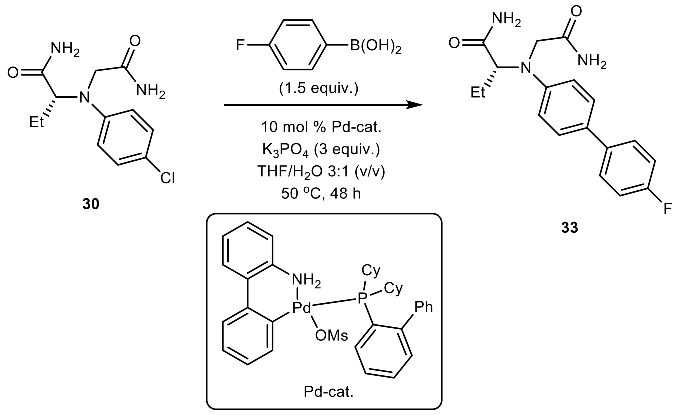

Synthesis of Pd-catalyst. Palladium precatalyst was prepared by mixing (2'-amino-1,1'-biphenyl-2-yl)methanesulfonatopalladium(II) dimer (250 mg, 0.340 mmol) and 2-(dicyclohexylphosphino)-1,1'-biphenyl (CyJohnPhos, 262 mg, 0.748 mmol) in 15.0 mL of dry THF under the atmosphere of dinitrogen. The reaction solution was stirred at room temperature for 2 h. THF was then evaporated, the residue was washed with hexane (3 x ca. 10 mL), and dried under vacuum to afford palladium precatalyst (near quantitative yield), which was used in the coupling reaction without further purification.

Suzuki coupling. An 8-mL screw-capped vial equipped with a magnetic stirring bar was charged with diamide **30** (40.4 mg, 0.150 mmol), 4-fluorophenylboronic acid (31.5 mg, 0.225 mmol), palladium catalyst (10.8 mg, 0.0150 mmol), anhydrous potassium phosphate (95.3 mg, 0.450 mmol), THF (3.00 mL), and water (1.00 mL). The system was flushed with nitrogen, sealed, and warmed to 50 °C. Stirring was continued at 50 °C for 48 h (conversion of **30** monitored by LC/MS). Subsequently, the reaction mixture was extracted with ethyl acetate (3 x ca. 3 mL). The organic layer was separated, dried over anhydrous sodium sulfate (ca. 300 mg), and solvents were evaporated under reduced pressure. The residue was purified by flash chromatography on silica gel using a gradient of DCM/methanol 19:1 to 9:1 (v/v) to afford **(*R*)-2-[(2-amino-2-oxoethyl)(4'-fluoro-[1,1'-biphenyl]-4-yl)amino]butanamide** (**33**) as a white solid (mp >300 °C, 39.0 mg, 79% yield), 90% ee [based on the ee of **3c**]. ^1H NMR (500 MHz, CD_3OD) δ 7.54 (dd, *J* = 8.7, 5.4 Hz, 2H, Ar), 7.46 (d, *J* = 8.7 Hz, 2H, Ar), 7.11 (t, *J* = 8.7 Hz, 2H, Ar), 6.78 (d, *J* = 8.7 Hz, 2H, Ar), 4.26 (d, *J* = 17.6 Hz, 1H, C<u>H</u>$_2$CONH$_2$), 4.13 – 4.06 (comp, 2H, C<u>H</u>Et, C<u>H</u>$_2$CONH$_2$), 2.15 – 2.03 (m, 1H, CH_2 in Et), 2.00 – 1.90 (m, 1H, CH_2 in Et), 1.00 (t, *J* = 7.4 Hz, 3H, CH_3 in Et) ppm. ^{13}C NMR (126 MHz, CD_3OD) δ 177.2, 175.5, 162.8, 146.7, 137.1, 128.7, 127.45 (d, *J* = 7.8 Hz), 127.1, 115.0, 113.1, 65.7, 50.6, 22.6, 10.4 ppm. ^{19}F NMR (471 MHz, CD_3OD) δ -119.6 (s) ppm. HRMS (ESI) *m/z* calcd for $C_{18}H_{21}FN_3O_2$ $[M+H]^+$ 330.1612; found: 330.1622.

Procedure for the ring opening of azetine 3c with 9-aminoacridine

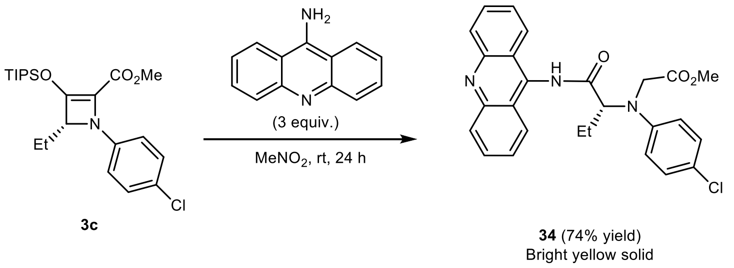

To an 8-mL screw-capped vial equipped with a magnetic stirring bar azetine **3c** (84.6 mg, 0.200 mmol), 9-aminoacridine (116 mg, 0.600 mmol), and nitromethane (4.00 mL) were sequentially introduced. The vial was capped, and the reaction suspension was stirred at room temperature for 24 h. Nitromethane was then evaporated under reduced pressure, and the residue was purified by flash chromatography on silica gel using pure ethyl acetate as eluent to afford methyl **(*R*)-*N*-[1-(acridin-9-ylamino)-1-oxobutan-2-yl]-*N*-(4-chlorophenyl)glycinate** (**34**) as a bright yellow solid (mp 174–176 °C, 68.2 mg, 74% yield), 90% ee [based on the ee of **3c**]. ^{1}H NMR (500 MHz, $CDCl_3$) δ 11.05 (s, 1H, NH), 8.76 (d, *J* = 8.6 Hz, 2H, Ar), 8.68 (d, *J* = 8.6 Hz, 2H, Ar), 8.22 (t, *J* = 7.6 Hz, 2H, Ar), 7.89 (t, *J* = 7.6 Hz, 2H, Ar), 7.19 (d, *J* = 8.9 Hz, 2H, Ar), 6.72 (d, *J* = 8.9 Hz, 2H, Ar), 4.36 (d, *J* = 18.2 Hz, 1H, C<u>H</u>$_2$CO$_2$Me), 4.19 (d, *J* = 18.2 Hz, 1H, C<u>H</u>$_2$CO$_2$Me), 4.09 (dd, *J* = 10.0, 4.7 Hz, 1H, CH), 3.92 (s, 3H, CO_2Me), 2.52 – 2.47 (m, 1H, CH_2 in Et), 2.11 – 1.97 (m, 1H, CH_2 in Et), 0.99 (t, *J* = 7.3 Hz, 3H, CH_3 in Et) ppm. ^{13}C NMR (126 MHz, $CDCl_3$) δ 174.4, 171.3, 167.9, 149.1, 144.8, 139.7, 136.1, 134.8, 128.3, 127.8, 123.5, 123.4, 118.9, 112.5, 108.1, 72.9, 56.0, 53.5, 25.4, 12.9 ppm. HRMS (ESI) *m/z* calcd for $C_{26}H_{25}ClN_3O_3$ $[M+H]^+$ 462.1584; found: 462.1587.

Fluorescence analysis for 34

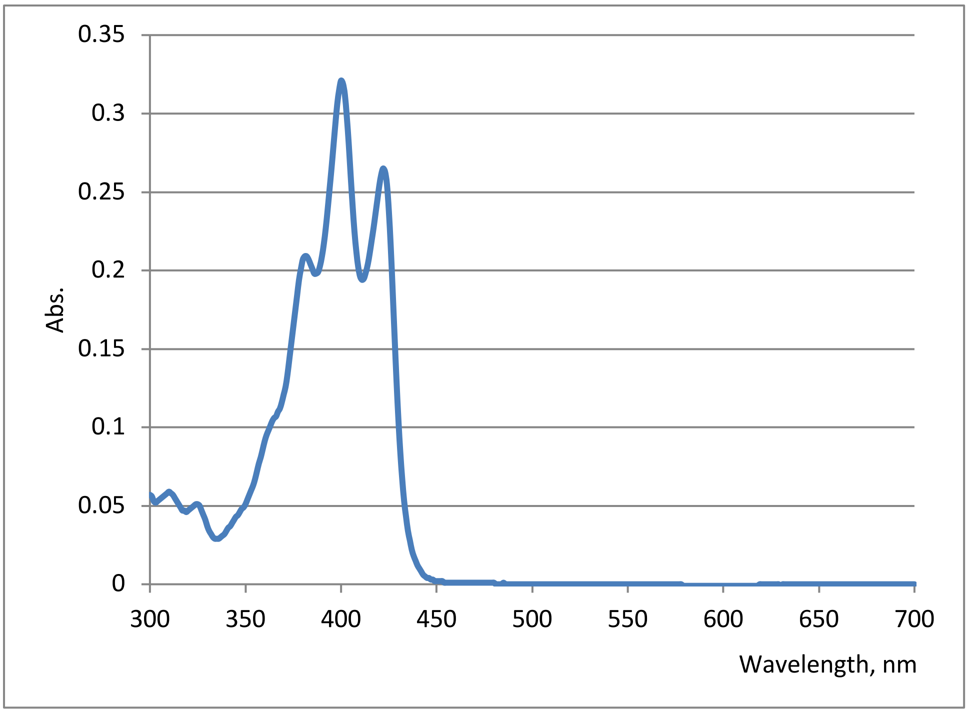

Supplementary Figure 3. UV-Visible spectrum of 34: λ_{max} = 380, 399, and 421 nm

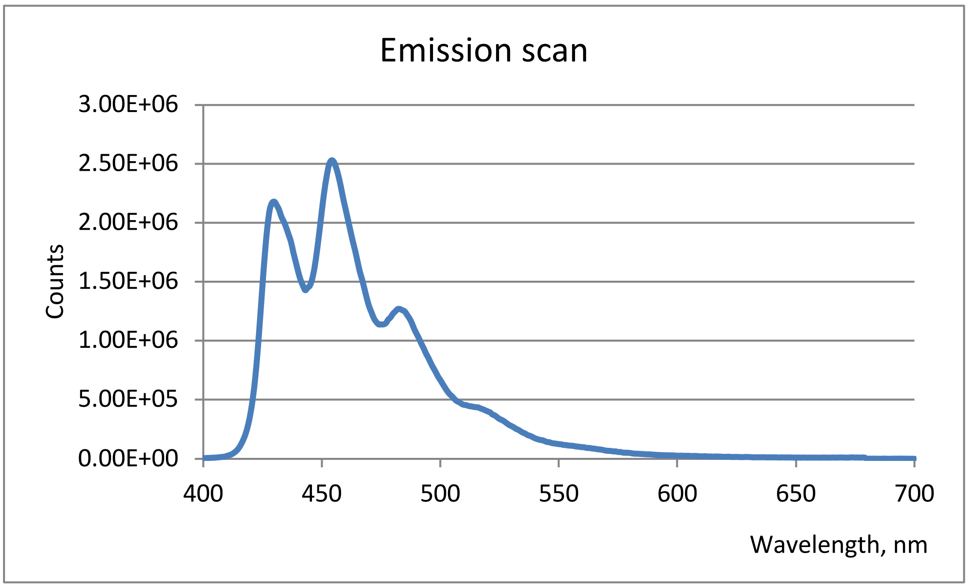

Supplementary Figure 4. Fluorescence spectrum of 34: λ_{max} = 428, 453, and 480 nm

Procedure for esterification of cholesterol and ergocalciferol (vitamin D$_2$)

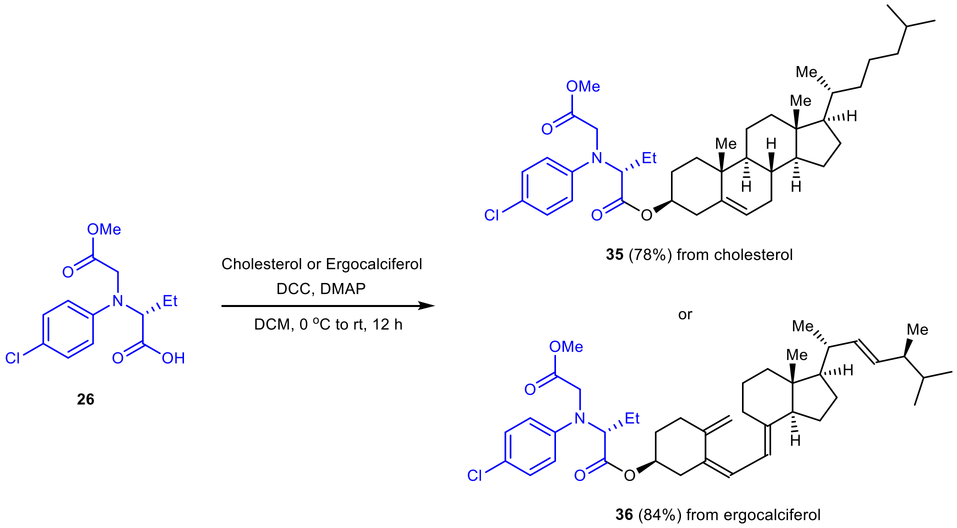

To an 10-mL oven-dried round-bottom flask equipped with a magnetic stirring bar and a rubber septum compound **26** (57 mg, 0.20 mmol), 4-dimethylaminopyridine (DMAP, 3.7 mg, 0.03 mmol), and cholesterol or ergocalciferol (0.22 mmol) were sequentially introduced, and the system was filled with nitrogen. Dry DCM (1.5 mL) was added via syringe, and the resulting solution was cooled to 0 °C. A solution of *N*,*N*'-dicyclohexylcarbodiimide (DCC, 50 mg, 0.24 mmol) in dry DCM (2.0 mL) was then added dropwise within 1 min at 0 °C. The reaction solution was then warmed to room temperature (within 30 min), and stirring was continued for 12 h. Subsequently, the reaction mixture was concentrated under reduced pressure, and the residue was purified by flash chromatography on silica gel using a mixture of hexane/ethyl acetate 19:1 (v/v) as eluent to afford esterification products **35** and **36**.

(3*S*,8*S*,9*S*,10*R*,13*R*,14*S*,17*R*)-10,13-Dimethyl-17-[(*R*)-6-methylheptan-2-yl]-2,3,4,7,8,9,10,11,12,13,14,15,16,17-tetradecahydro-1*H*-cyclopenta[a]phenanthren-3-yl (*R*)-2-[(4-chlorophenyl)(2-methoxy-2-oxoethyl)amino]butanoate (35)

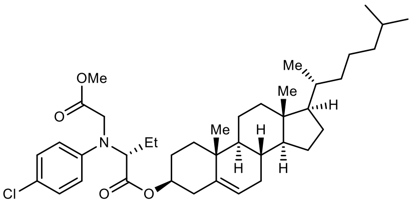

35

102 mg, 78% yield. White solid, mp 103–104 °C. Single enantiomer (>99% ee). ^{1}H NMR (500 MHz, $CDCl_3$) δ 7.19 (d, *J* = 9.1 Hz, 2H, Ar), 6.70 (d, *J* = 9.1 Hz, 2H, Ar), 5.38 (d, *J* = 4.6 Hz, 1H, CH=), 4.70 – 4.60 (m, 1H, CHO), 4.27 (d, *J* = 18.4 Hz, 1H, $C\underline{H}_2CO_2Me$), 4.19 (t, *J* = 7.5 Hz, 1H, $C\underline{H}Et$), 4.05 (d, *J* = 18.4 Hz, 1H, $C\underline{H}_2CO_2Me$), 3.76 (s, 3H, CO_2Me), 2.31 (comp, 2H, alkyl), 2.08 – 1.91 (comp, 4H, alkyl), 1.90 – 1.68 (comp, 6H, alkyl), 1.65 – 1.43 (comp, 6H, alkyl), 1.42 – 1.22 (comp, 6H, alkyl), 1.21 – 1.06 (comp, 6H, alkyl), 1.04 (t, *J* = 7.4 Hz, 3H, CH_3 in Et), 1.03 (s, 3H, $C\underline{H}_3C$), 0.93 (d, *J* = 6.5 Hz, 3H, $C\underline{H}_3CH$), 0.89 (d, *J* = 2.2 Hz, 3H, CH_3 in *i*Pr), 0.88 (d, *J* = 2.2 Hz, 3H, CH_3 in *i*Pr), 0.70 (s, 3H, $C\underline{H}_3C$) ppm. ^{13}C NMR (126 MHz, $CDCl_3$) δ 171.91, 171.77, 147.54, 139.26, 129.00, 123.49, 122.95, 114.94, 74.83, 63.08, 56.67, 56.13, 52.12, 49.99, 49.32, 42.31, 39.71, 39.52, 38.04, 36.90, 36.56, 36.18, 35.79, 31.88, 31.83, 28.22, 28.02, 27.82, 24.27, 24.01, 23.83, 22.82, 22.56, 21.03, 19.32, 18.72, 11.86, 11.00 ppm. HRMS (ESI) *m/z* calcd for $C_{40}H_{61}ClNO_4$ $[M+H]^+$ 654.4284; found: 654.4290.

(*S*,*Z*)-3-(2-((1*R*,3a*S*,7a*R*,*E*)-1-((2*R*,5*S*,*E*)-5,6-Dimethylhept-3-en-2-yl)-7a-methyloctahydro-4*H*-inden-4-ylidene)ethylidene)-4-methylenecyclohexyl (*R*)-2-((4-chlorophenyl)(2-methoxy-2-oxoethyl)amino)butanoate (36)

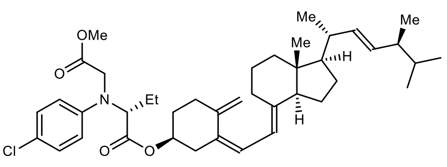

37

112 mg, 84% yield. White solid, mp 95–96 °C. Single enantiomer (>99% ee). ^{1}H NMR (500 MHz, $CDCl_3$) δ 7.11 (d, *J* = 9.0 Hz, 2H, Ar), 6.64 (d, *J* = 9.0 Hz, 2H, Ar), 6.14 (d, *J* = 11.2 Hz, 1H, CH=), 6.01 (d, *J* = 11.2 Hz, 1H, CH=), 5.25 – 5.14 (comp, 2H, CH=), 5.06 – 4.99 (comp, 2H, CHO, $=CH_2$), 4.83 (d, *J* = 1.9 Hz, 1H, $=CH_2$), 4.22 (d, *J* = 18.5 Hz, 1H, $C\underline{H}_2CO_2Me$), 4.15 (t, *J* = 7.5 Hz, 1H, $C\underline{H}Et$), 3.97 (d, *J* = 18.5 Hz, 1H, $C\underline{H}_2CO_2Me$), 3.73 (s, 3H, CO_2Me), 2.80 – 2.71 (m, 1H, alkyl), 2.54 – 2.47 (m, 1H, alkyl), 2.42 – 2.36 (m, 1H, alkyl), 2.30 (m, 1H, alkyl), 2.23 – 2.13 (m, 1H, alkyl), 2.08 – 1.80 (comp, 6H, alkyl), 1.80 – 1.61 (comp, 4H, alkyl), 1.54 – 1.43 (comp, 4H, alkyl), 1.38 – 1.27 (comp, 4H, alkyl), 1.01 (comp, 6H, CH_3 in Et, $C\underline{H}_3CH$), 0.92 (d, *J* = 6.8 Hz, 3H, $C\underline{H}_3CH$), 0.83 (dd, *J* = 7.2, 1.0 Hz, 6H, $C\underline{H}_3CH$ in *i*Pr), 0.55 (s, 3H, CH_3C) ppm. ^{13}C NMR (126 MHz, $CDCl_3$) δ 172.09, 171.81, 147.54, 144.43, 142.51, 135.62, 133.85, 131.95, 128.97, 123.48, 122.83, 117.38, 114.98, 112.84, 72.32, 63.08, 56.47, 56.44, 52.11, 49.26, 45.84, 42.82, 41.78, 40.41, 33.11, 31.62, 29.03, 27.80, 24.05, 23.61, 22.27, 21.12, 19.96, 19.65, 17.59, 12.22, 10.99 ppm. HRMS (ESI) *m/z* calcd for $C_{41}H_{59}ClNO_4$ $[M+H]^+$ 664.4127; found: 664.4135.

X-ray crystallographic report for 35

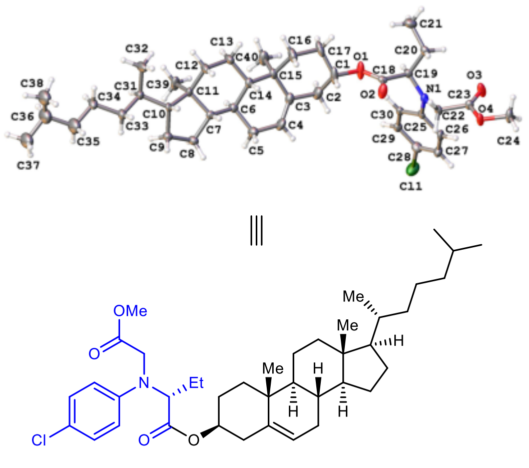

35

Single crystals of **35** ($C_{40}H_{60}ClNO_4$) were prepared by slow evaporation of hexane solution. A suitable colorless plate-like crystal, with dimensions of 0.47 mm × 0.23 mm × 0.07 mm, was mounted in paratone oil onto a nylon loop. All data were collected at 98(2)ºK, using a Rigaku AFC12 / Saturn 724 CCD fitted with MoKα radiation (λ = 0.71075 Å). Data collection and unit cell refinement were performed using *CrysAlisPro* software.[5] The total number of data were measured in the range 4.8° < 2θ < 52.0°, using ω scans. Data processing and absorption correction, giving minimum and maximum transmission factors (0.9682, 1.000) were accomplished with *CrysAlisPro*[5] and *SCALE3 ABSPACK*,[6] respectively. The structure, using Olex2,[7] was solved with the ShelXT[8] structure solution program using direct methods and refined (on F^2) with the ShelXL[9] refinement package using full-matrix, least-squares techniques. All non-hydrogen atoms were refined with anisotropic displacement parameters. All hydrogen atom positions were determined by geometry and refined by a riding model.

Supplementary Table 2. Crystallographic data and structure refinement for cd1852 (35)

Identification code	cd1852
Empirical formula	$C_{40}H_{60}ClNO_4$
Formula weight	654.34
Crystal system	Monoclinic
Space group	*P*2$_1$
a (Å)	15.2622(5)
b (Å)	6.0645(2)
c (Å)	20.4620(8)
α (°)	90
β (°)	90.768(3)
γ (°)	90
Volume (Å^3)	1893.7(1)
Z	2
ρ (calc.)	1.148

λ	0.71075
Temp. (K)	98(2)
F(000)	712
μ (mm^{-1})	0.140
T_{min}, T_{max}	0.9682, 1.000
$2\theta_{range}$ (°)	4.8 to 52.0
Reflections collected	42683
Independent reflections	7453 [R(int) = 0.0429]
Completeness	99.9%
Data / restraints / parameters	7453/ 1 / 404
Observed data [I > 2σ(I)]	7198
wR(F^2 all data)	0.1345
R(F obsd data)	0.0661
Goodness-of-fit on F^2	1.08
largest diff. peak and hole (e $Å^{-3}$)	1.00 / -0.78

$$wR_2 = \{ \Sigma [w(F_o^2 - F_c^2)^2] / \Sigma [w(F_o^2)^2] \}^{1/2}$$

$$R_1 = \Sigma \, ||F_o| - |F_c|| / \Sigma |F_o|$$

Supplementary Figures

NMR spectra of new compounds

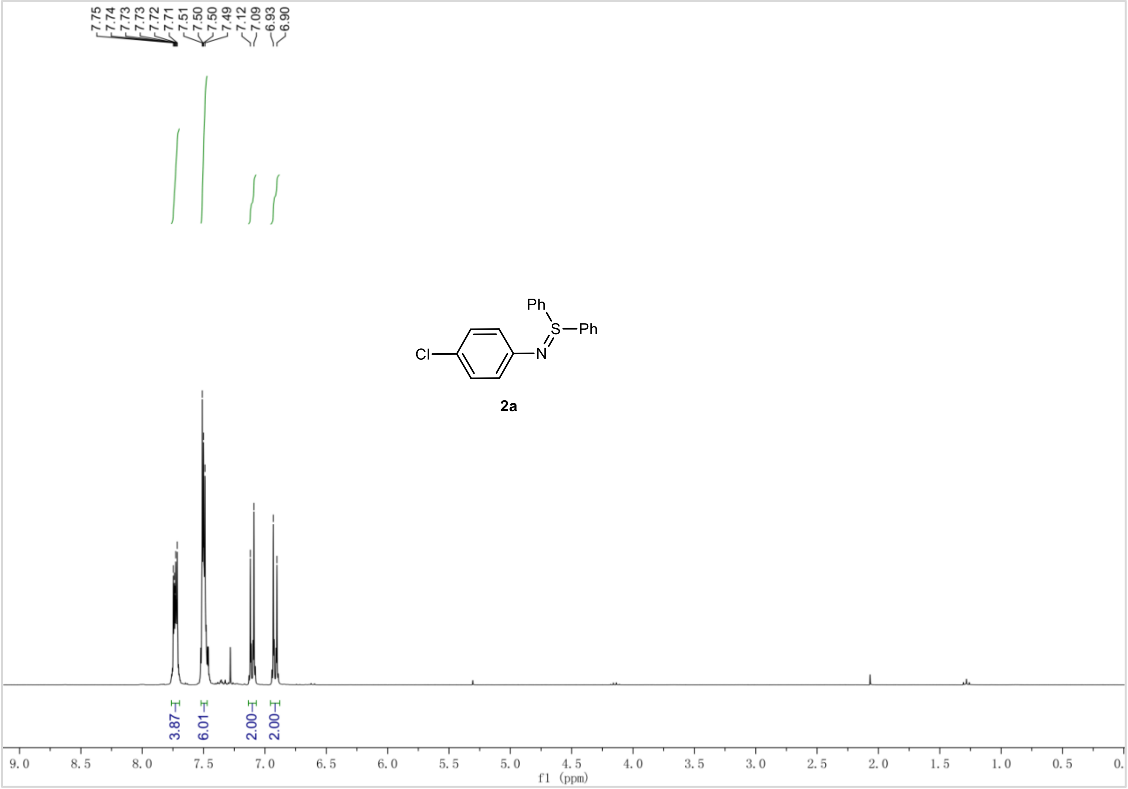

Supplementary Figure 5. ^{1}H NMR (300 MHz, $CDCl_3$) spectrum for 2a

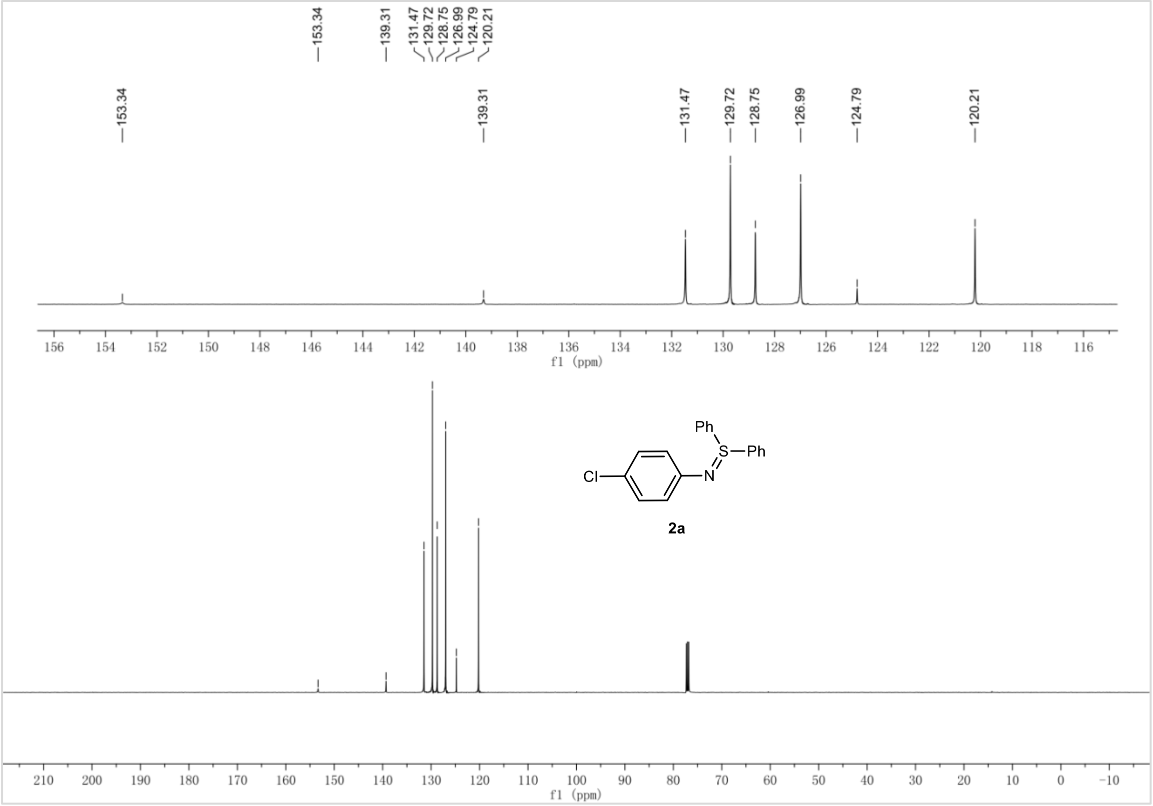

Supplementary Figure 6. ^{13}C NMR (126 MHz, $CDCl_3$) spectrum for 2a

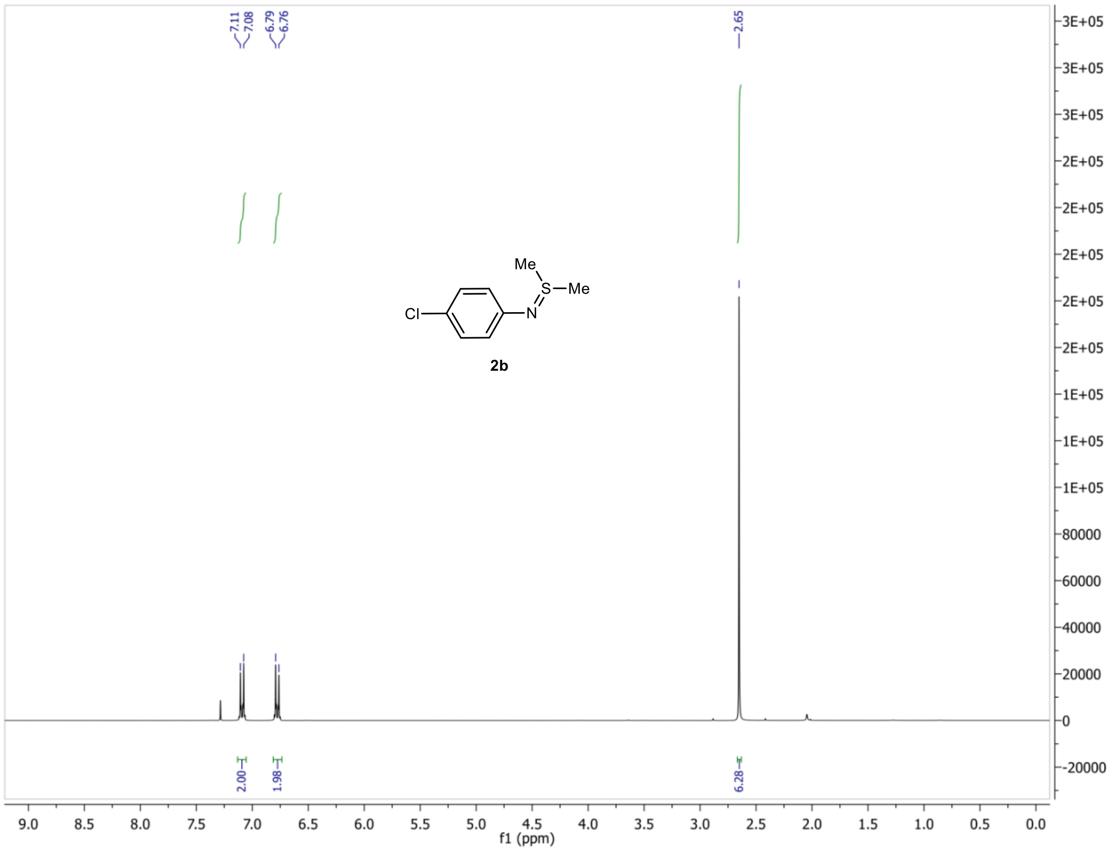

Supplementary Figure 7. ^{1}H NMR (300 MHz, $CDCl_3$) spectrum for 2b

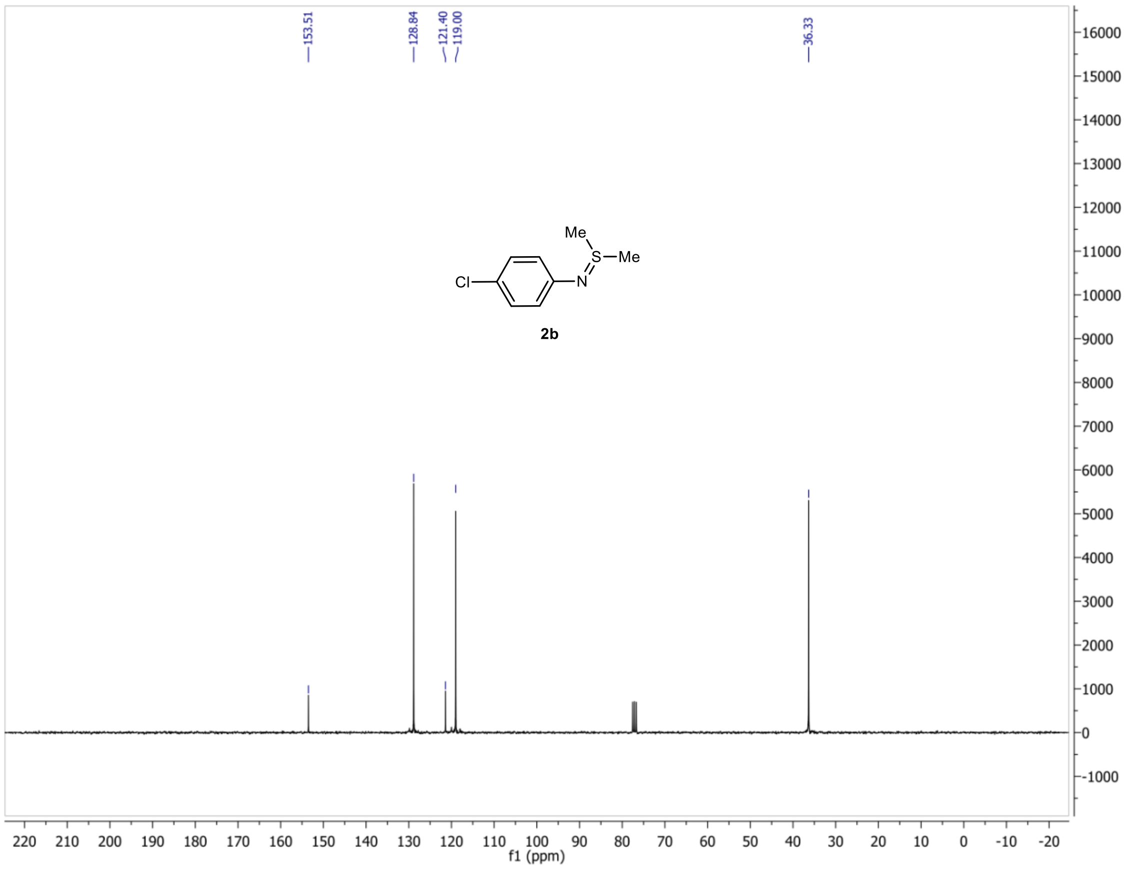

Supplementary Figure 8. ^{13}C NMR (75 MHz, $CDCl_3$) spectrum for 2b

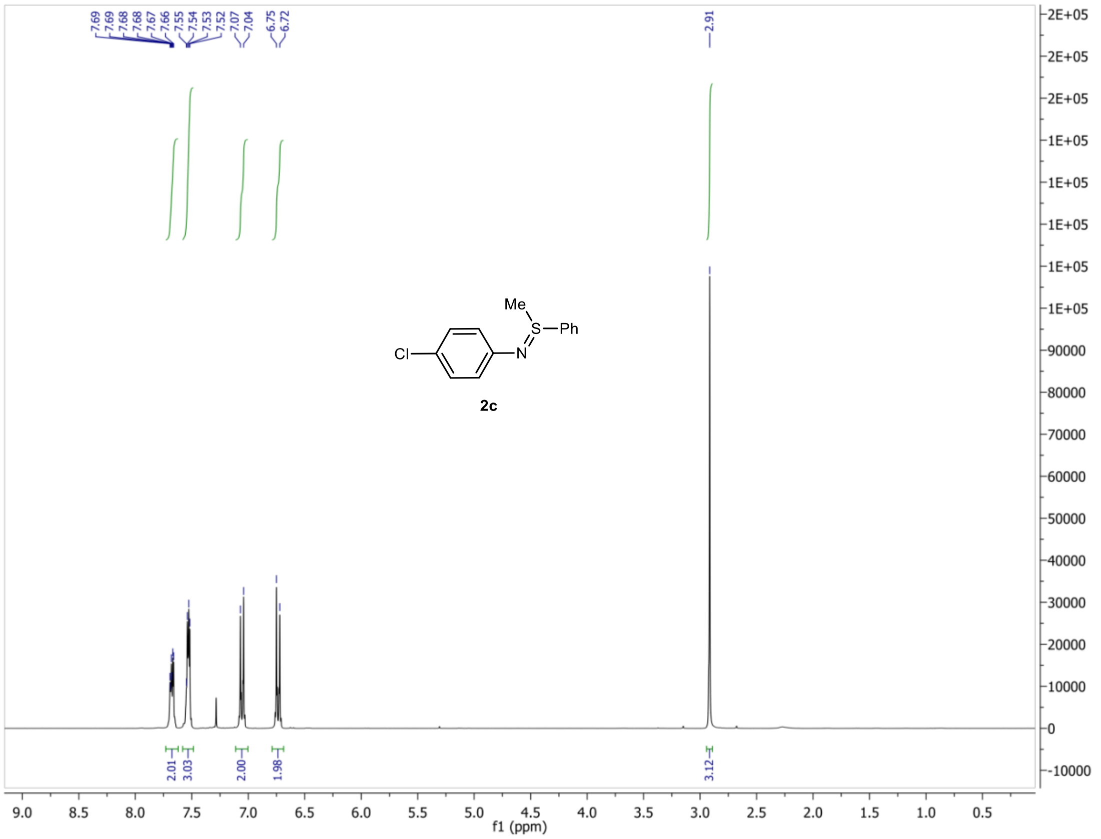

Supplementary Figure 9. ^{1}H NMR (300 MHz, $CDCl_3$) spectrum for 2c

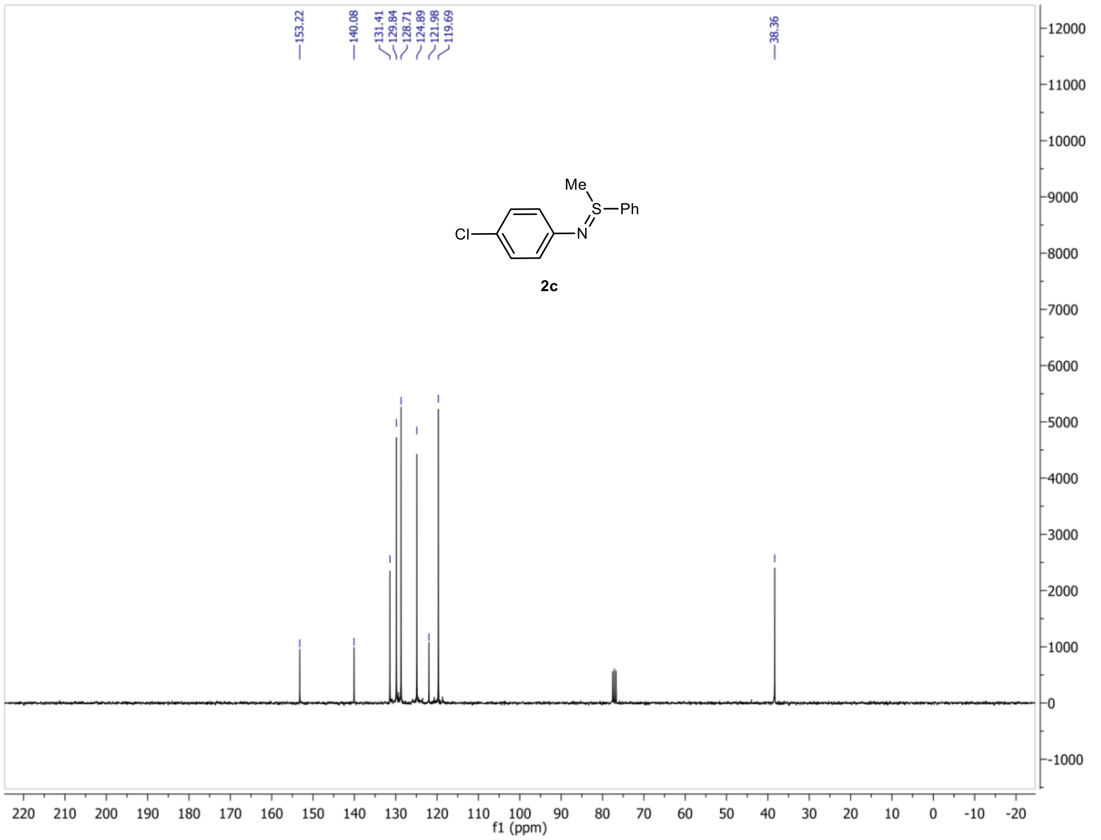

Supplementary Figure 10. ^{13}C NMR (75 MHz, $CDCl_3$) spectrum for 2c

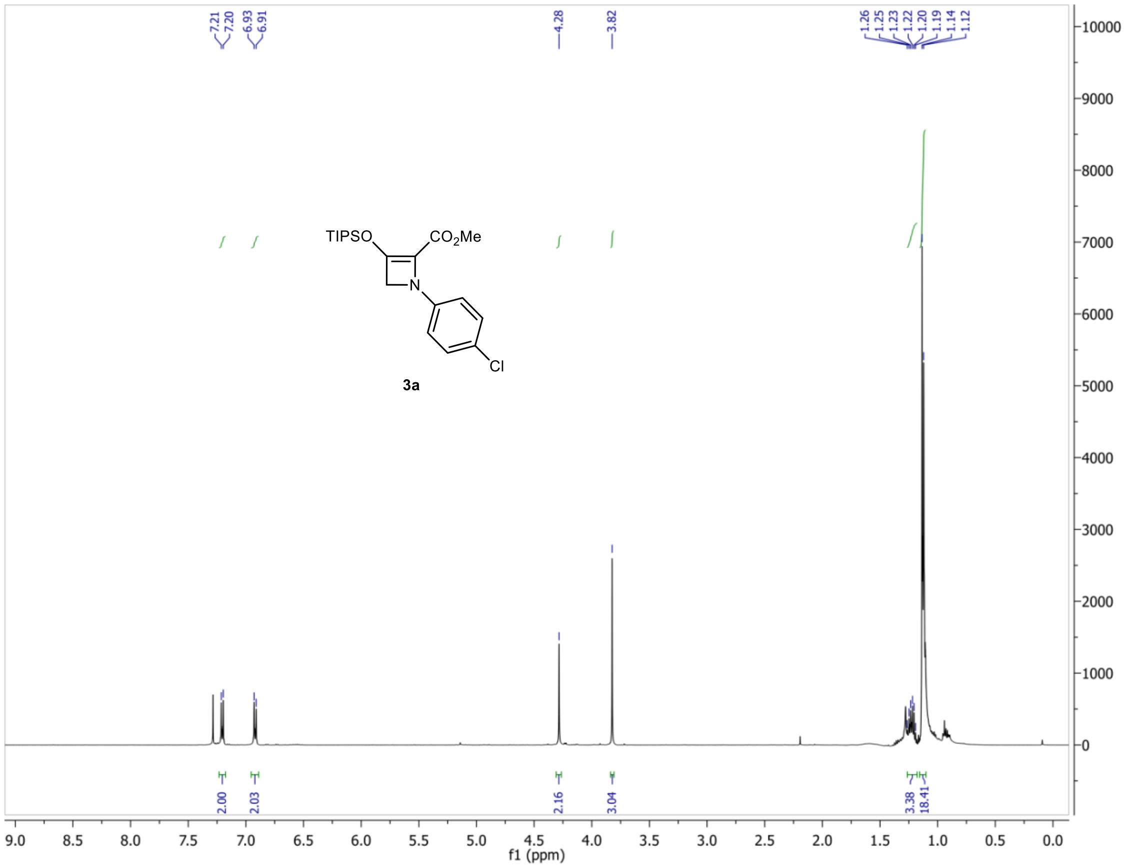

Supplementary Figure 11. ^{1}H NMR (500 MHz, $CDCl_3$) spectrum for 3a

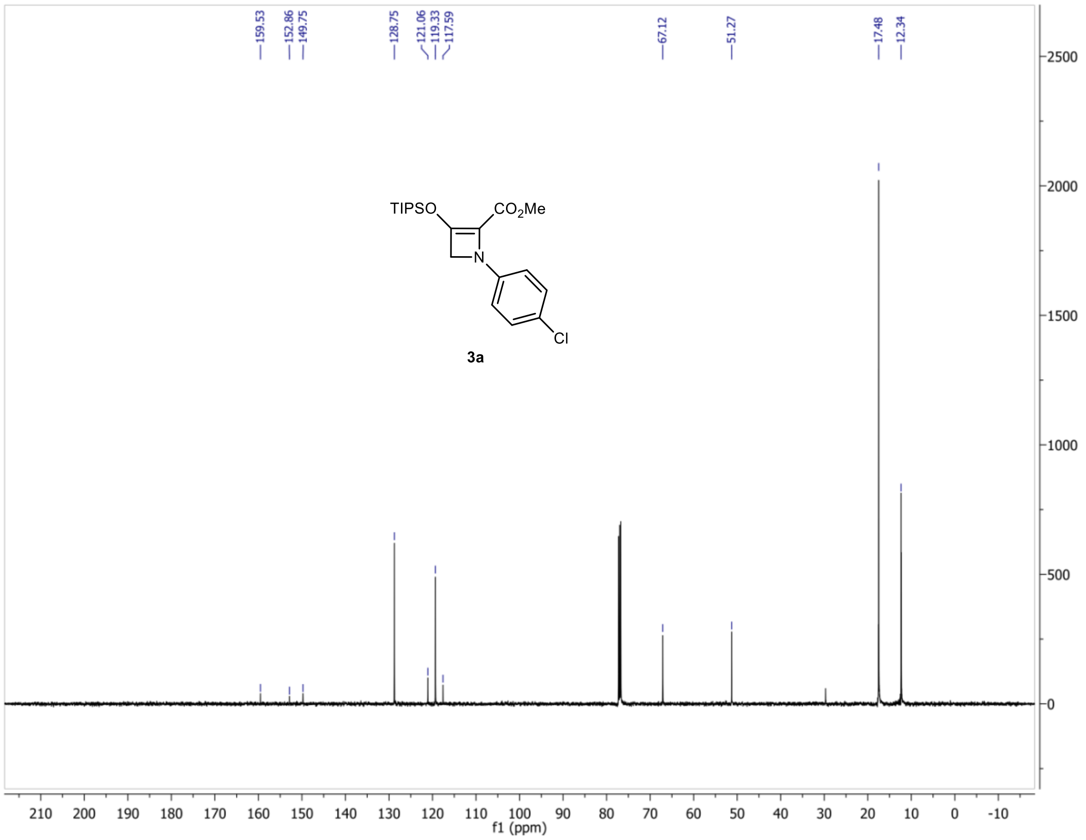

Supplementary Figure 12. ^{13}C NMR (126 MHz, $CDCl_3$) spectrum for 3a

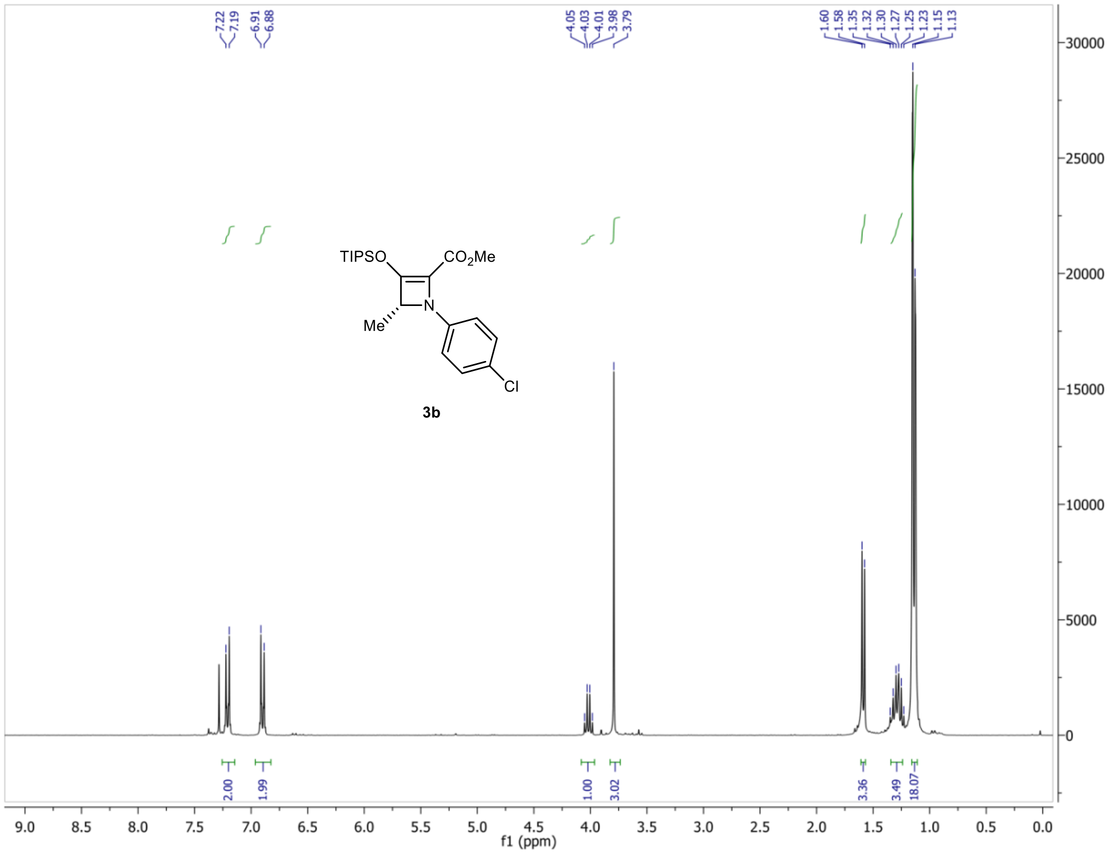

Supplementary Figure 13. ^{1}H NMR (300 MHz, $CDCl_3$) spectrum for 3b

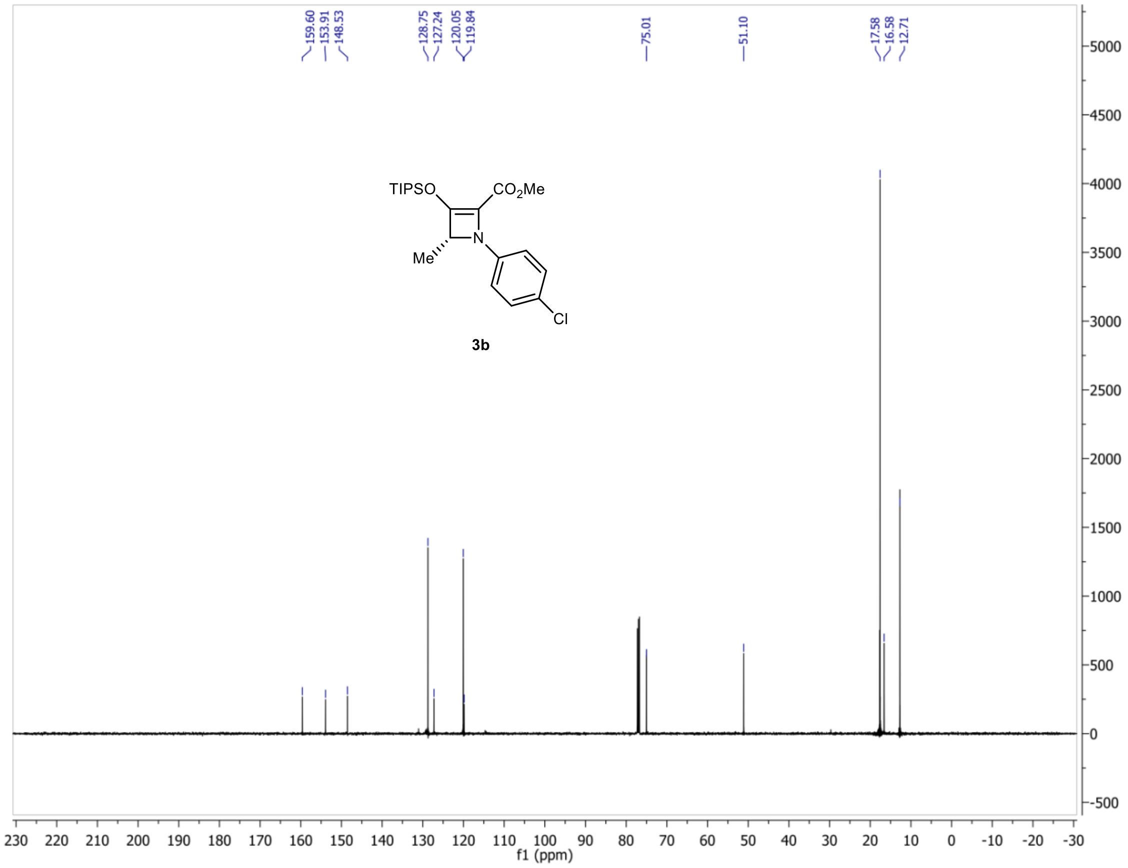

Supplementary Figure 14. ^{13}C NMR (126 MHz, $CDCl_3$) spectrum for 3b

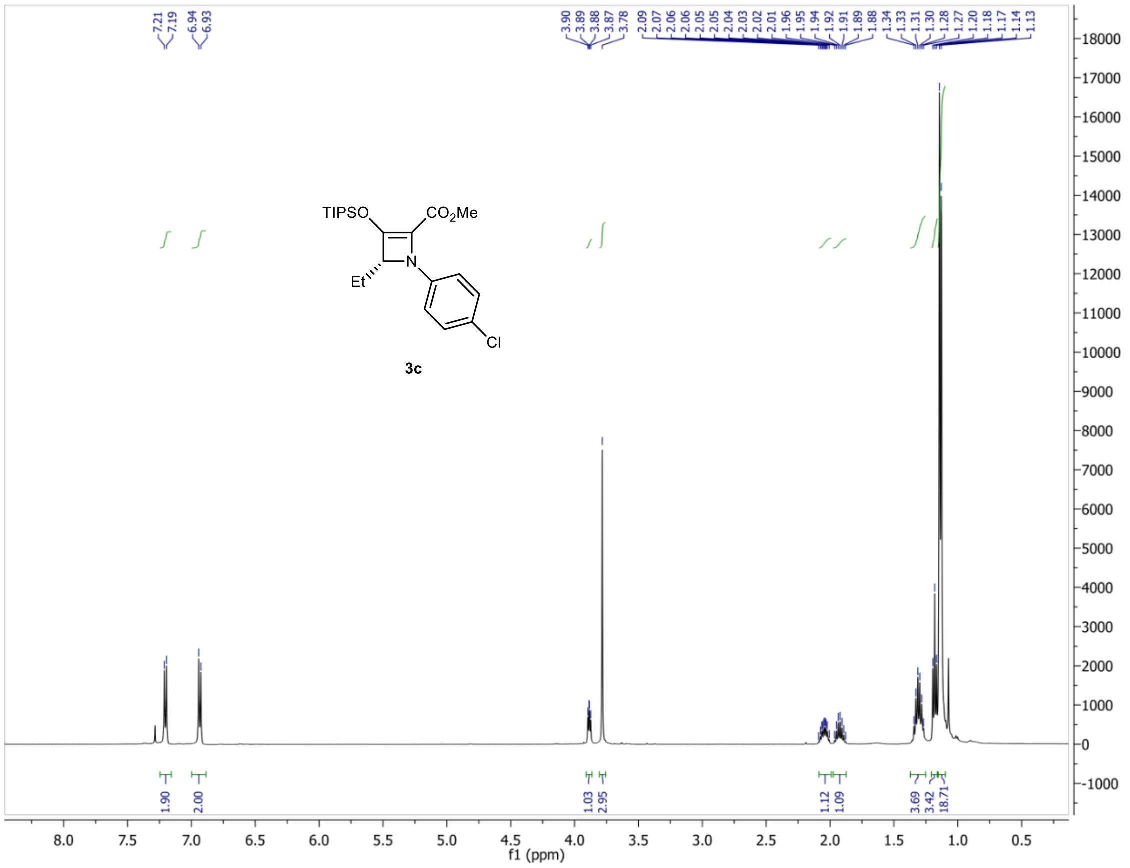

Supplementary Figure 15. ^{1}H NMR (500 MHz, $CDCl_3$) spectrum for 3c

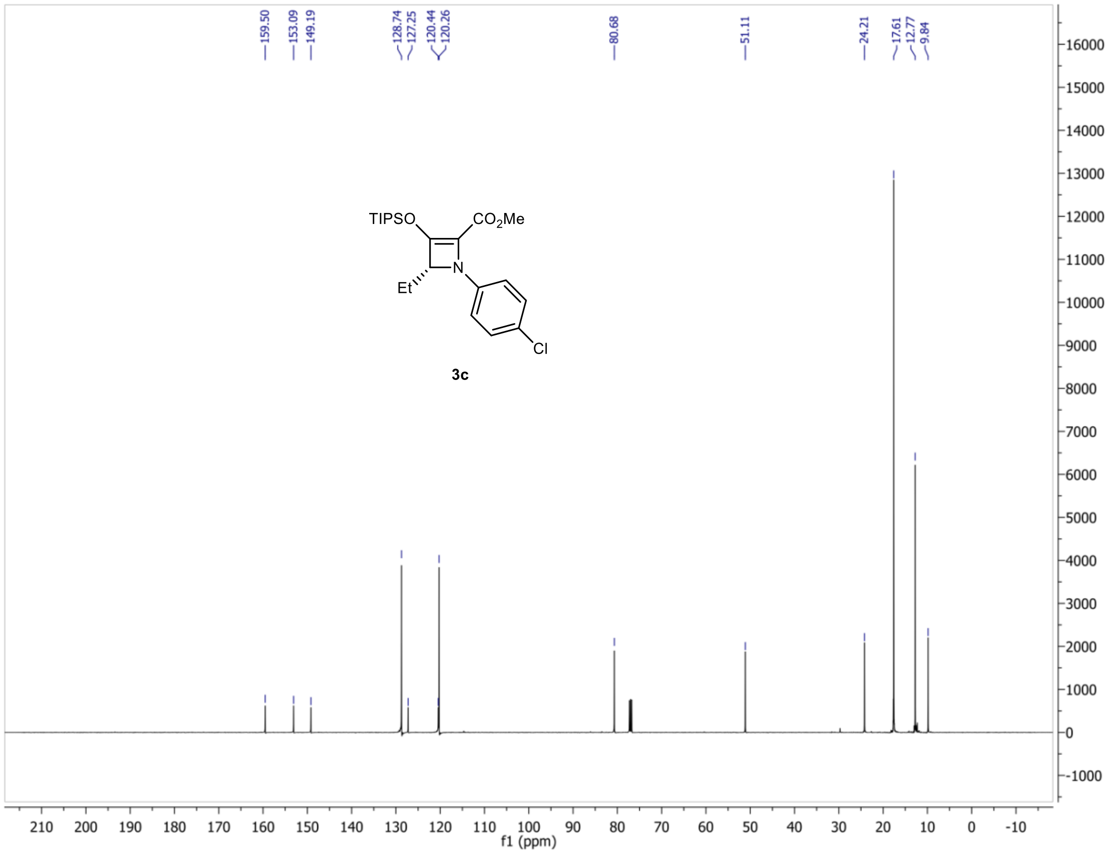

Supplementary Figure 16. ^{13}C NMR (126 MHz, $CDCl_3$) spectrum for 3c

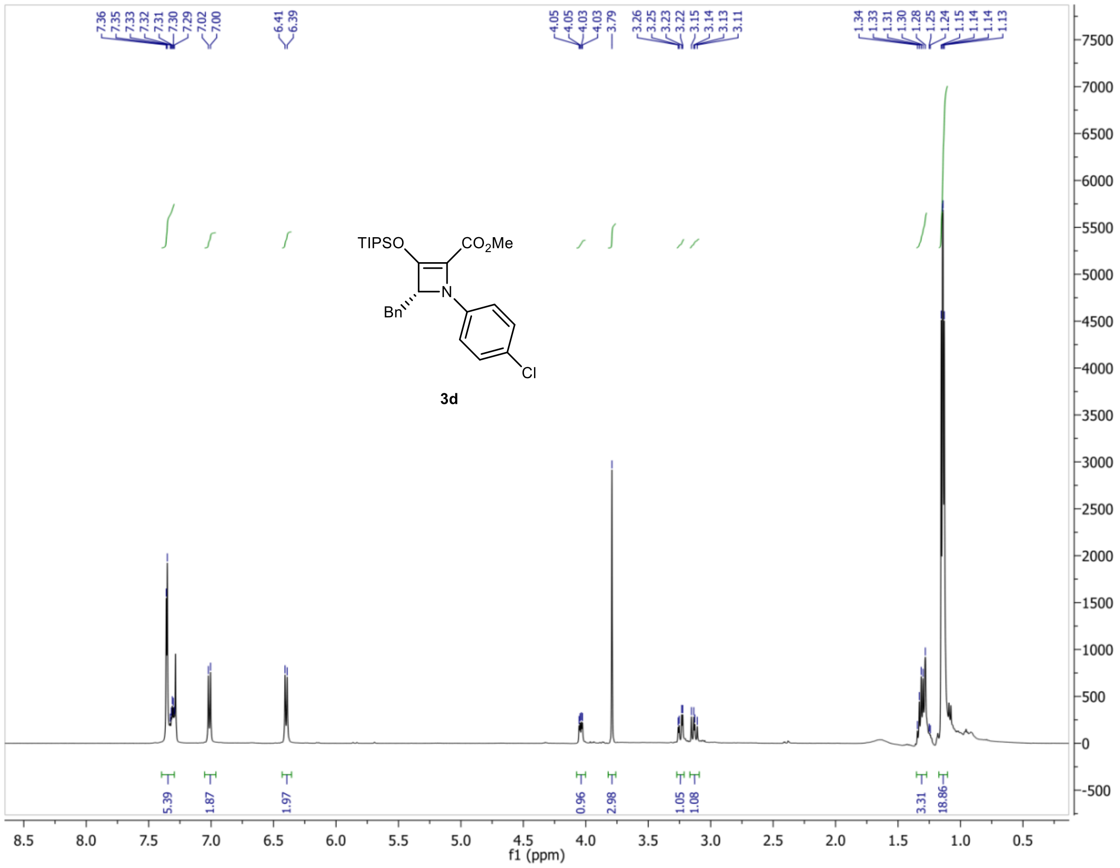

Supplementary Figure 17. ^{1}H NMR (500 MHz, $CDCl_3$) spectrum for 3d

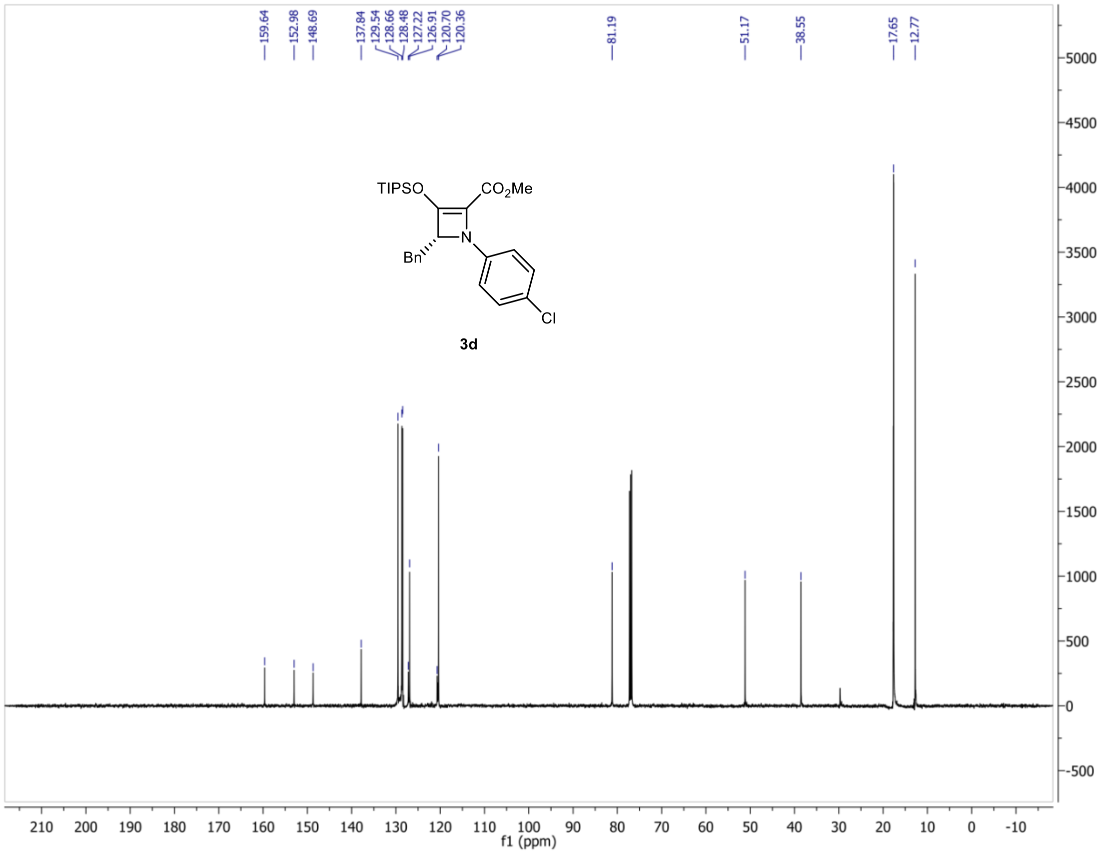

Supplementary Figure 18. ^{13}C NMR (126 MHz, $CDCl_3$) spectrum for 3d

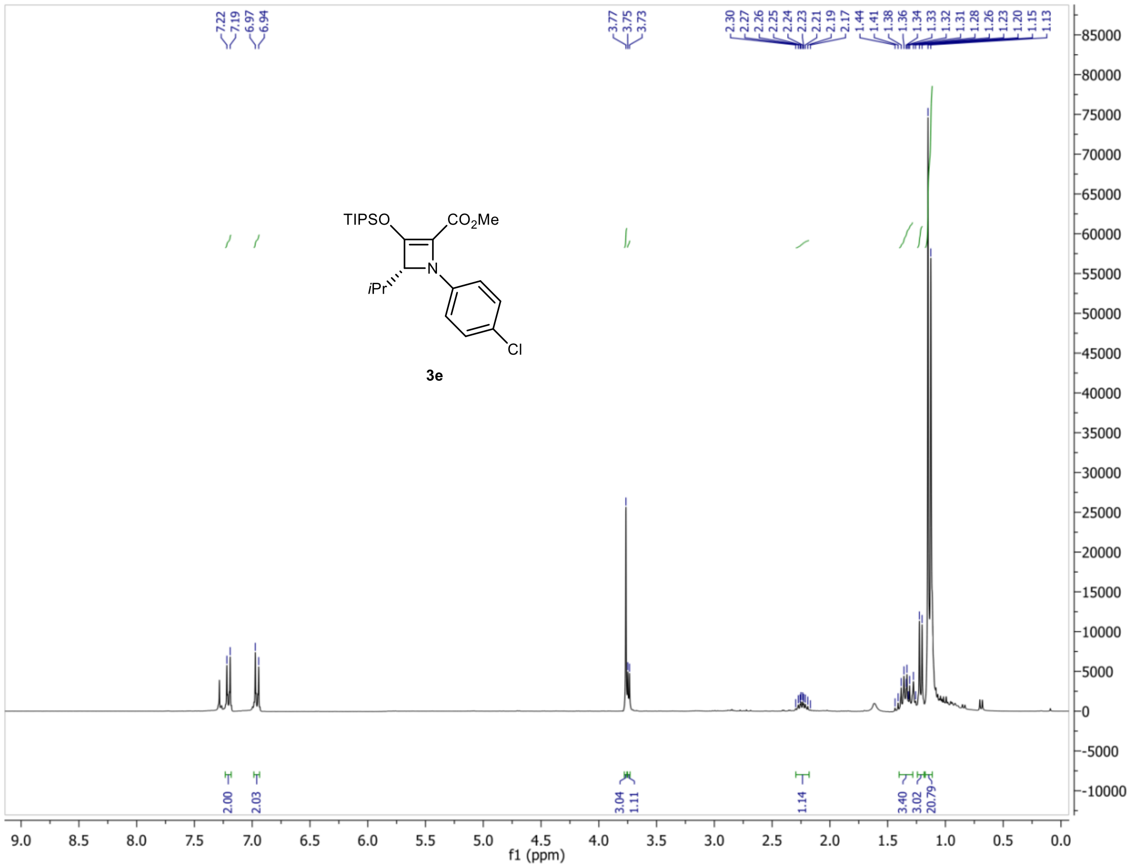

Supplementary Figure 19. ^{1}H **NMR (300 MHz,** $CDCl_3$**) spectrum for 3e**

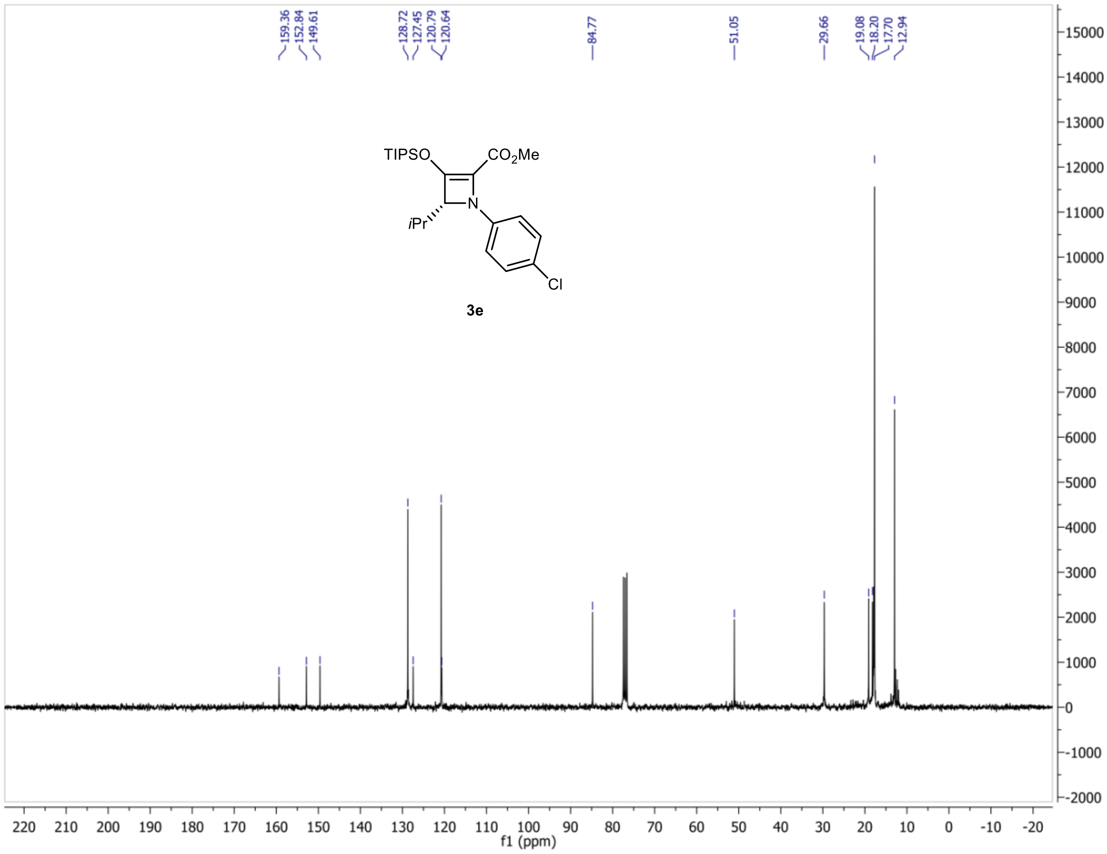

Supplementary Figure 20. ^{13}C **NMR (75 MHz,** $CDCl_3$**) spectrum for 3e**

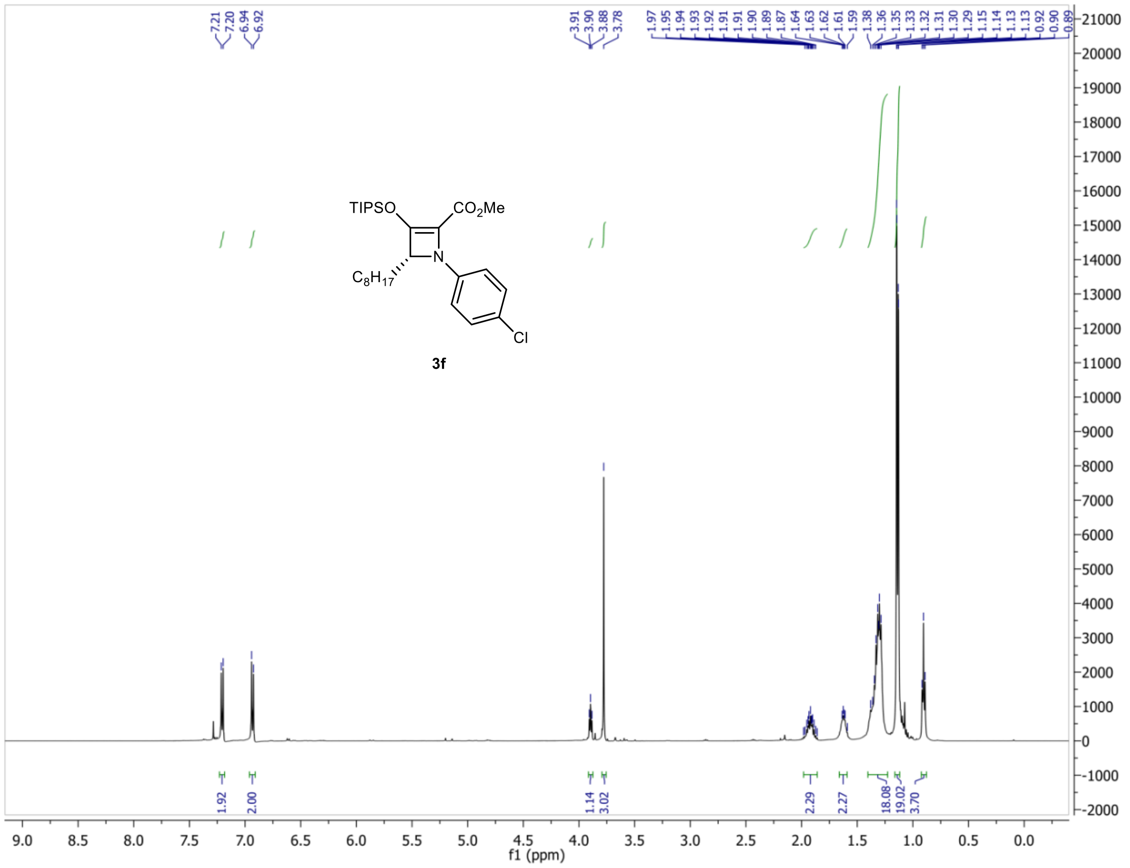

Supplementary Figure 21. ^{1}H NMR (500 MHz, $CDCl_3$) spectrum for 3f

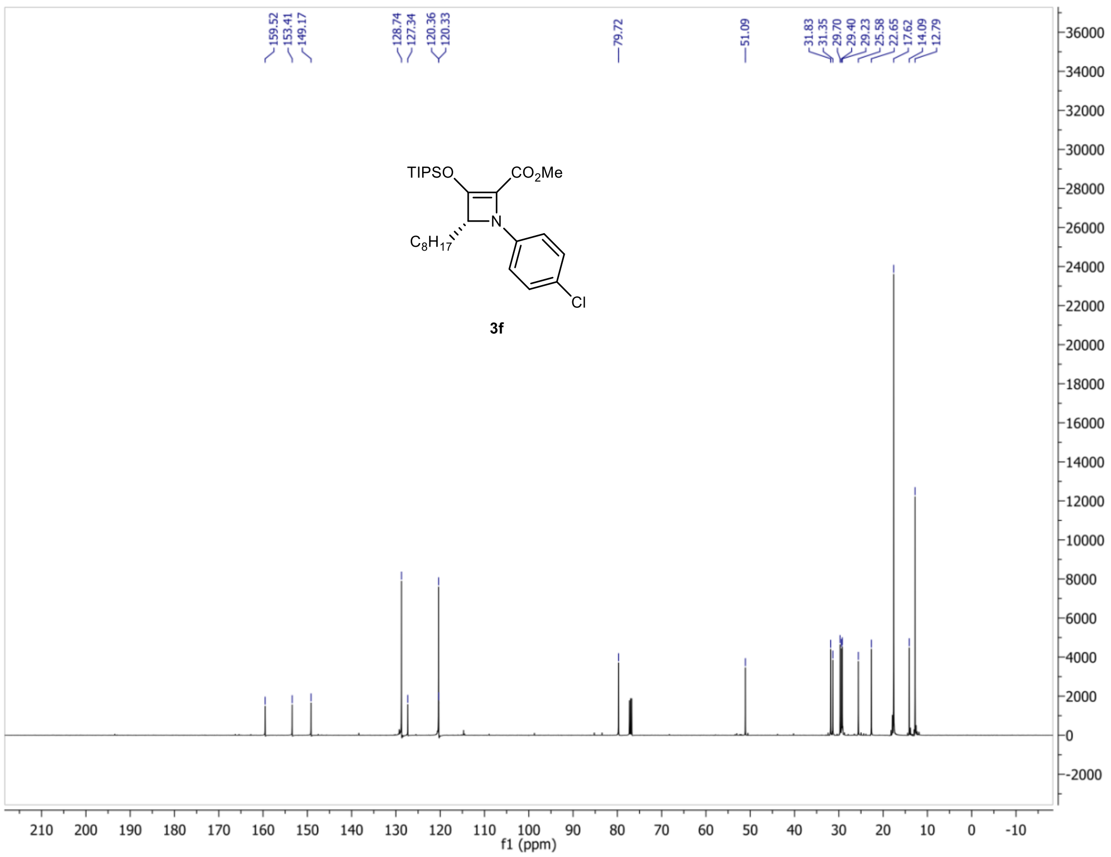

Supplementary Figure 22. ^{13}C NMR (126 MHz, $CDCl_3$) spectrum for 3f

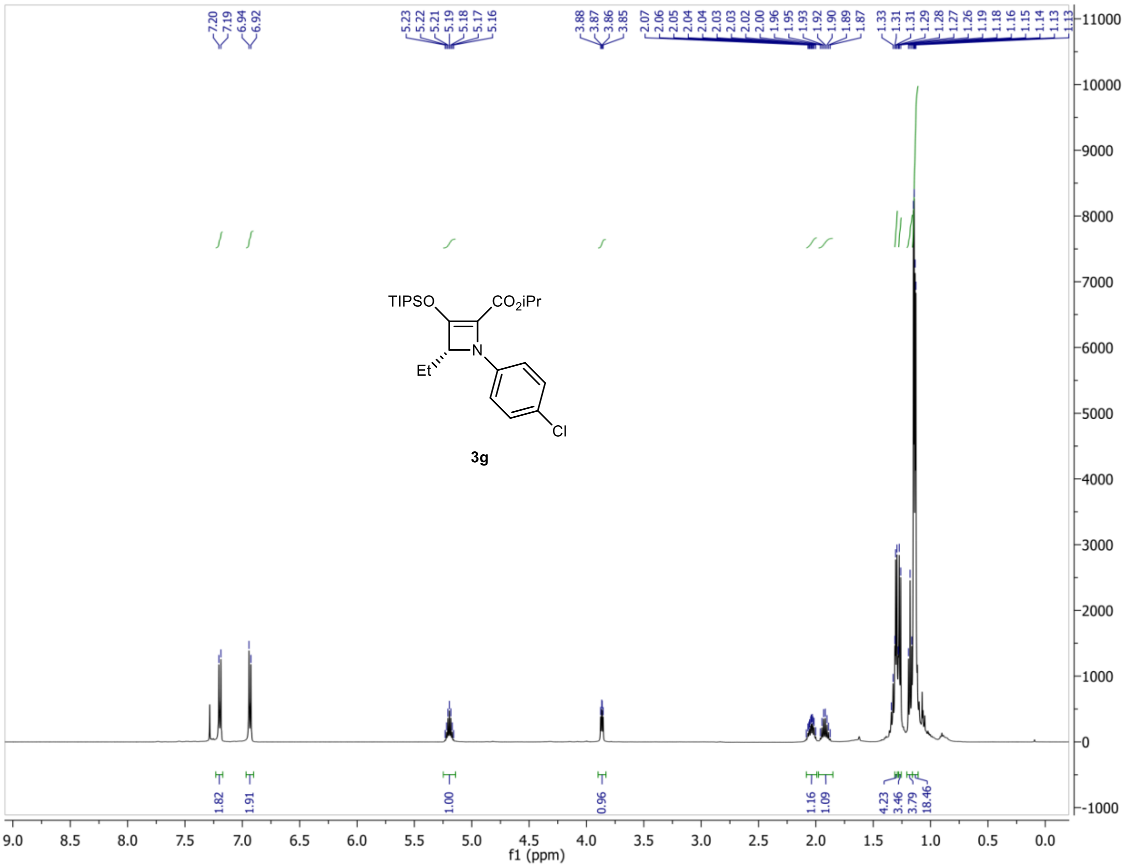

Supplementary Figure 23. ^{1}H NMR (500 MHz, $CDCl_3$) spectrum for 3g

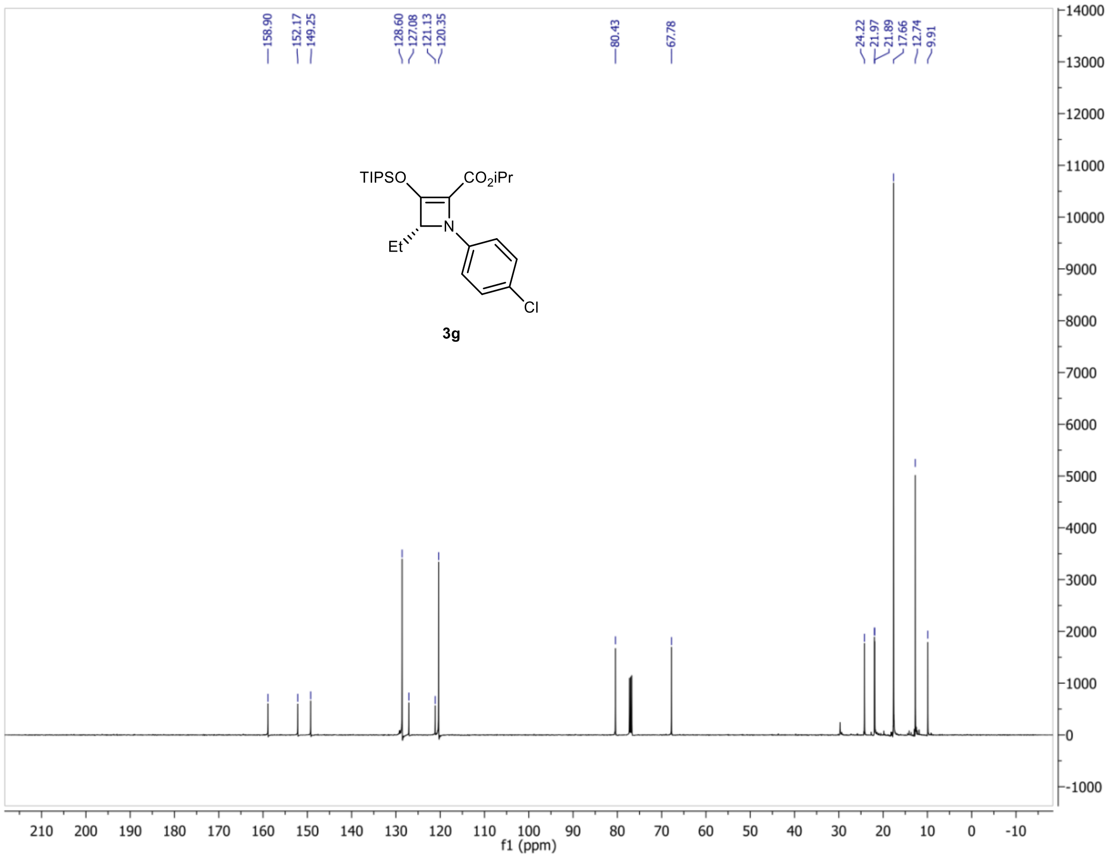

Supplementary Figure 24. ^{13}C NMR (126 MHz, $CDCl_3$) spectrum for 3g

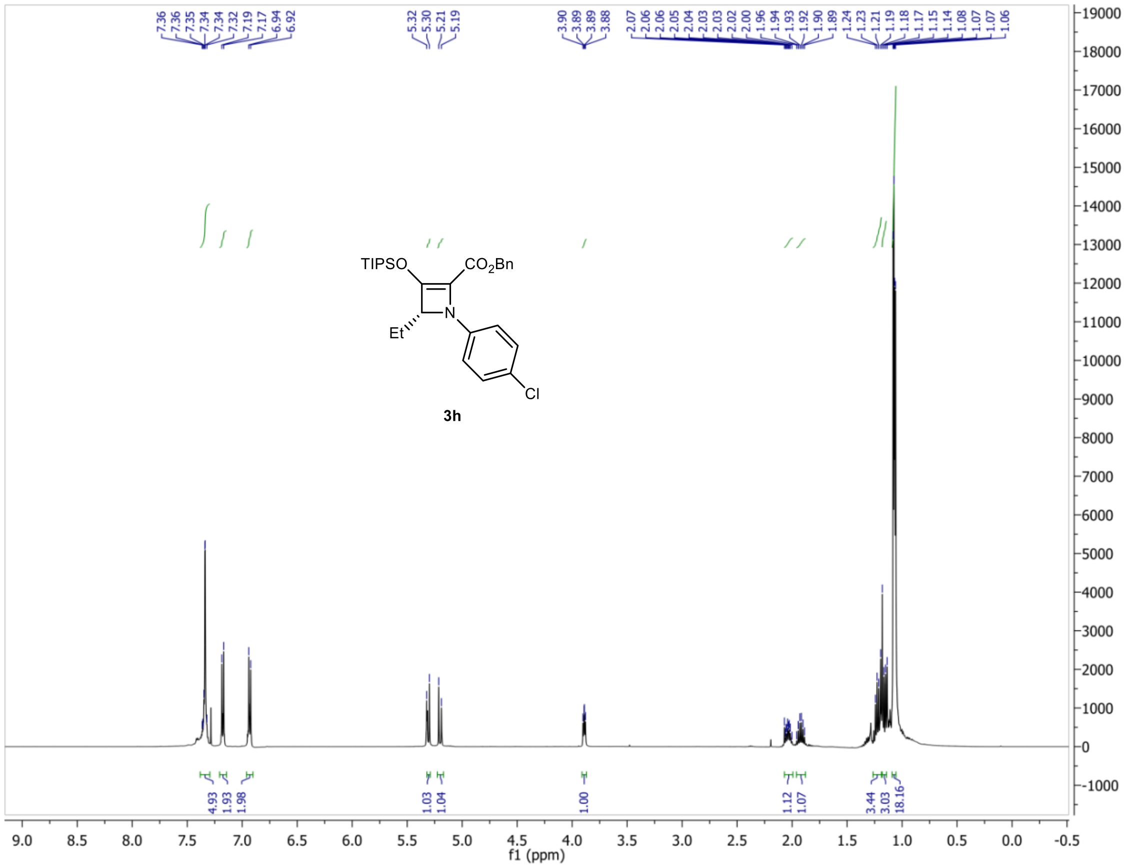

Supplementary Figure 25. ^1H **NMR (500 MHz,** $CDCl_3$**) spectrum for 3h**

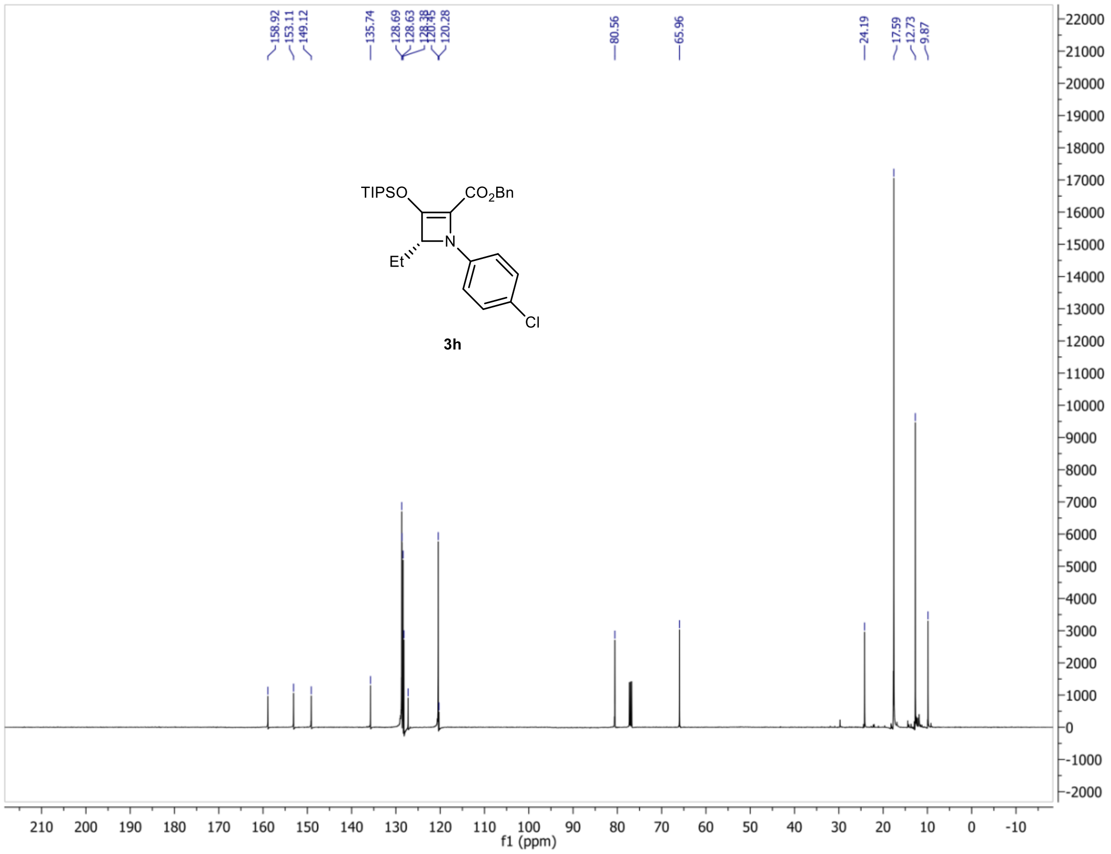

Supplementary Figure 26. ^{13}C **NMR (126 MHz,** $CDCl_3$**) spectrum for 3h**

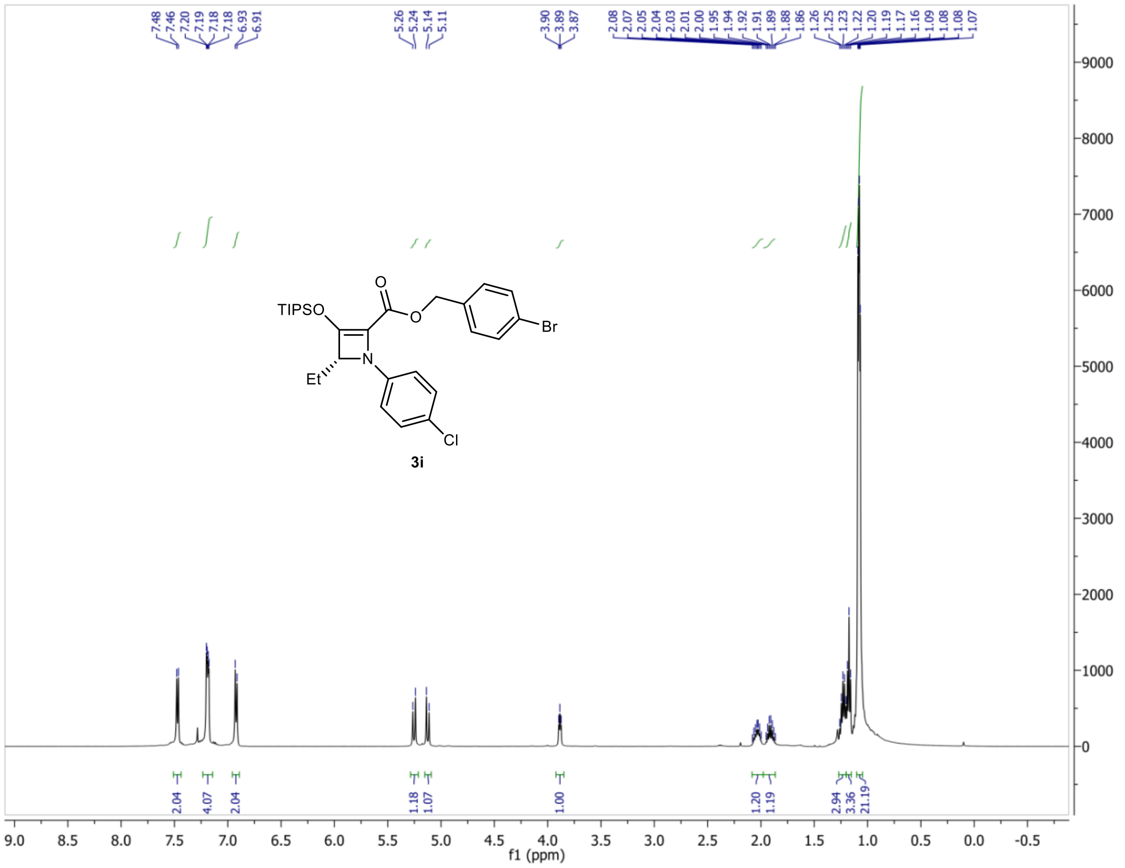

Supplementary Figure 27. ^{1}H **NMR (500 MHz,** $CDCl_3$**) spectrum for 3i**

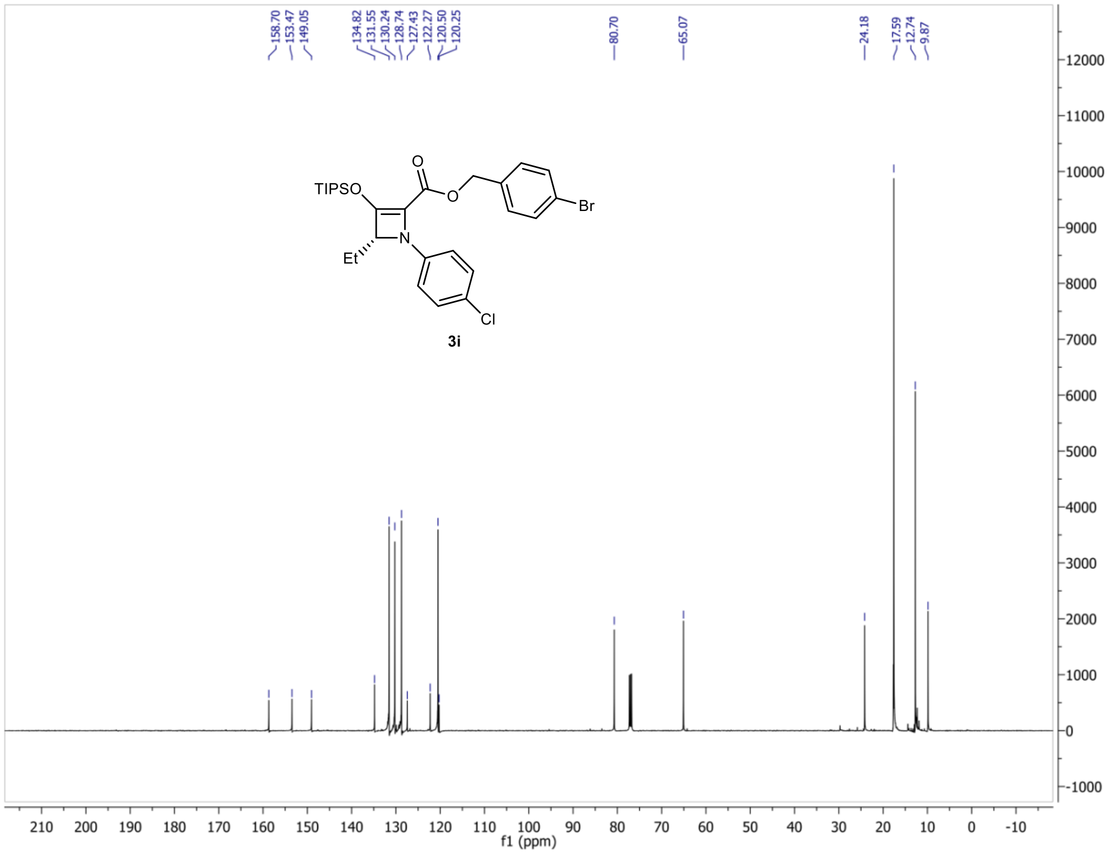

Supplementary Figure 28. ^{13}C **NMR (126 MHz,** $CDCl_3$**) spectrum for 3i**

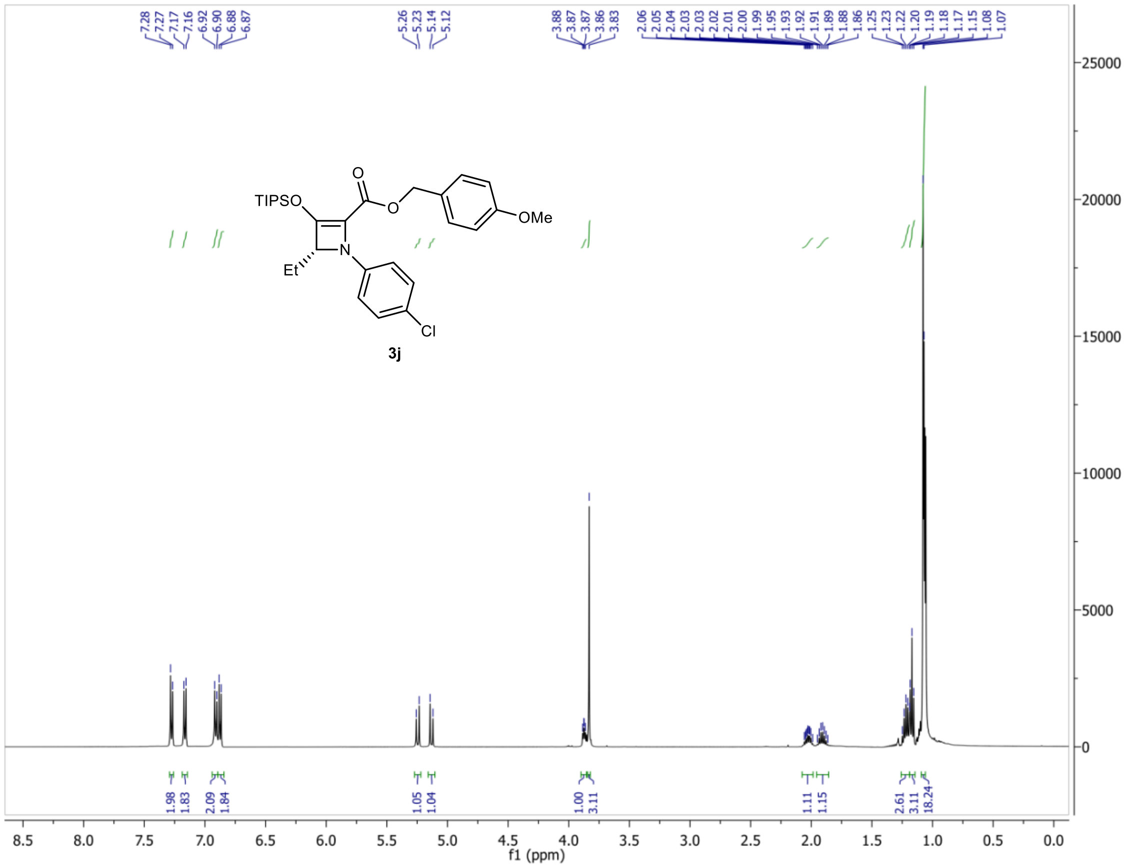

Supplementary Figure 29. ^{1}**H NMR (500 MHz, CDCl$_3$) spectrum for 3j**

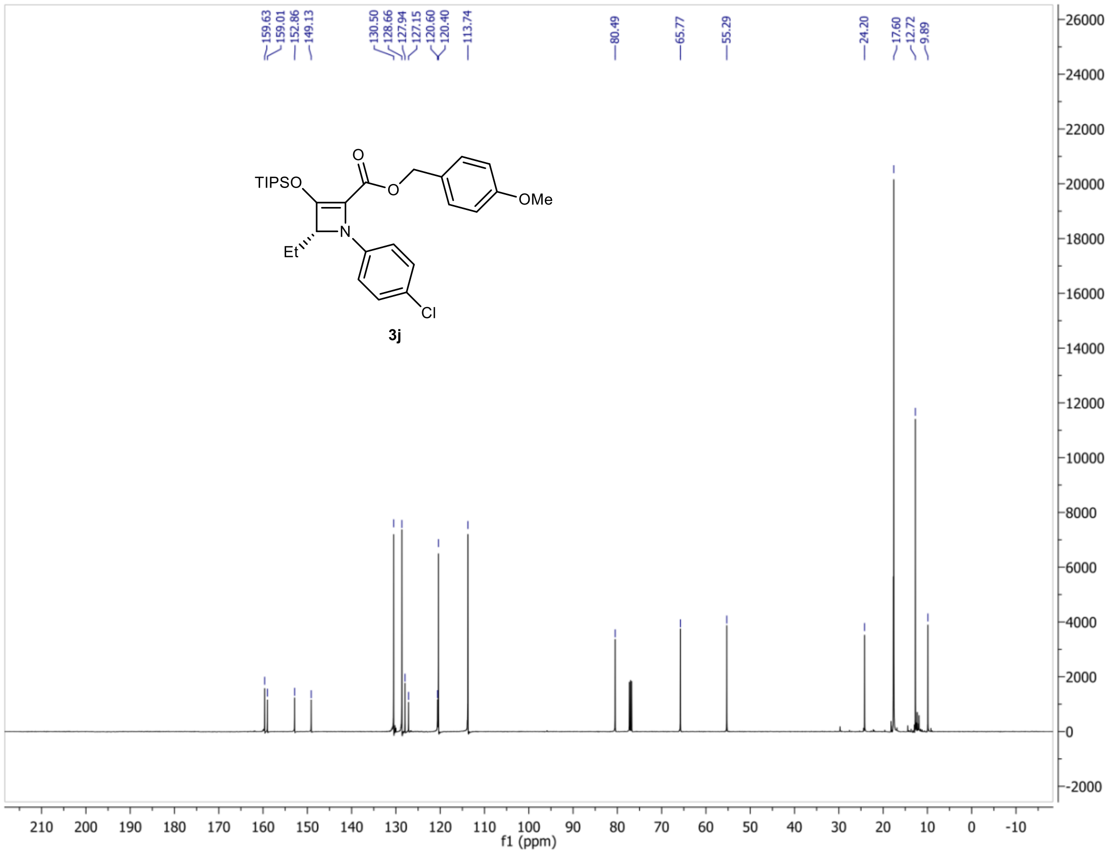

Supplementary Figure 30. ^{13}**C NMR (126 MHz, CDCl$_3$) spectrum for 3j**

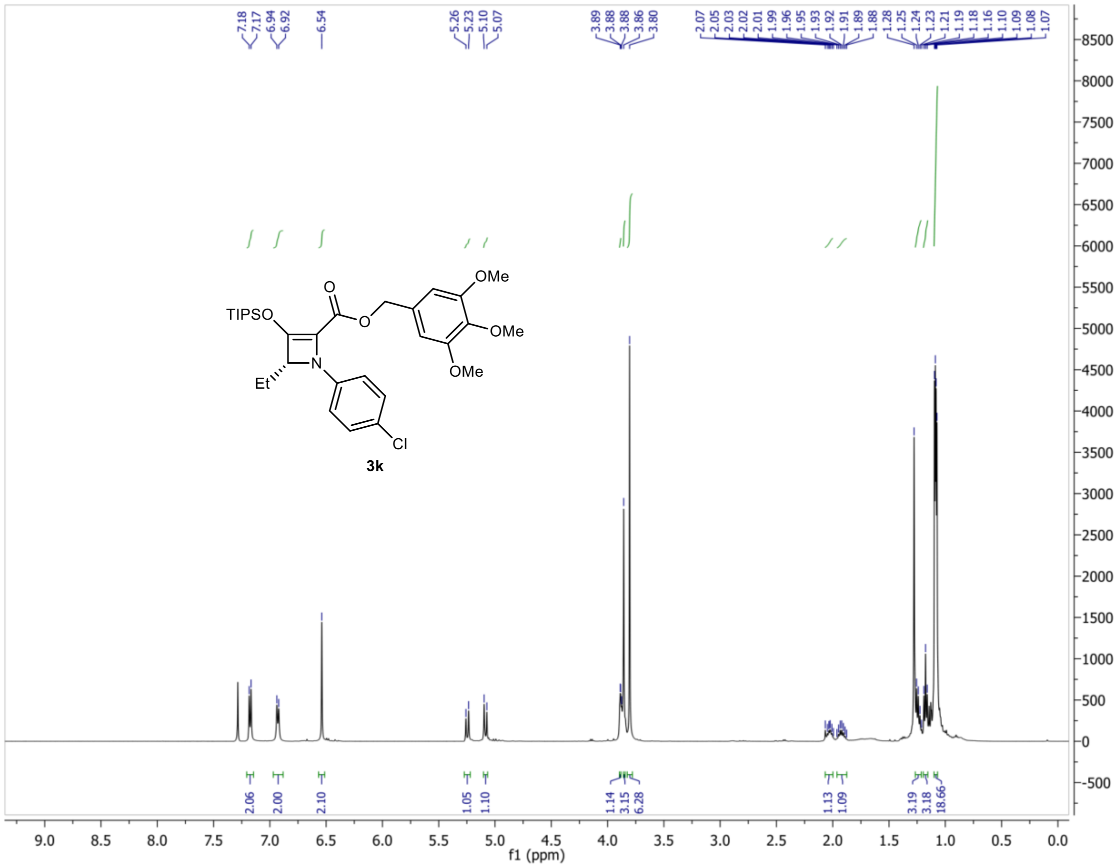

Supplementary Figure 31. ^{1}H NMR (500 MHz, $CDCl_3$) spectrum for 3k

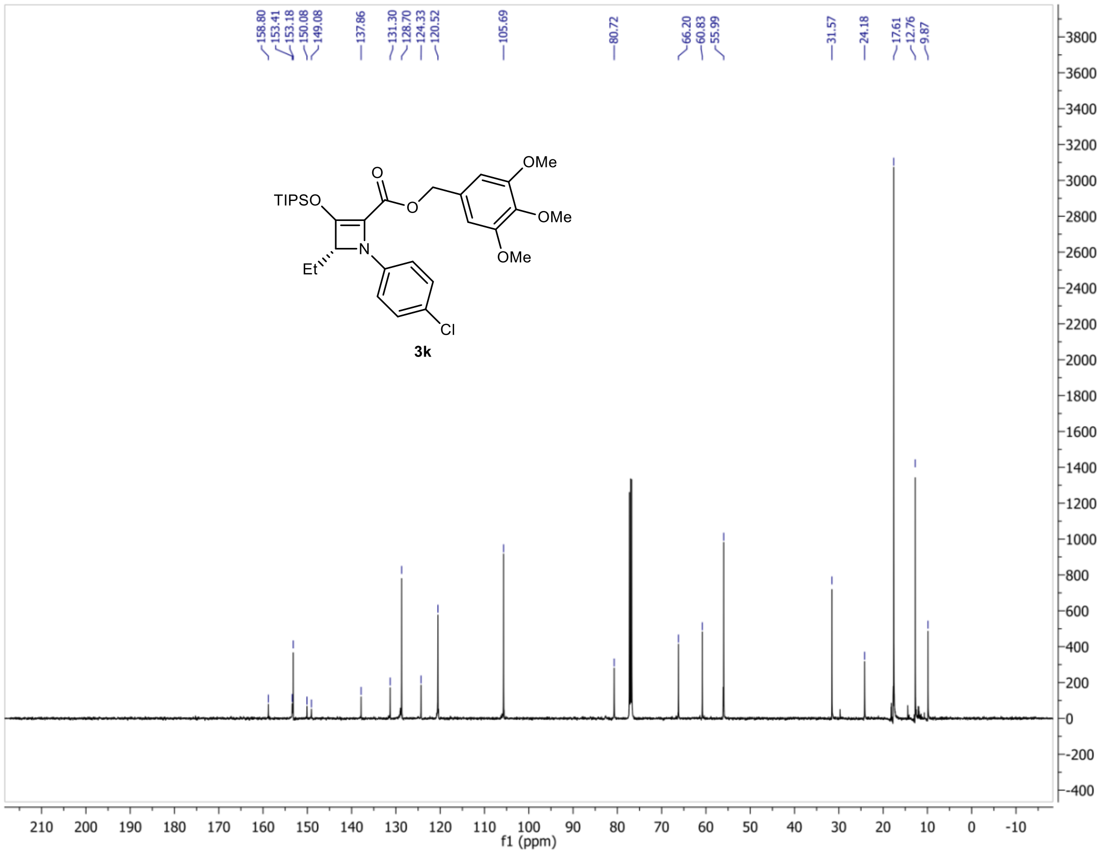

Supplementary Figure 32. ^{13}C NMR (126 MHz, $CDCl_3$) spectrum for 3k

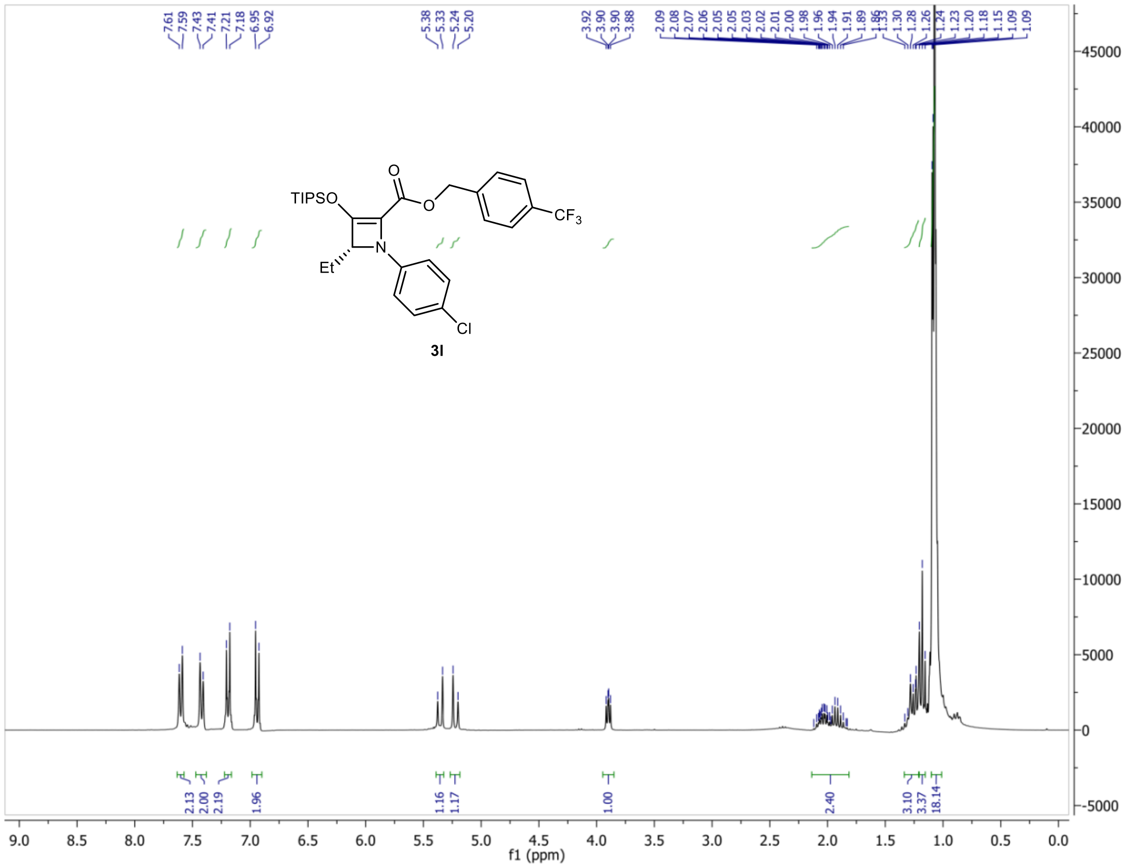

Supplementary Figure 33. ^{1}H NMR (300 MHz, $CDCl_3$) spectrum for 3l

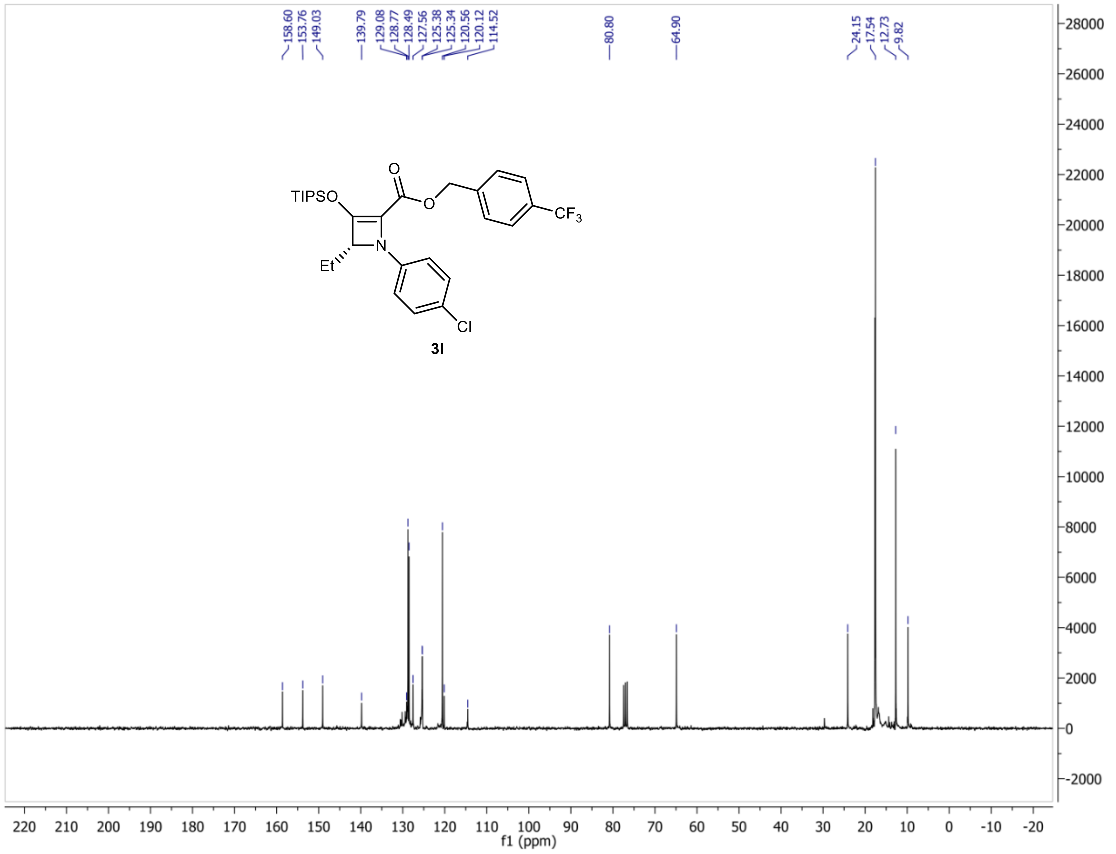

Supplementary Figure 34. ^{13}C NMR (75 MHz, $CDCl_3$) spectrum for 3l

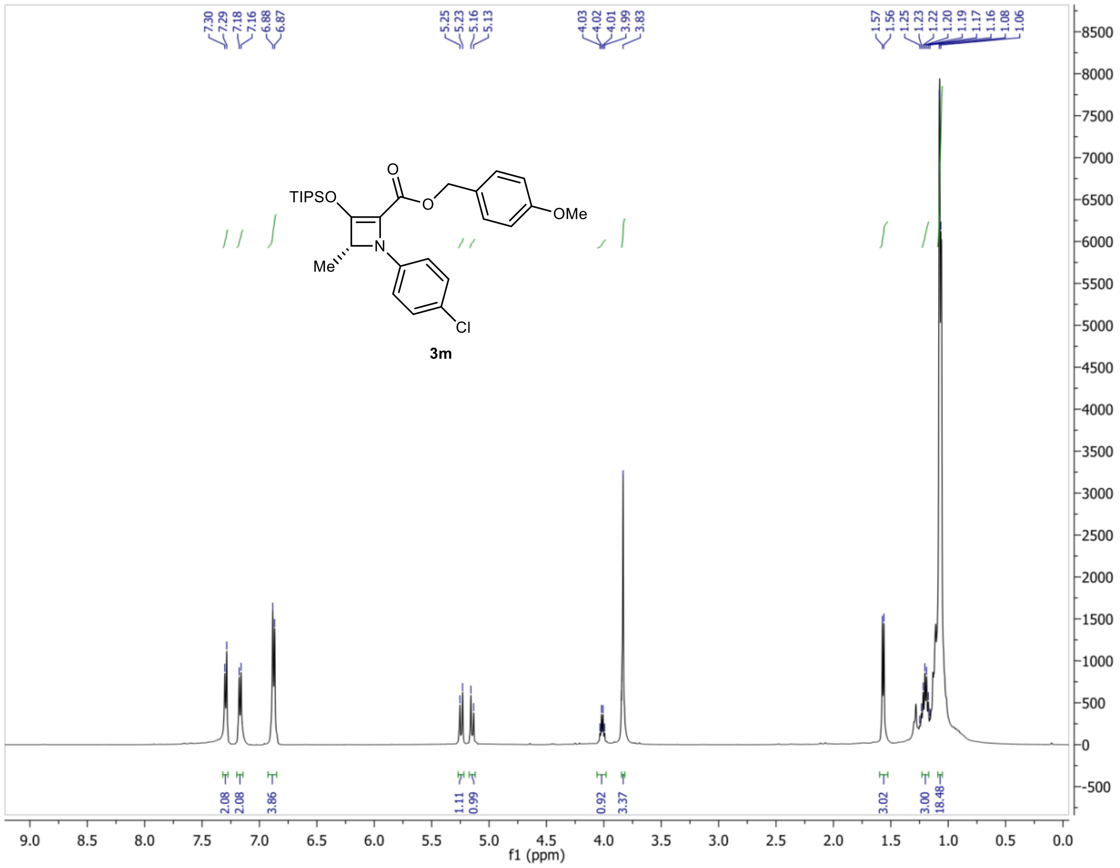

Supplementary Figure 35. ^{1}H NMR (500 MHz, $CDCl_3$) spectrum for 3m

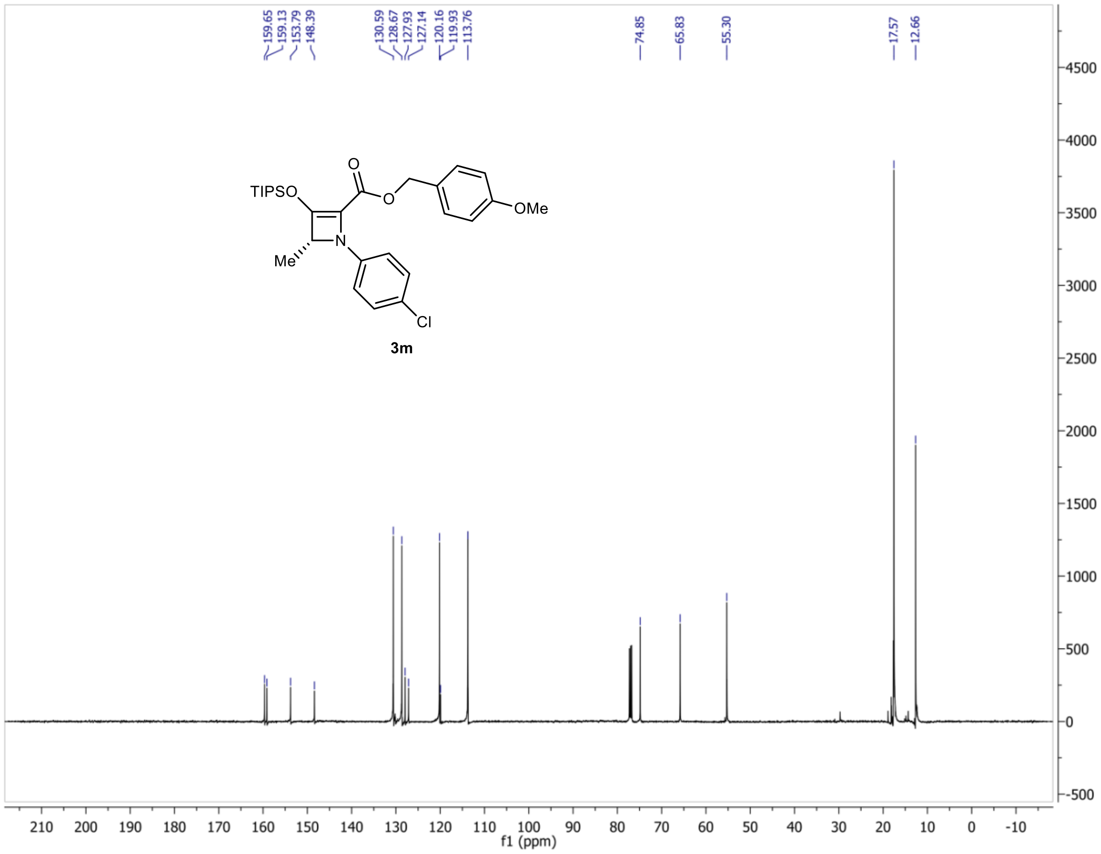

Supplementary Figure 36. ^{13}C NMR (126 MHz, $CDCl_3$) spectrum for 3m

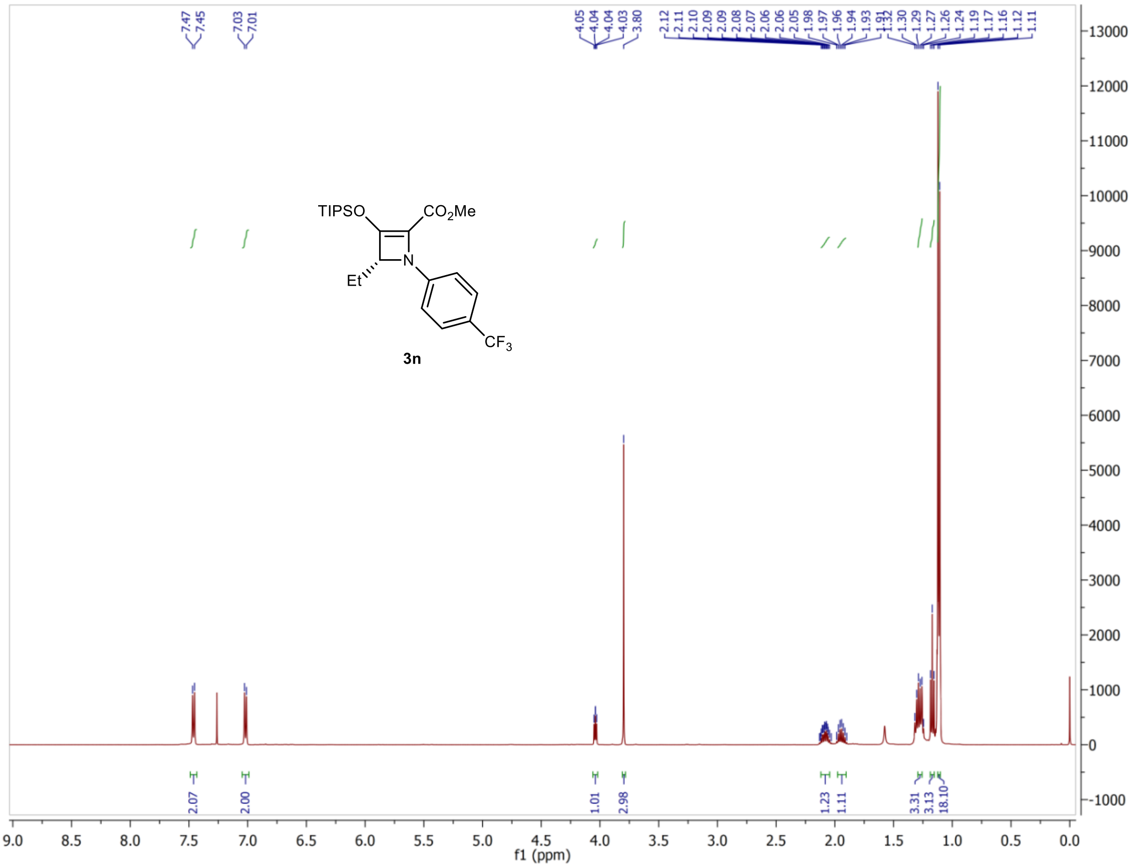

Supplementary Figure 37. ^{1}H NMR (500 MHz, $CDCl_3$) spectrum for 3n

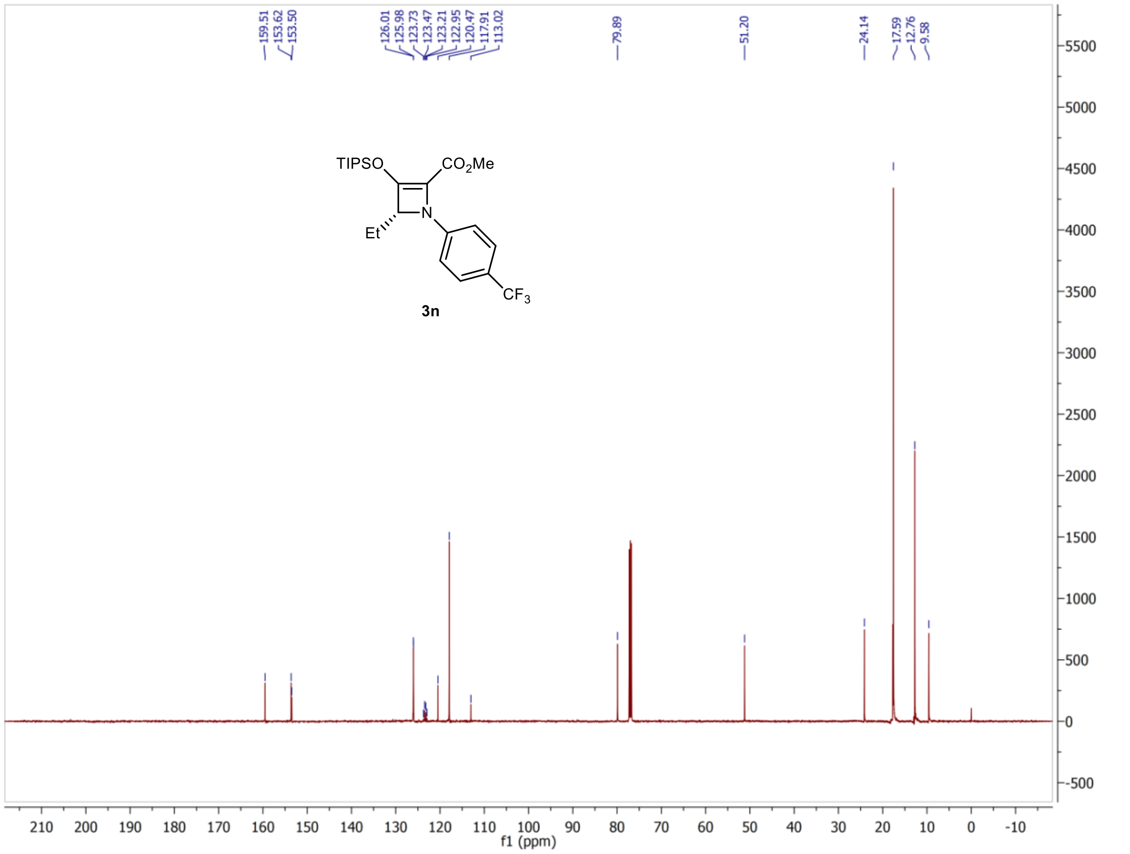

Supplementary Figure 38. ^{13}C NMR (126 MHz, $CDCl_3$) spectrum for 3n

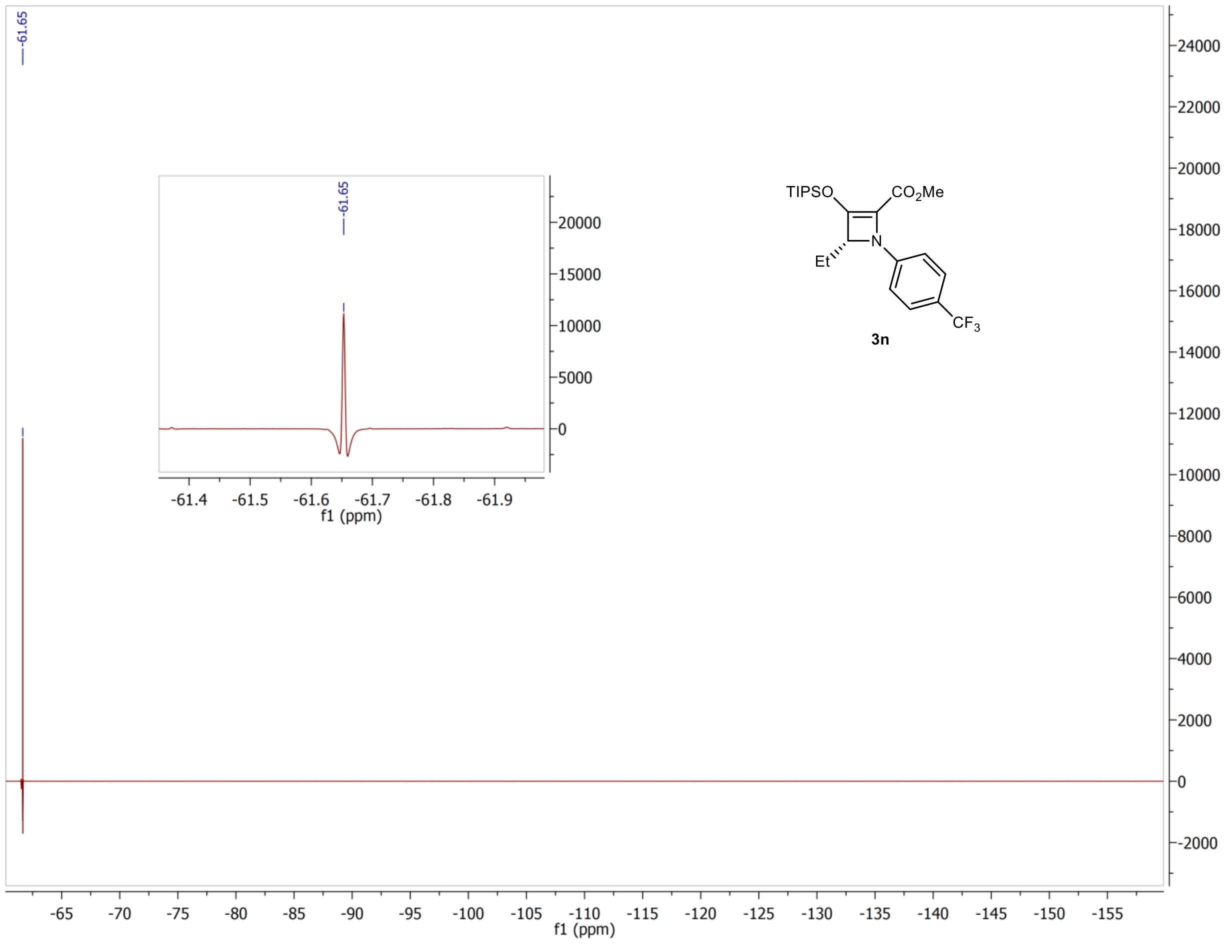

Supplementary Figure 39. ^{19}F NMR (471 MHz, $CDCl_3$) spectrum for 3n

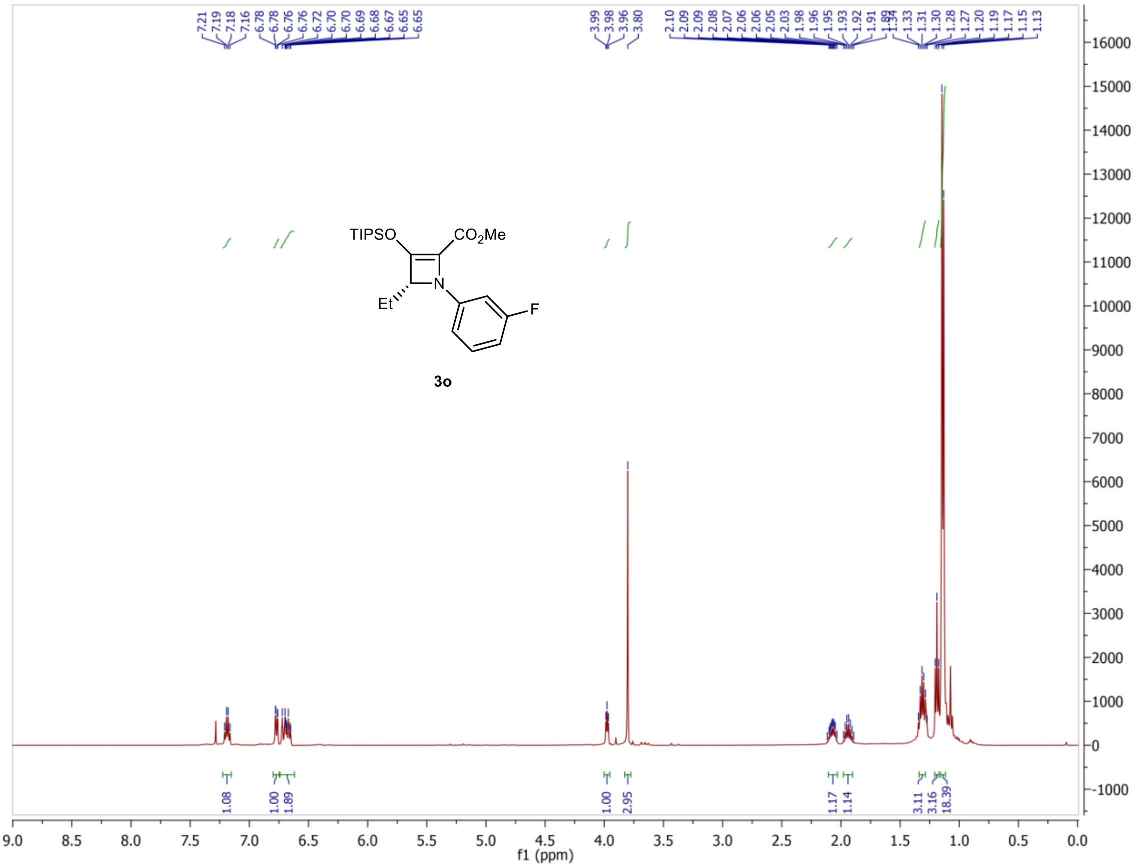

Supplementary Figure 40. ^{1}H NMR (500 MHz, $CDCl_3$) spectrum for 3o

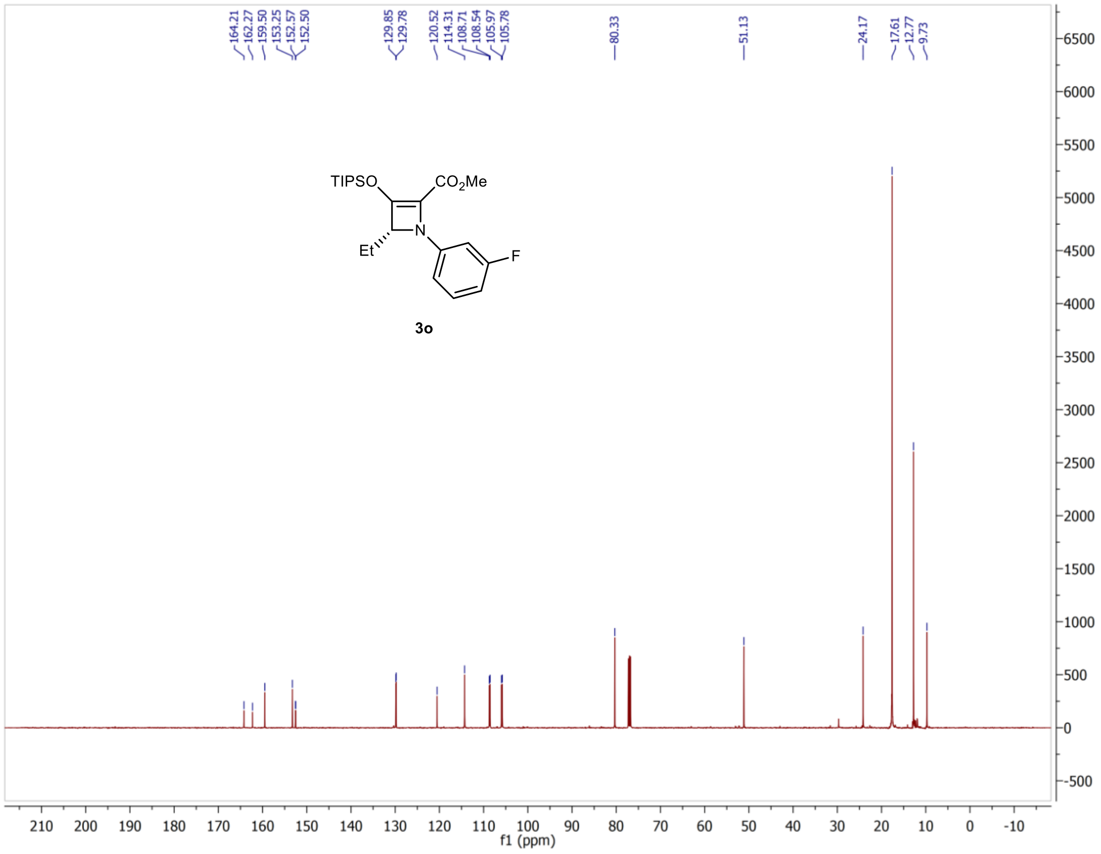

Supplementary Figure 41. ^{13}C NMR (126 MHz, $CDCl_3$) spectrum for 3o

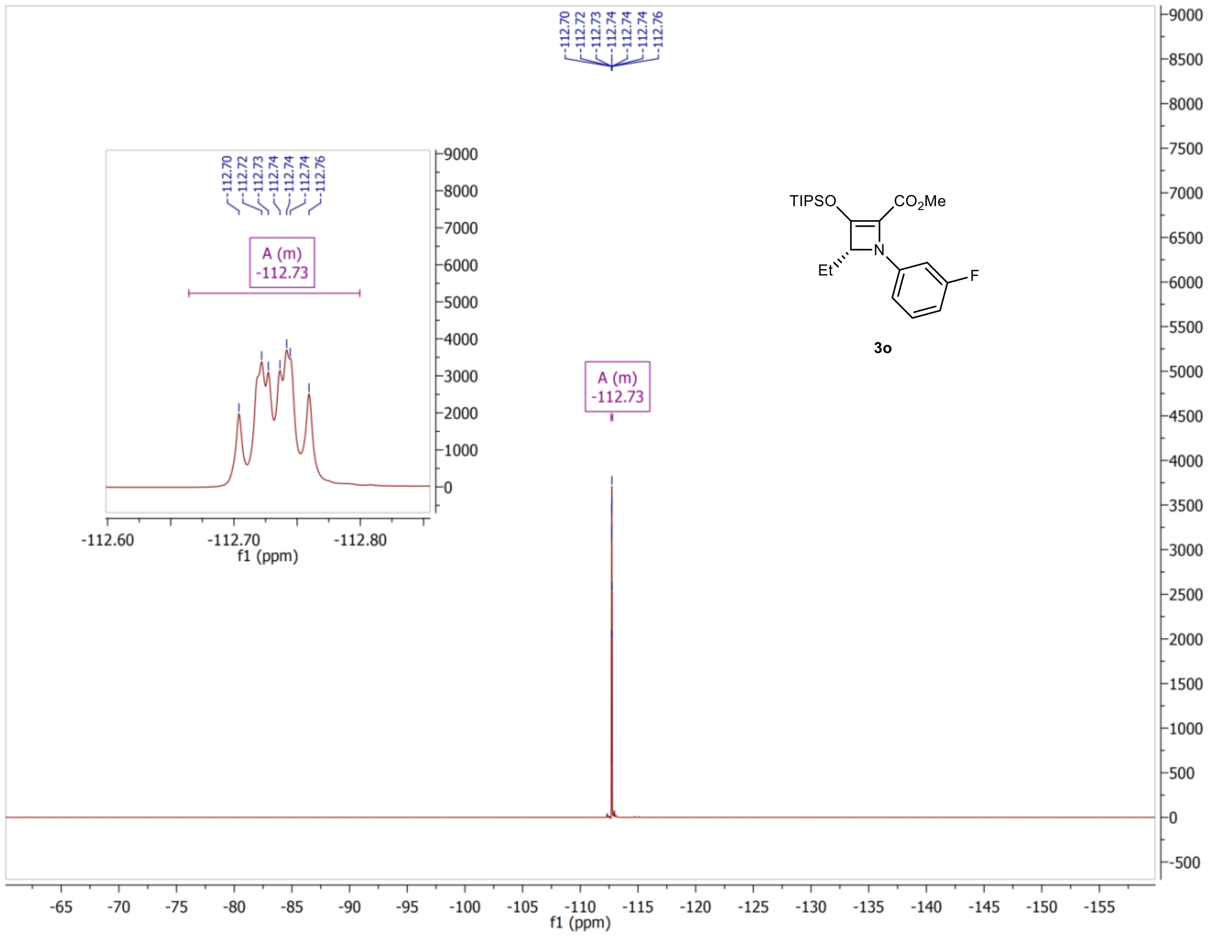

Supplementary Figure 42. ^{19}F NMR (471 MHz, $CDCl_3$) spectrum for 3o

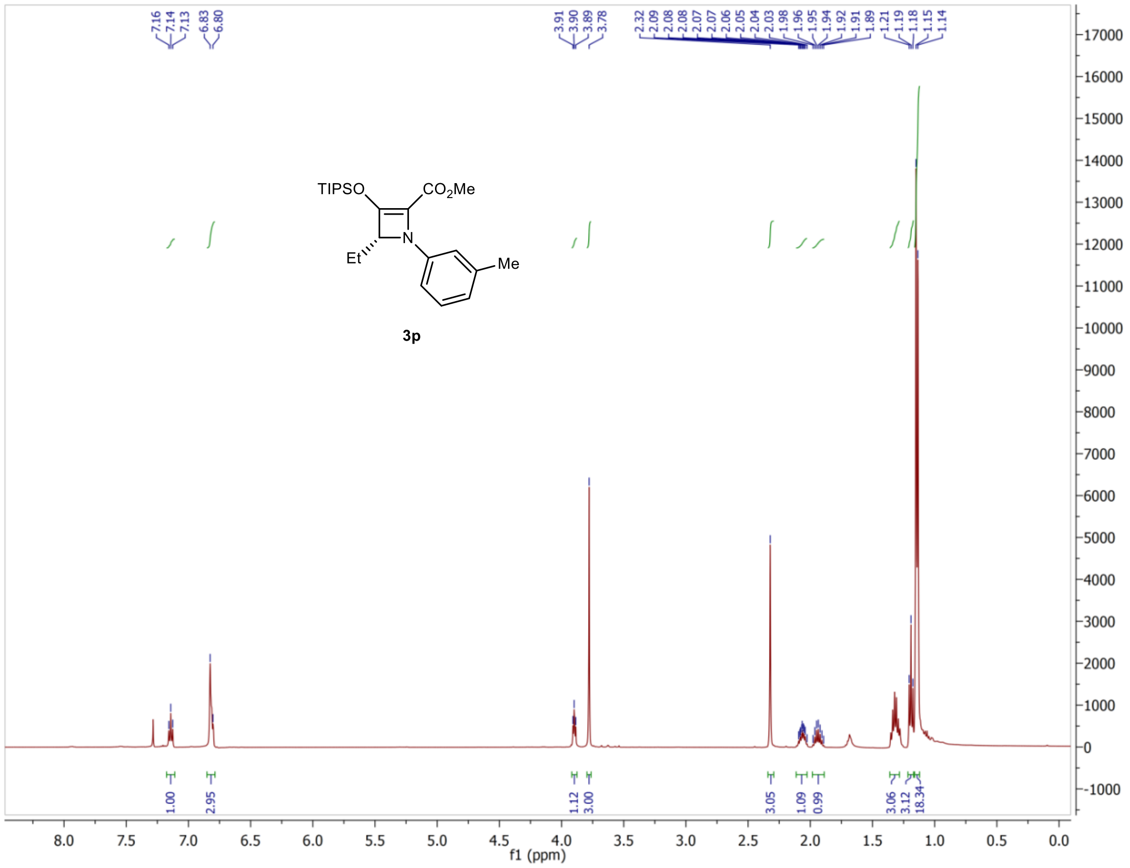

Supplementary Figure 43. ^{1}H NMR (500 MHz, $CDCl_3$) spectrum for 3p

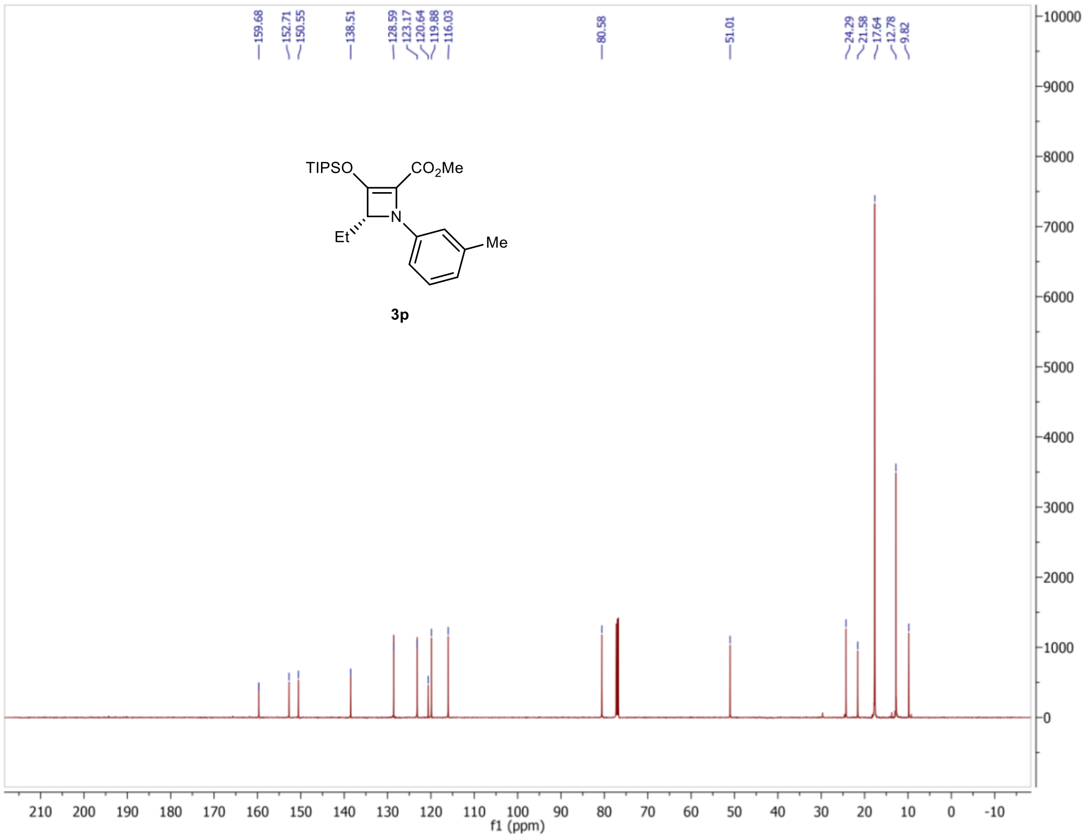

Supplementary Figure 44. ^{13}C NMR (126 MHz, $CDCl_3$) spectrum for 3p

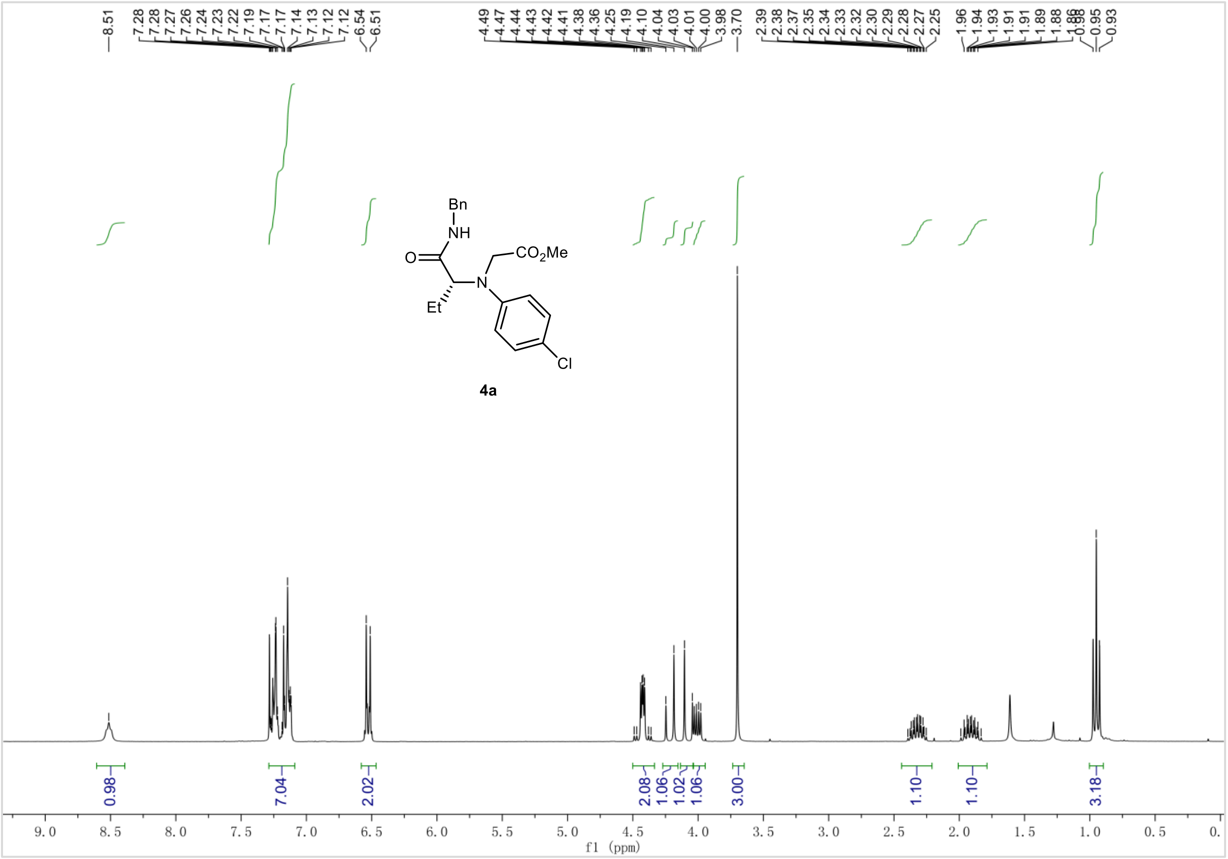

Supplementary Figure 45. ^{1}H NMR (300 MHz, $CDCl_3$) spectrum for 4a

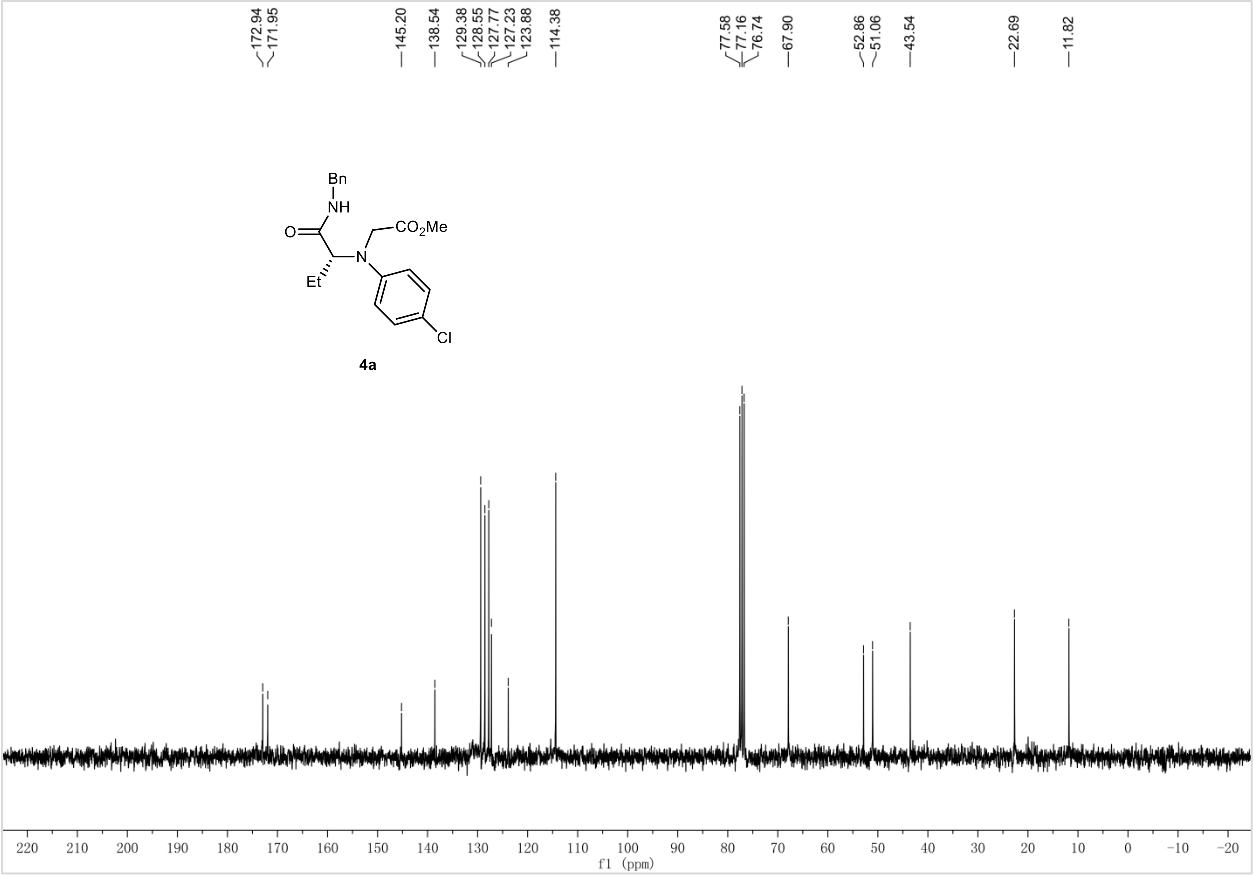

Supplementary Figure 46. ^{13}C NMR (75 MHz, $CDCl_3$) spectrum for 4a

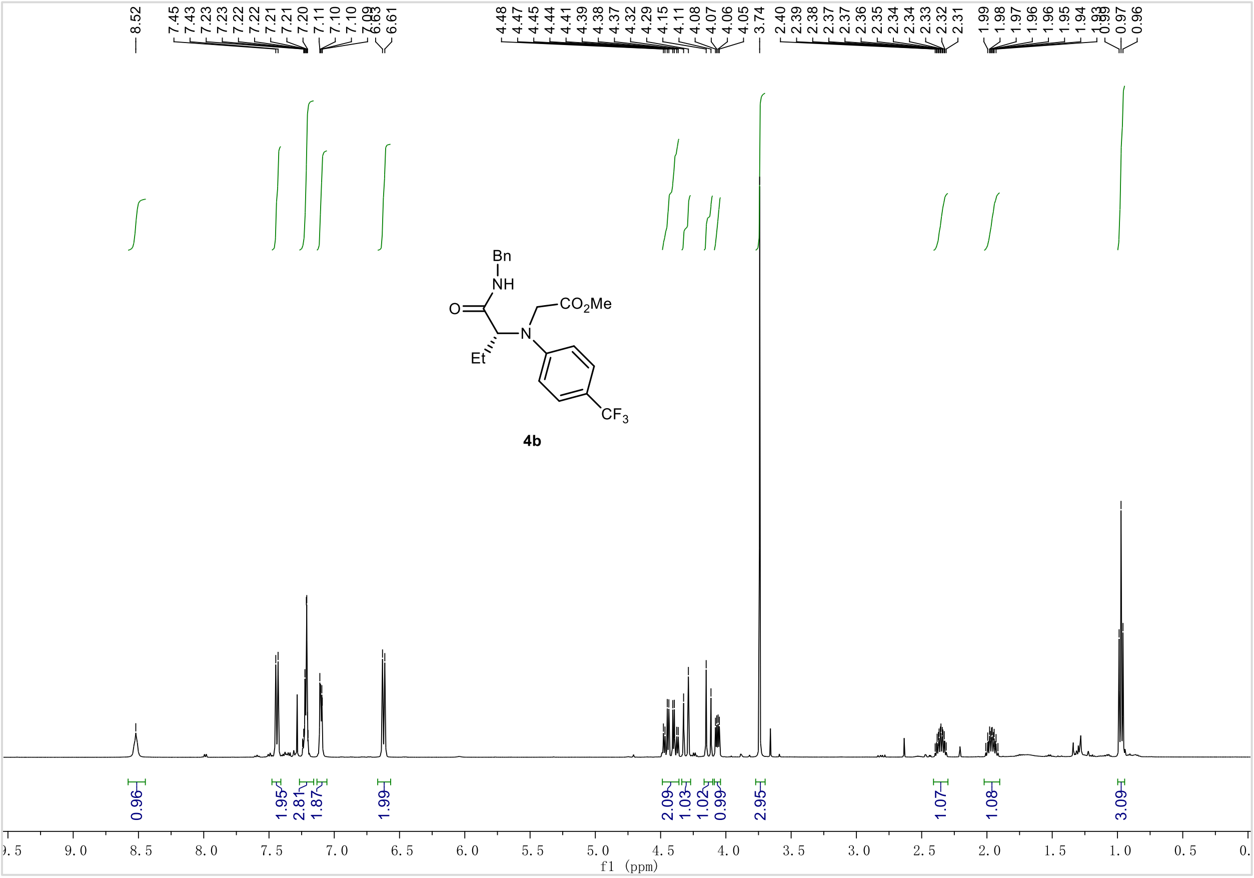

Supplementary Figure 47. ^{1}H NMR (500 MHz, $CDCl_3$) spectrum for 4b

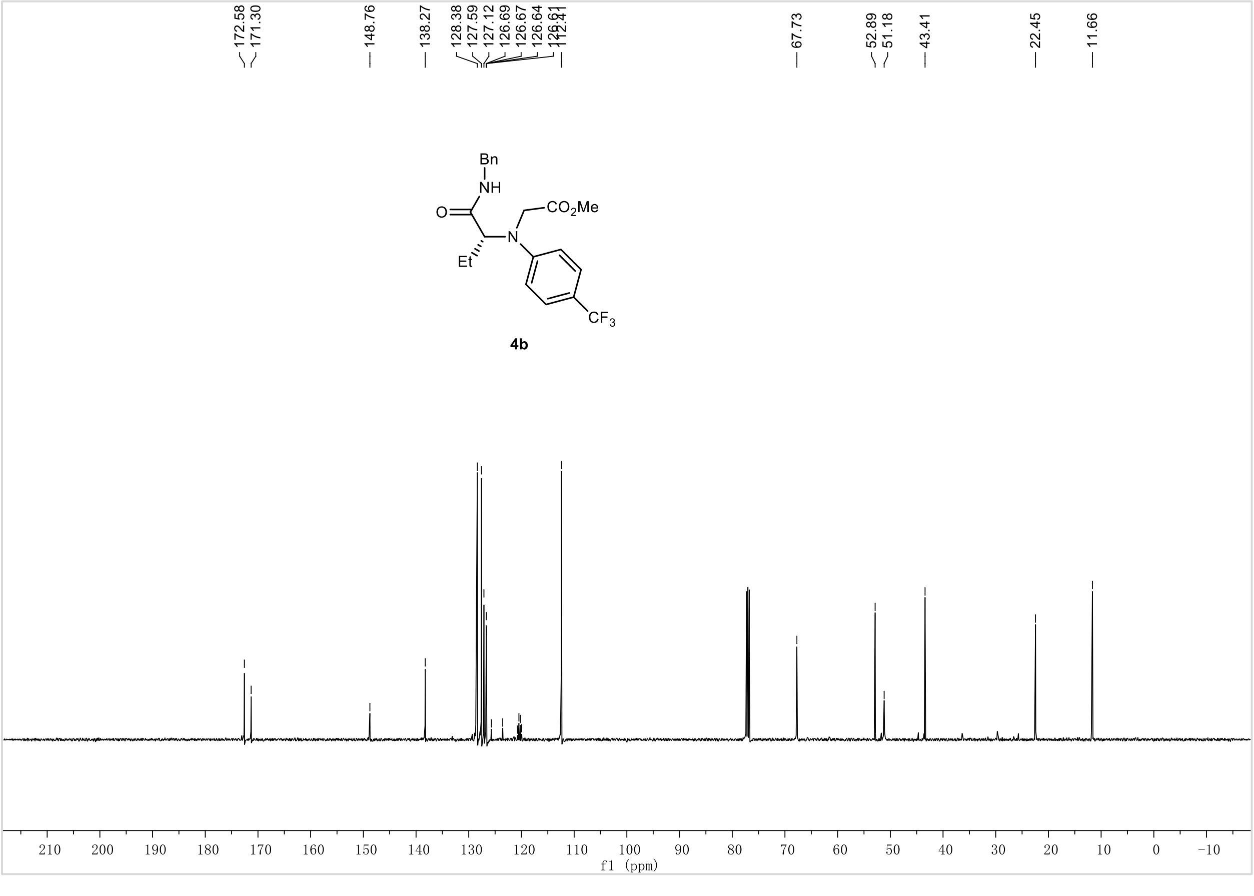

Supplementary Figure 48. ^{13}C NMR (126 MHz, $CDCl_3$) spectrum for 4b

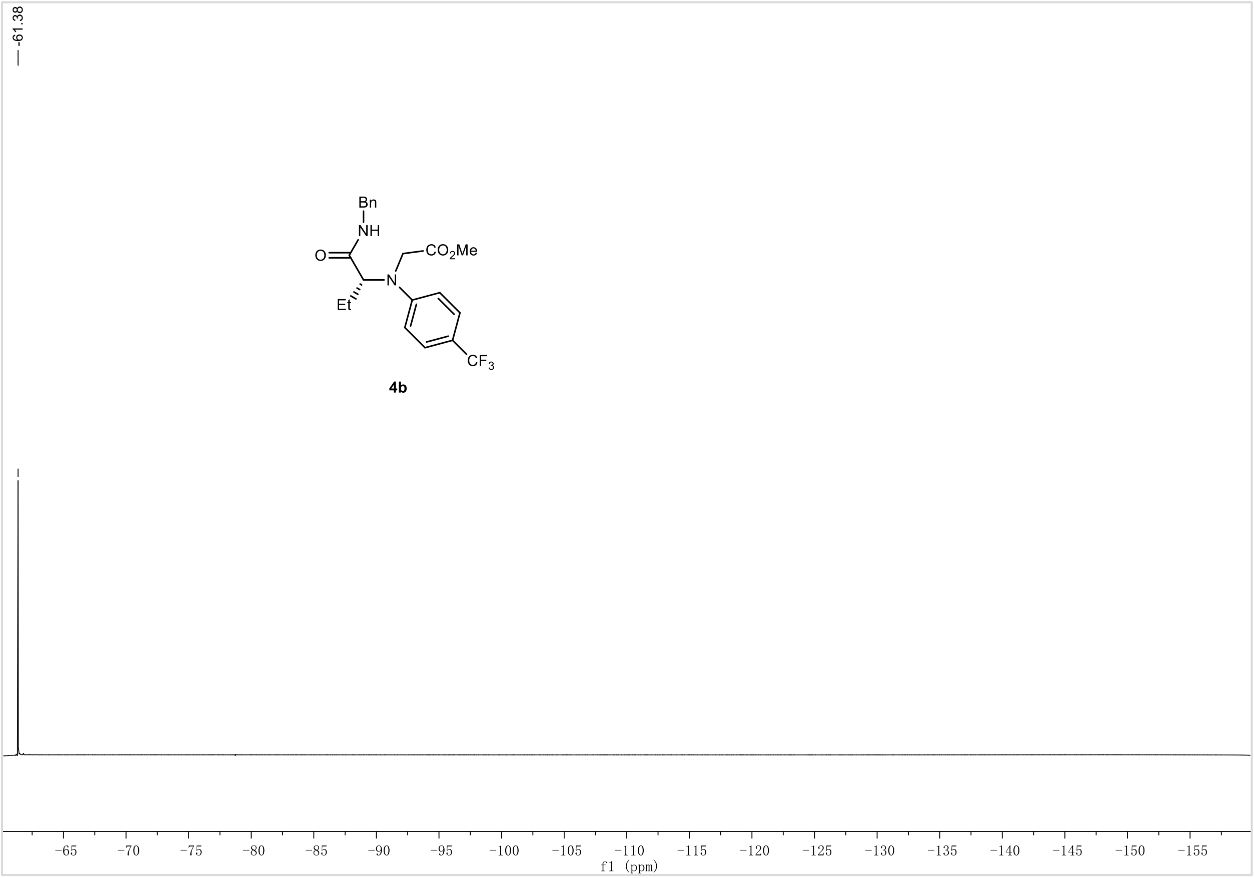

Supplementary Figure 49. ^{19}F NMR (471 MHz, $CDCl_3$) spectrum for 4b

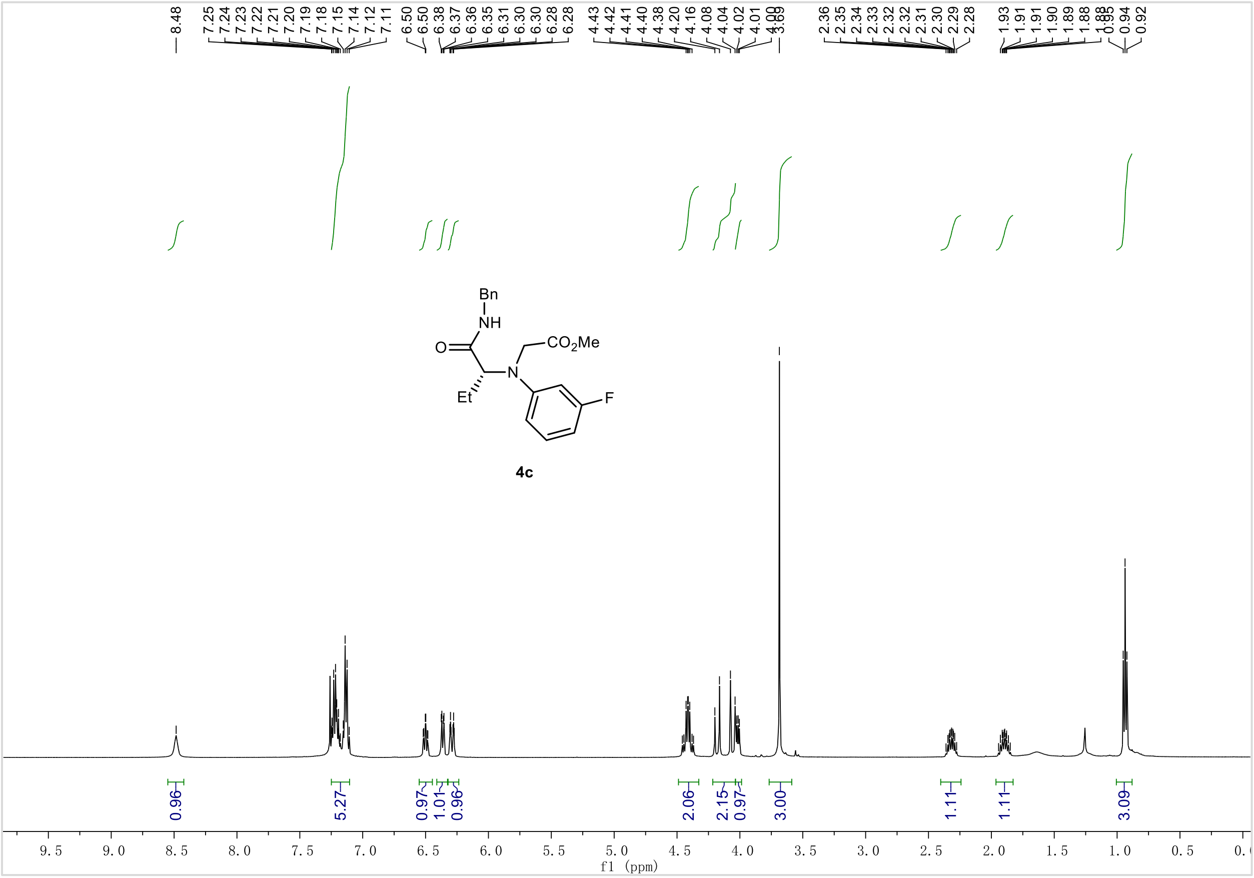

Supplementary Figure 50. ^{1}H NMR (500 MHz, $CDCl_3$) spectrum for 4c

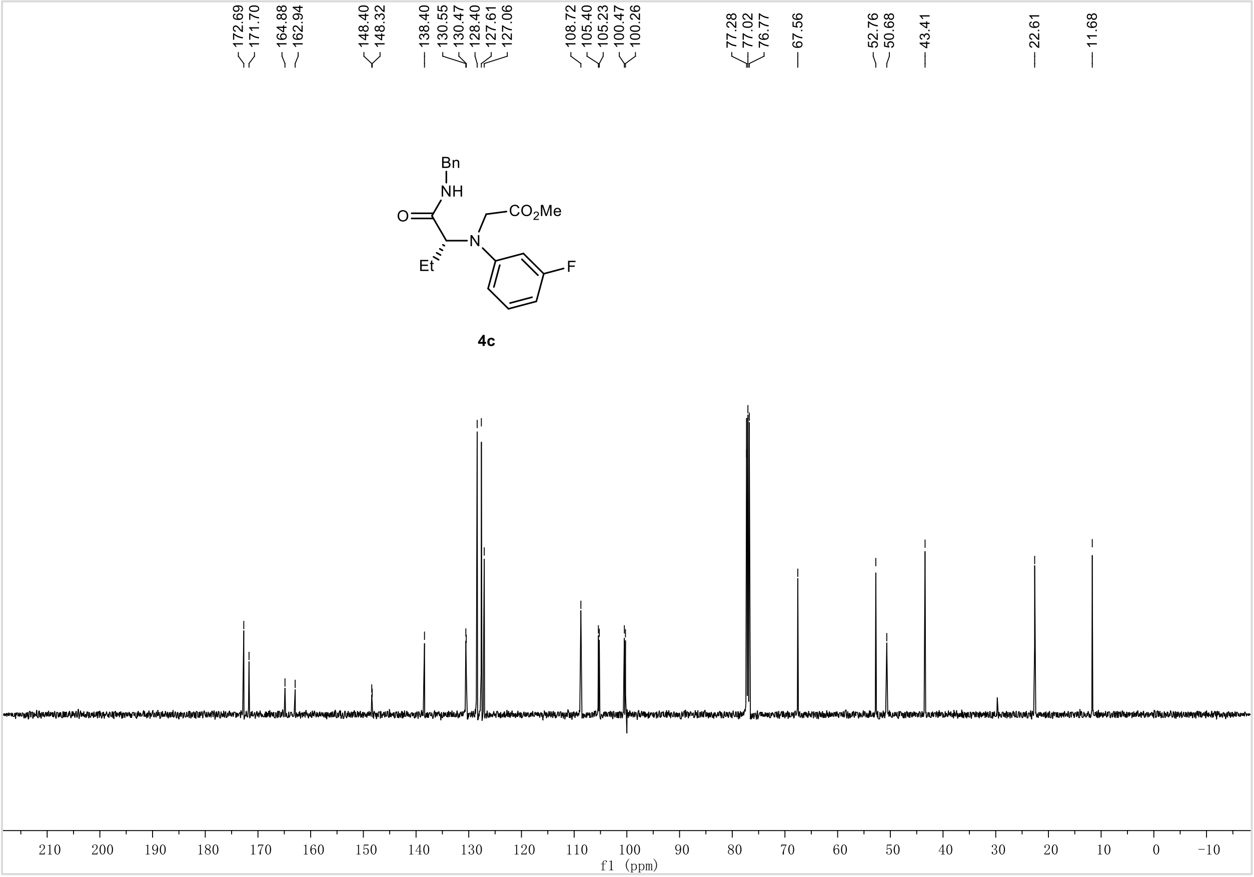

Supplementary Figure 51. ^{13}C NMR (126 MHz, $CDCl_3$) spectrum for 4c

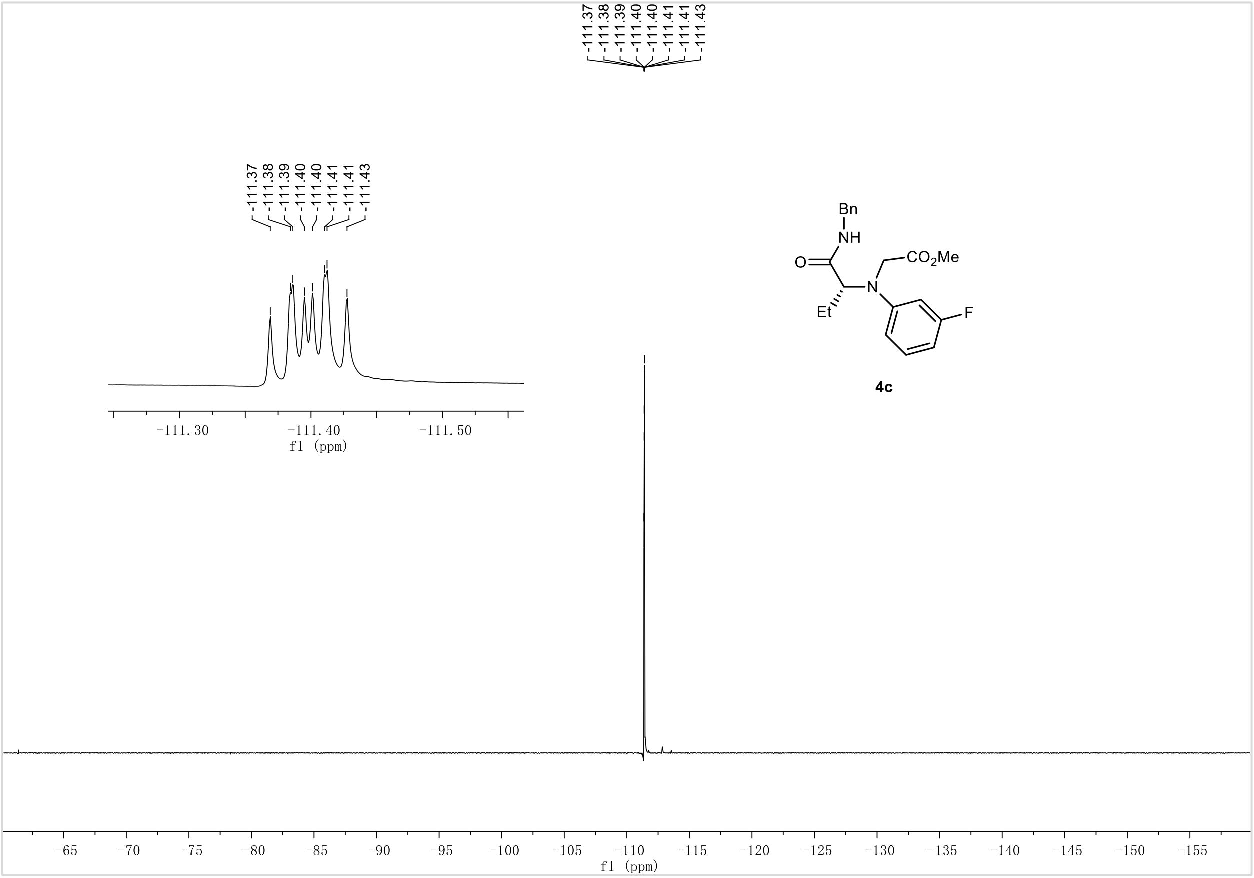

Supplementary Figure 52. ^{19}F NMR (471 MHz, $CDCl_3$) spectrum for 4c

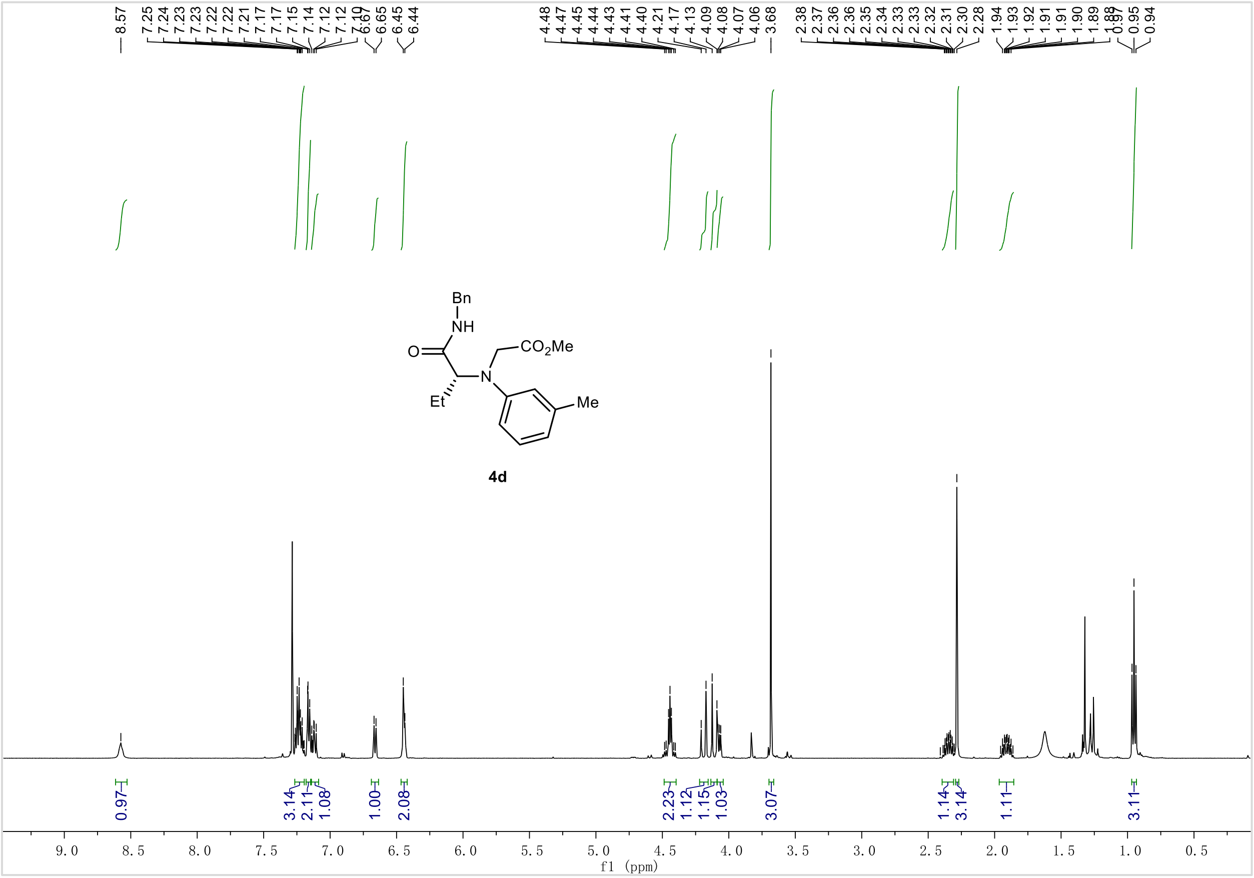

Supplementary Figure 53. ^{1}H **NMR (500 MHz,** $CDCl_3$**) spectrum for 4d**

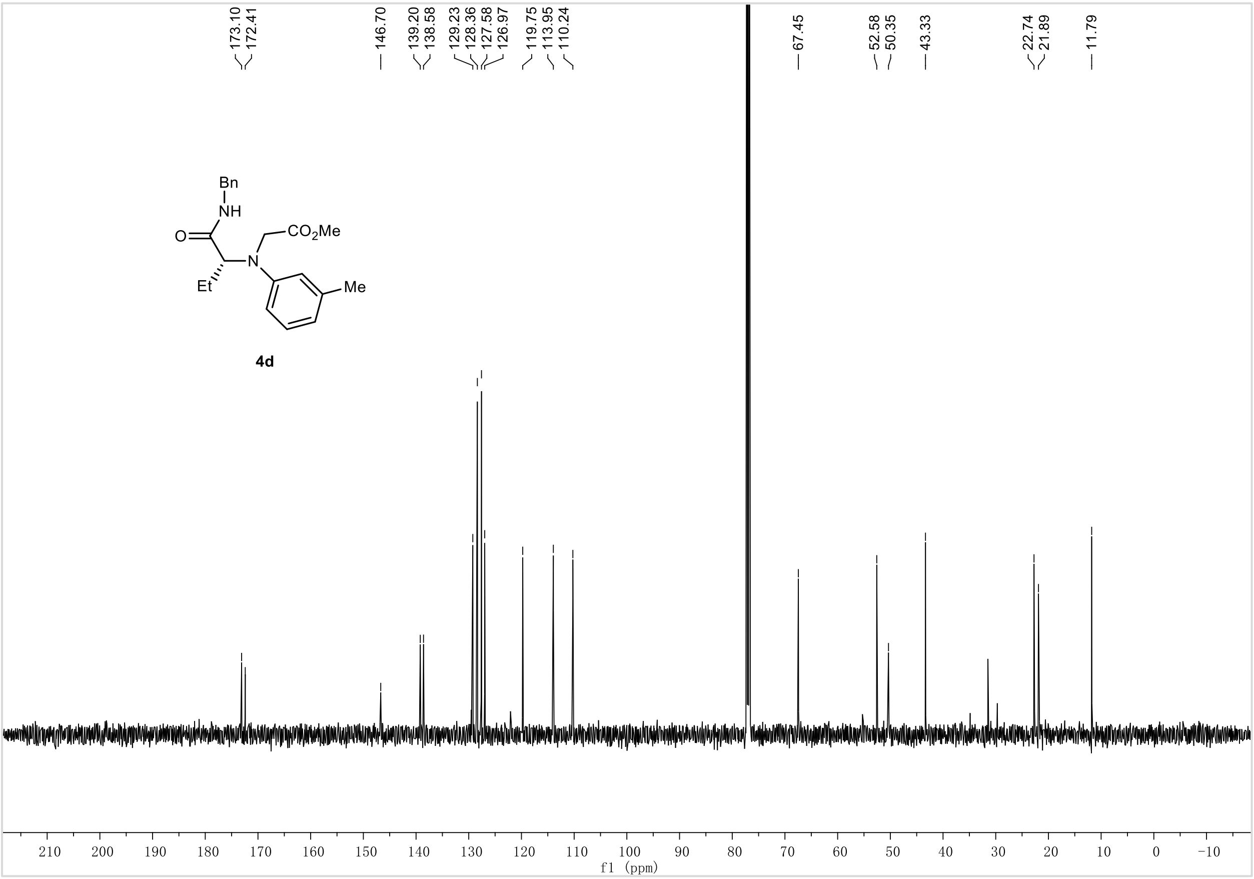

Supplementary Figure 54. ^{13}C **NMR (126 MHz,** $CDCl_3$**) spectrum for 4d**

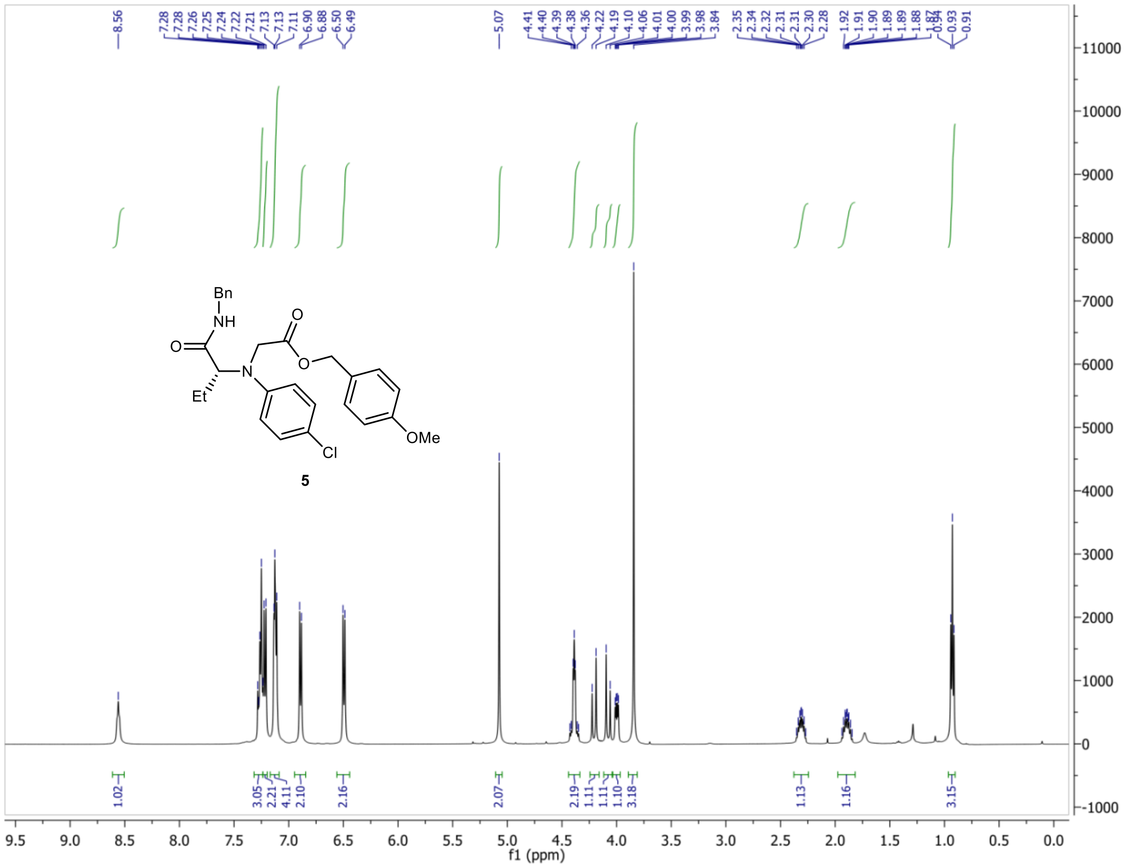

Supplementary Figure 55. ^{1}H NMR (500 MHz, $CDCl_3$) spectrum for 5

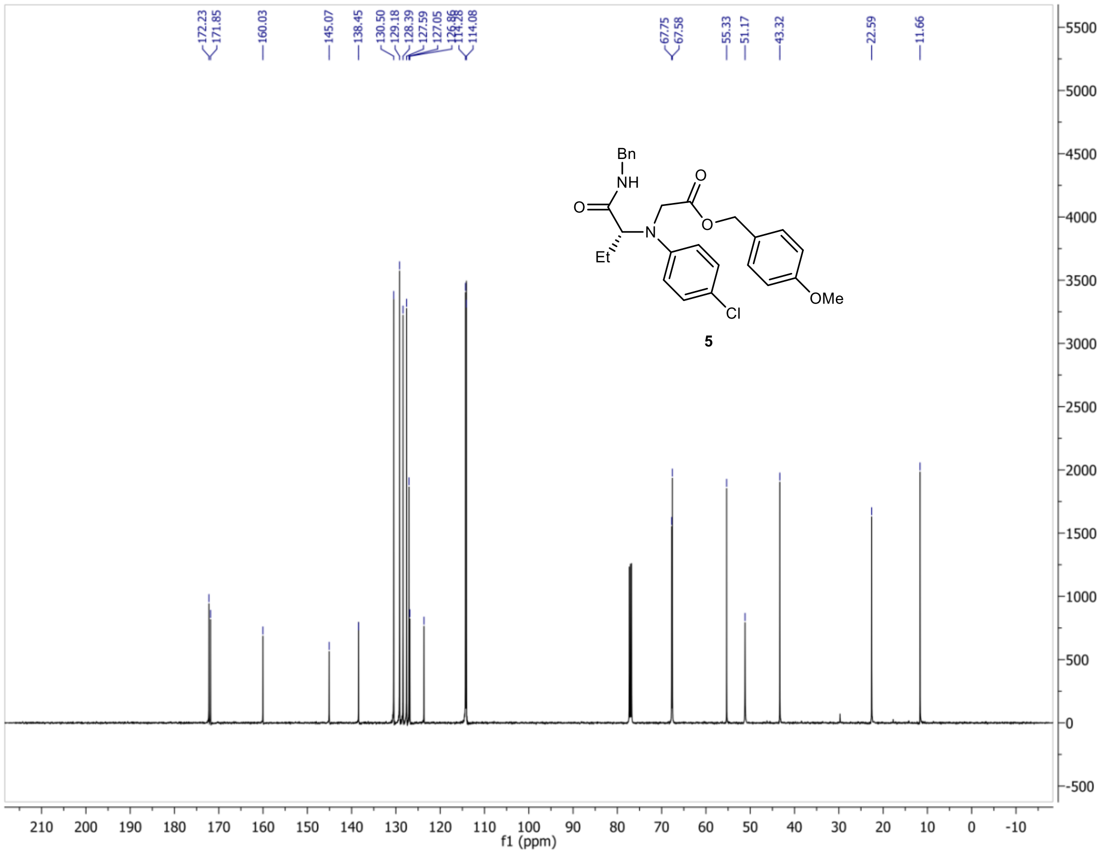

Supplementary Figure 56. ^{13}C NMR (126 MHz, $CDCl_3$) spectrum for 5

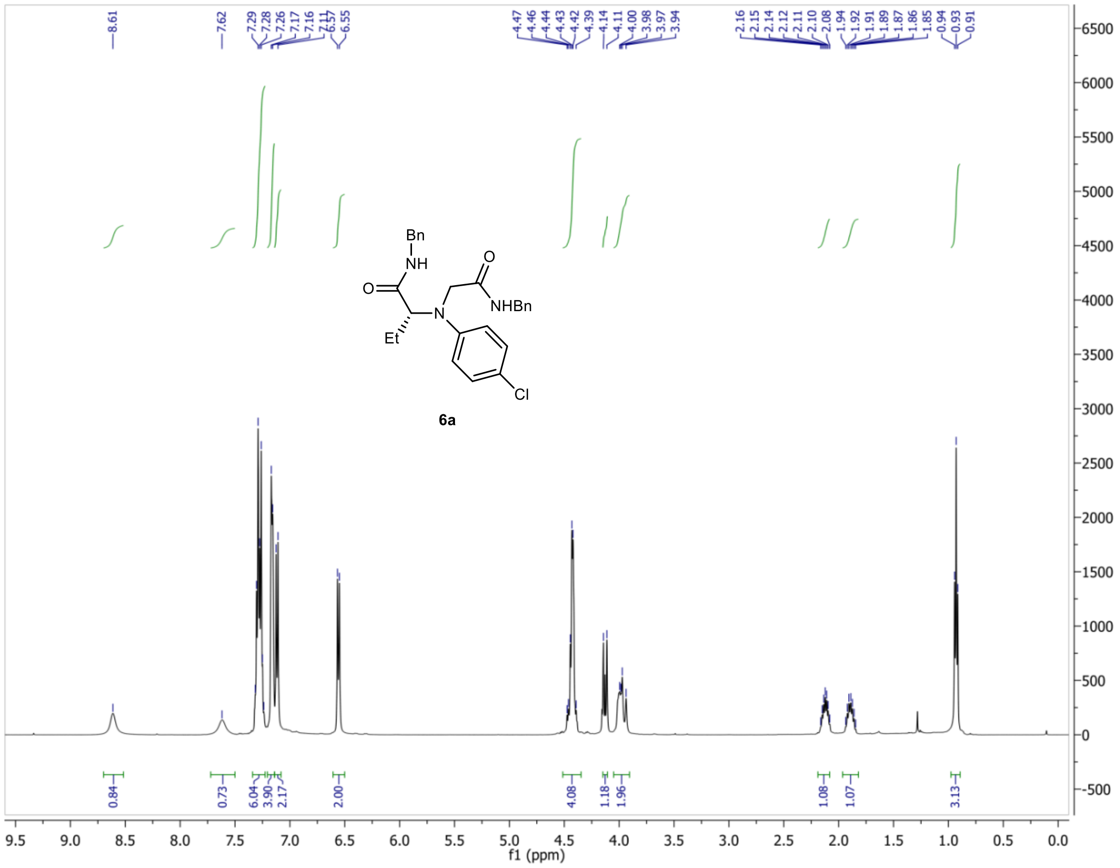

Supplementary Figure 57. ^{1}H NMR (500 MHz, $CDCl_3$) spectrum for 6a

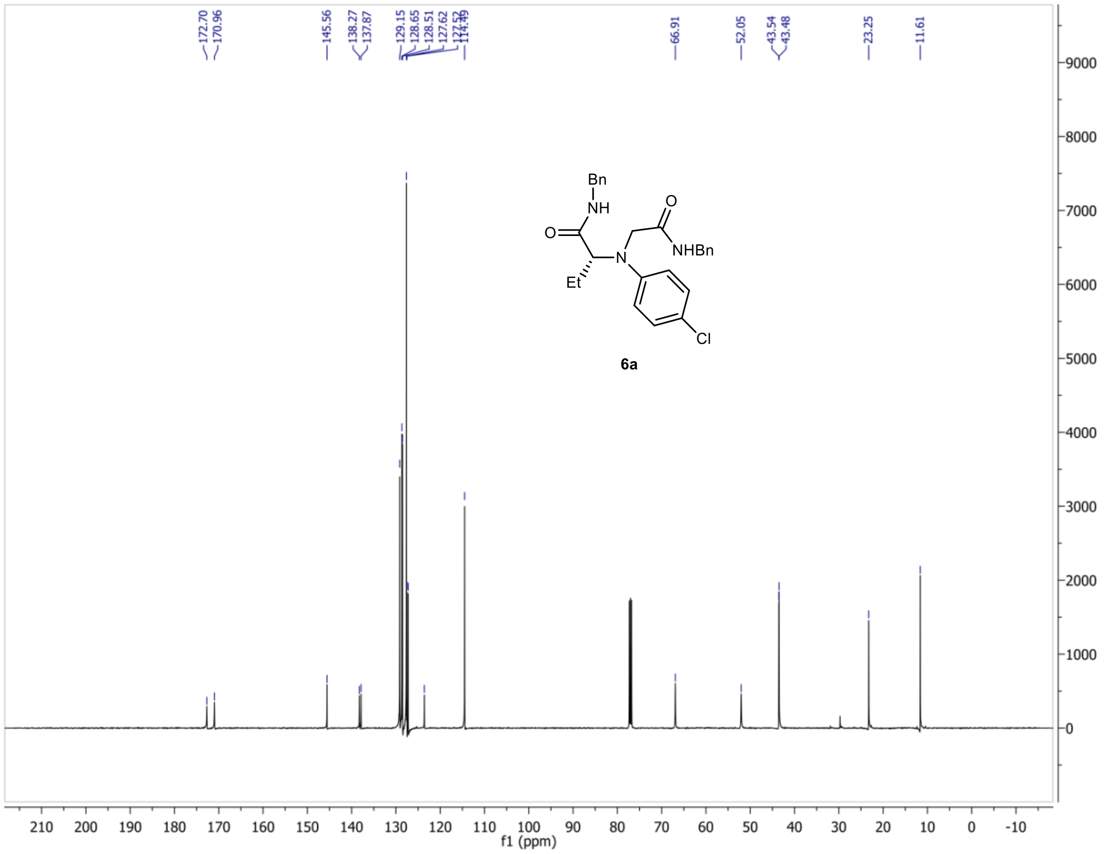

Supplementary Figure 58. ^{13}C NMR (126 MHz, $CDCl_3$) spectrum for 6a

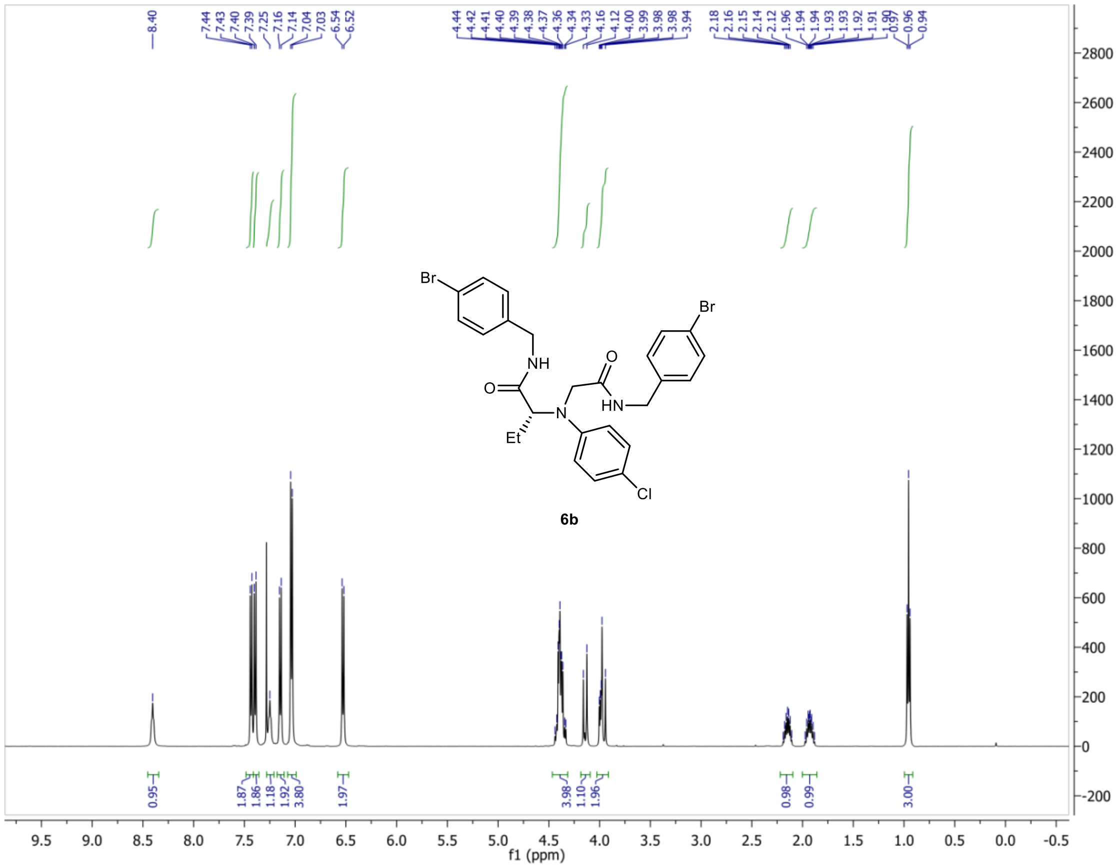

Supplementary Figure 59. ^{1}H NMR (500 MHz, $CDCl_3$) spectrum for 6b

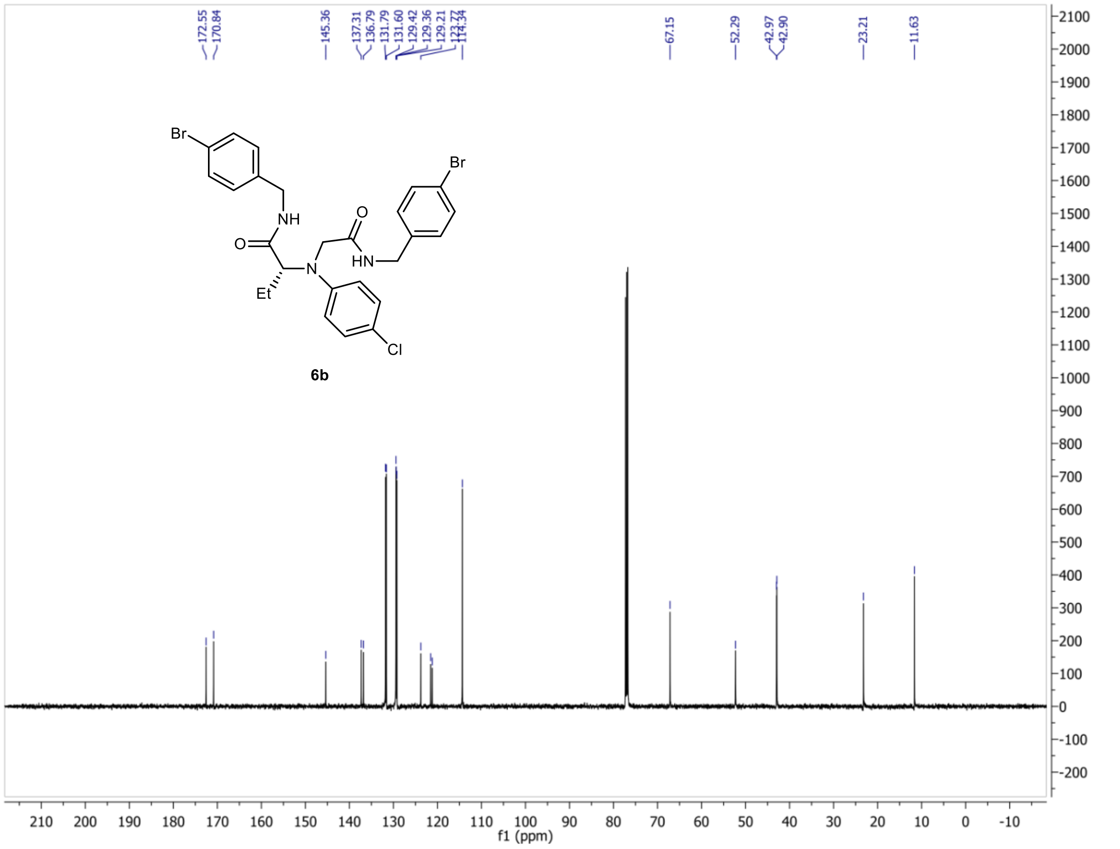

Supplementary Figure 60. ^{13}C NMR (126 MHz, $CDCl_3$) spectrum for 6b

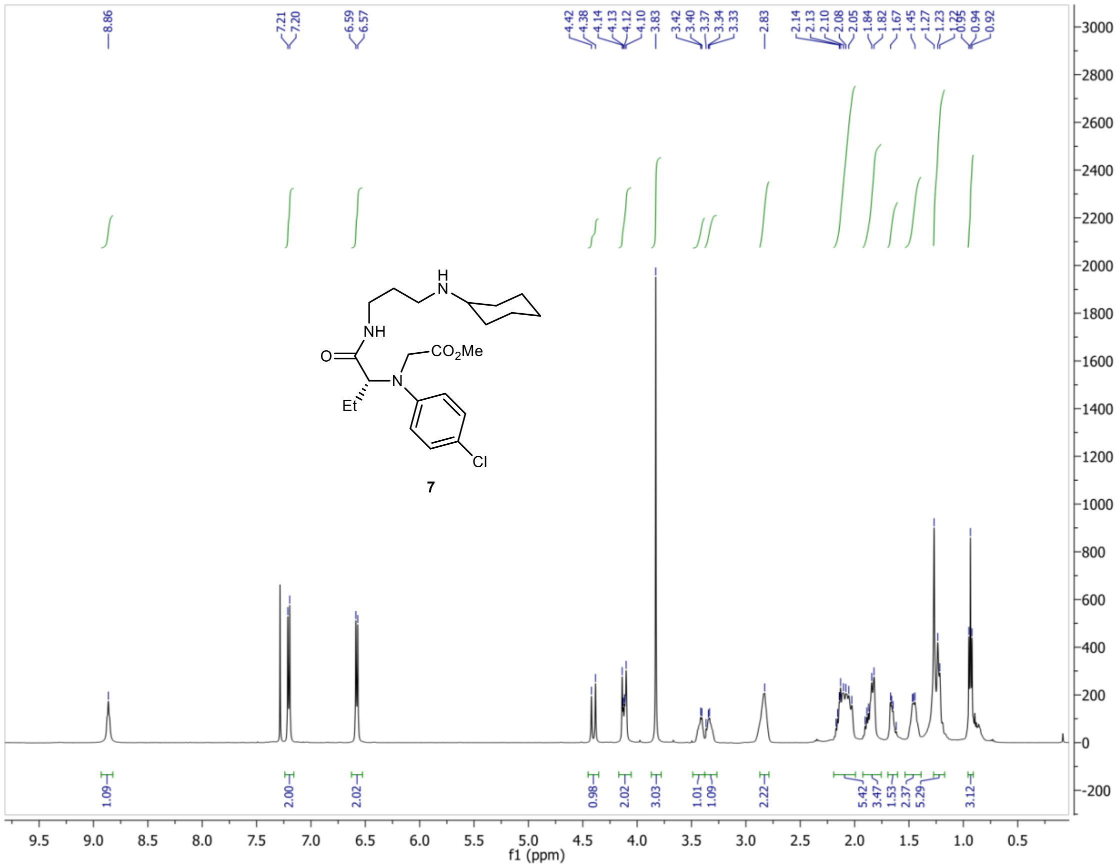

Supplementary Figure 61. ^{1}H NMR (500 MHz, $CDCl_3$) spectrum for 7

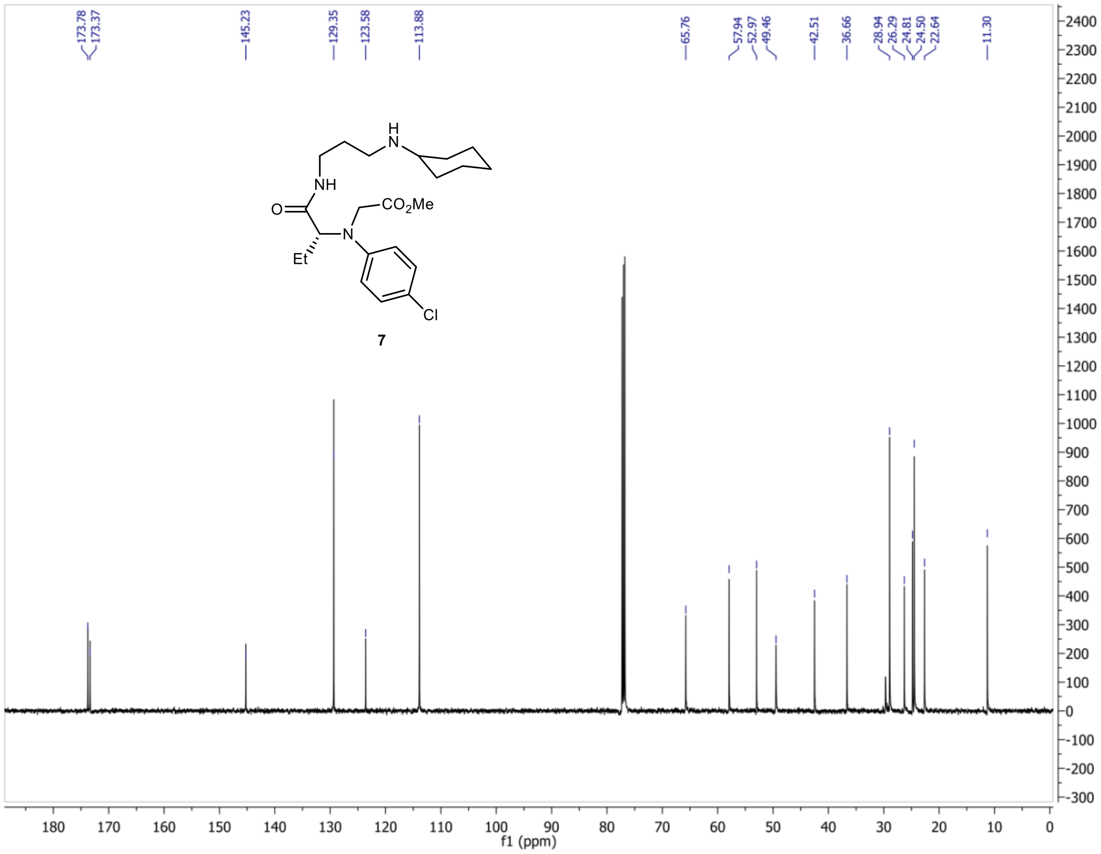

Supplementary Figure 62. ^{13}C NMR (126 MHz, $CDCl_3$) spectrum for 7

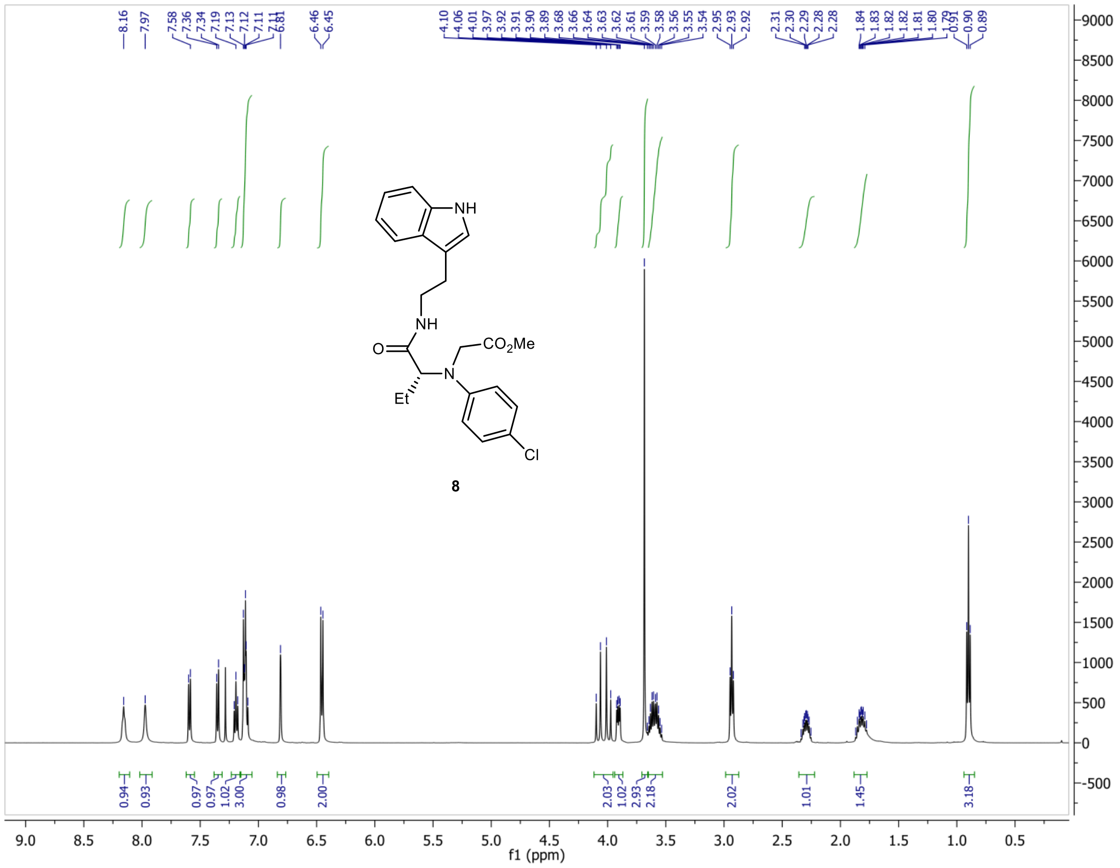

Supplementary Figure 63. ^{1}H NMR (500 MHz, $CDCl_3$) spectrum for 8

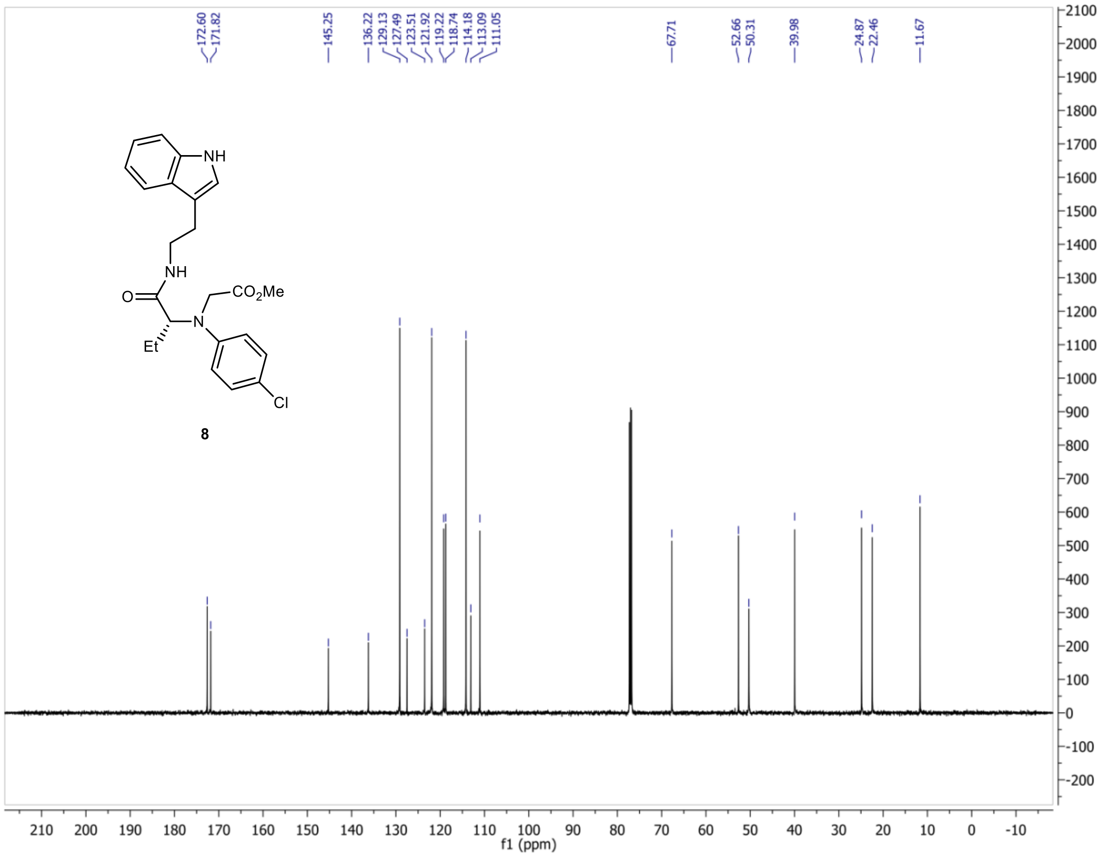

Supplementary Figure 64. ^{13}C NMR (126 MHz, $CDCl_3$) spectrum for 8

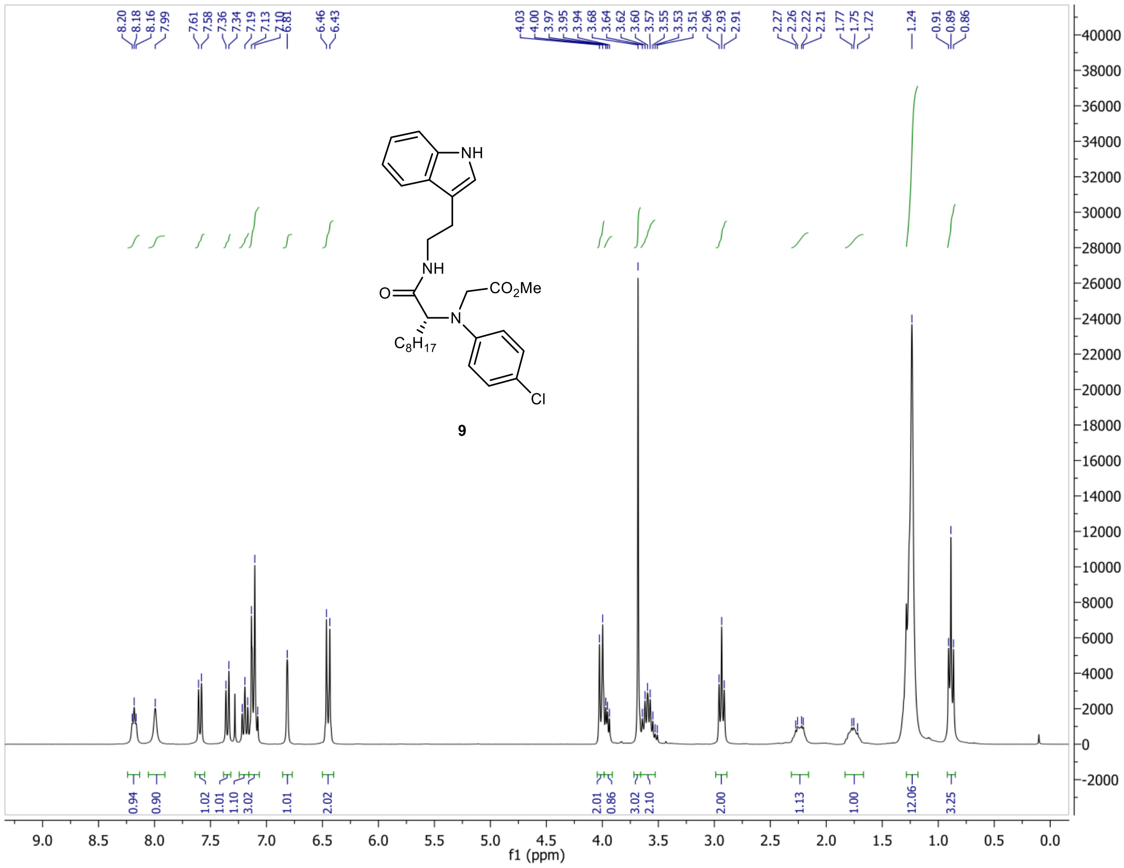

Supplementary Figure 65. ^{1}H NMR (300 MHz, $CDCl_3$) spectrum for 9

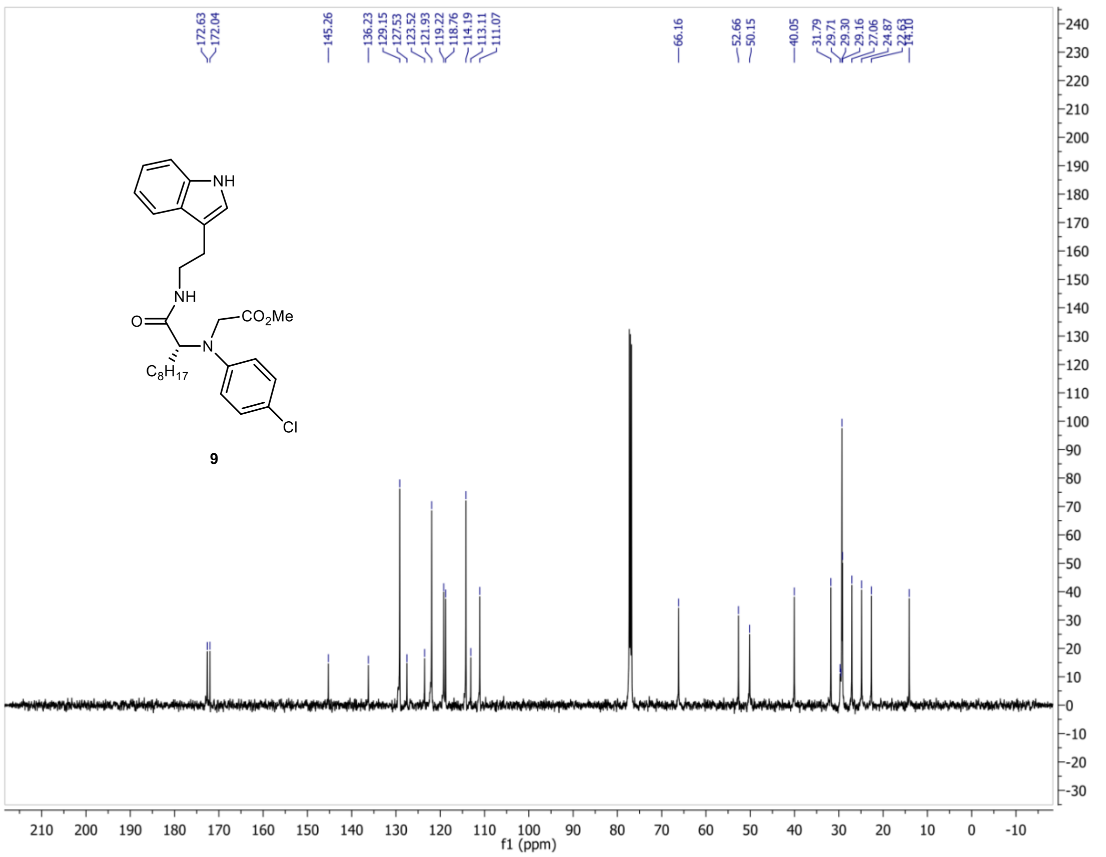

Supplementary Figure 66. ^{13}C NMR (126 MHz, $CDCl_3$) spectrum for 9

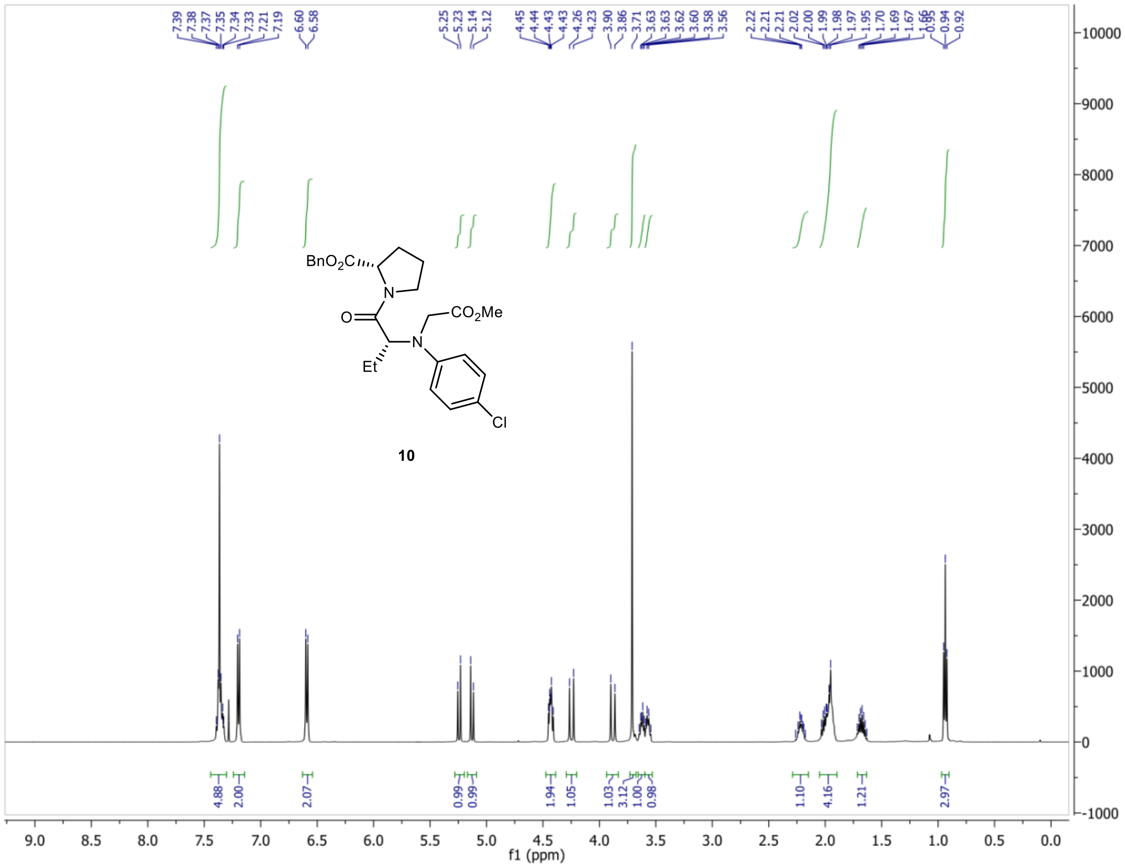

Supplementary Figure 67. ^{1}H NMR (500 MHz, $CDCl_3$) spectrum for 10

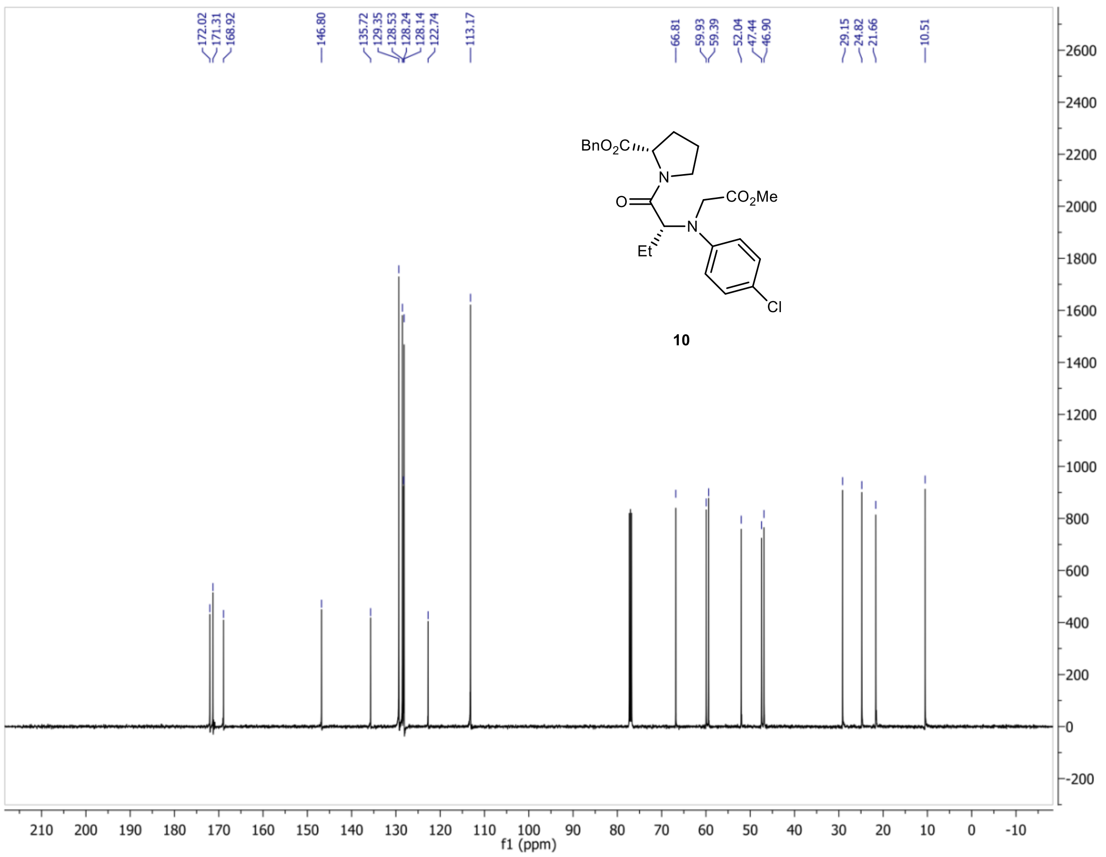

Supplementary Figure 68. ^{13}C NMR (126 MHz, $CDCl_3$) spectrum for 10

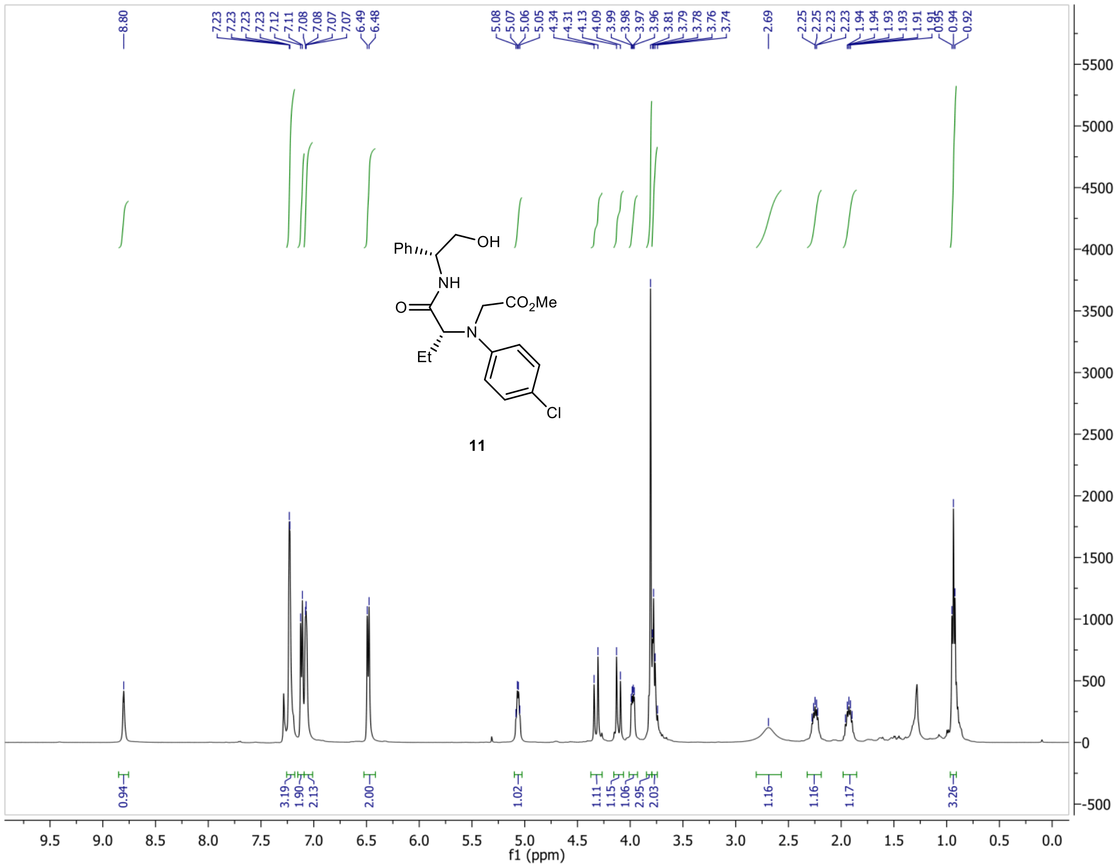

Supplementary Figure 69. ^{1}H NMR (500 MHz, $CDCl_3$) spectrum for 11

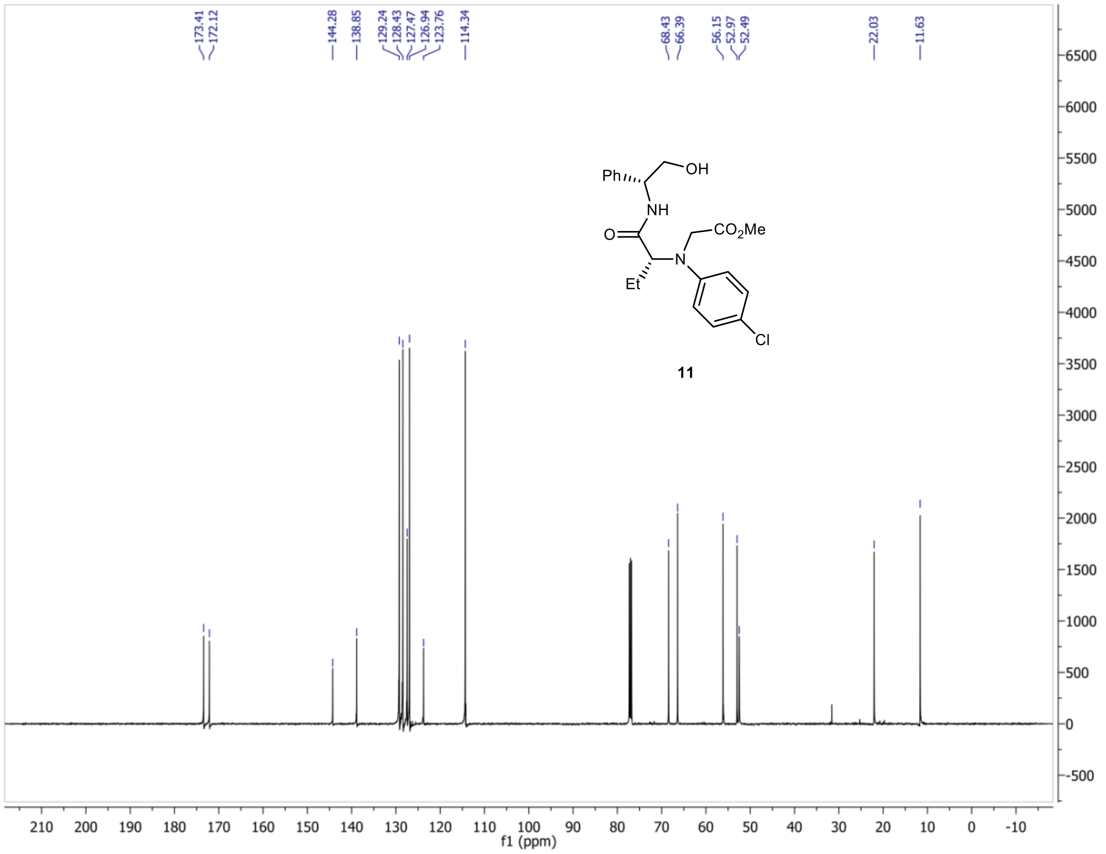

Supplementary Figure 70. ^{13}C NMR (126 MHz, $CDCl_3$) spectrum for 11

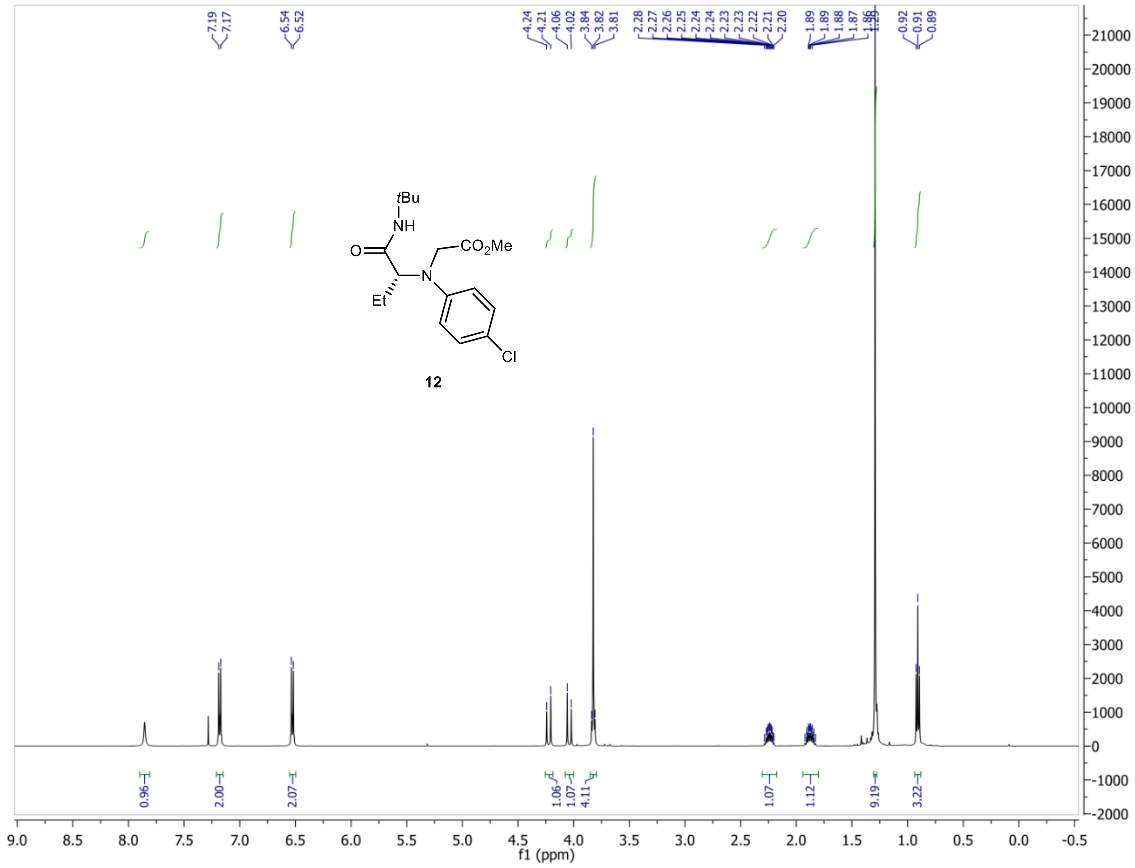

Supplementary Figure 71. ^{1}H NMR (500 MHz, $CDCl_3$) spectrum for 12

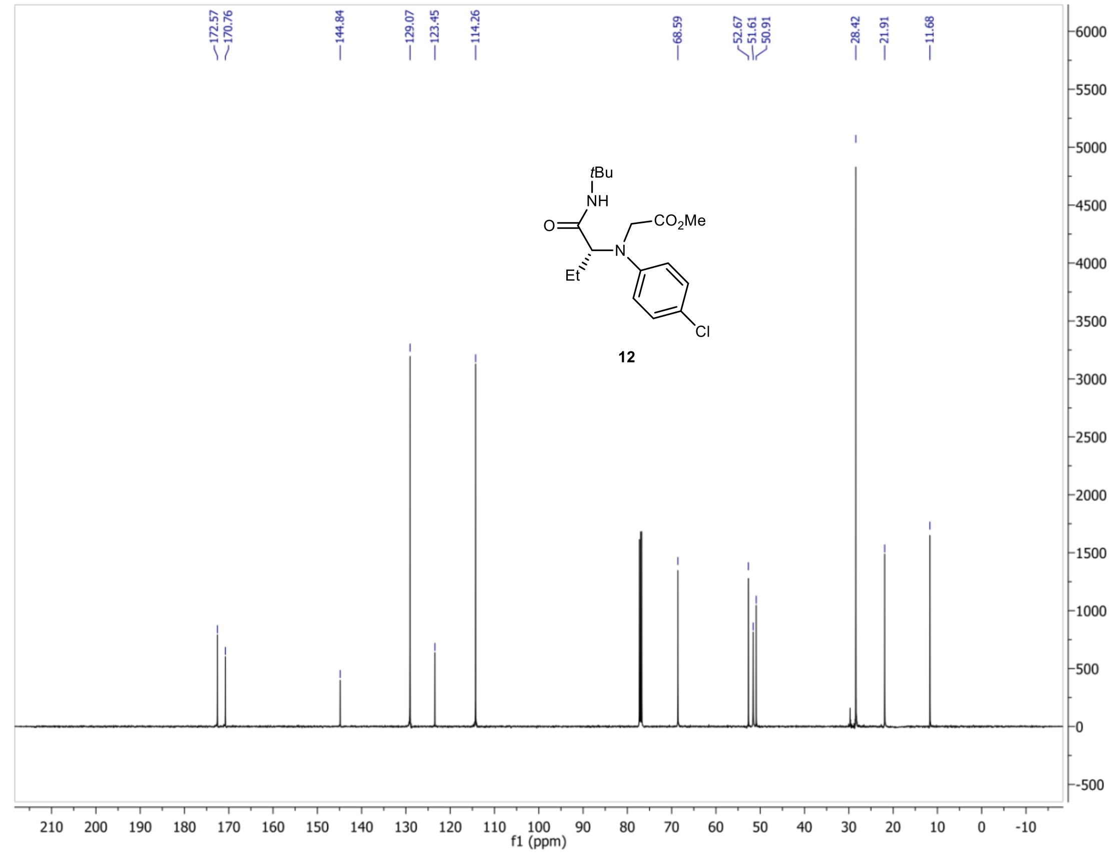

Supplementary Figure 72. ^{13}C NMR (126 MHz, $CDCl_3$) spectrum for 12

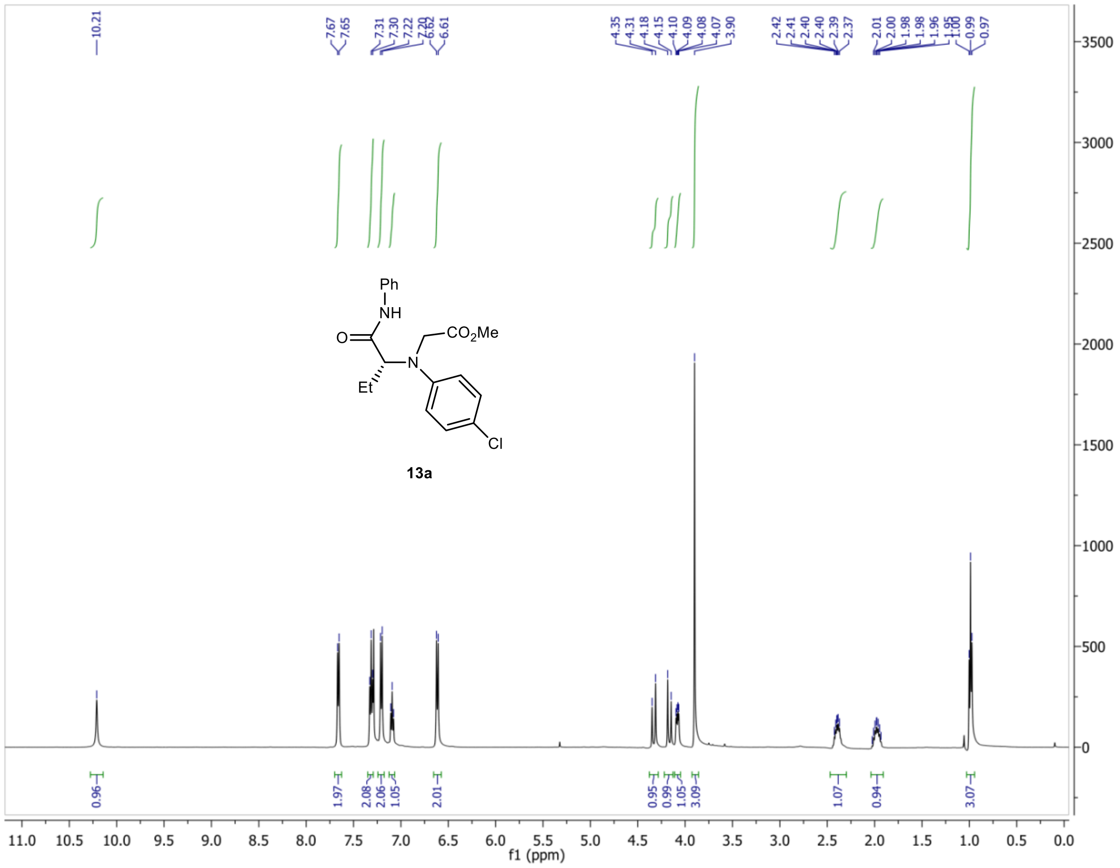

Supplementary Figure 73. ^{1}H NMR (500 MHz, CDCl$_3$) spectrum for 13a

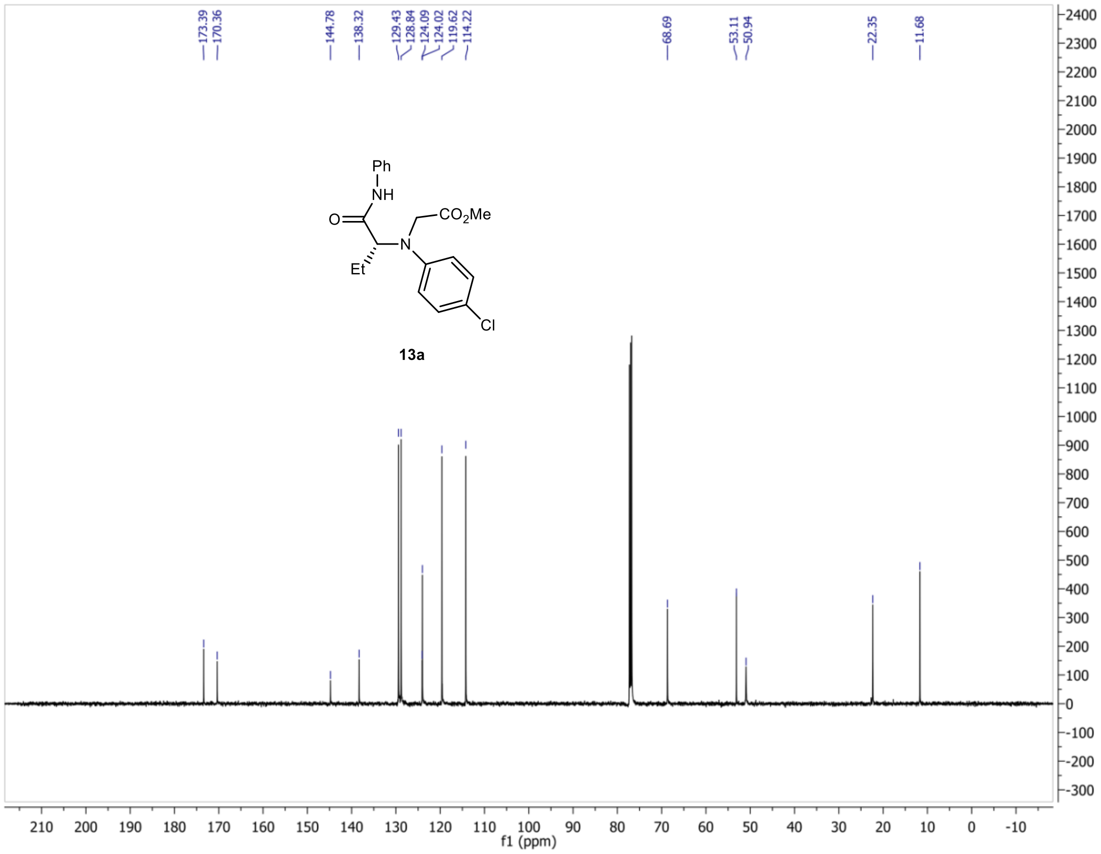

Supplementary Figure 74. ^{13}C NMR (126 MHz, CDCl$_3$) spectrum for 13a

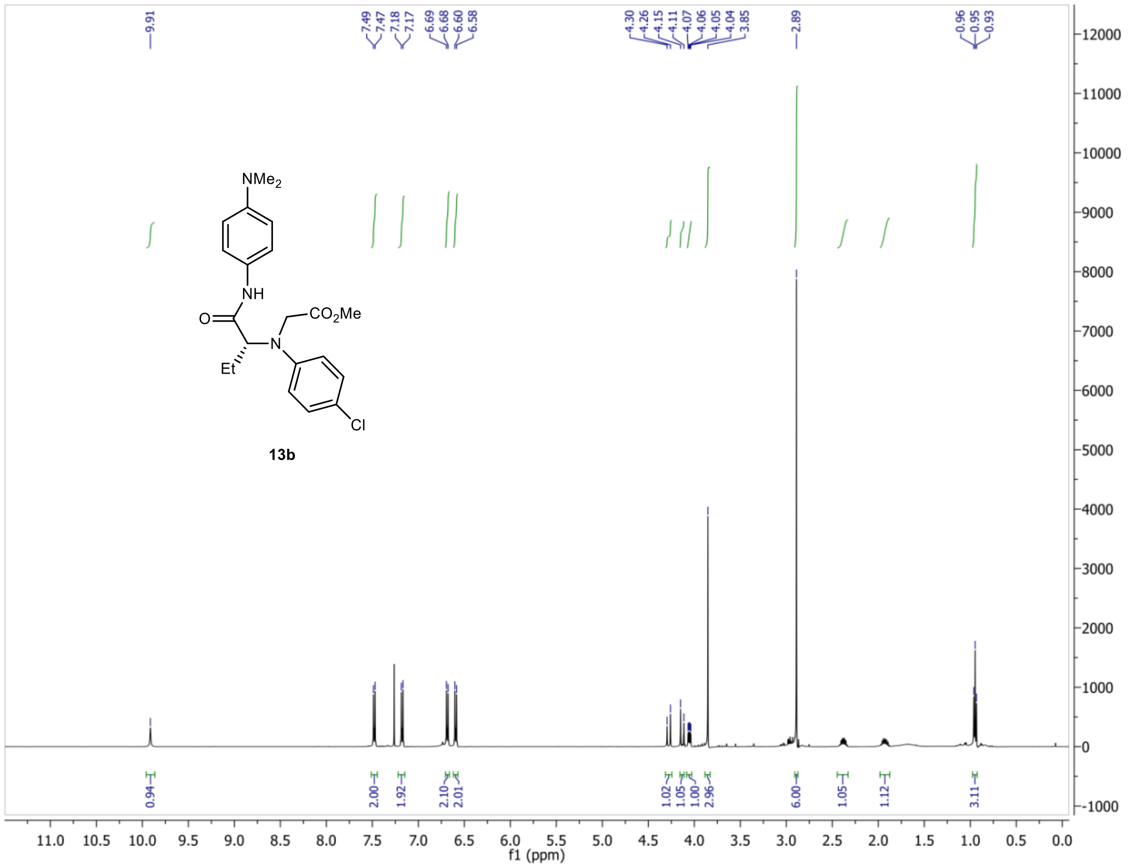

Supplementary Figure 75. ^{1}H NMR (500 MHz, $CDCl_3$) spectrum for 13b

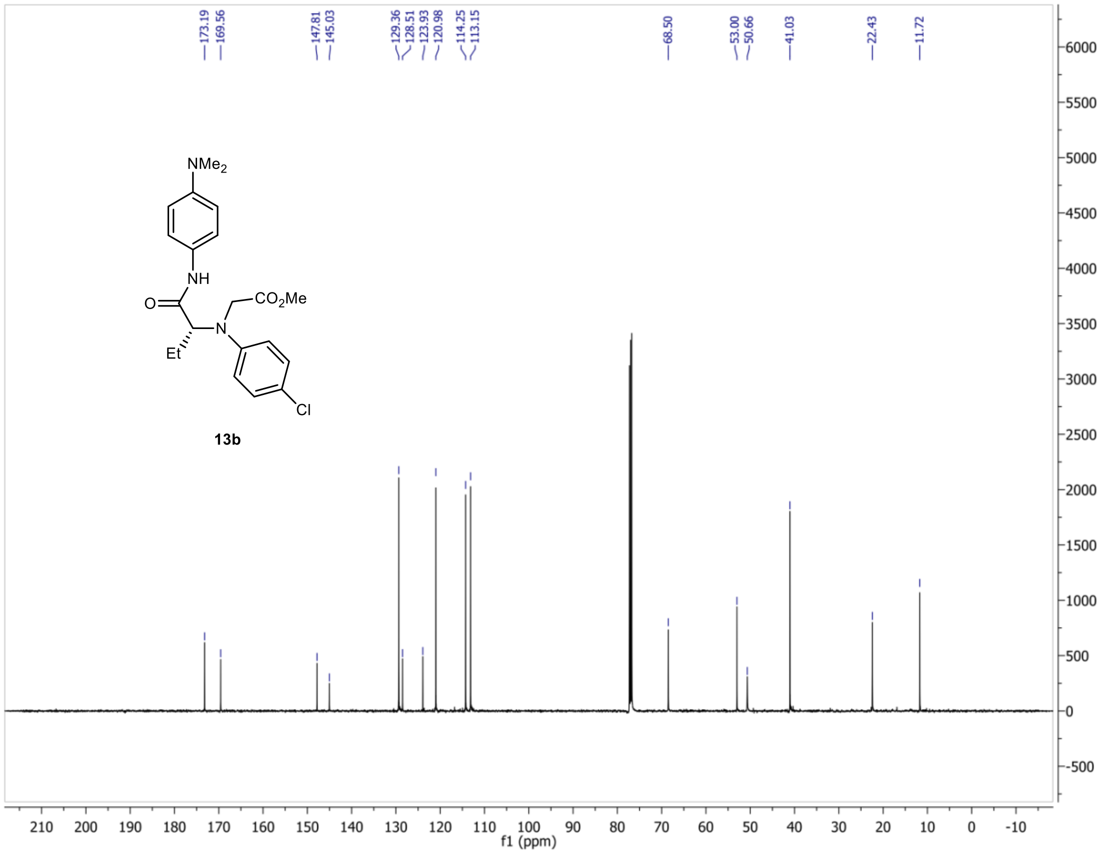

Supplementary Figure 76. ^{13}C NMR (126 MHz, $CDCl_3$) spectrum for 13b

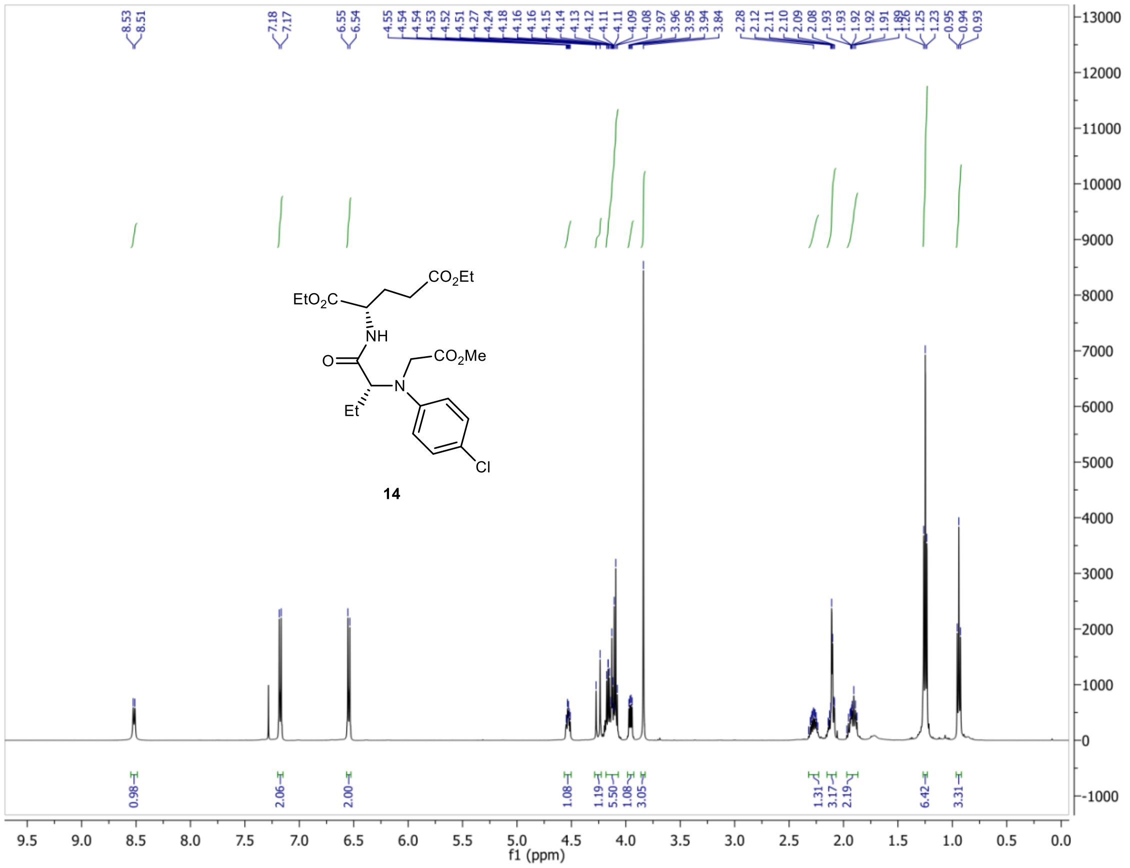

Supplementary Figure 77. ^{1}H NMR (500 MHz, CDCl$_3$) spectrum for 14

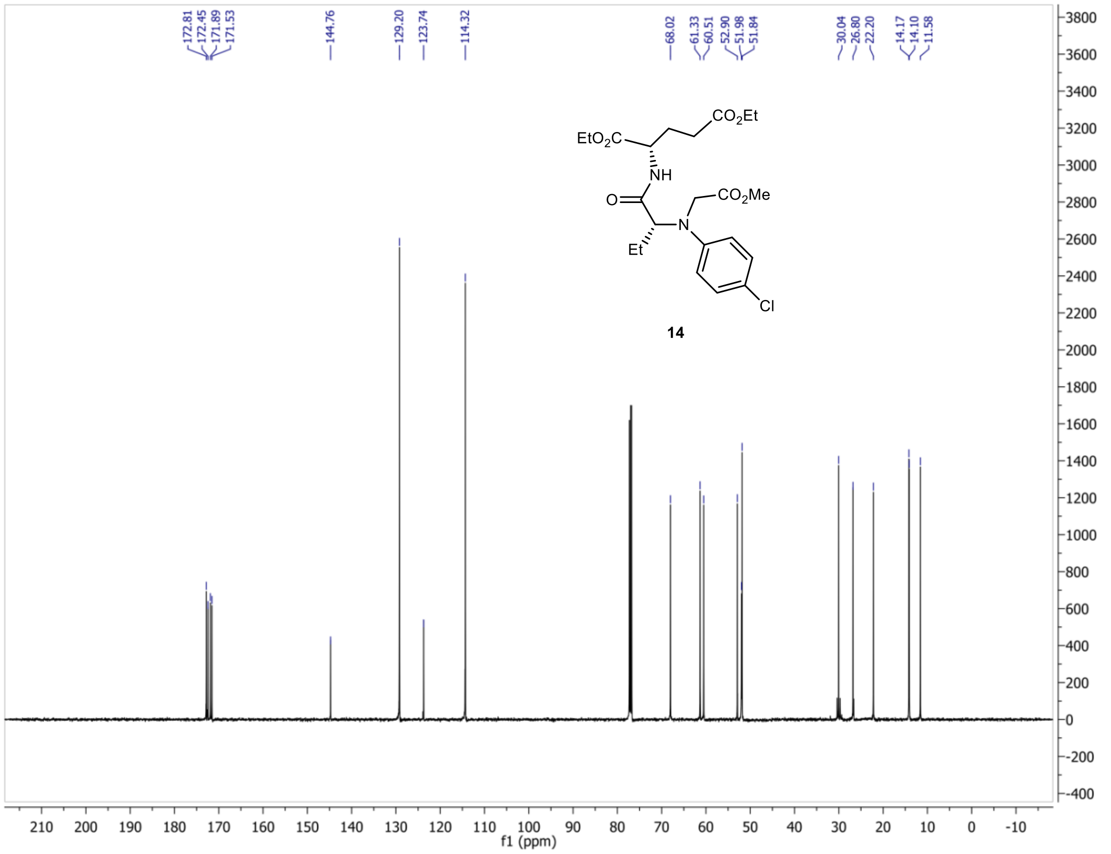

Supplementary Figure 78. ^{13}C NMR (126 MHz, CDCl$_3$) spectrum for 14

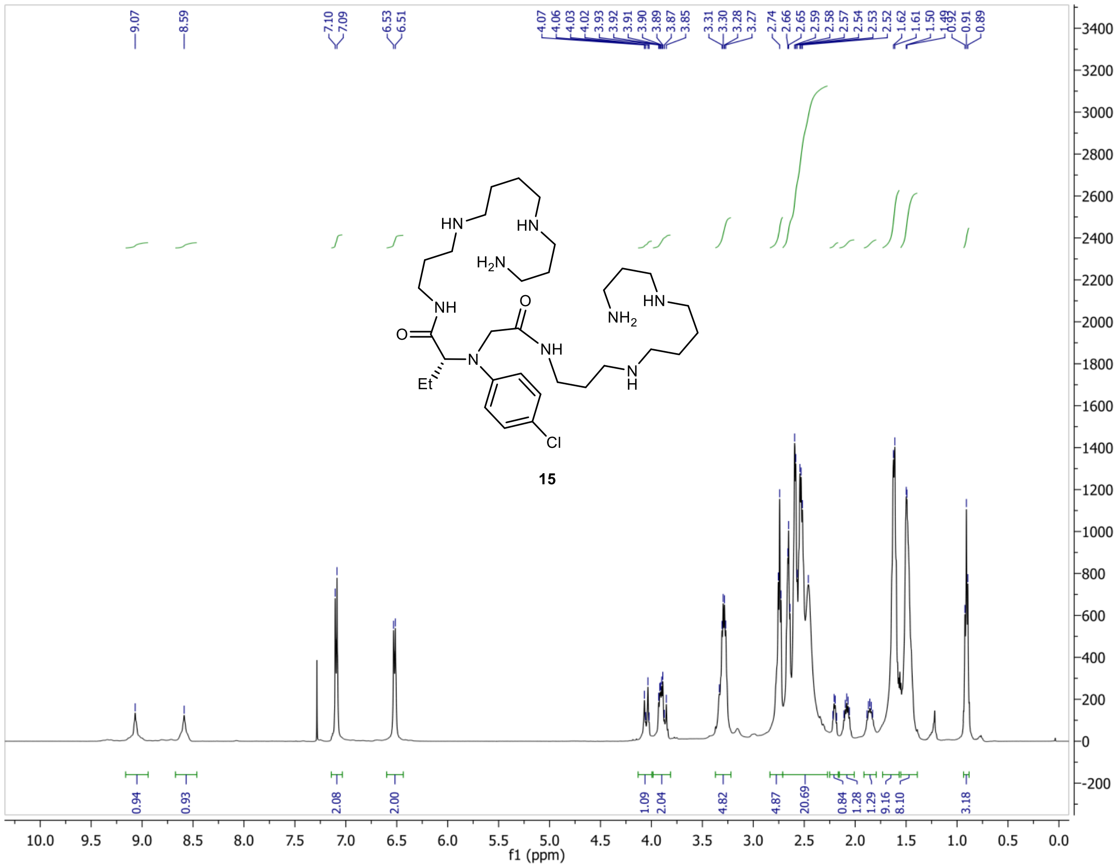

Supplementary Figure 79. ^{1}H NMR (500 MHz, $CDCl_3$) spectrum for 15

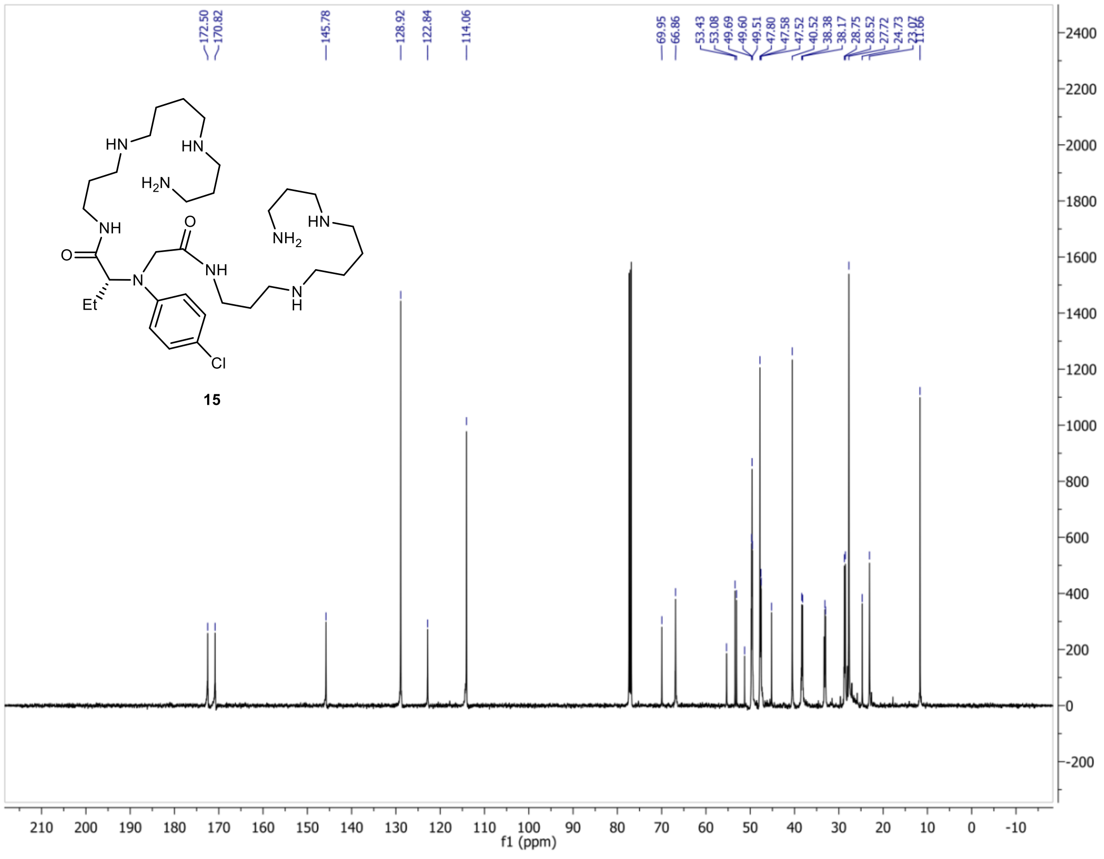

Supplementary Figure 80. ^{13}C NMR (126 MHz, $CDCl_3$) spectrum for 15

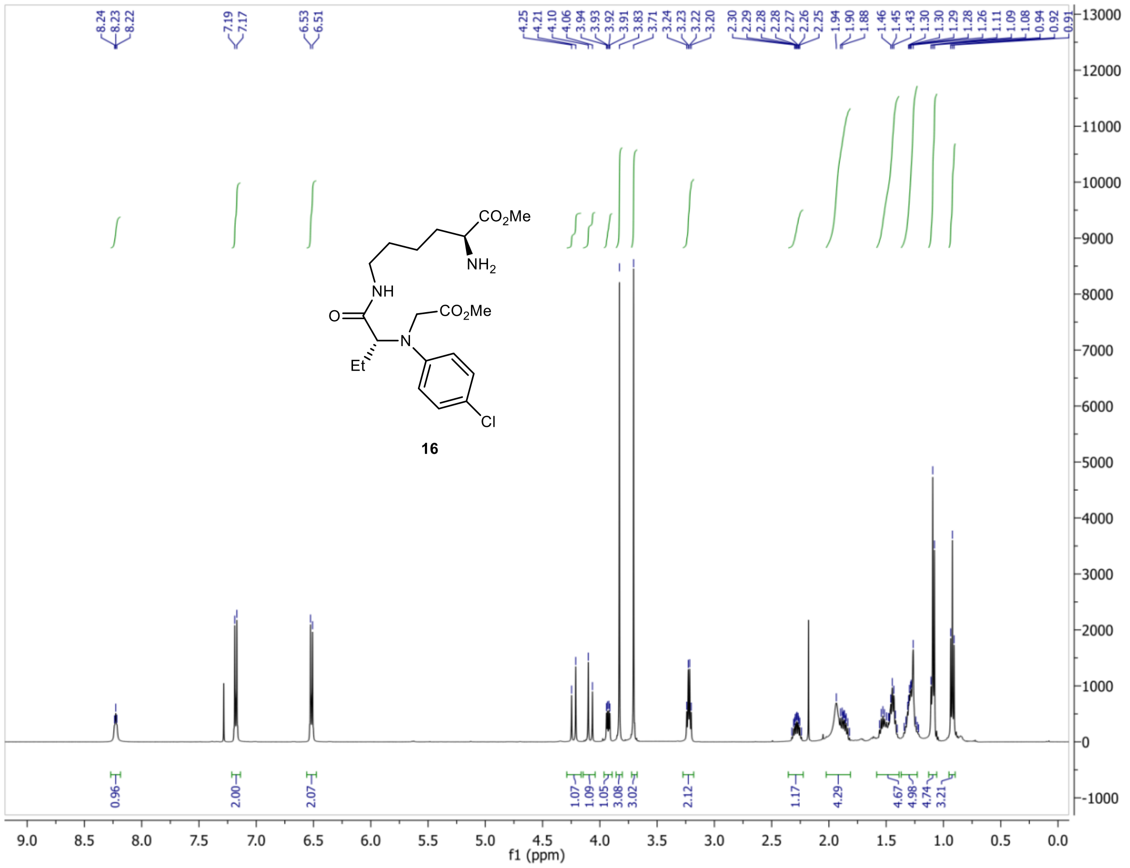

Supplementary Figure 81. ^{1}H NMR (500 MHz, $CDCl_3$) spectrum for 16

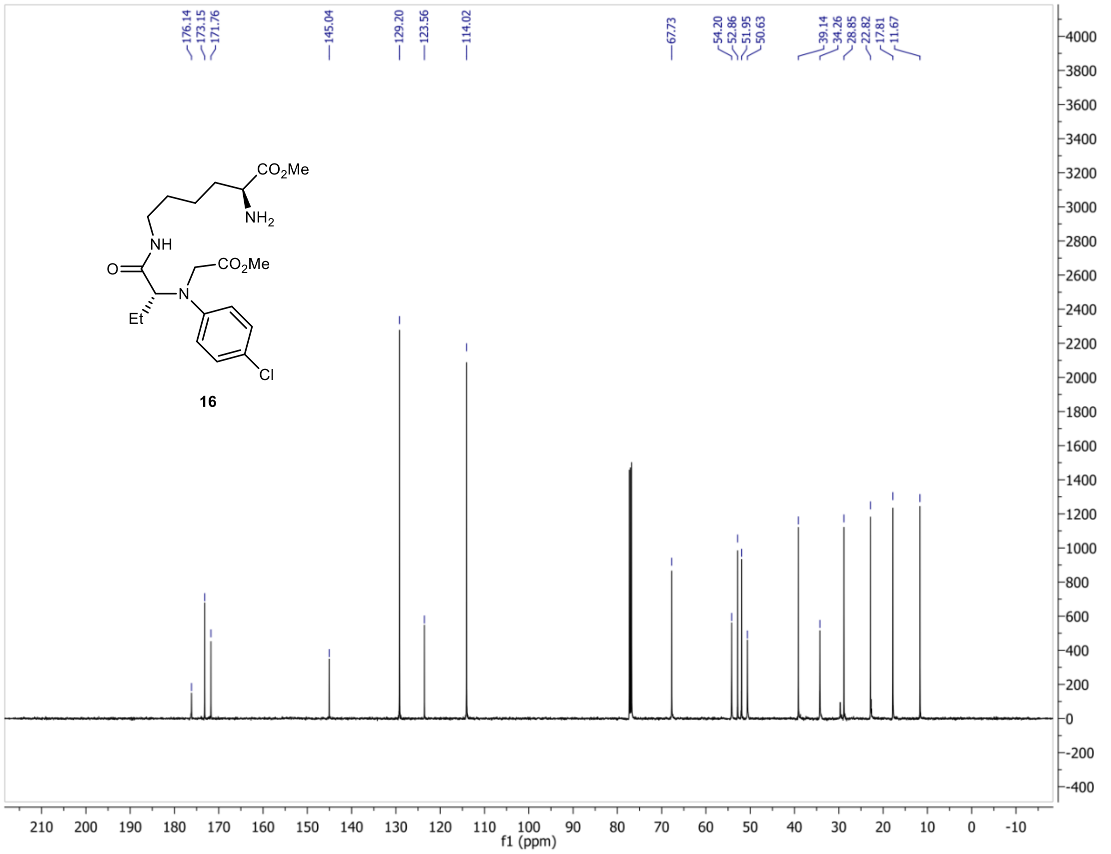

Supplementary Figure 82. ^{13}C NMR (126 MHz, $CDCl_3$) spectrum for 16

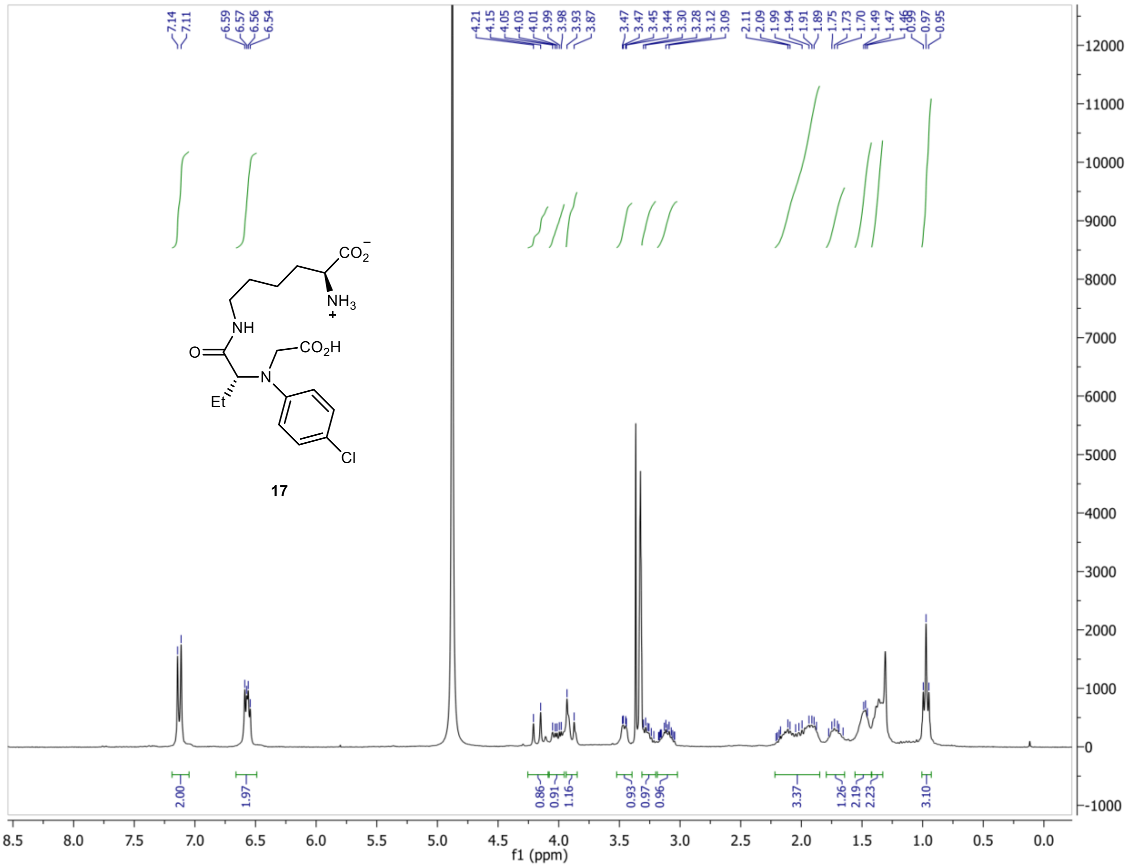

Supplementary Figure 83. ^{1}H NMR (300 MHz, CD_3OD) spectrum for 17

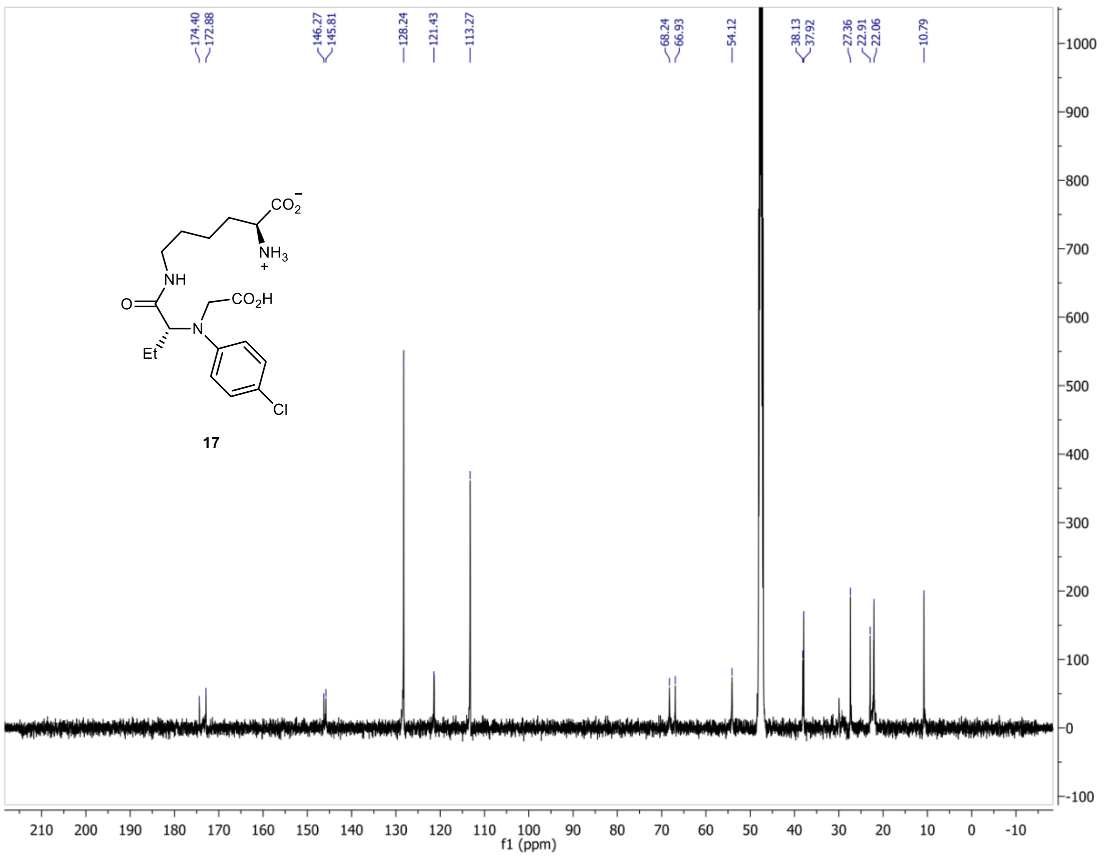

Supplementary Figure 84. ^{13}C NMR (126 MHz, CD_3OD) spectrum for 17

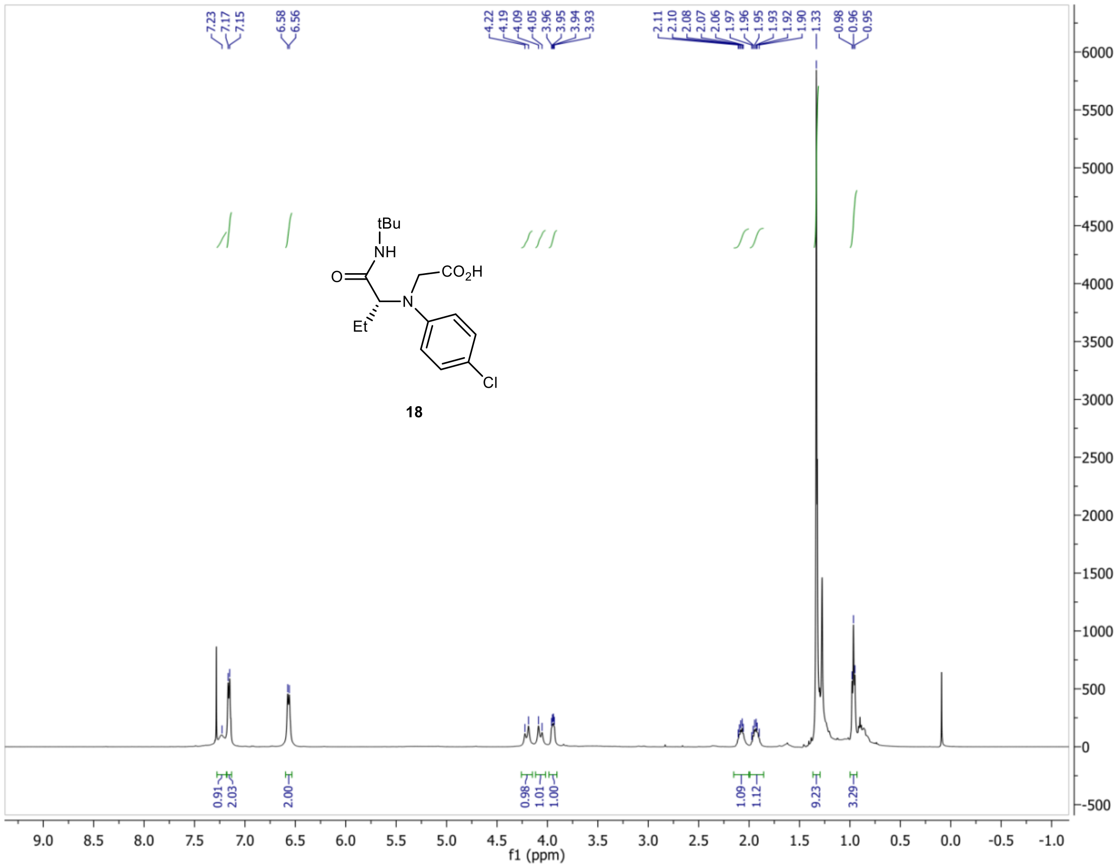

Supplementary Figure 85. ^{1}H NMR (500 MHz, $CDCl_3$) spectrum for 18

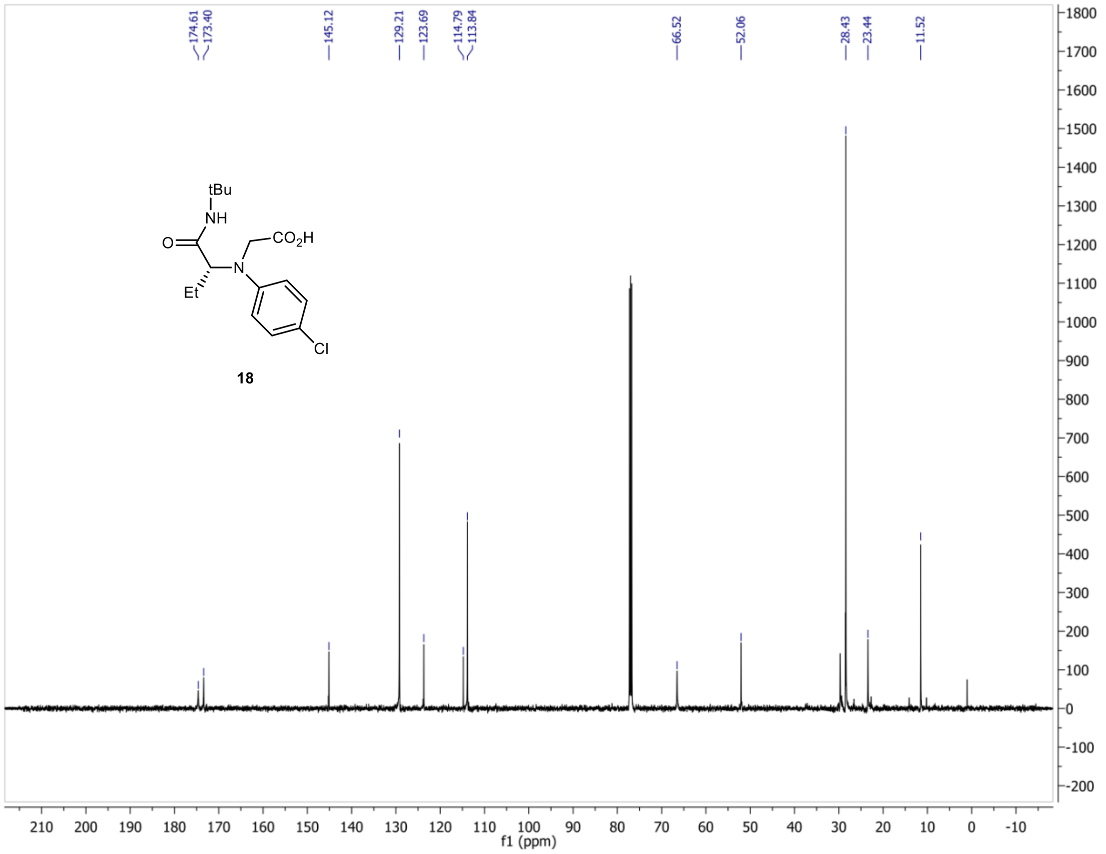

Supplementary Figure 86. ^{13}C NMR (126 MHz, $CDCl_3$) spectrum for 18

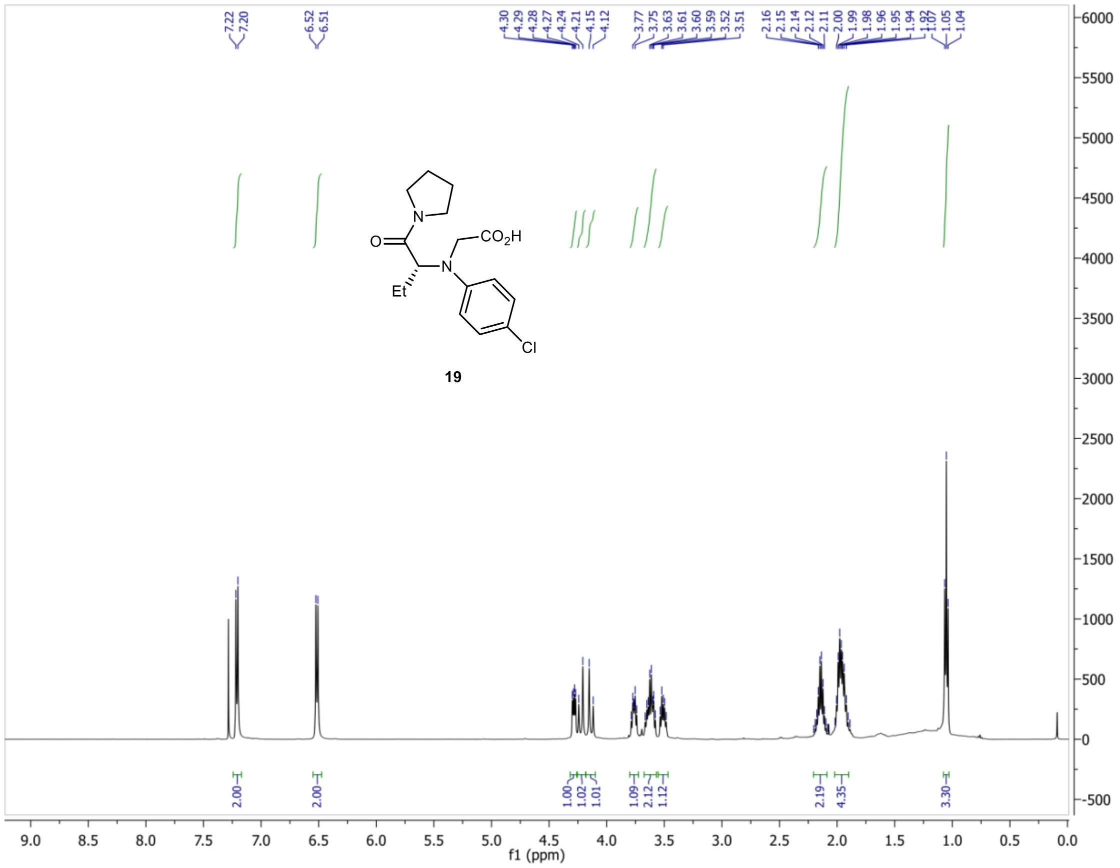

Supplementary Figure 87. ^{1}**H NMR (500 MHz,** $CDCl_3$**) spectrum for 19**

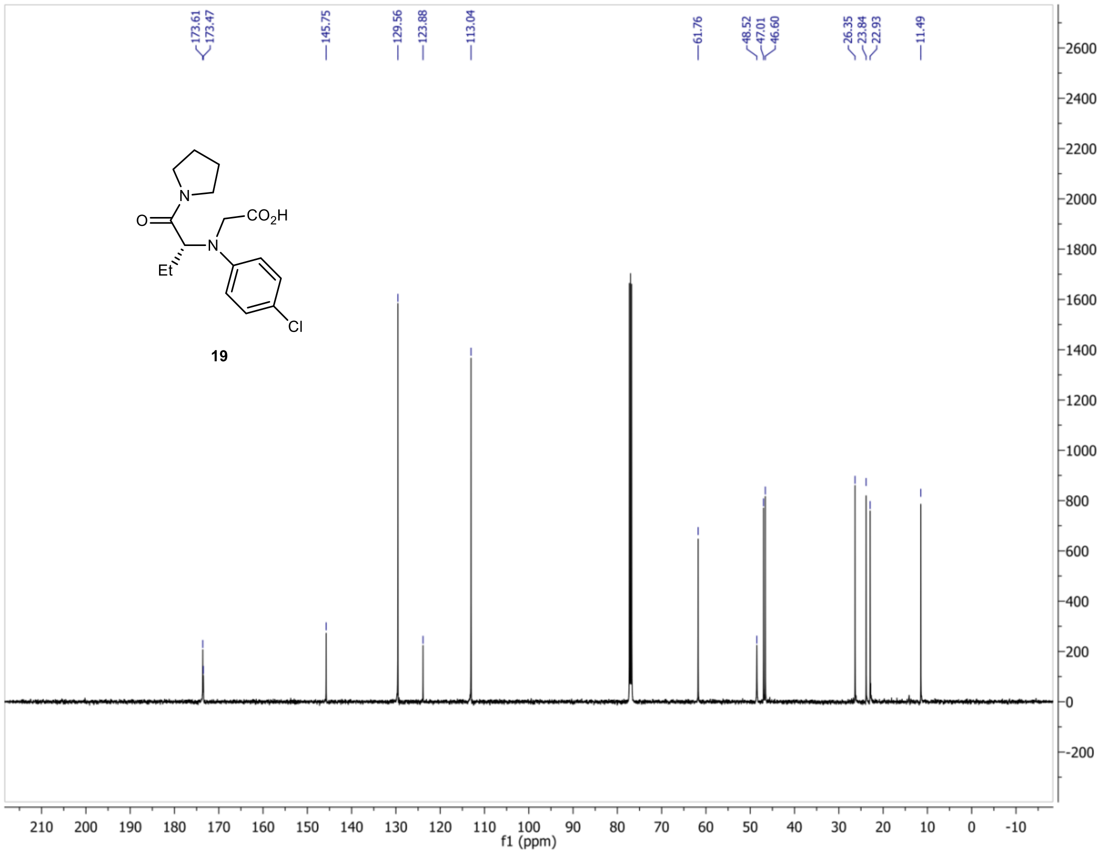

Supplementary Figure 88. ^{13}**C NMR (126 MHz,** $CDCl_3$**) spectrum for 19**

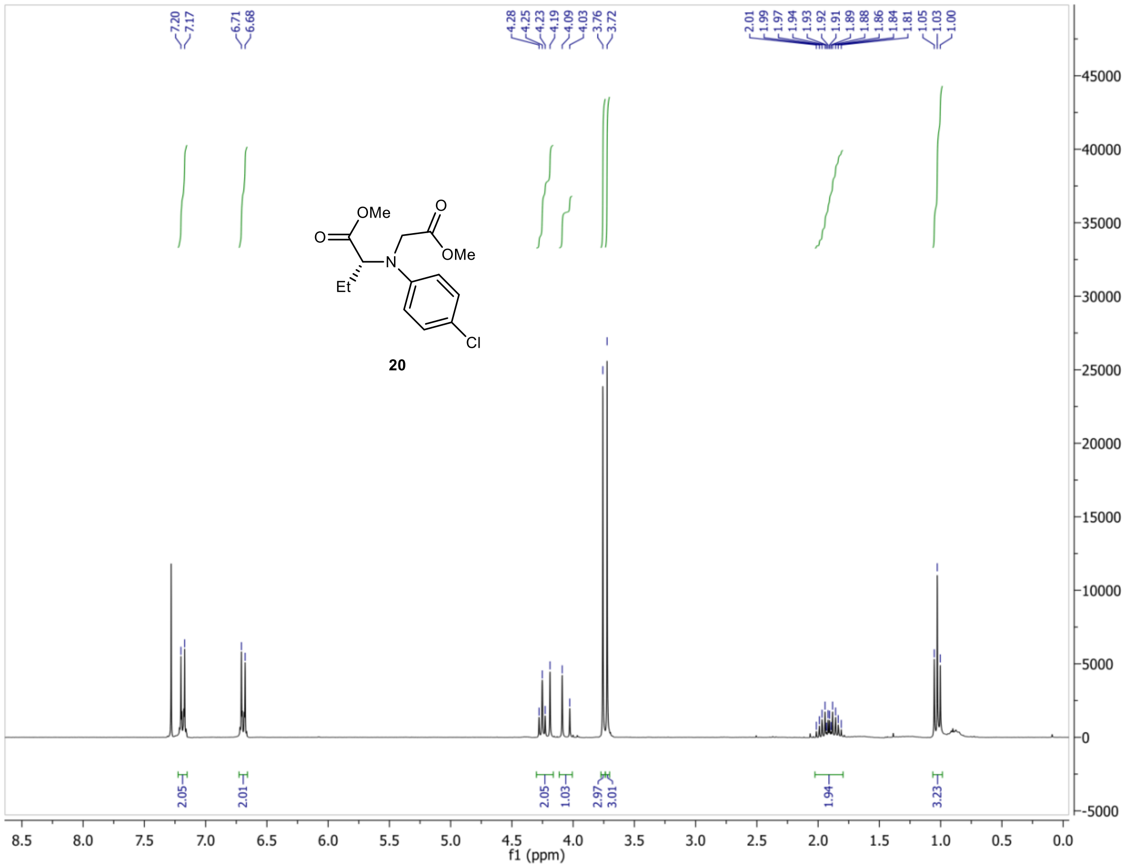

Supplementary Figure 89. ^{1}H NMR (500 MHz, $CDCl_3$) spectrum for 20

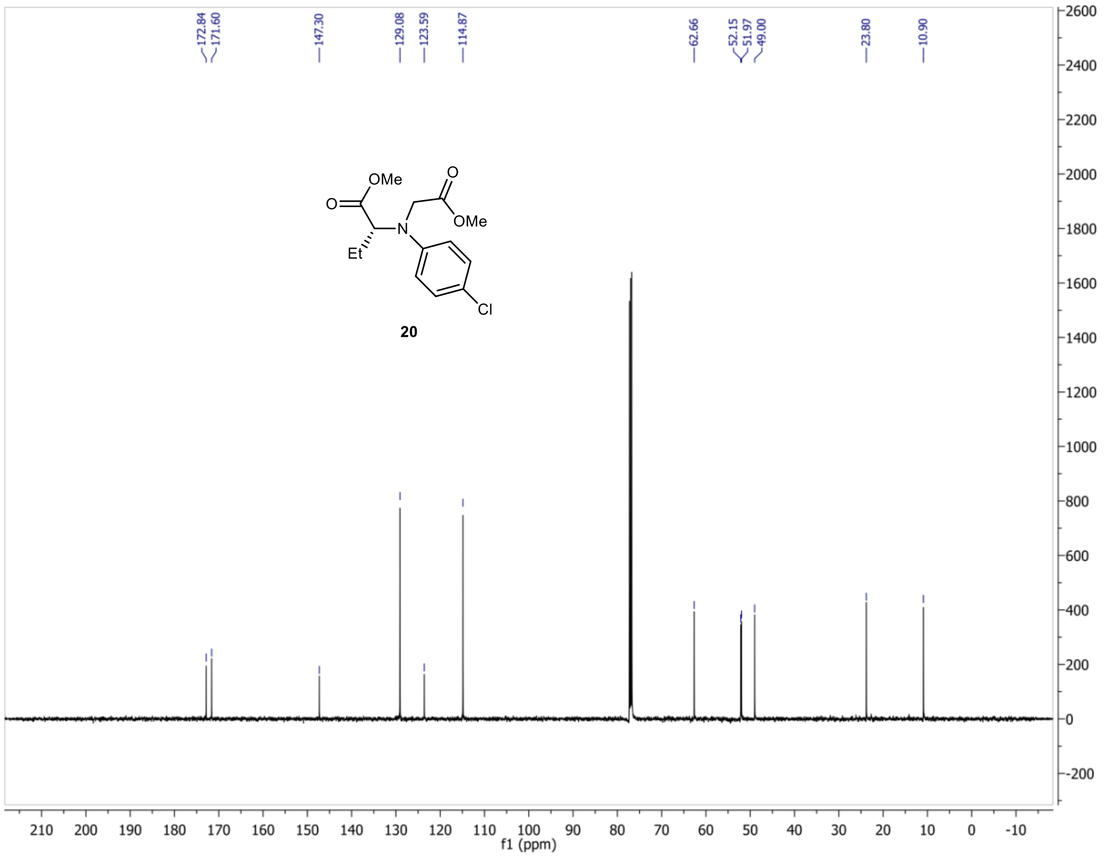

Supplementary Figure 90. ^{13}C NMR (126 MHz, $CDCl_3$) spectrum for 20

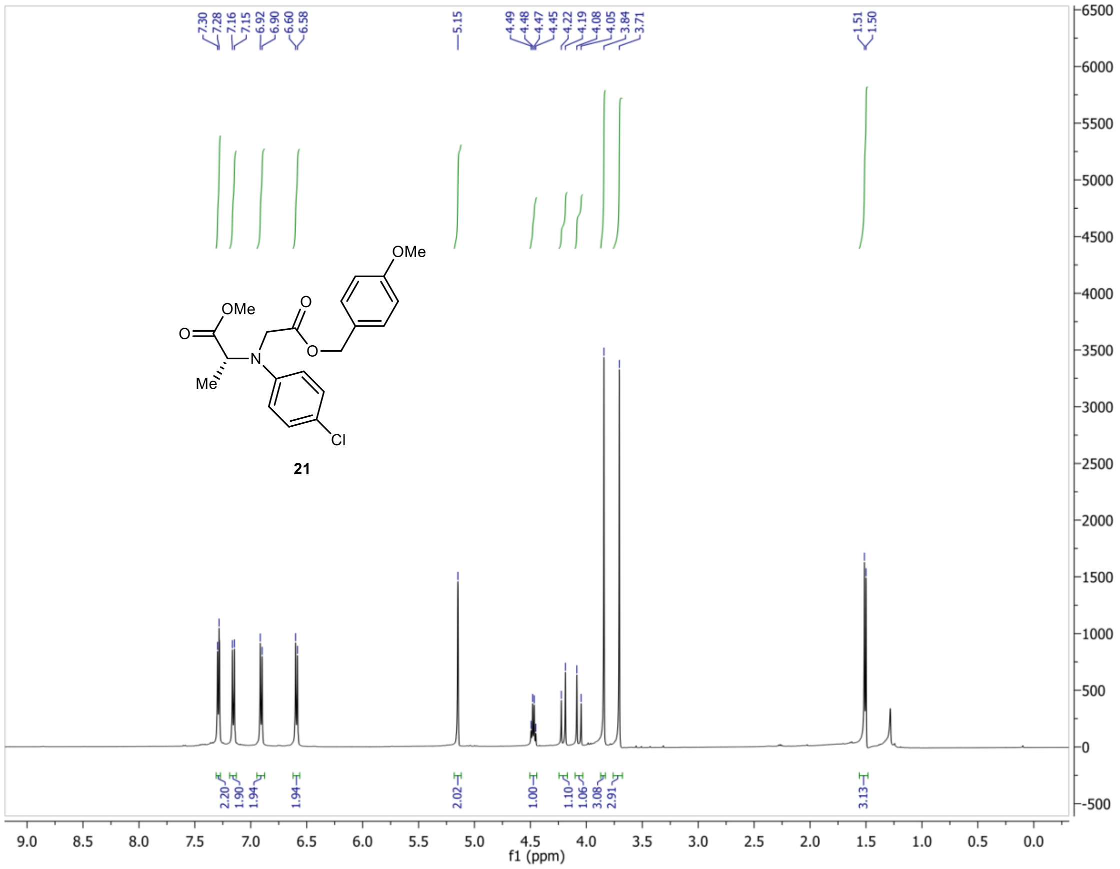

Supplementary Figure 91. ^{1}H NMR (500 MHz, $CDCl_3$) spectrum for 21

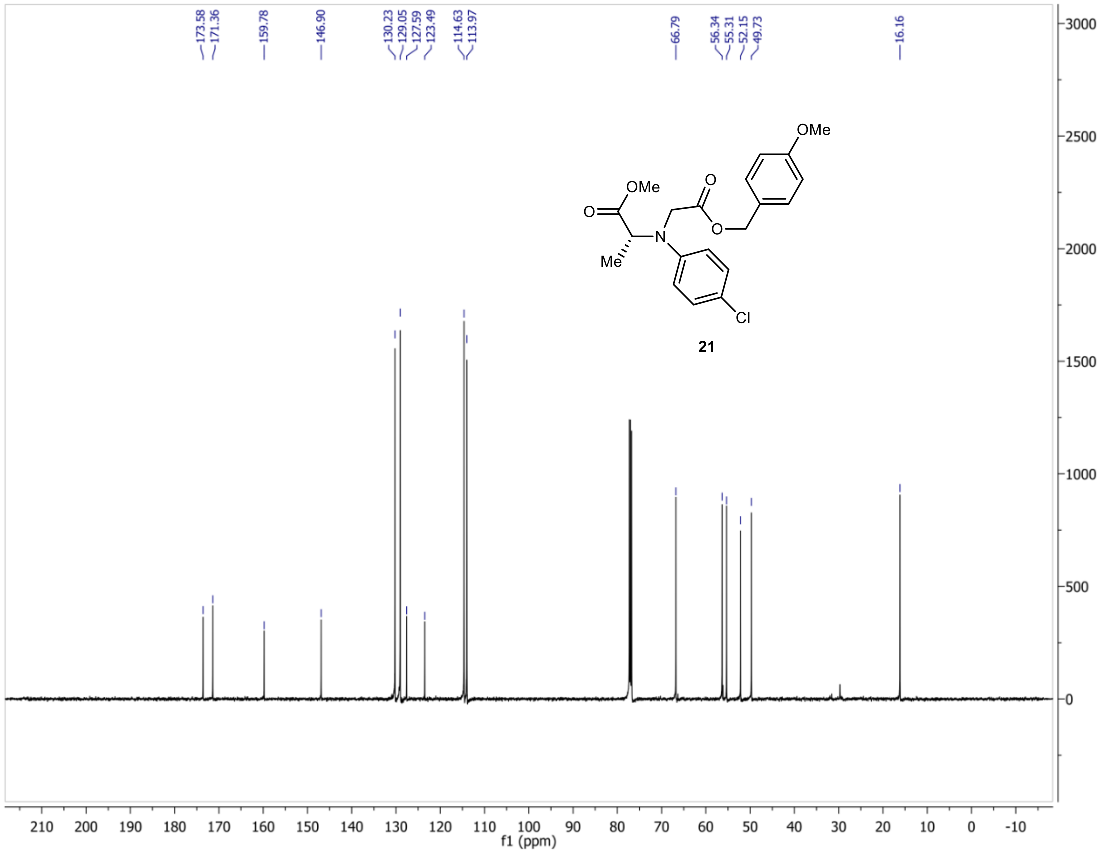

Supplementary Figure 92. ^{13}C NMR (126 MHz, $CDCl_3$) spectrum for 21

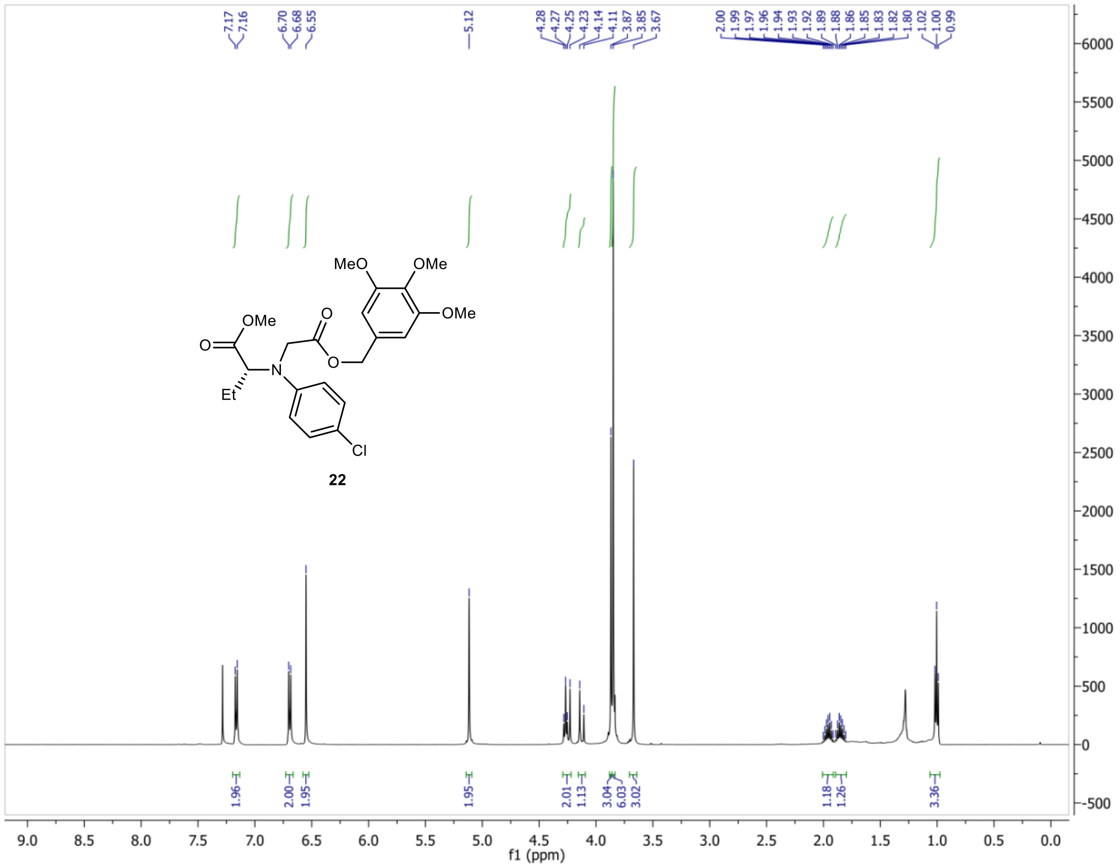

Supplementary Figure 93. ^{1}H NMR (500 MHz, $CDCl_3$) spectrum for 22

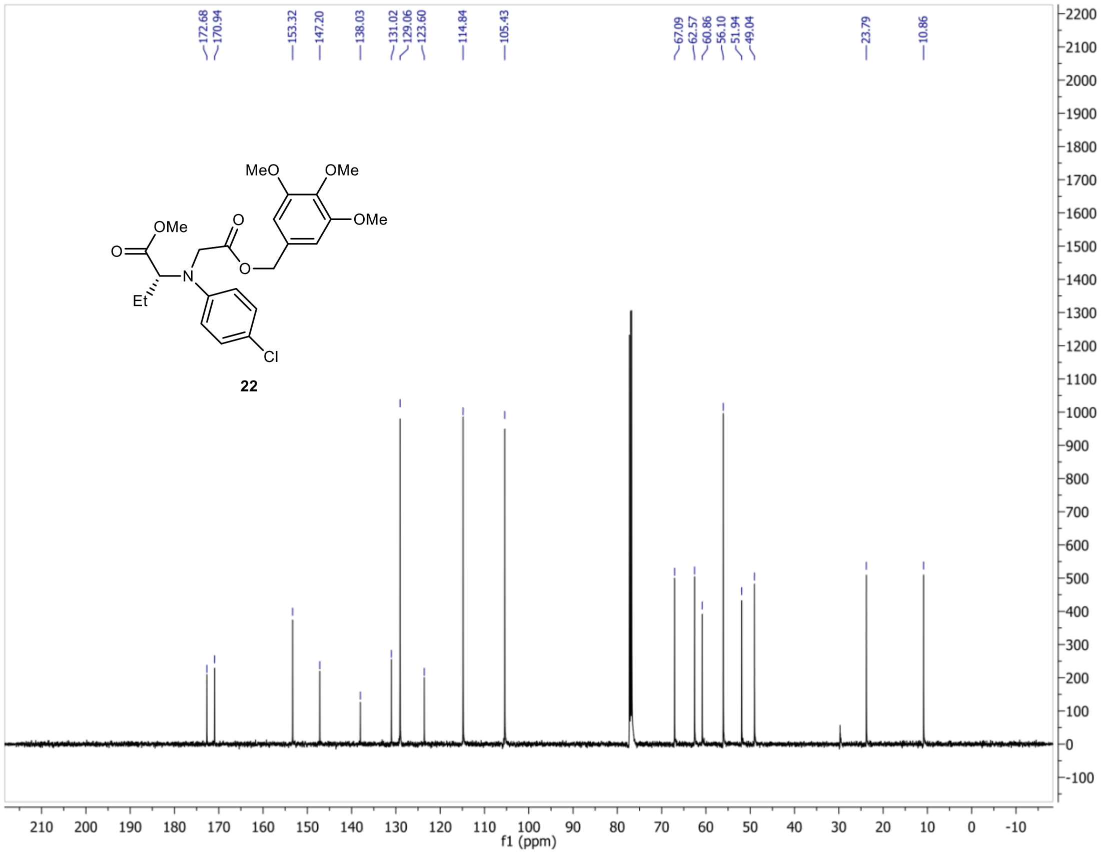

Supplementary Figure 94. ^{13}C NMR (126 MHz, $CDCl_3$) spectrum for 22

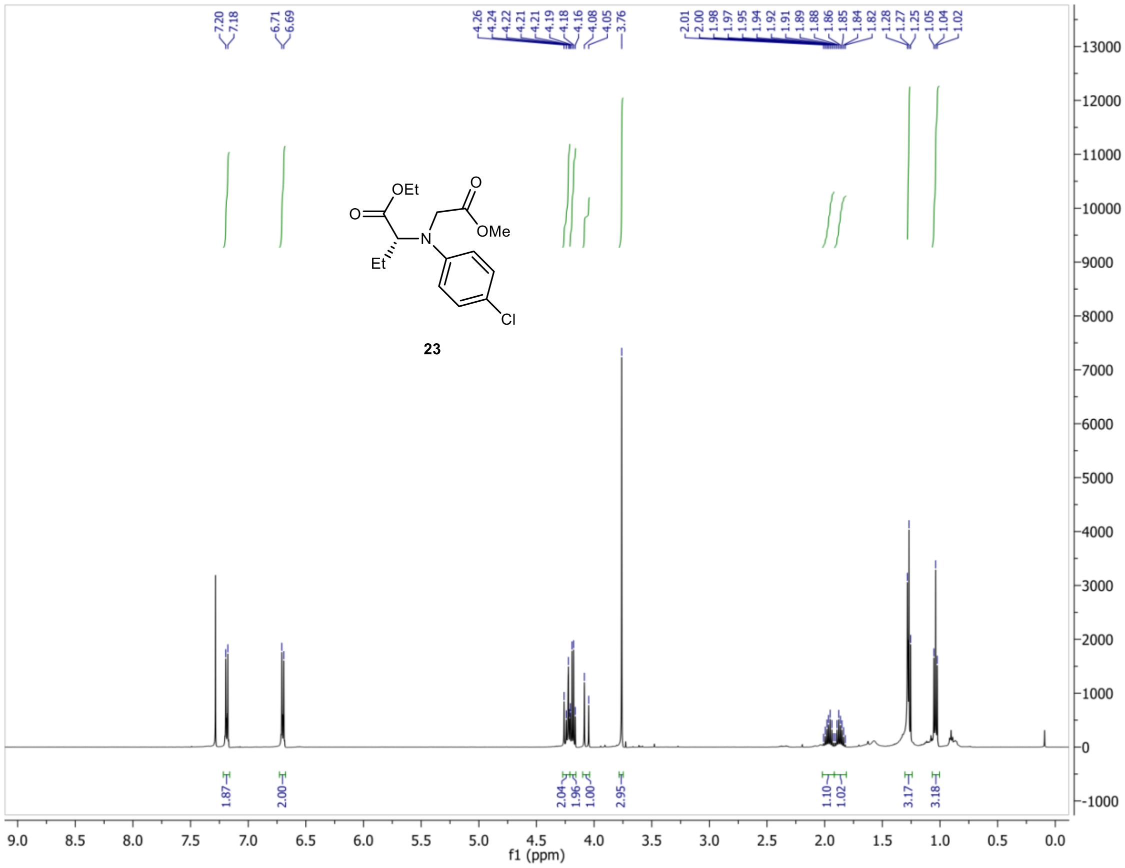

Supplementary Figure 95. ^{1}H NMR (500 MHz, $CDCl_3$) spectrum for 23

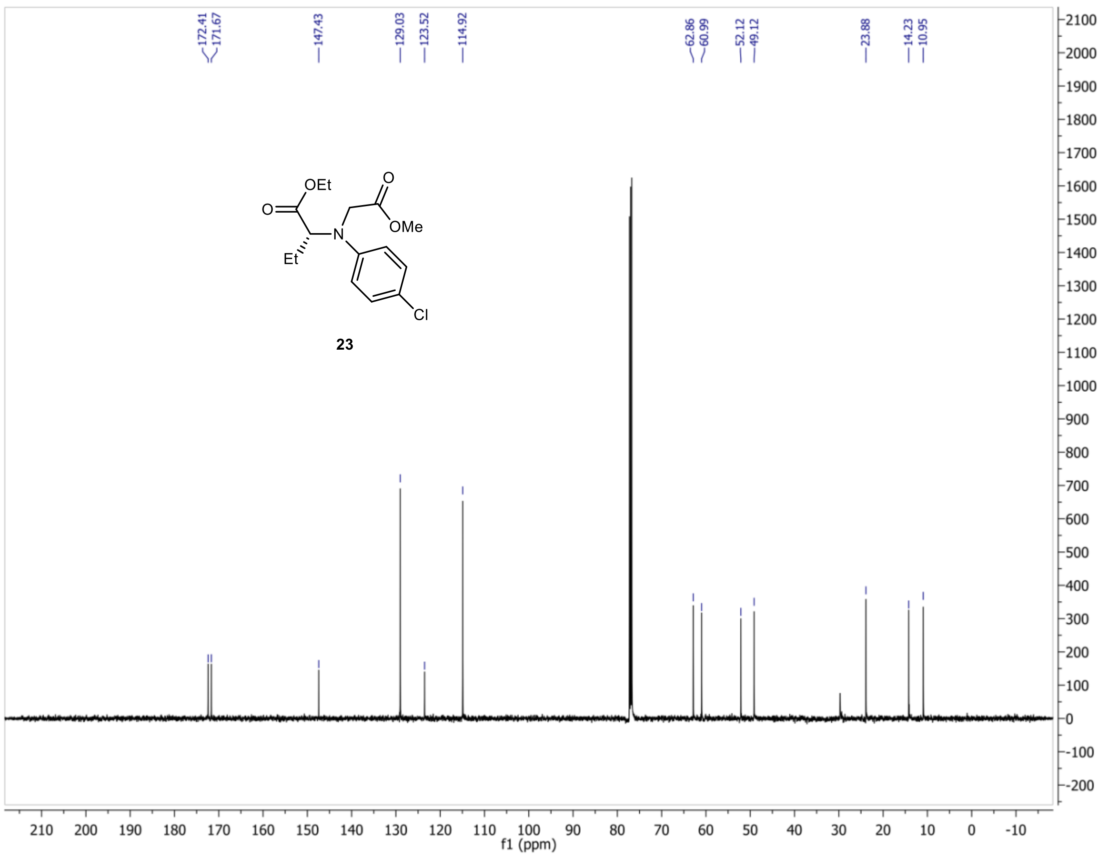

Supplementary Figure 96. ^{13}C NMR (126 MHz, $CDCl_3$) spectrum for 23

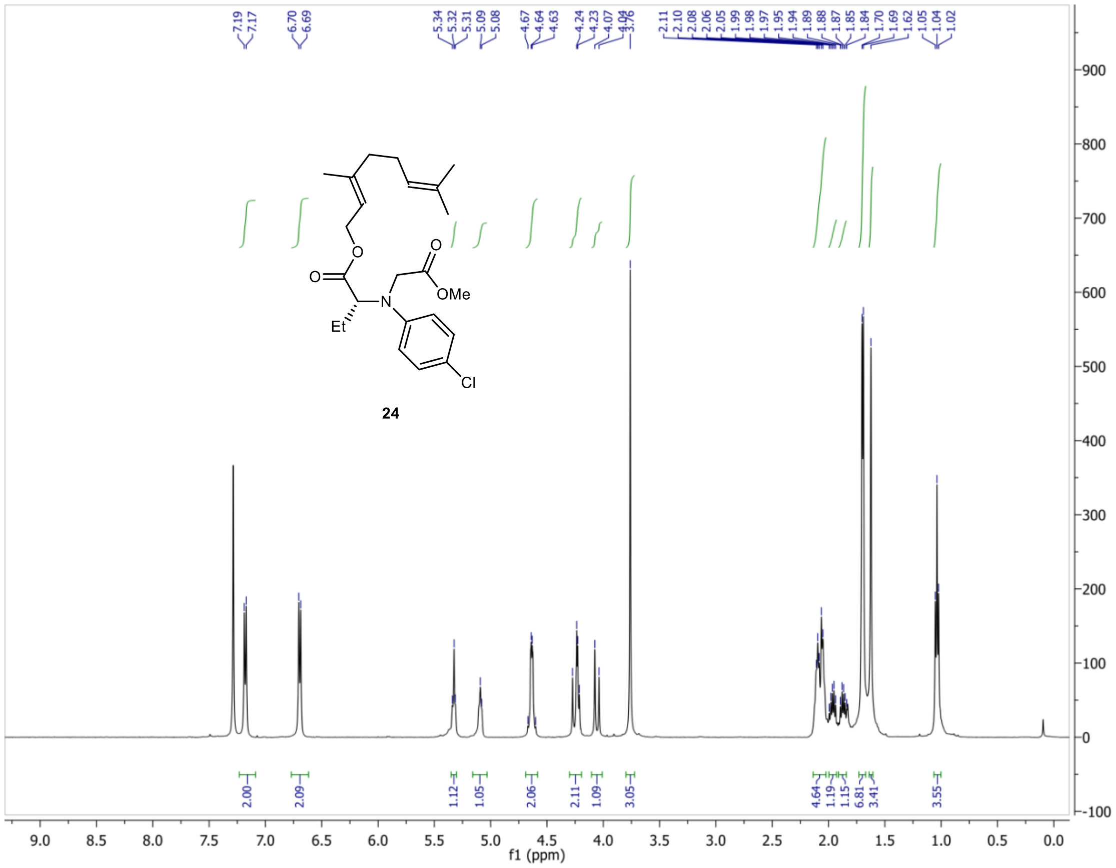

Supplementary Figure 97. ^{1}H NMR (500 MHz, $CDCl_3$) spectrum for 24

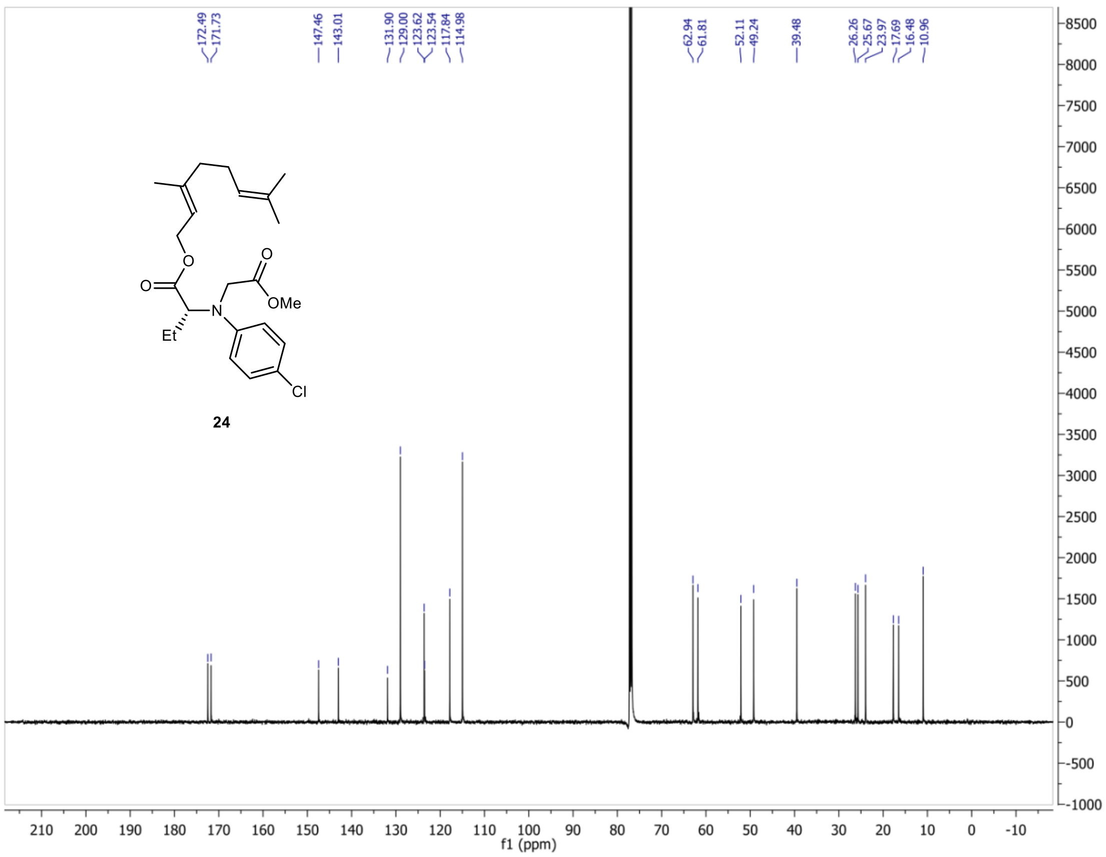

Supplementary Figure 98. ^{13}C NMR (126 MHz, $CDCl_3$) spectrum for 24

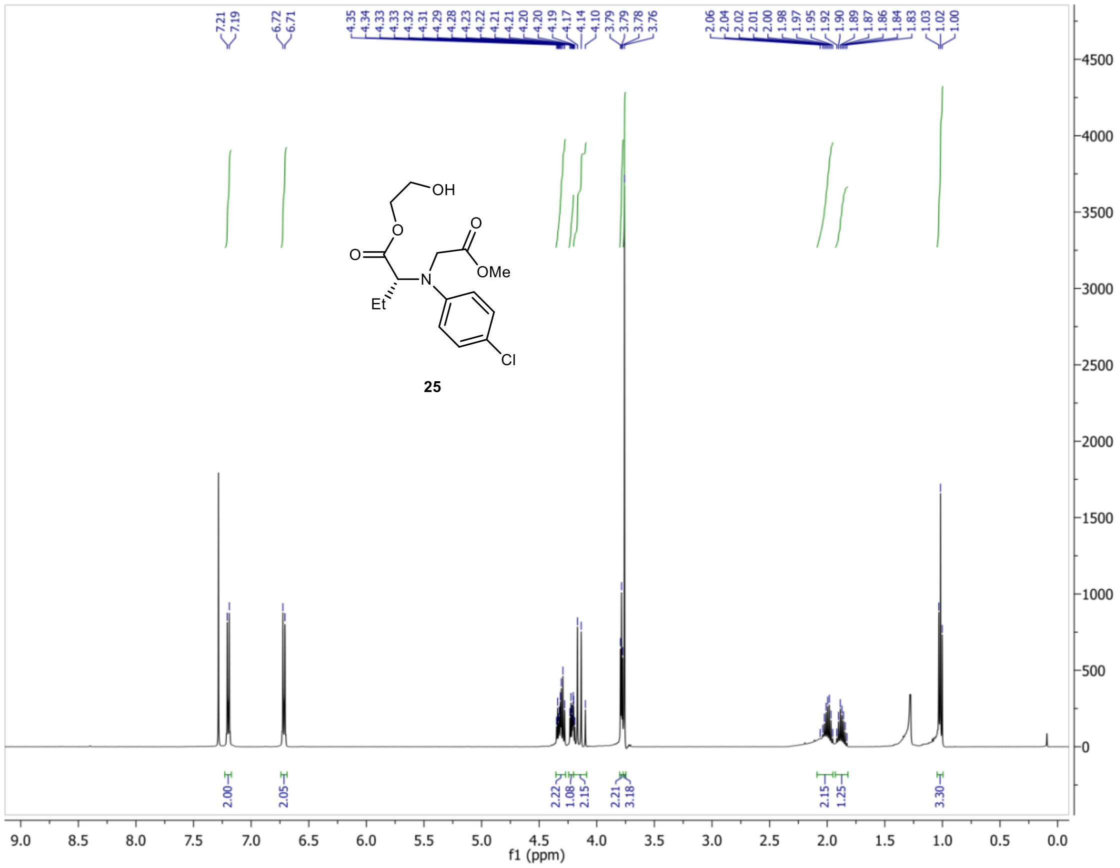

25

Supplementary Figure 99. ^{1}H NMR (500 MHz, $CDCl_3$) spectrum for 25

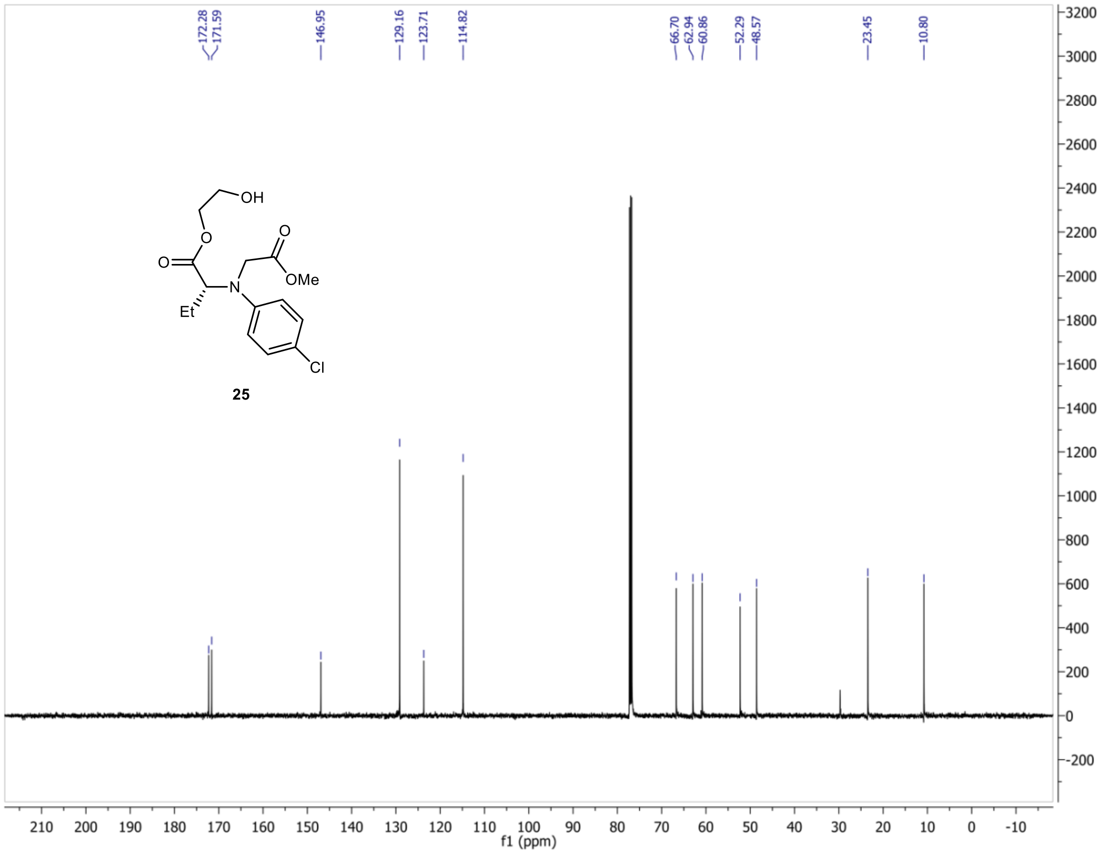

25

Supplementary Figure 100. ^{13}C NMR (126 MHz, $CDCl_3$) spectrum for 25

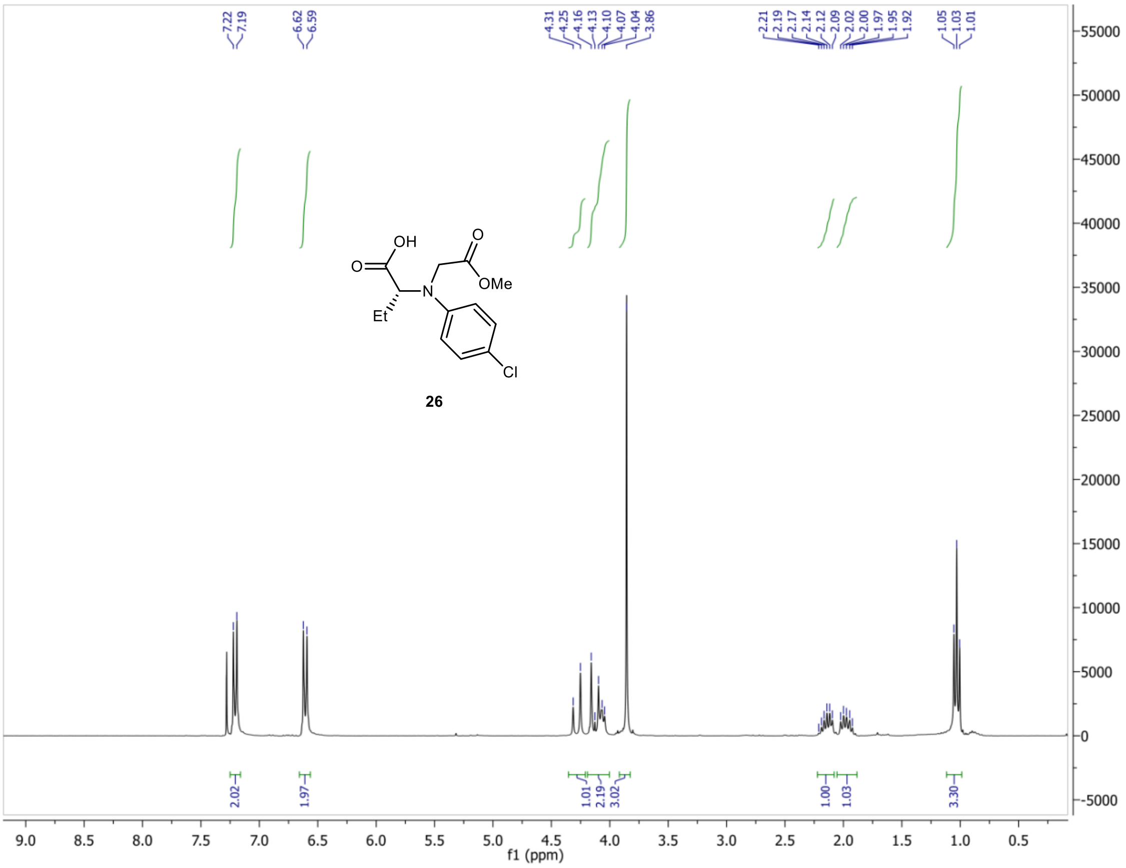

Supplementary Figure 101. ^{1}H **NMR (500 MHz,** $CDCl_3$**) spectrum for 26**

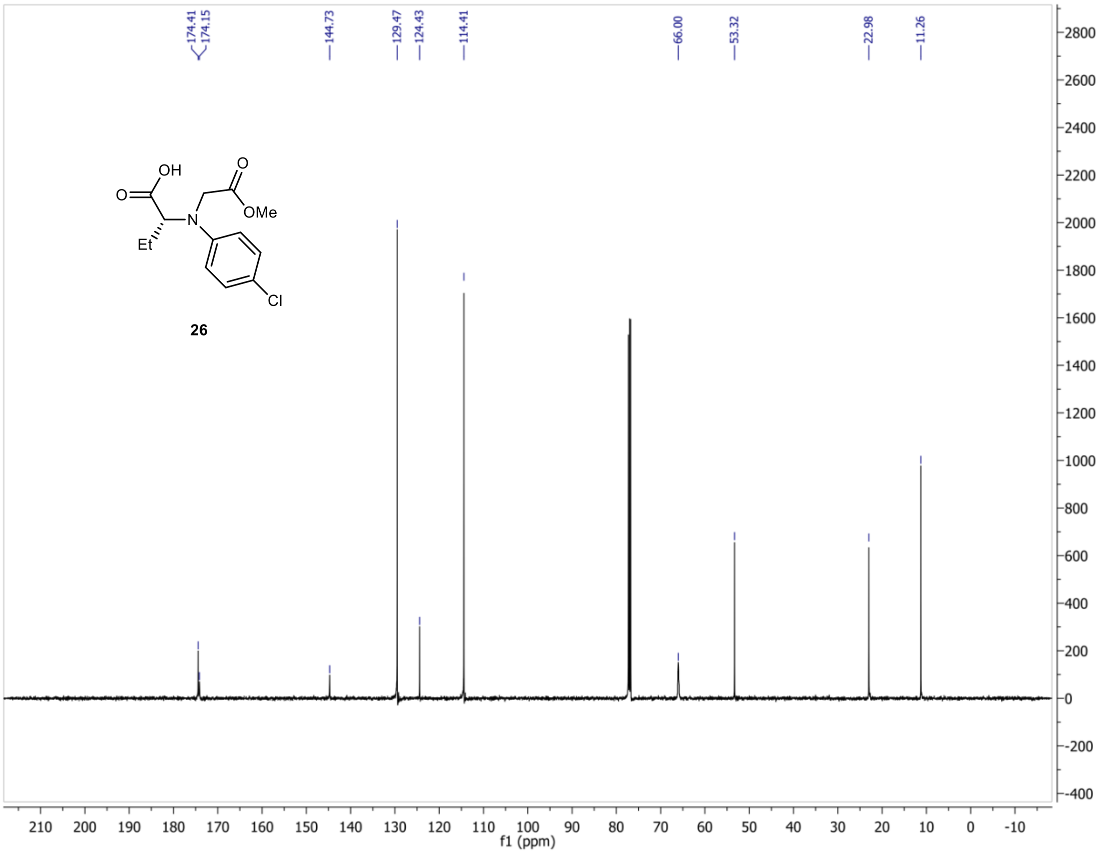

Supplementary Figure 102. ^{13}C **NMR (126 MHz,** $CDCl_3$**) spectrum for 26**

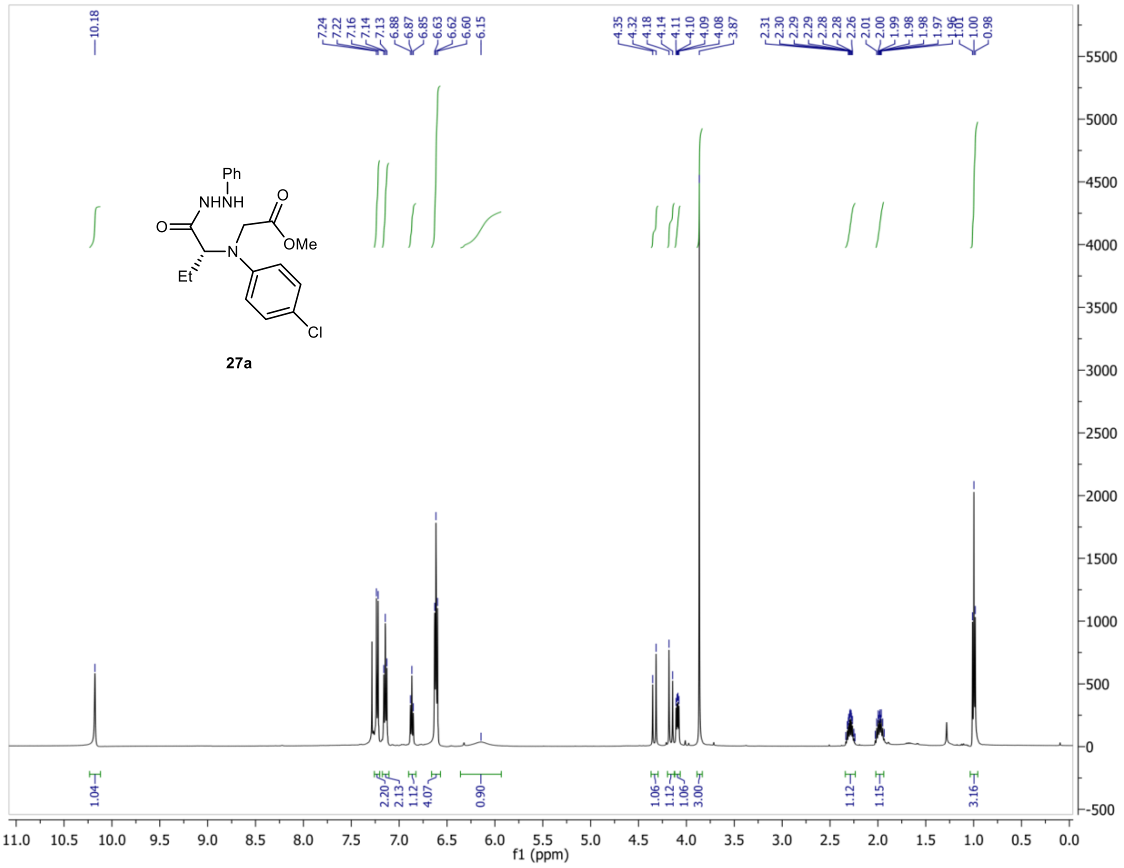

Supplementary Figure 103. ^{1}H NMR (500 MHz, $CDCl_3$) spectrum for 27a

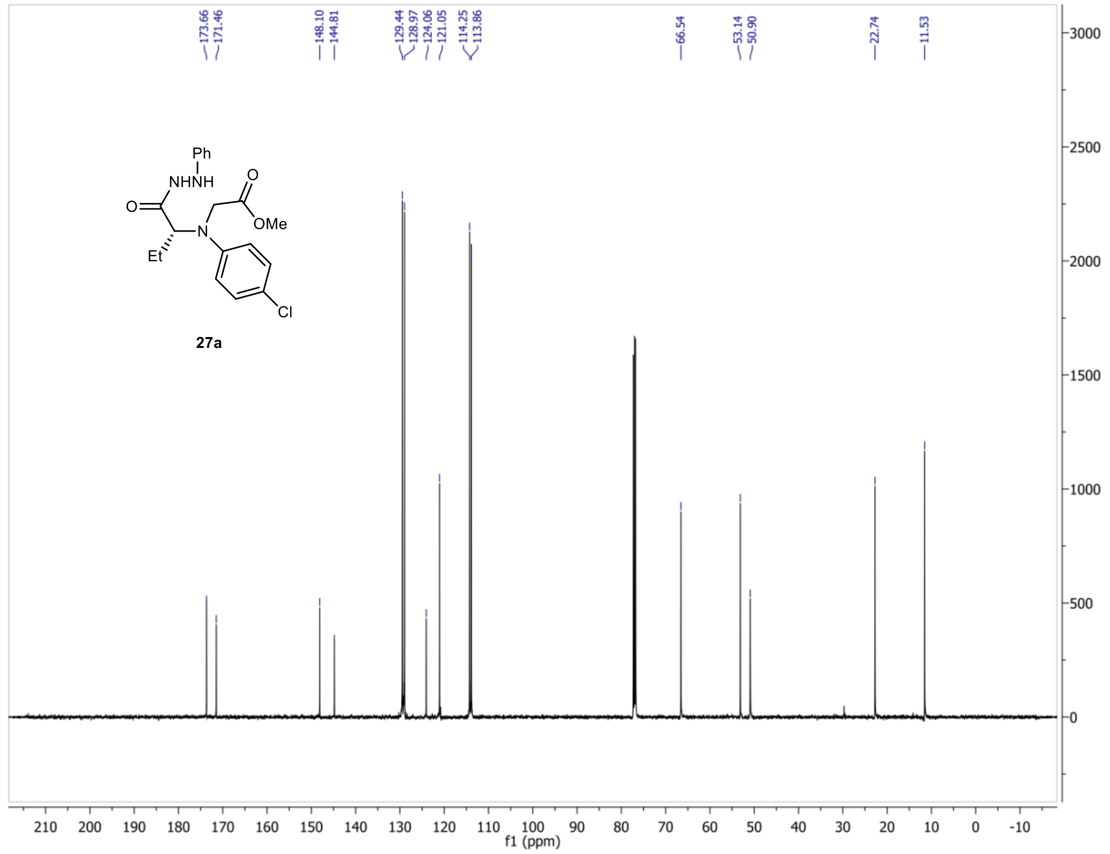

Supplementary Figure 104. ^{13}C NMR (126 MHz, $CDCl_3$) spectrum for 27a

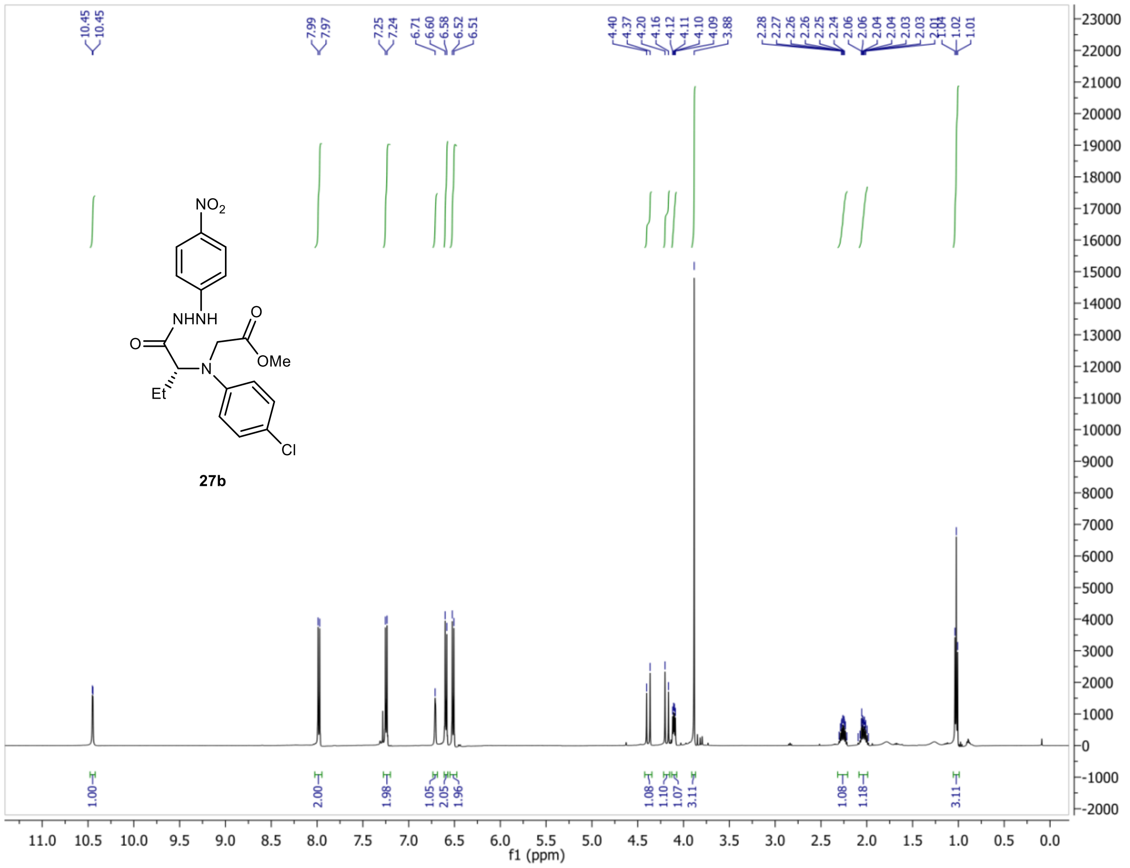

Supplementary Figure 105. ^{1}H NMR (500 MHz, $CDCl_3$) spectrum for 27b

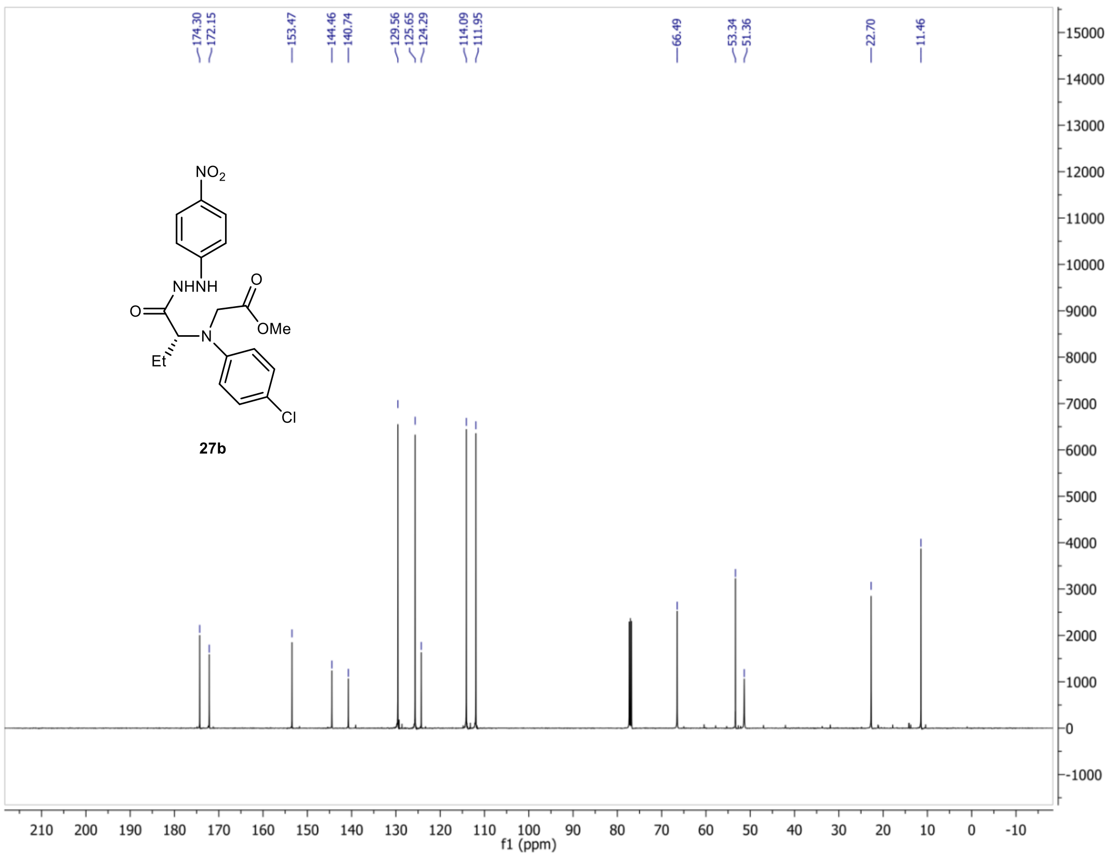

Supplementary Figure 106. ^{13}C NMR (126 MHz, $CDCl_3$) spectrum for 27b

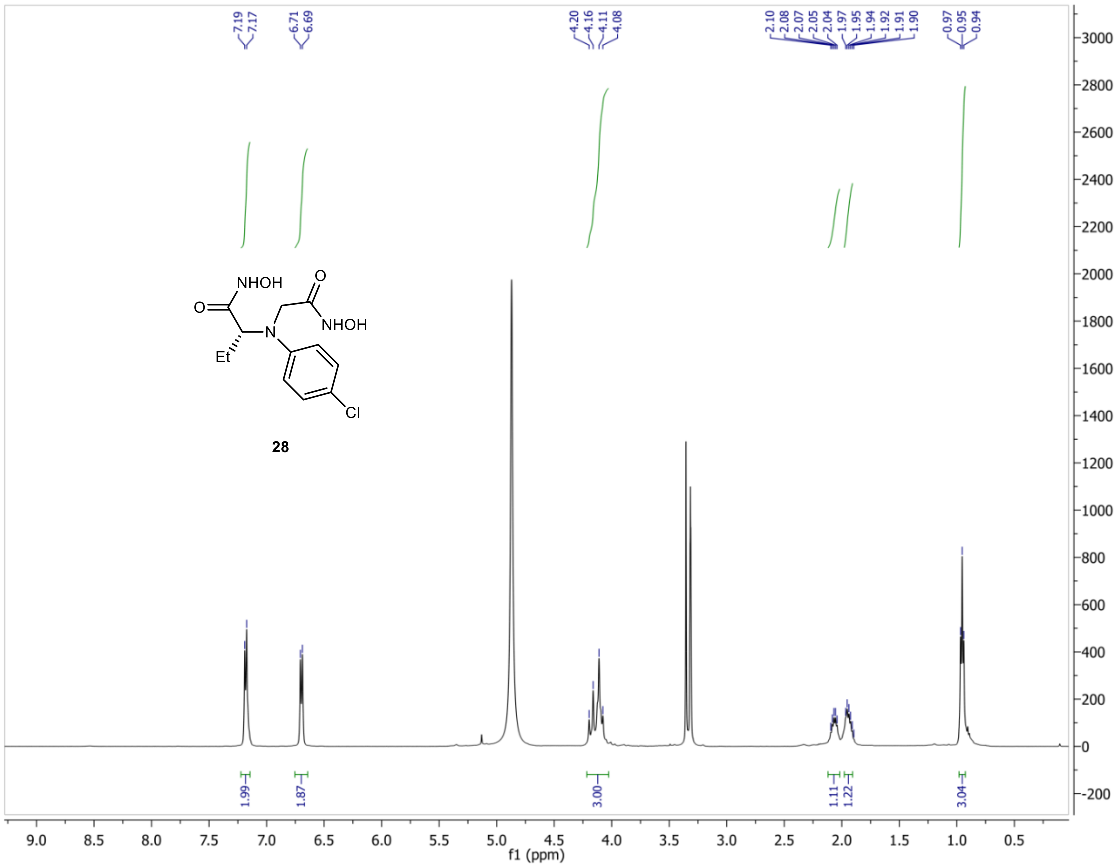

Supplementary Figure 107. ^{1}H NMR (500 MHz, CD_3OD) spectrum for 28

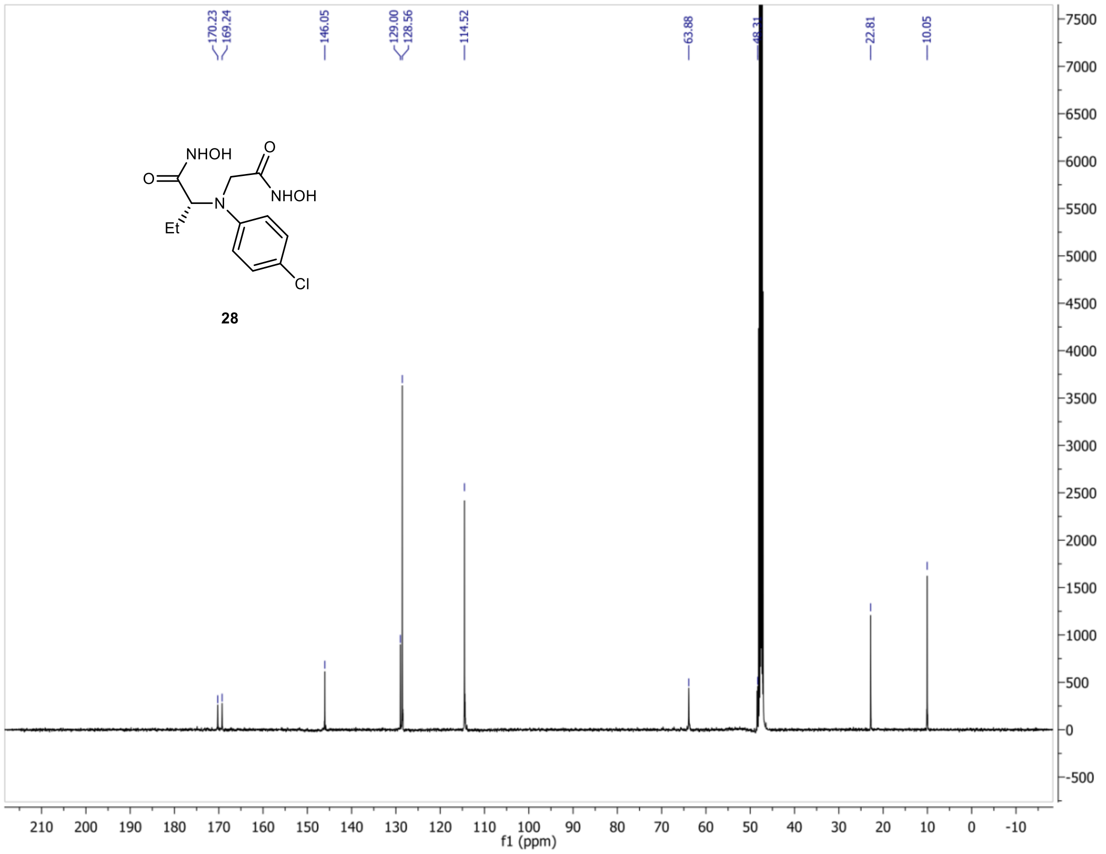

Supplementary Figure 108. ^{13}C NMR (126 MHz, CD_3OD) spectrum for 28

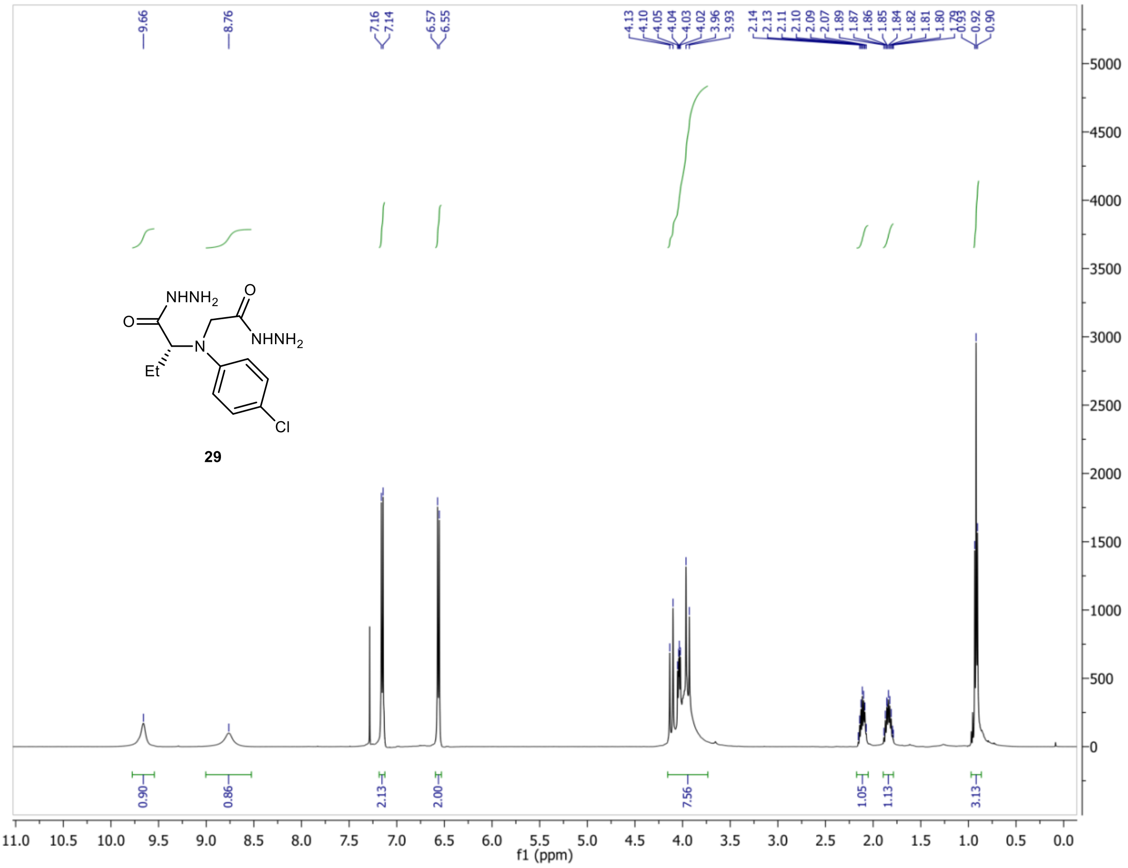

Supplementary Figure 109. ^{1}H NMR (500 MHz, $CDCl_3$) spectrum for 29

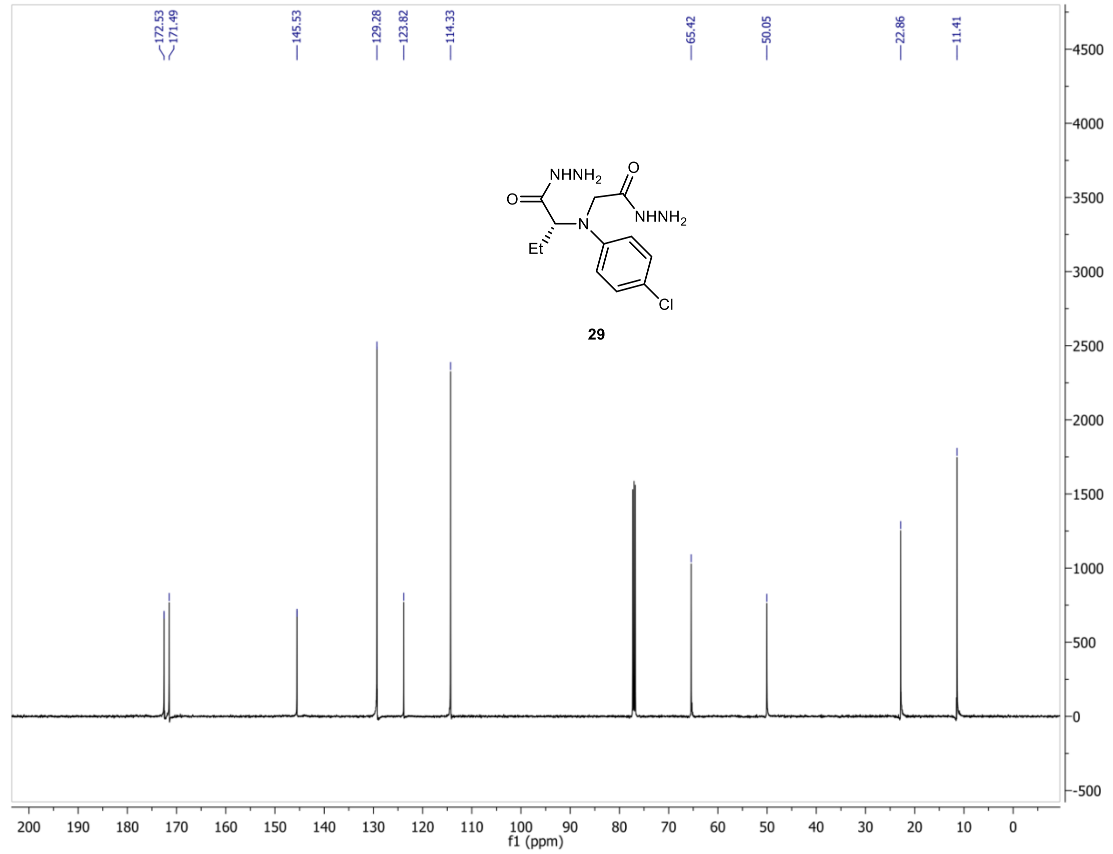

Supplementary Figure 110. ^{13}C NMR (126 MHz, $CDCl_3$) spectrum for 29

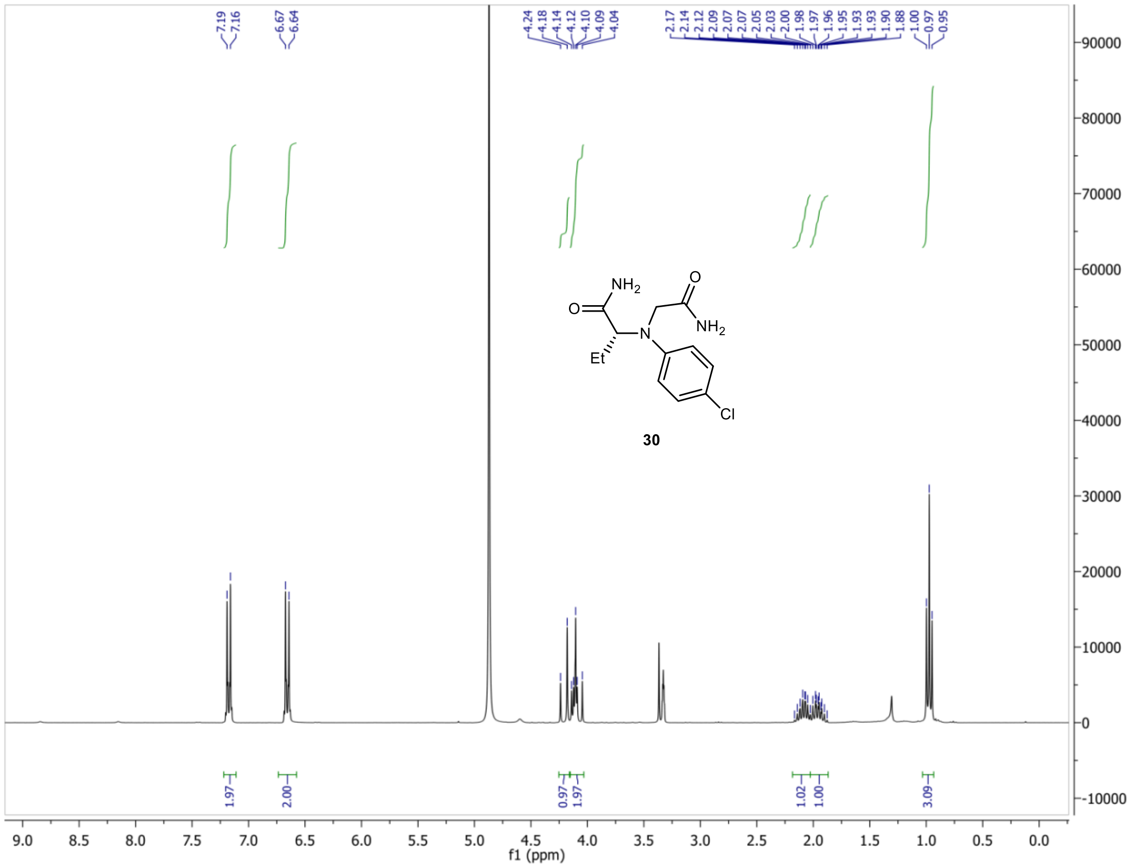

Supplementary Figure 111. ^{1}H NMR (300 MHz, CD_3OD) spectrum for 30

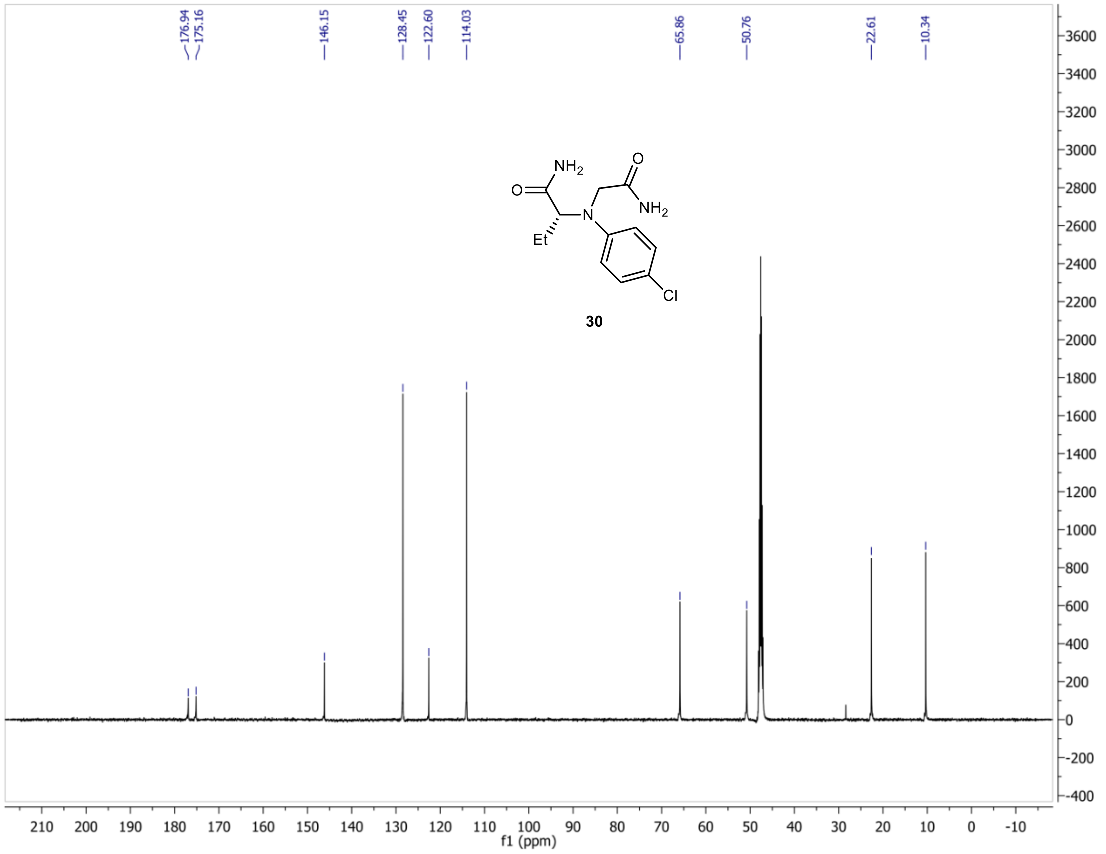

Supplementary Figure 112. ^{13}C NMR (126 MHz, CD_3OD) spectrum for 30

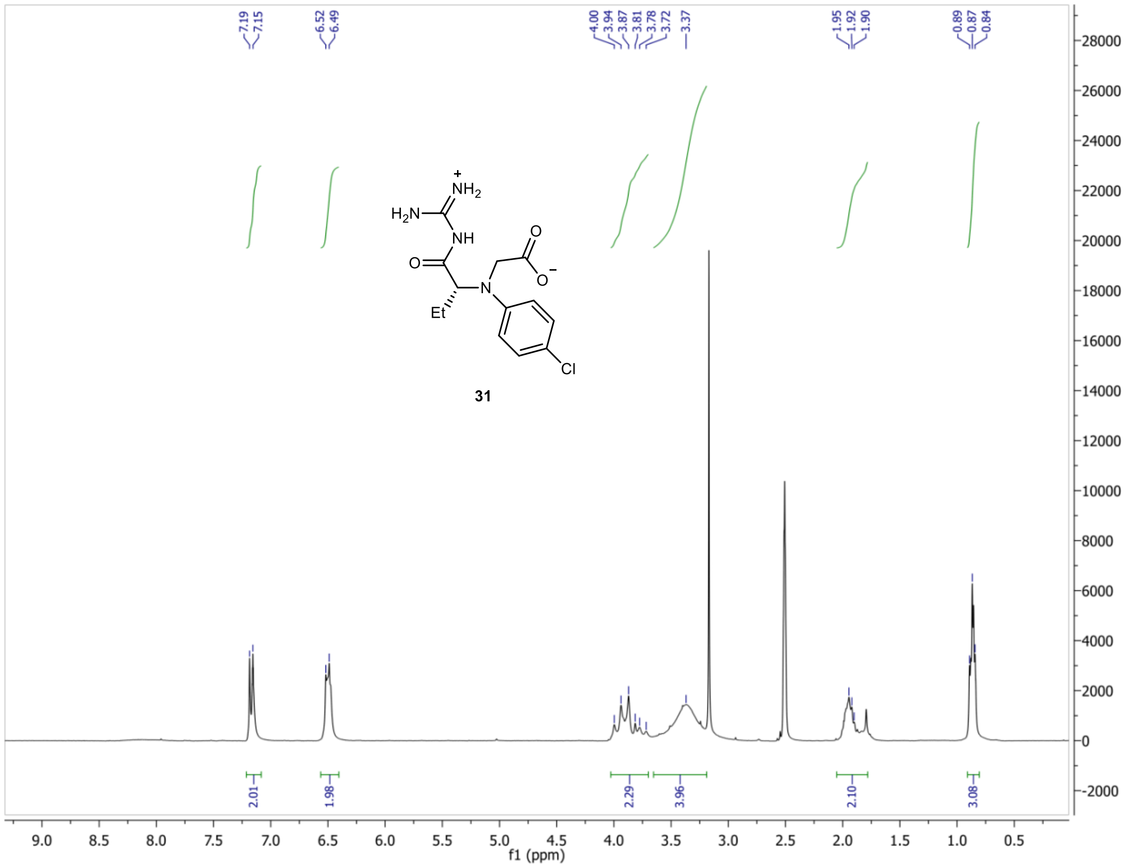

Supplementary Figure 113. ^{1}H NMR (300 MHz, DMSO-d_6) spectrum for 31

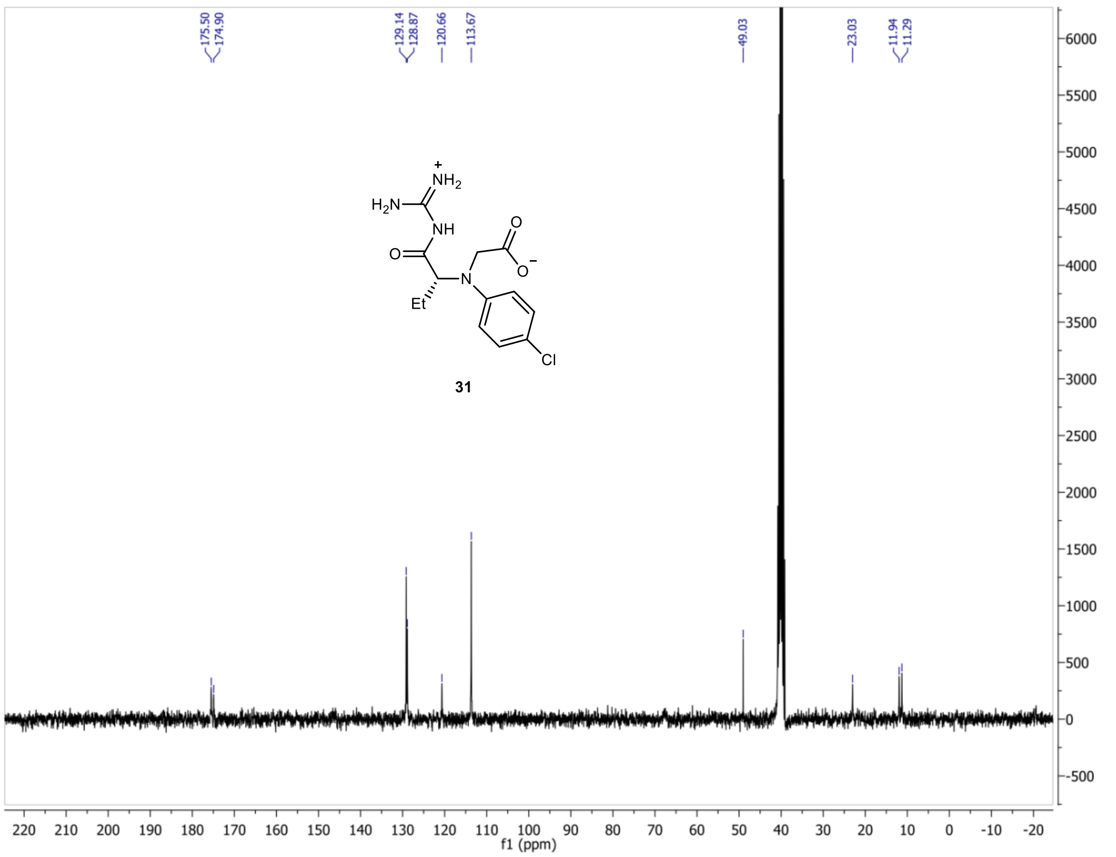

Supplementary Figure 114. ^{13}C NMR (75 MHz, DMSO-d_6) spectrum for 31

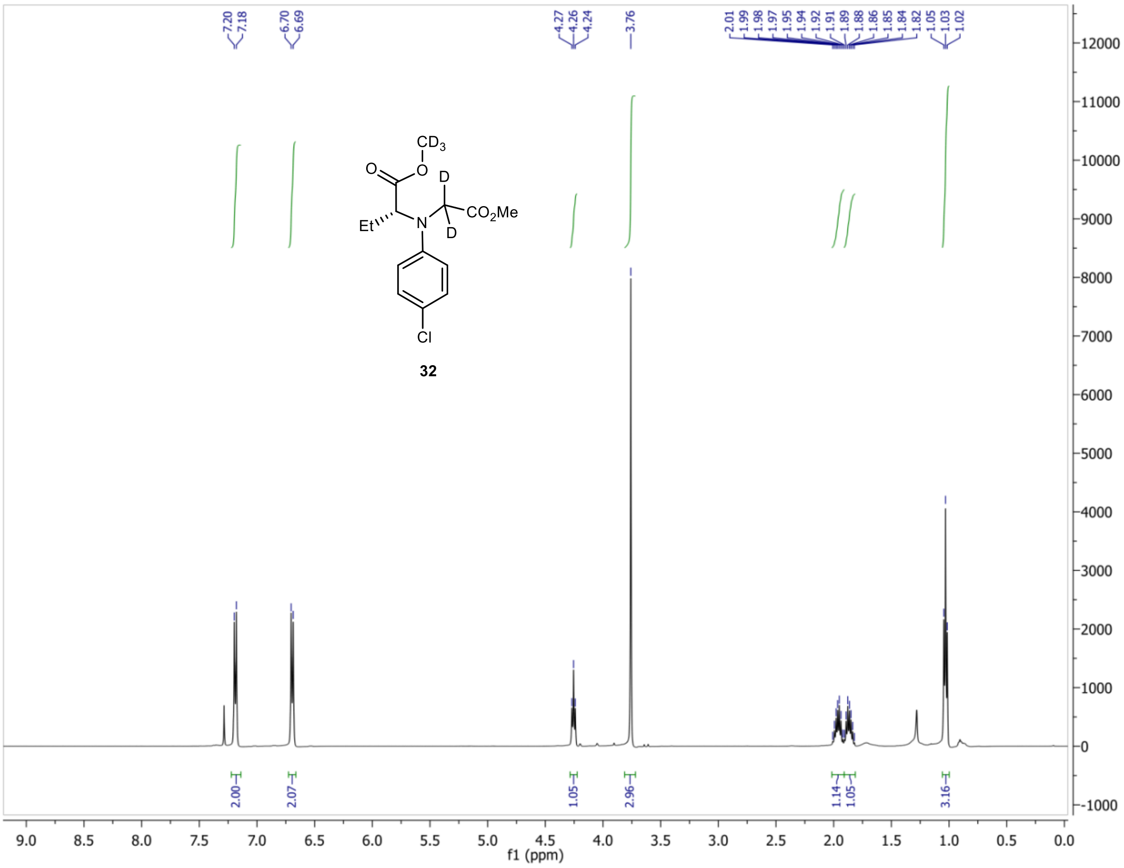

Supplementary Figure 115. ^{1}H **NMR (500 MHz, $CDCl_3$) spectrum for 32**

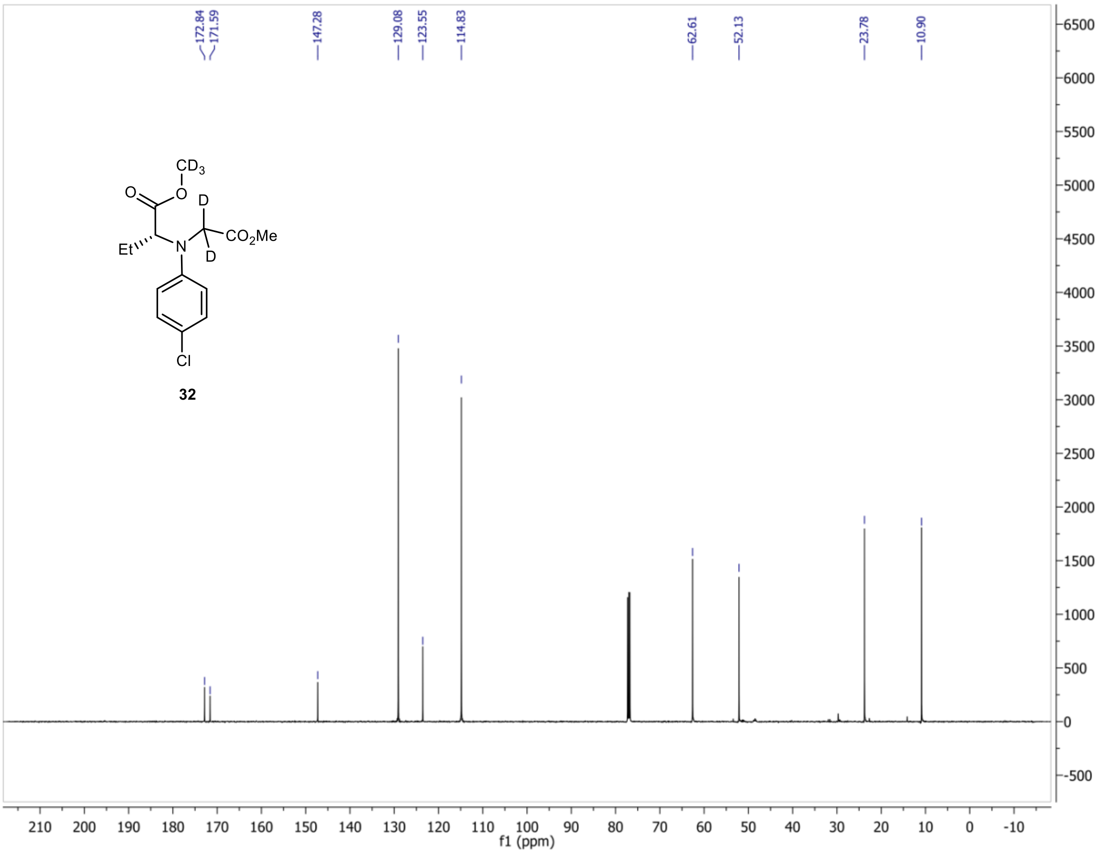

Supplementary Figure 116. ^{13}C **NMR (126 MHz, $CDCl_3$) spectrum for 32**

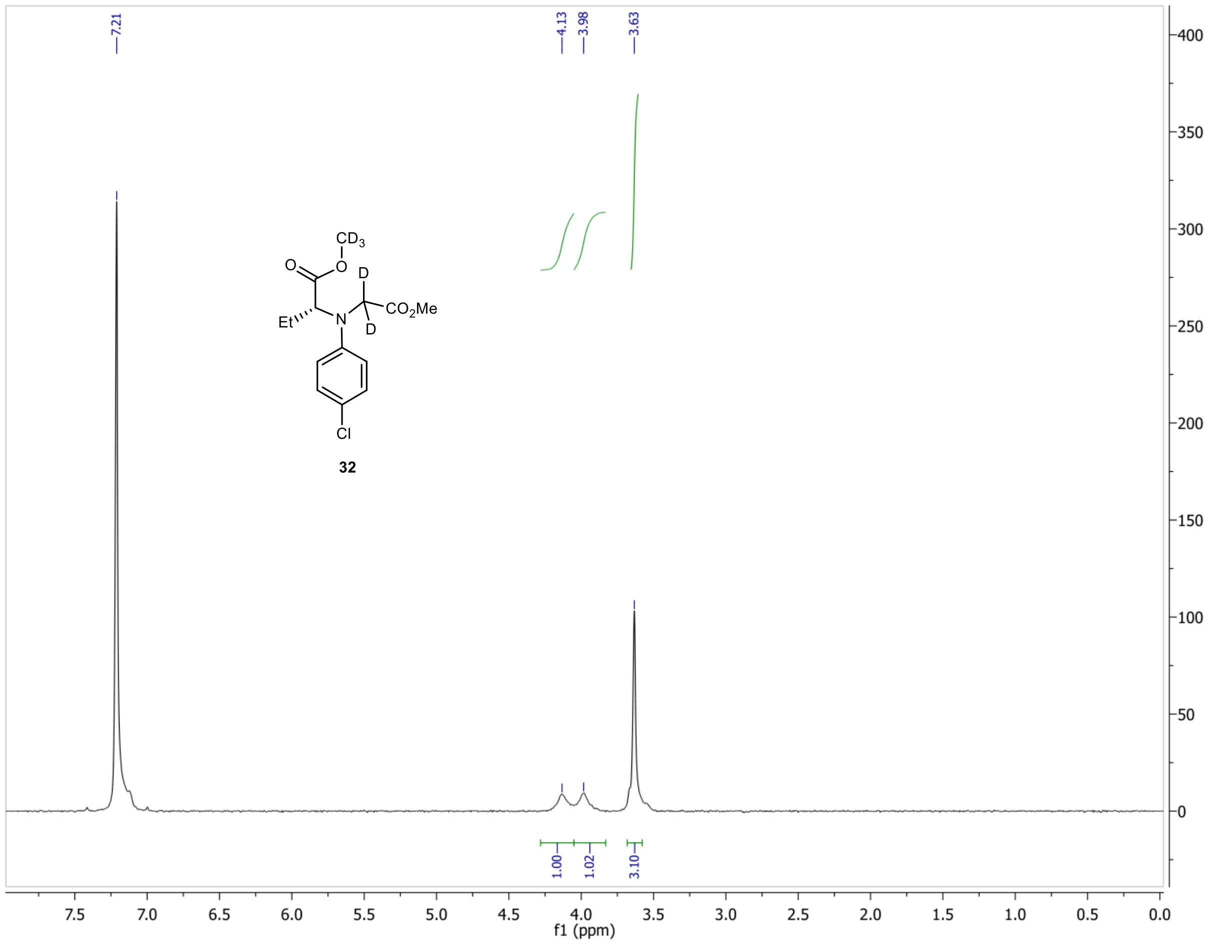

Supplementary Figure 117. ^{2}H NMR (77 MHz, $CHCl_3/CDCl_3$ 4:1) spectrum for 32

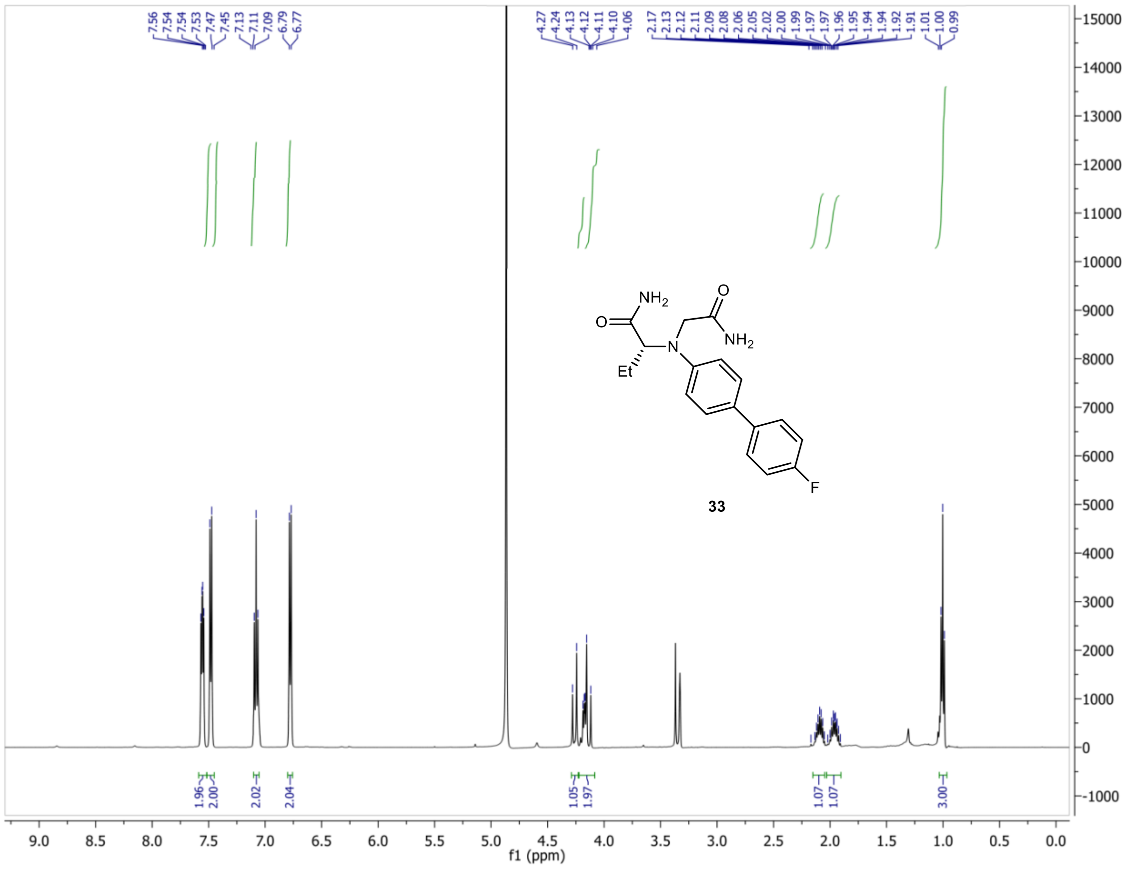

Supplementary Figure 118. **^{1}H NMR (500 MHz, CD_3OD) spectrum for 33**

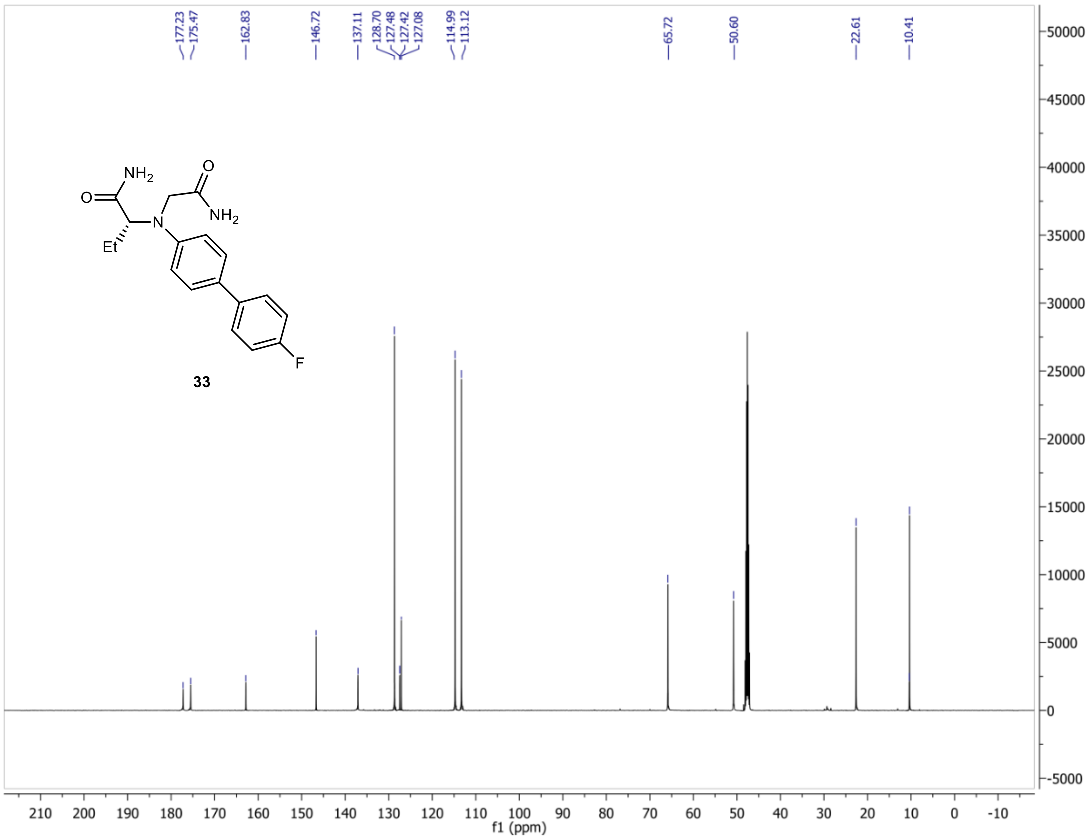

Supplementary Figure 119. **^{13}C NMR (126 MHz, CD_3OD) spectrum for 33**

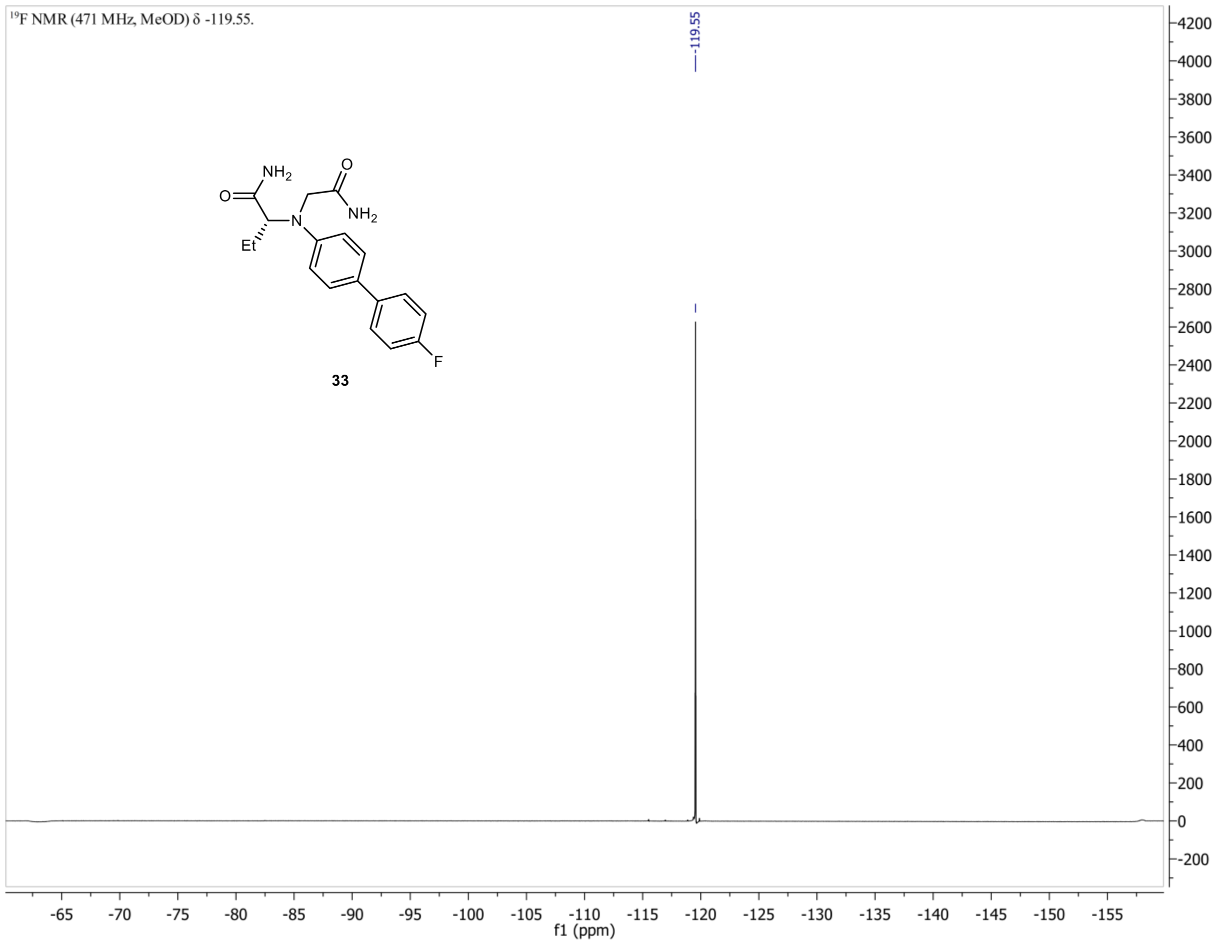

Supplementary Figure 120. ^{19}F NMR (471 MHz, CD_3OD) spectrum for 33

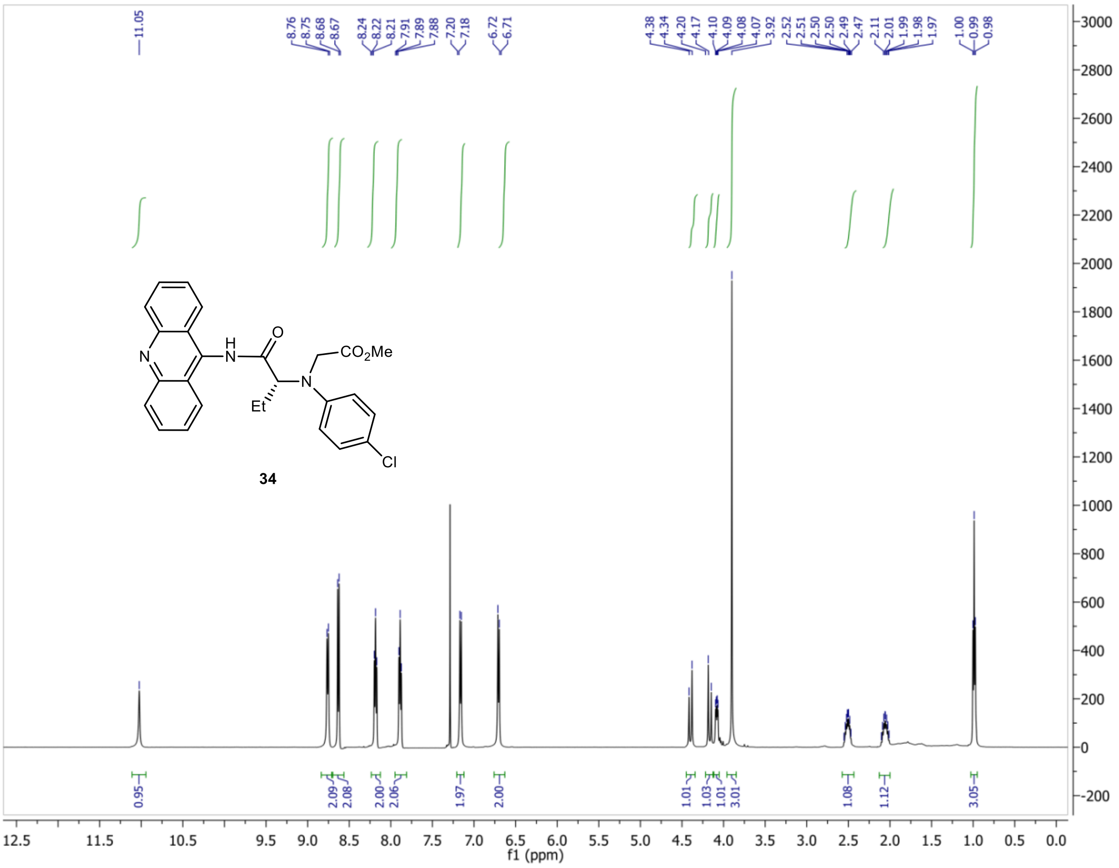

Supplementary Figure 121. ^{1}H NMR (500 MHz, $CDCl_3$) spectrum for 34

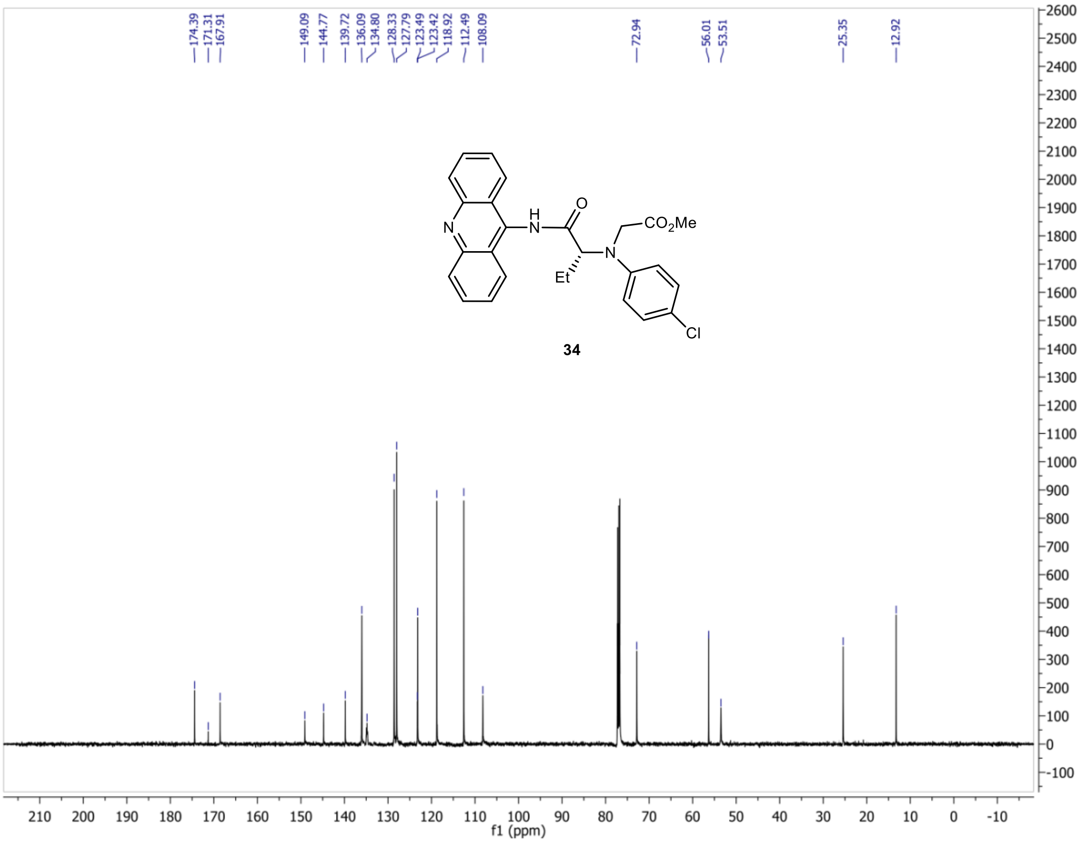

Supplementary Figure 122. ^{13}C NMR (126 MHz, $CDCl_3$) spectrum for 34

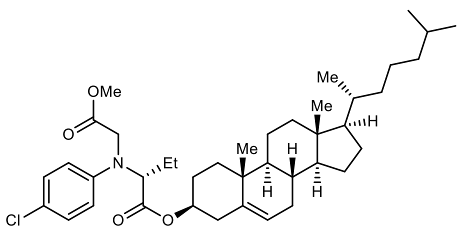

35

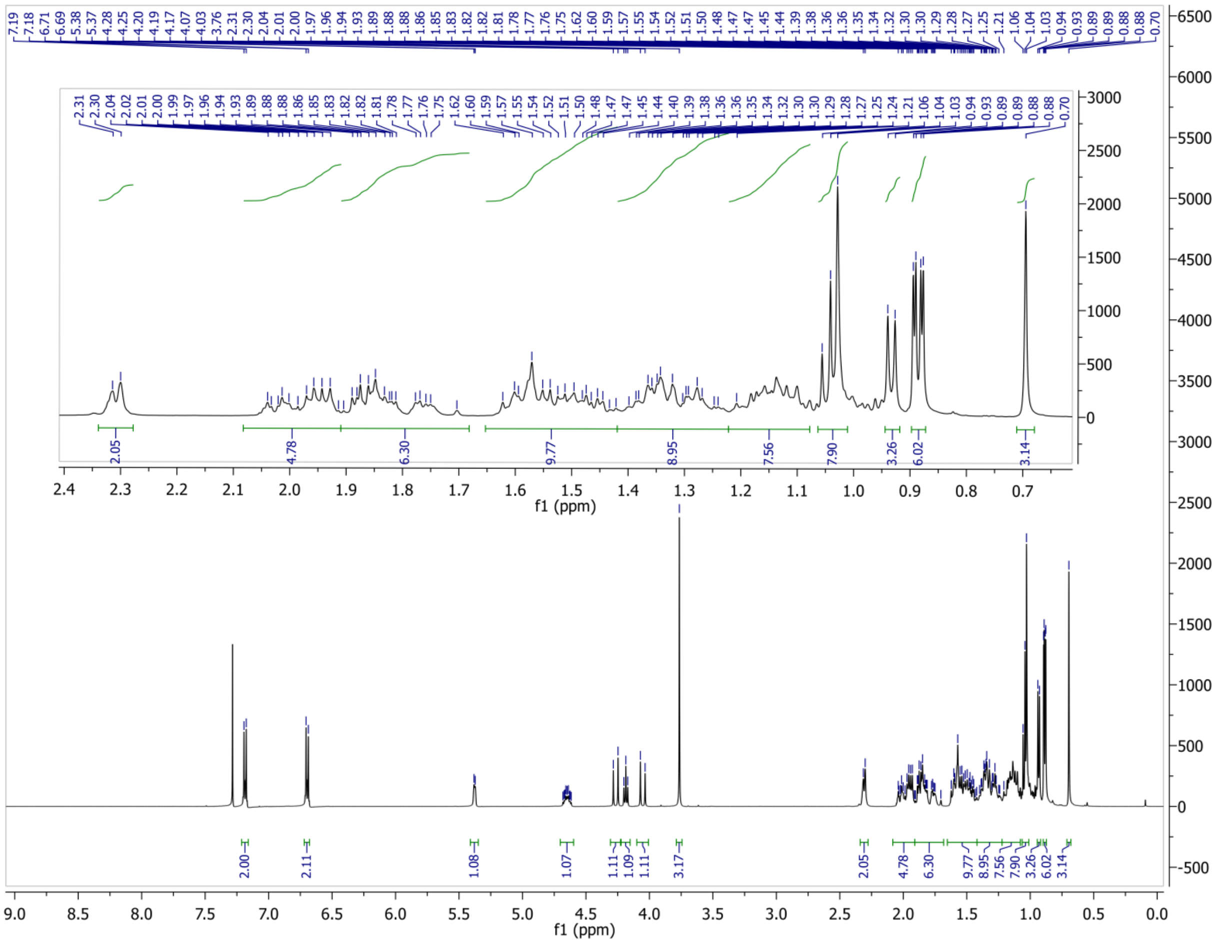

Supplementary Figure 123. ^{1}H NMR (500 MHz, $CDCl_3$) spectrum for 35

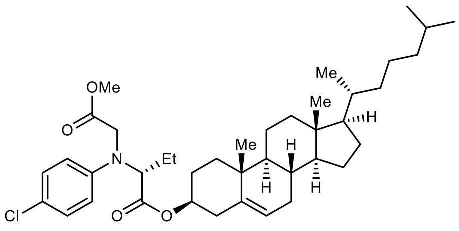

35

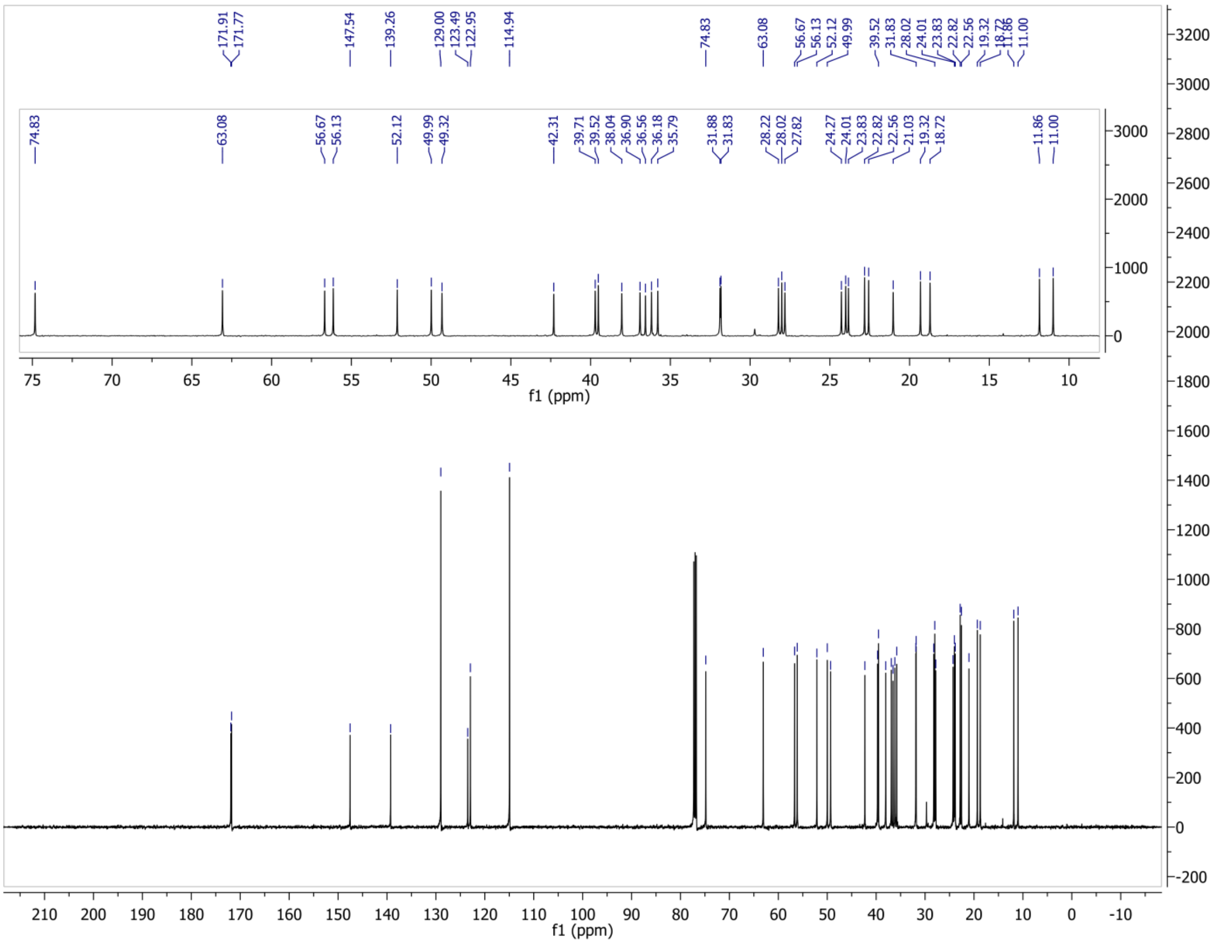

Supplementary Figure 124. ^{13}C NMR (126 MHz, $CDCl_3$) spectrum for 35

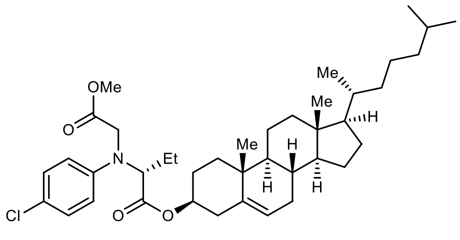

35

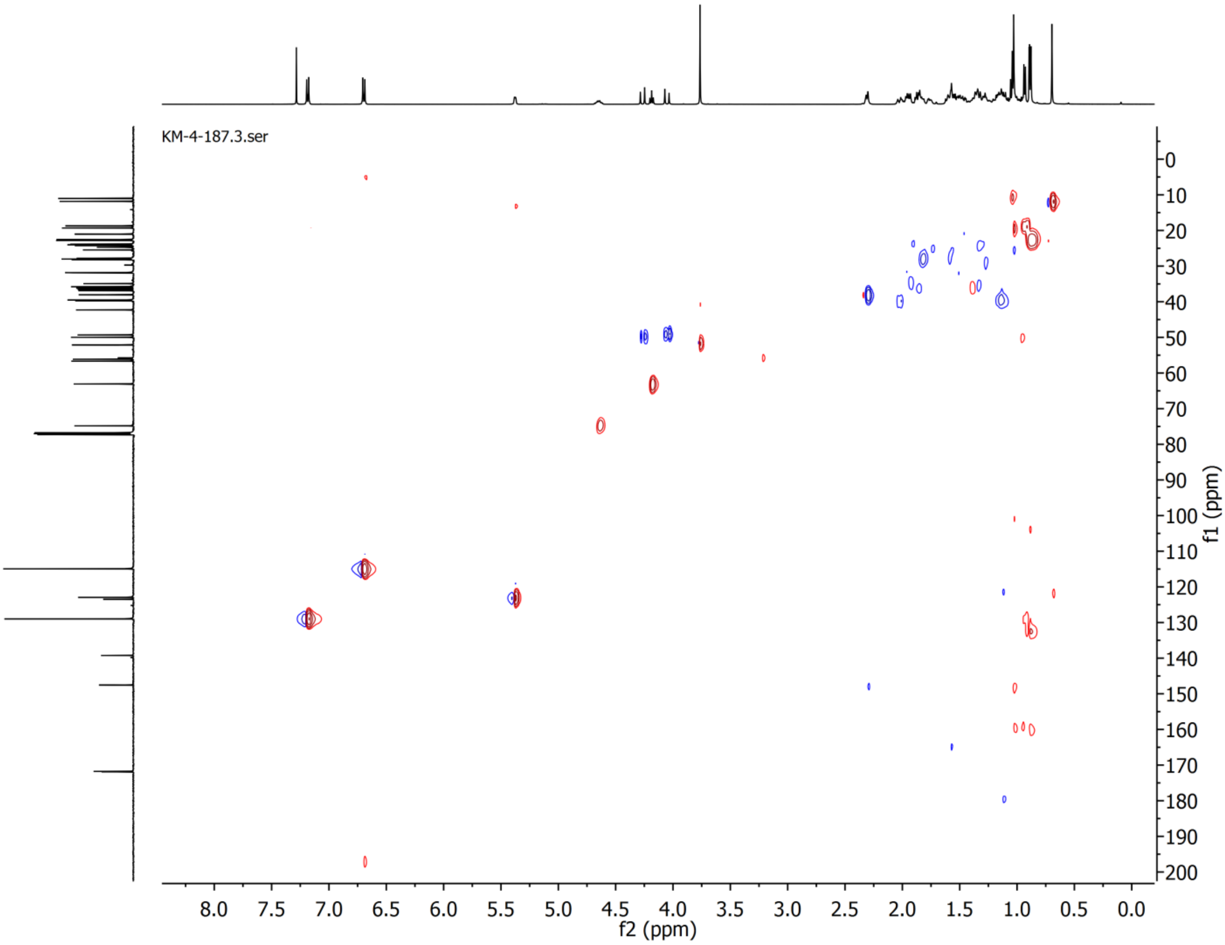

Supplementary Figure 125. HSQC NMR ($CDCl_3$) spectrum for 35

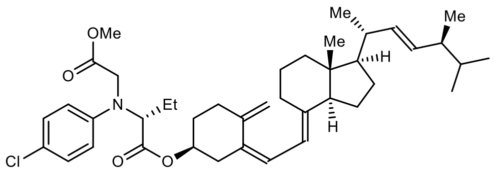

36

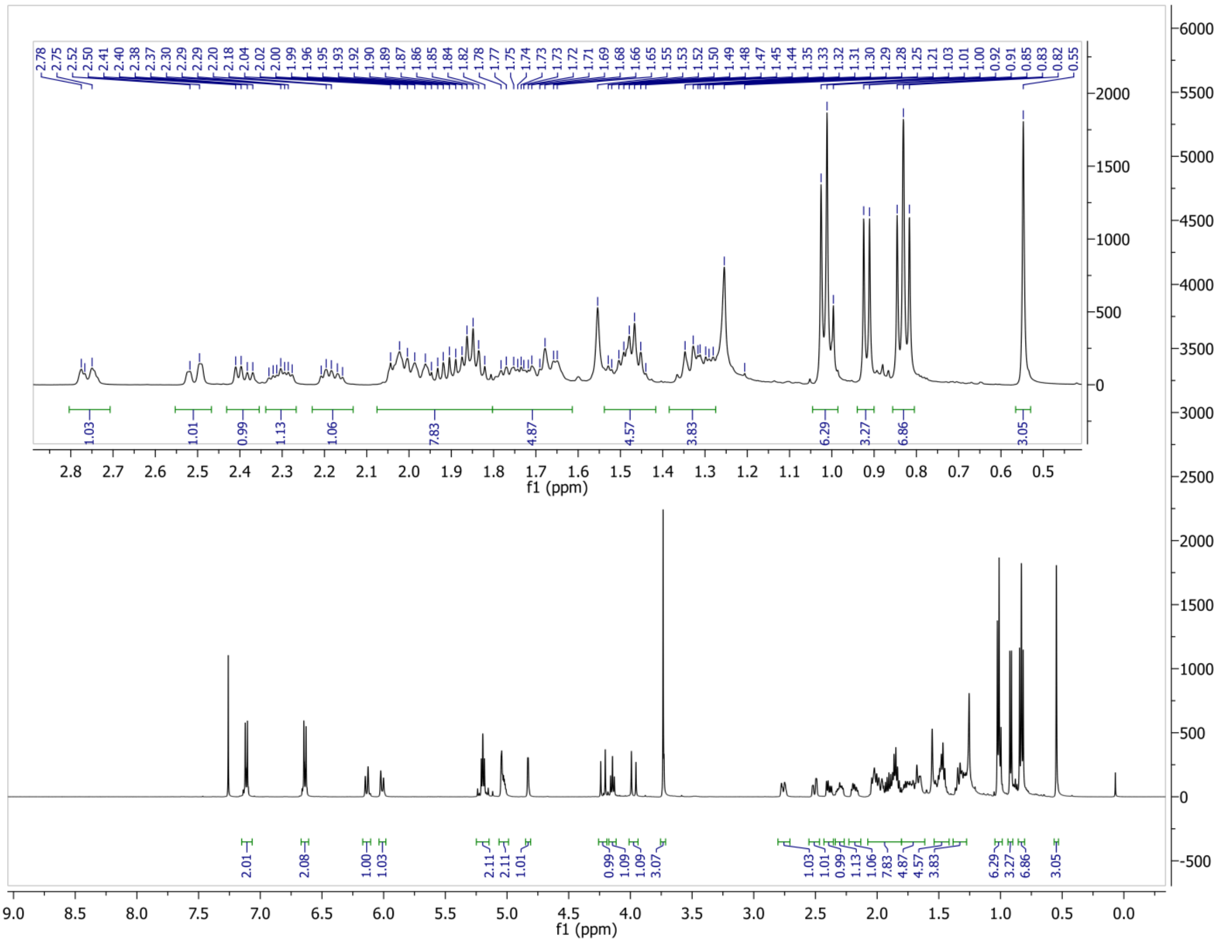

Supplementary Figure 126. ^{1}H NMR (500 MHz, $CDCl_3$) spectrum for 36

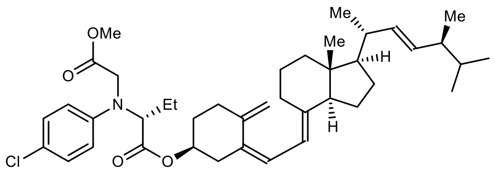

36

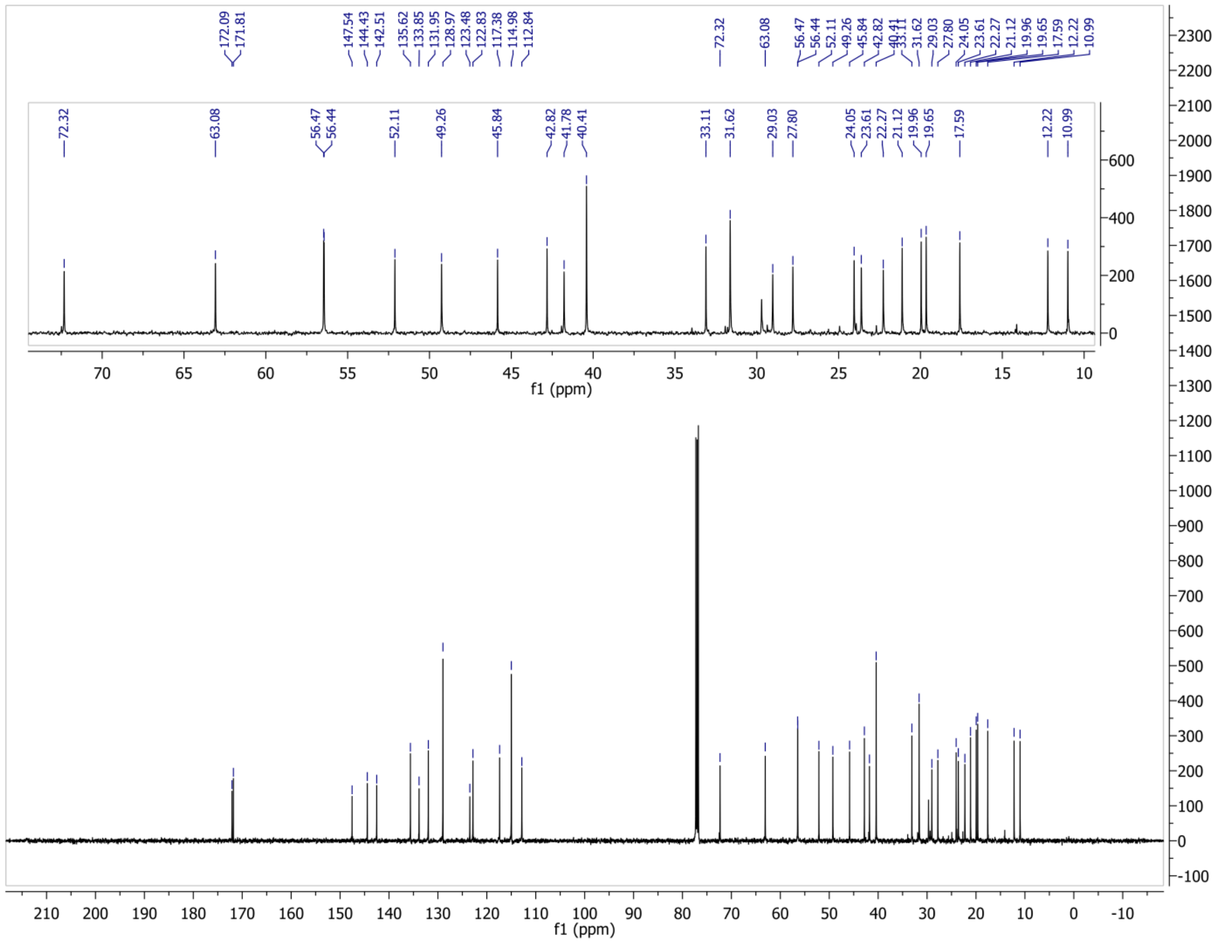

Supplementary Figure 127. ^{13}**C NMR (126 MHz, CDCl**$_3$**) spectrum for 36**

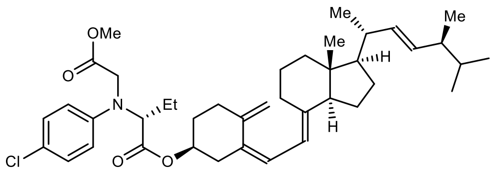

36

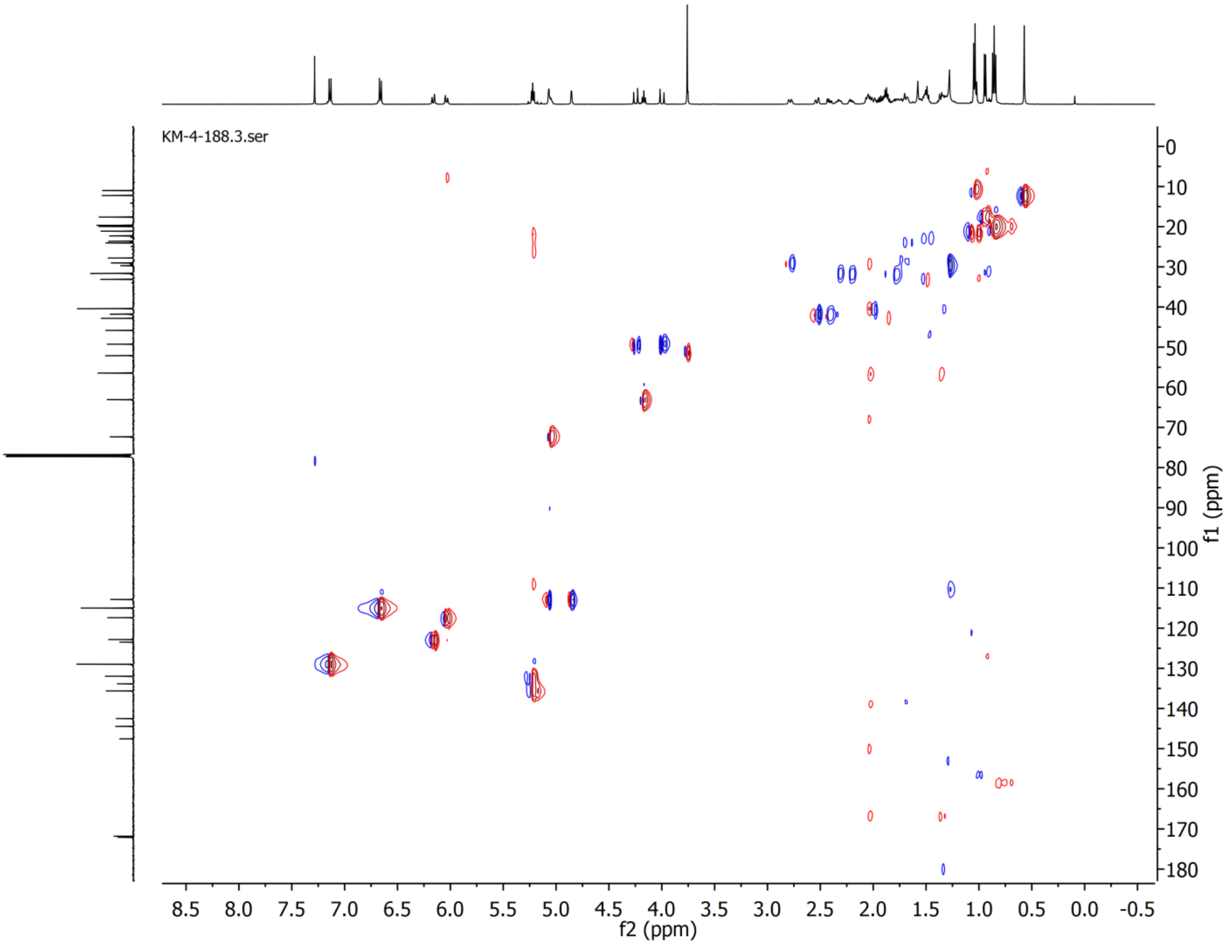

Supplementary Figure 128. HSQC NMR ($CDCl_3$) spectrum for 36

HPLC traces of racemic and chiral azetines 3

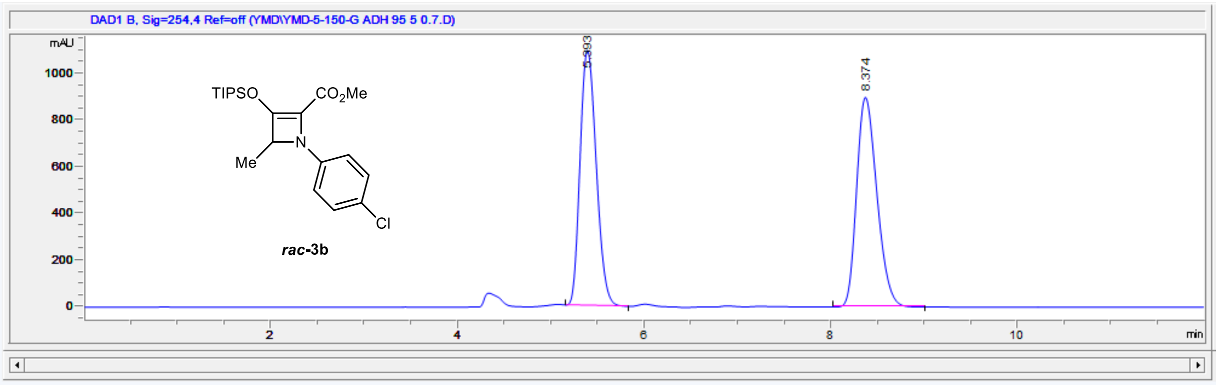

#	Time	Area	Height	Width	Area%	Symmetry
1	5.393	13218.3	1095.8	0.1907	49.281	0.786
2	8.374	13603.8	900.8	0.2362	50.719	0.726

Supplementary Figure 129. HPLC trace for methyl 1-(4-chlorophenyl)-4-methyl-3-[(triisopropylsilyl)oxy]-1,4-dihydroazete-2-carboxylate (*rac*-3b)

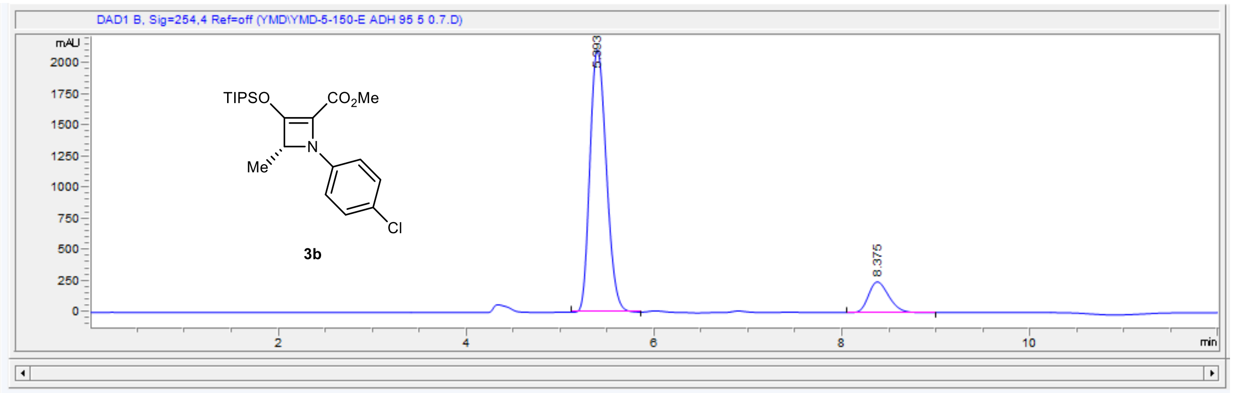

#	Time	Area	Height	Width	Area%	Symmetry
1	5.393	25662.9	2108.6	0.192	87.413	0.781
2	8.375	3695.4	246.8	0.2327	12.587	0.74

Supplementary Figure 130. HPLC trace for methyl (*R*)-1-(4-chlorophenyl)-4-methyl-3-[(triisopropylsilyl)oxy]-1,4-dihydroazete-2-carboxylate (3b)

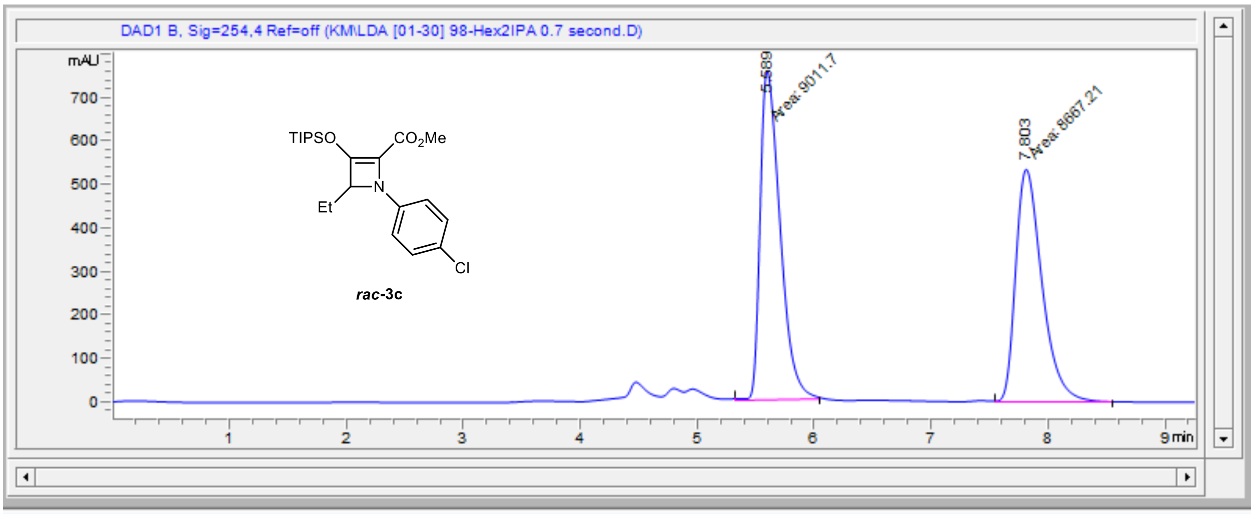

#	Time	Area	Height	Width	Area%	Symmetry
1	5.589	9011.7	761.1	0.1973	50.974	0.536
2	7.803	8667.2	541.6	0.2667	49.026	0.63

Supplementary Figure 131. HPLC trace for methyl 1-(4-chlorophenyl)-4-ethyl-3-[(triisopropylsilyl)oxy]-1,4-dihydroazete-2-carboxylate (*rac*-3c)

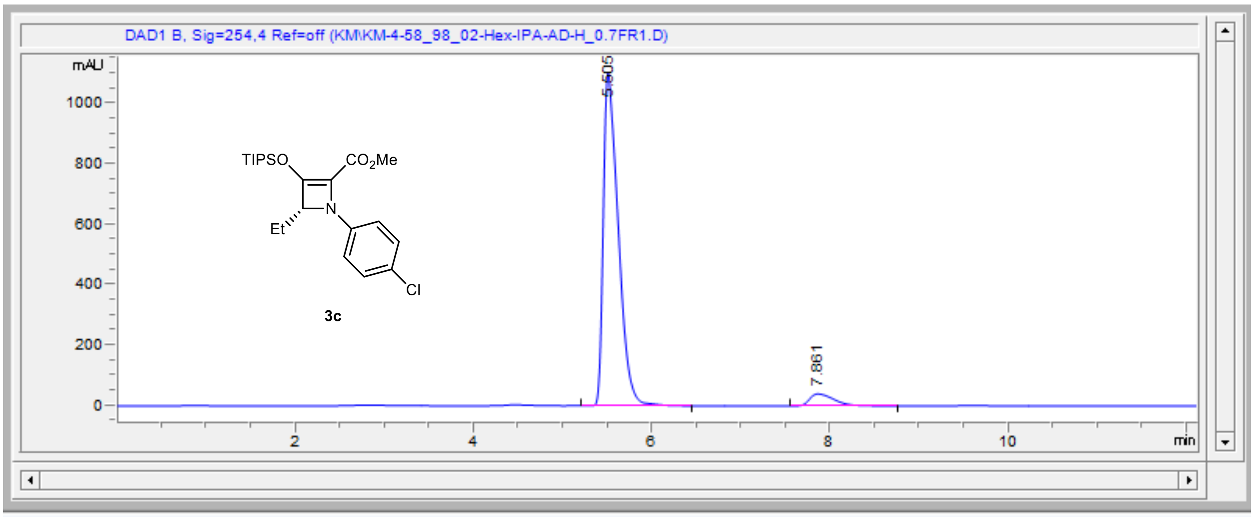

#	Time	Area	Height	Width	Area%	Symmetry
1	5.505	12727.5	1098.7	0.1852	94.967	0.436
2	7.861	675.5	39.5	0.3	5.033	0.495

Supplementary Figure 132. HPLC trace for methyl (*R*)-1-(4-chlorophenyl)-4-ethyl-3-[(triisopropylsilyl)-oxy]-1,4-dihydroazete-2-carboxylate (3c)

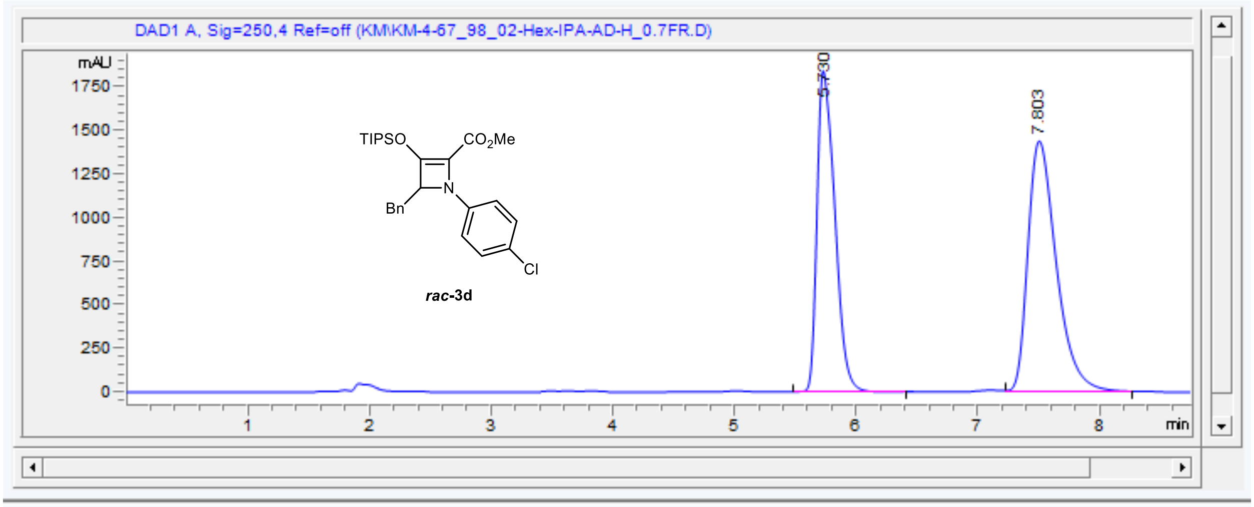

#	Time	Area	Height	Width	Area%	Symmetry
1	5.73	18857.1	1850.2	0.1662	50.466	0.536
2	7.803	18487.7	1425.6	0.2689	49.534	0.619

Supplementary Figure 133. HPLC trace for methyl 4-benzyl-1-(4-chlorophenyl)-3-[(triisopropylsilyl)-oxy]-1,4-dihydroazete-2-carboxylate (*rac*-3d)

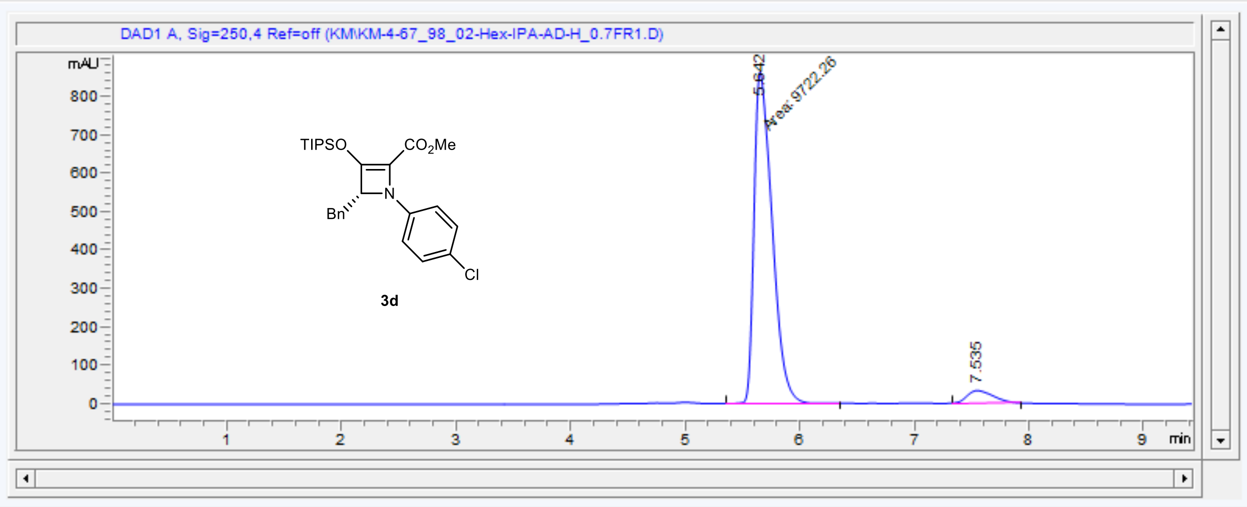

#	Time	Area	Height	Width	Area%	Symmetry
1	5.642	9722.3	869.5	0.1864	94.868	0.493
2	7.535	546.1	36.8	0.2922	5.132	0.615

Supplementary Figure 134. HPLC trace for methyl (*R*)-4-benzyl-1-(4-chlorophenyl)-3-[(triisopropylsilyl)-oxy]-1,4-dihydroazete-2-carboxylate (3d)

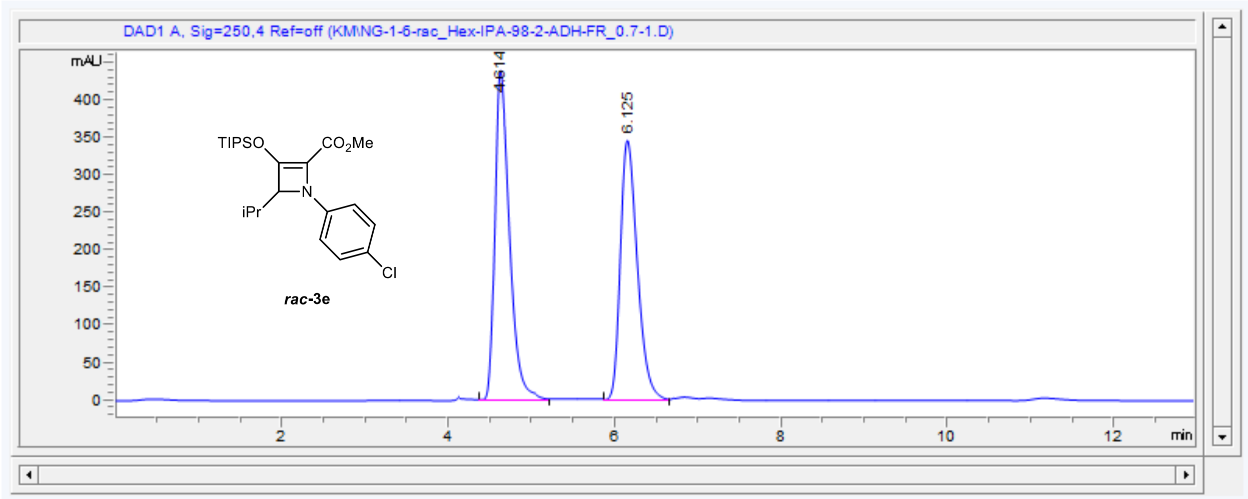

#	Time	Area	Height	Width	Area%	Symmetry
1	4.614	5678.4	442.5	0.2339	51.475	0.672
2	6.125	5357.2	342.1	0.2272	48.525	0.526

Supplementary Figure 135. HPLC trace for methyl 1-(4-chlorophenyl)-4-isopropyl-3-[(triisopropylsilyl)-oxy]-1,4-dihydroazete-2-carboxylate (*rac*-3e)

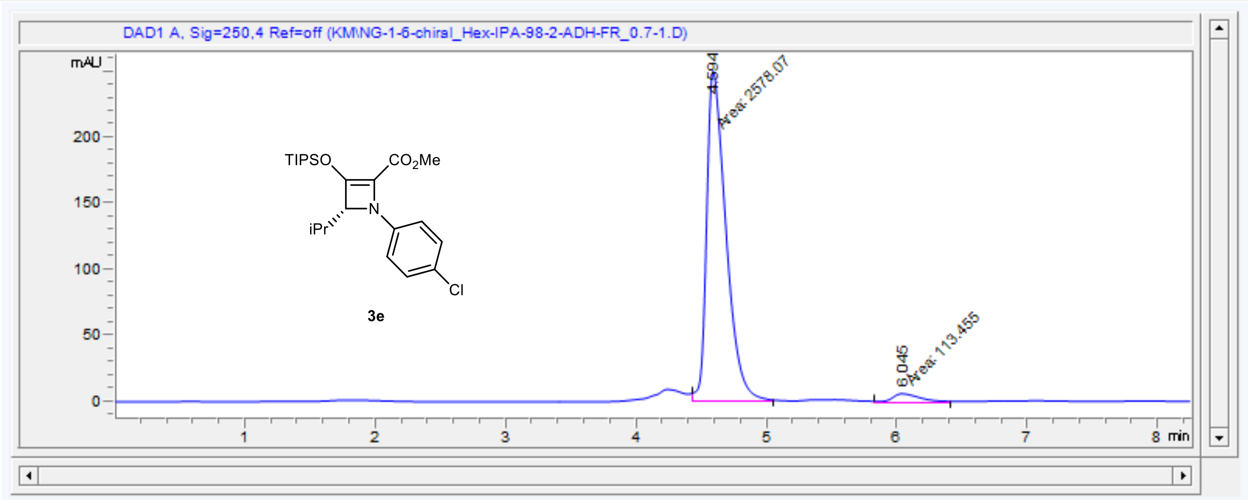

#	Time	Area	Height	Width	Area%	Symmetry
1	4.594	2578.1	250.9	0.1712	95.785	0.504
2	6.045	113.5	6.9	0.2748	4.215	0.554

Supplementary Figure 136. HPLC trace for methyl (*R*)-1-(4-chlorophenyl)-4-isopropyl-3-[(triisopropyl-silyl)oxy]-1,4-dihydroazete-2-carboxylate (3e)

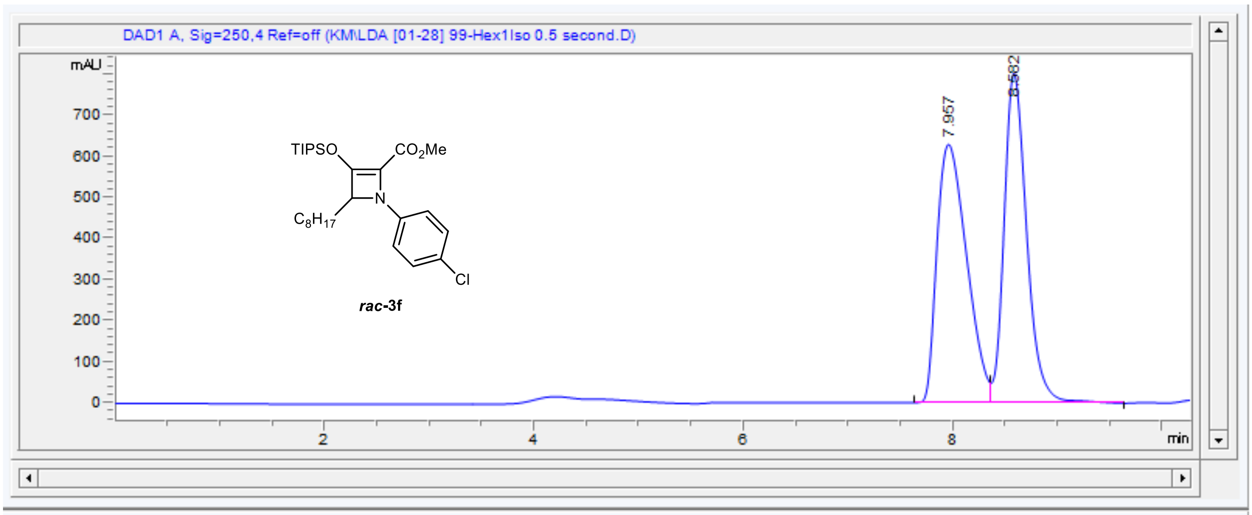

#	Time	Area	Height	Width	Area%	Symmetry
1	7.957	12391.9	628.7	0.314	50.704	0.602
2	8.582	12047.9	802.3	0.2312	49.296	0.708

Supplementary Figure 137. HPLC trace for methyl 1-(4-chlorophenyl)-4-octyl-3-[(triisopropylsilyl)oxy]-1,4-dihydroazete-2-carboxylate (*rac*-3f)

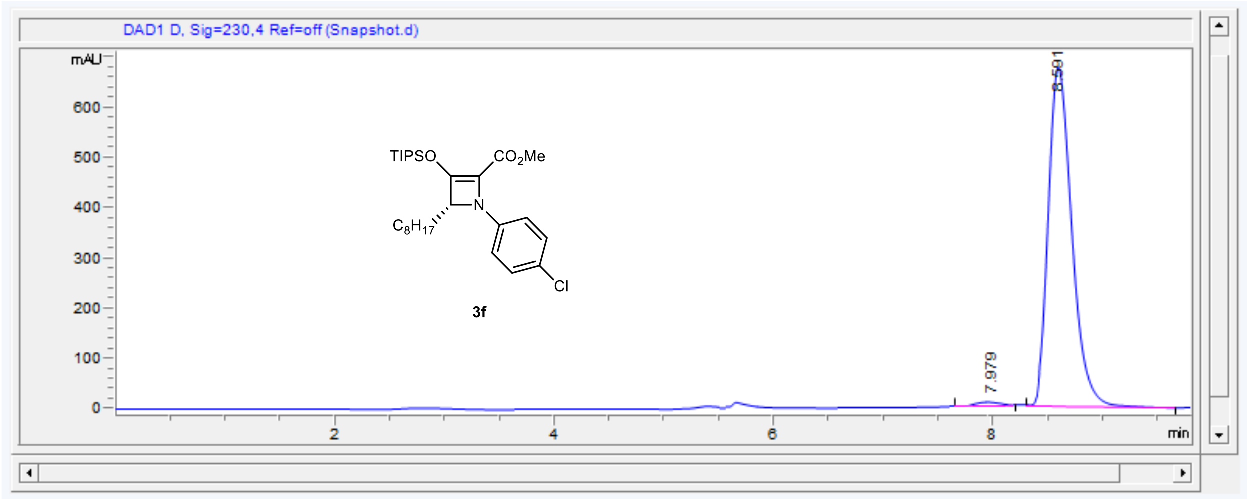

#	Time	Area	Height	Width	Area%	Symmetry
1	7.979	153.2	8.8	0.177	1.499	2.505
2	8.591	10269.8	675.7	0.2353	98.501	0.705

Supplementary Figure 138. HPLC trace for methyl (*R*)-1-(4-chlorophenyl)-4-octyl-3-[(triisopropylsilyl)-oxy]-1,4-dihydroazete-2-carboxylate (3f)

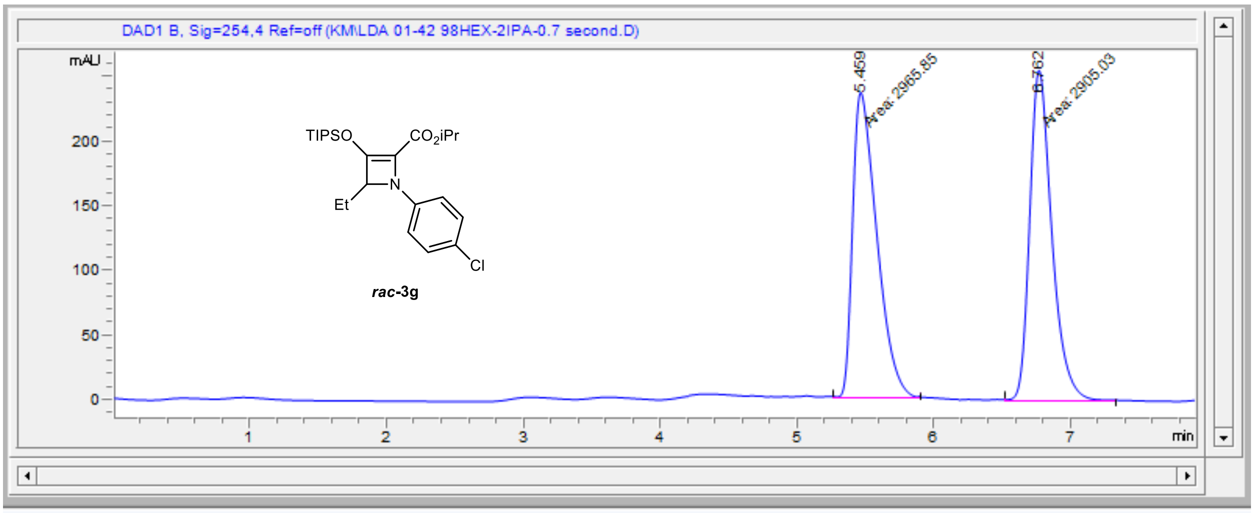

#	Time	Area	Height	Width	Area%	Symmetry
1	5.459	2965.8	238.5	0.2073	50.518	0.491
2	6.762	2905	256.9	0.1885	49.482	0.703

Supplementary Figure 139. HPLC trace for isopropyl 1-(4-chlorophenyl)-4-ethyl-3-[(triisopropylsilyl)-oxy]-1,4-dihydroazete-2-carboxylate (*rac*-3g)

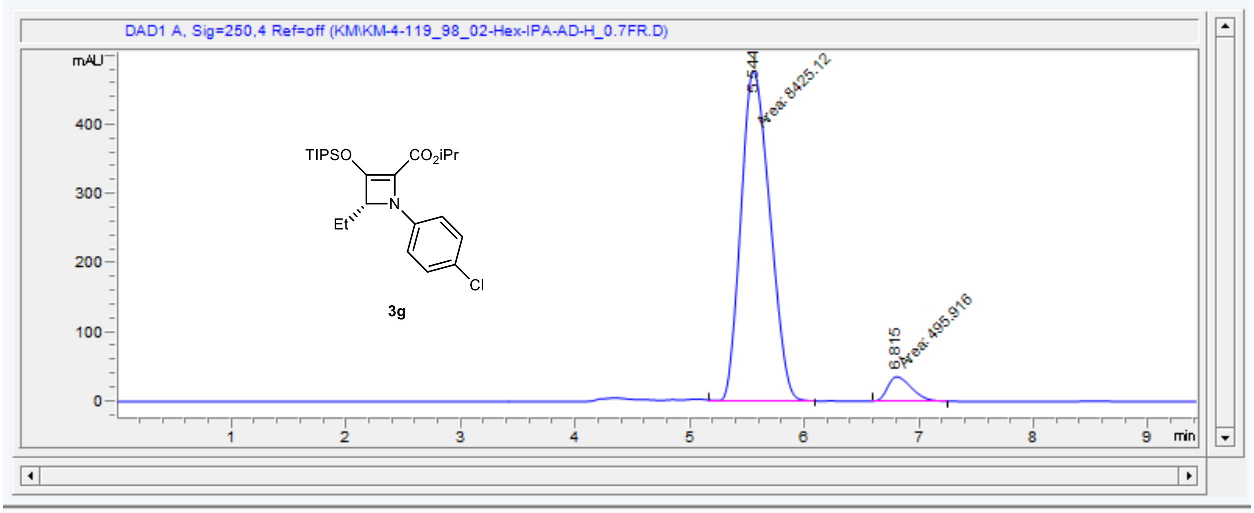

#	Time	Area	Height	Width	Area%	Symmetry
1	5.544	8425.1	480.6	0.2922	94.394	0.74
2	6.815	495.9	37.7	0.2636	5.606	0.589

Supplementary Figure 140. HPLC trace for isopropyl (*R*)-1-(4-chlorophenyl)-4-ethyl-3-[(triisopropyl-silyl)oxy]-1,4-dihydroazete-2-carboxylate (3g)

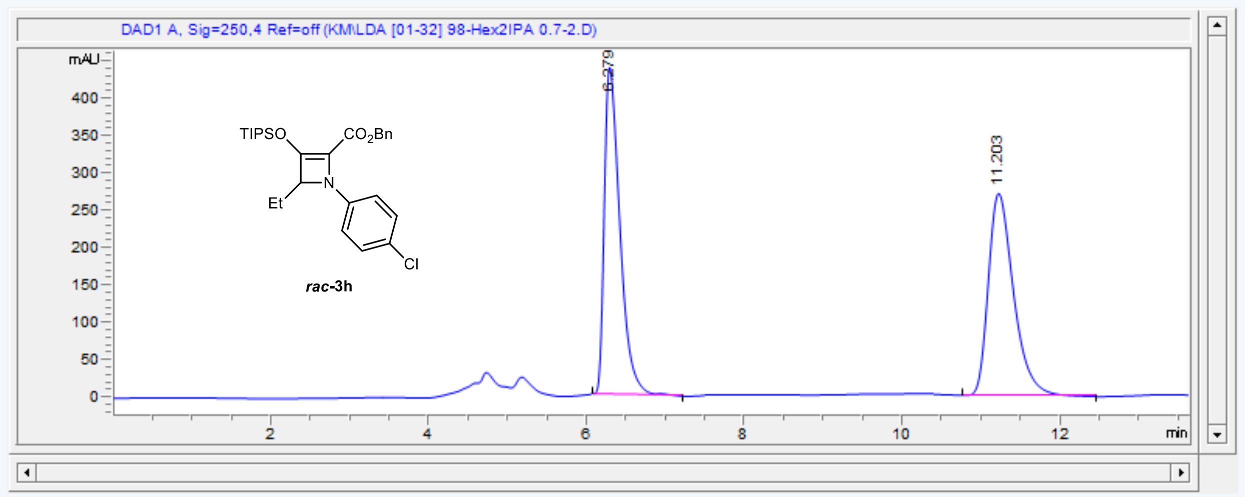

#	Time	Area	Height	Width	Area%	Symmetry
1	6.279	6011.2	436.3	0.2121	49.943	0.496
2	11.203	6025	270.7	0.3437	50.057	0.646

Supplementary Figure 141. HPLC trace for benzyl 1-(4-chlorophenyl)-4-ethyl-3-[(triisopropylsilyl)oxy]-1,4-dihydroazete-2-carboxylate (*rac*-3h)

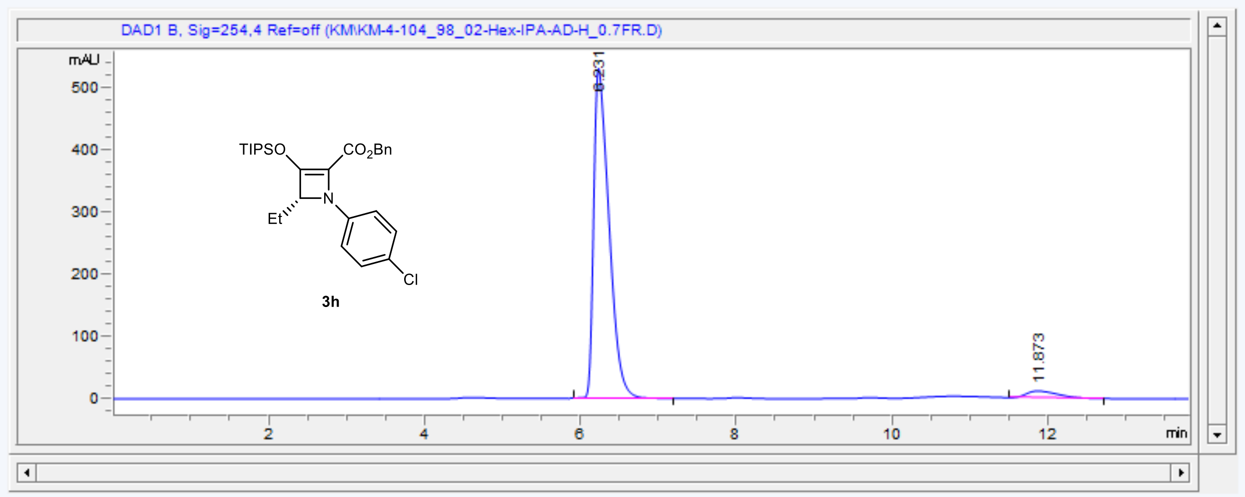

#	Time	Area	Height	Width	Area%	Symmetry
1	6.231	7257.3	531.2	0.2174	95.881	0.439
2	11.873	311.8	11.2	0.4242	4.119	0.545

Supplementary Figure 142. HPLC trace for benzyl (*R*)-1-(4-chlorophenyl)-4-ethyl-3-[(triisopropylsilyl)-oxy]-1,4-dihydroazete-2-carboxylate (3h)

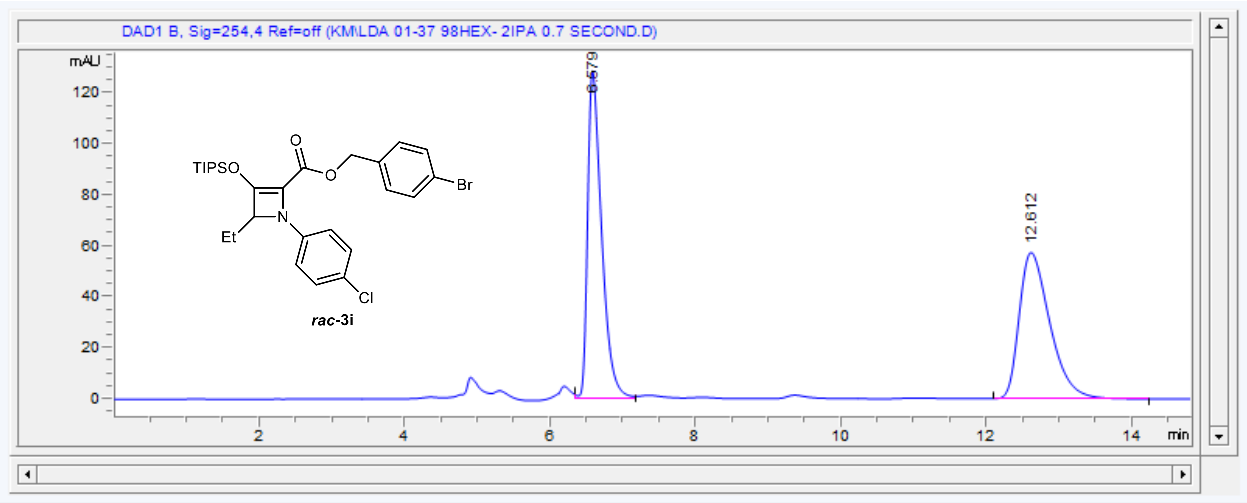

#	Time	Area	Height	Width	Area%	Symmetry
1	6.579	1719.8	129.1	0.222	50.555	0.527
2	12.612	1682.1	57.6	0.4489	49.445	0.594

Supplementary Figure 143. HPLC trace for 4-bromobenzyl 1-(4-chlorophenyl)-4-ethyl-3-[(triisopropyl-silyl)oxy]-1,4-dihydroazete-2-carboxylate (*rac*-3i)

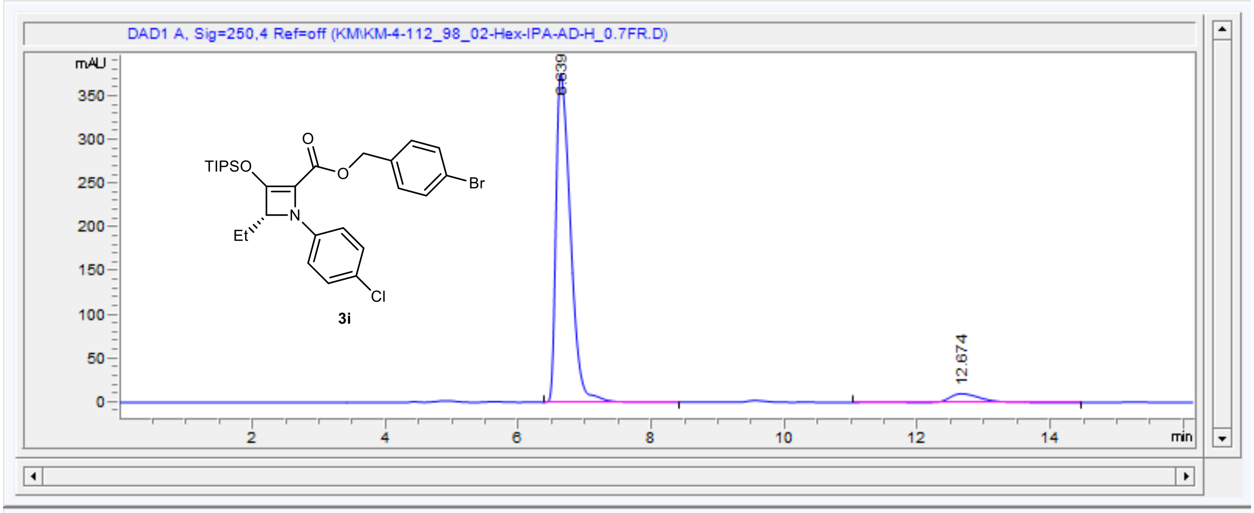

#	Time	Area	Height	Width	Area%	Symmetry
1	6.639	5711.6	378.1	0.2402	94.776	0.476
2	12.674	321.2	9.9	0.4904	5.224	0.697

Supplementary Figure 144. HPLC trace for 4-bromobenzyl (*R*)-1-(4-chlorophenyl)-4-ethyl-3-[(triisopropylsilyl)oxy]-1,4-dihydroazete-2-carboxylate (3i)

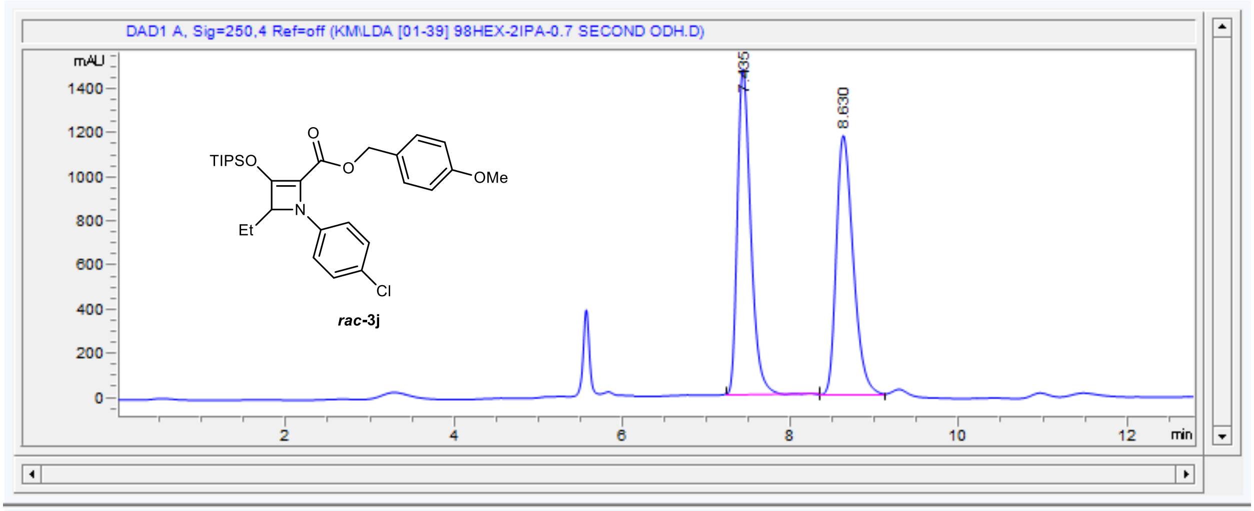

#	Time	Area	Height	Width	Area%	Symmetry
1	7.435	16502.9	1476	0.1736	49.536	0.668
2	8.63	16812	1182.9	0.2369	50.464	0.662

Supplementary Figure 145. HPLC trace for 4-methoxybenzyl 1-(4-chlorophenyl)-4-ethyl-3-[(triisopropylsilyl)oxy]-1,4-dihydroazete-2-carboxylate (*rac*-3j)

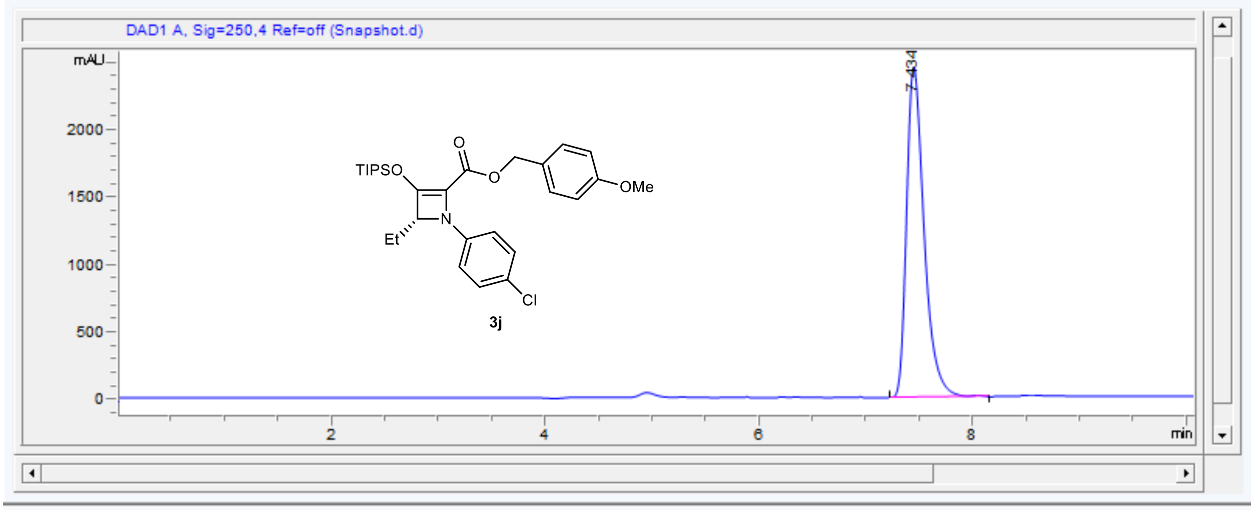

#	Time	Area	Height	Width	Area%	Symmetry
1	7.434	28264.2	2445.9	0.1736	100.000	0.634

Supplementary Figure 146. HPLC trace for 4-methoxybenzyl (*R*)-1-(4-chlorophenyl)-4-ethyl-3-[(triisopropylsilyl)oxy]-1,4-dihydroazete-2-carboxylate (3j)

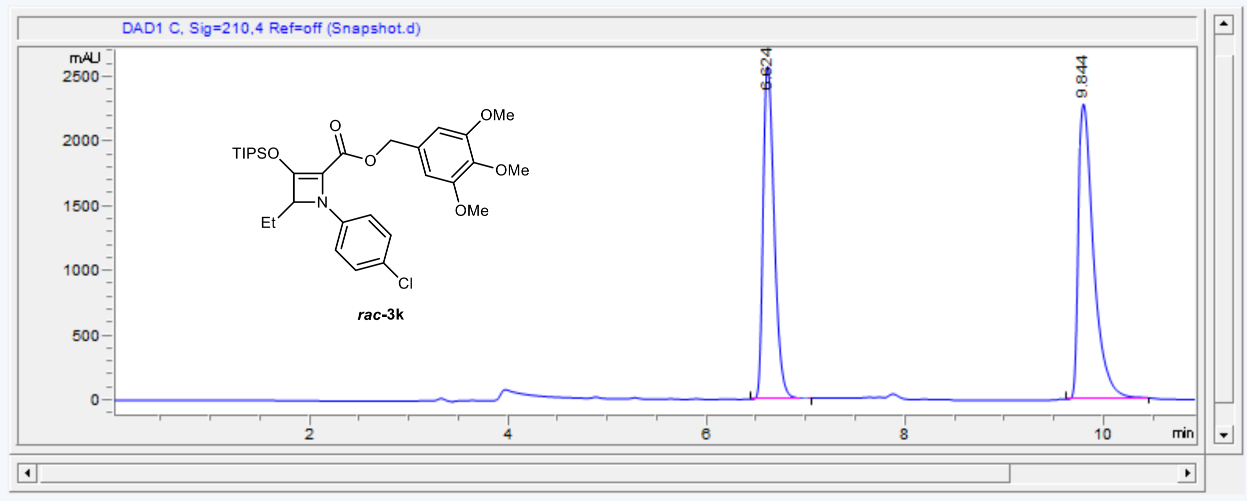

#	Time	Area	Height	Width	Area%	Symmetry
1	6.624	20093.3	2570.6	0.1226	50.683	0.593
2	9.844	19565.5	2392.3	0.1821	49.317	0.656

Supplementary Figure 147. HPLC trace for 3,4,5-trimethoxybenzyl 1-(4-chlorophenyl)-4-ethyl-3-[(triisopropylsilyl)oxy]-1,4-dihydroazete-2-carboxylate (*rac*-3k)

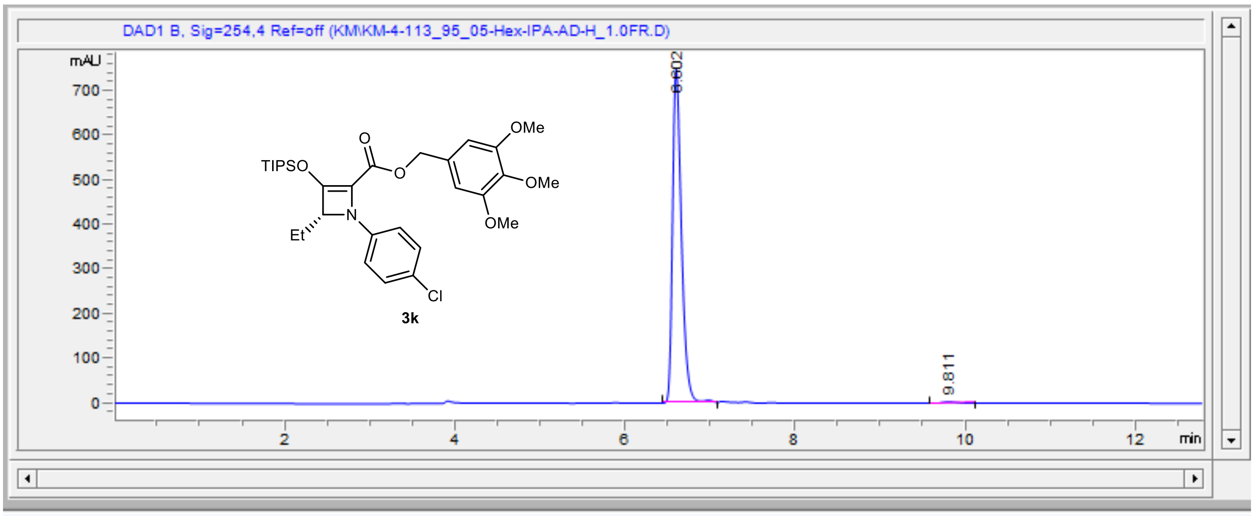

#	Time	Area	Height	Width	Area%	Symmetry
1	6.602	5437	748	0.1102	99.050	0.633
2	9.811	35.6	3.2	0.1704	0.950	0.819

Supplementary Figure 148. HPLC trace for 3,4,5-trimethoxybenzyl (*R*)-1-(4-chlorophenyl)-4-ethyl-3-[(triisopropylsilyl)oxy]-1,4-dihydroazete-2-carboxylate (3k)

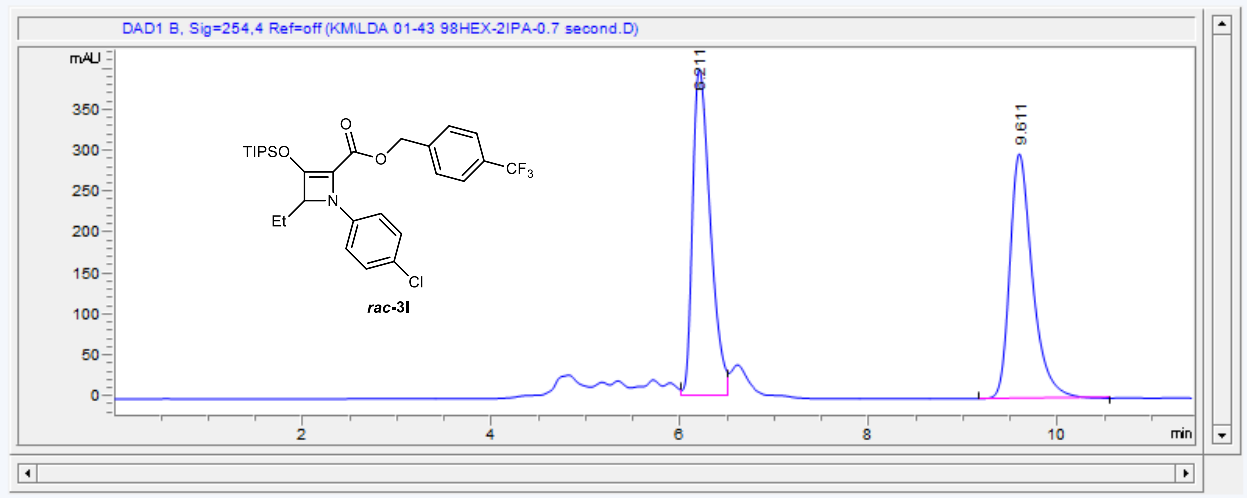

#	Time	Area	Height	Width	Area%	Symmetry
1	6.211	5259.2	401.9	0.2181	50.975	0.599
2	9.611	5057.9	301.1	0.254	49.025	0.67

Supplementary Figure 149. HPLC trace for 4-(trifluoromethyl)benzyl 1-(4-chlorophenyl)-4-ethyl-3-[(triisopropylsilyl)oxy]-1,4-dihydroazete-2-carboxylate (*rac*-3l)

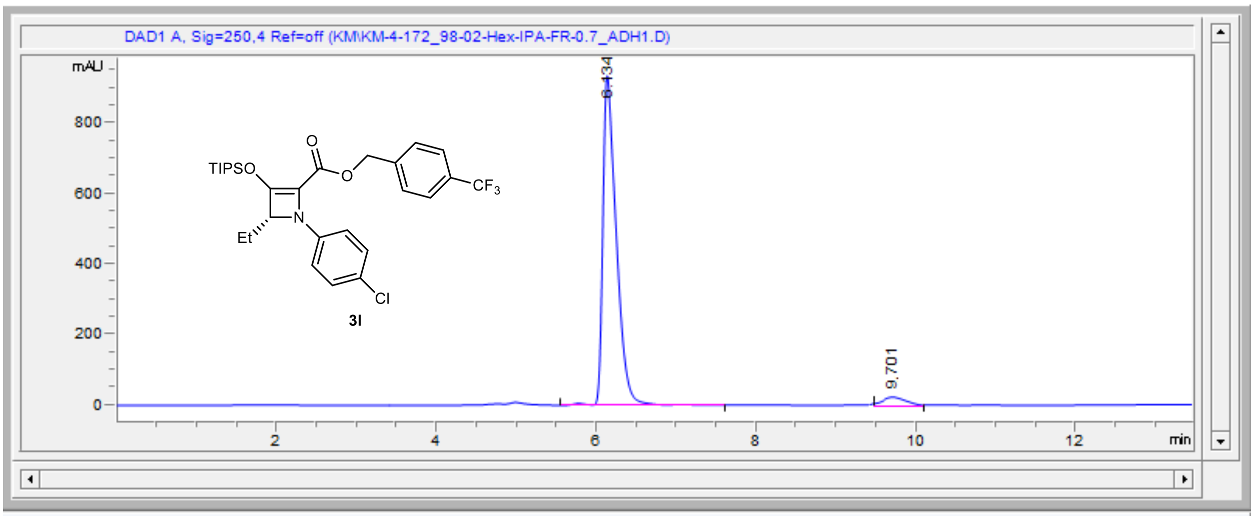

#	Time	Area	Height	Width	Area%	Symmetry
1	6.134	10915.1	934.5	0.1672	93.688	0.482
2	9.701	735.4	30.8	0.3978	6.312	0.611

Supplementary Figure 150. HPLC trace for 4-(trifluoromethyl)benzyl (*R*)-1-(4-chlorophenyl)-4-ethyl-3-[(triisopropylsilyl)oxy]-1,4-dihydroazete-2-carboxylate (3l)

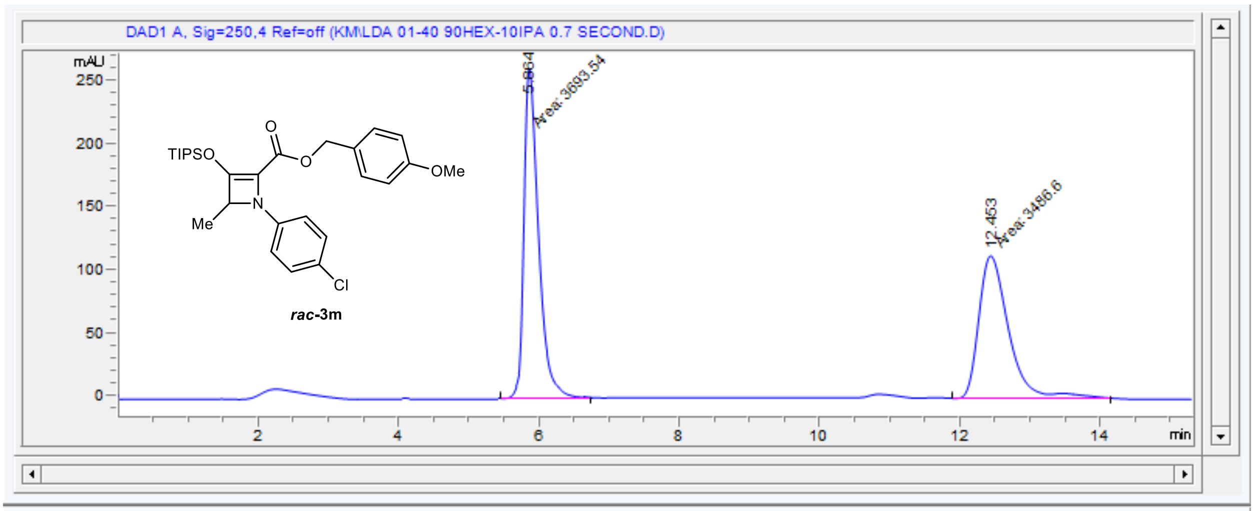

#	Time	Area	Height	Width	Area%	Symmetry
1	5.864	3693.5	262.4	0.2346	51.441	0.607
2	12.453	3486.6	113.6	0.5116	48.559	0.619

Supplementary Figure 151. HPLC trace for 4-methoxybenzyl 1-(4-chlorophenyl)-4-methyl-3-[(triisopropylsilyl)oxy]-1,4-dihydroazete-2-carboxylate (*rac*-3m)

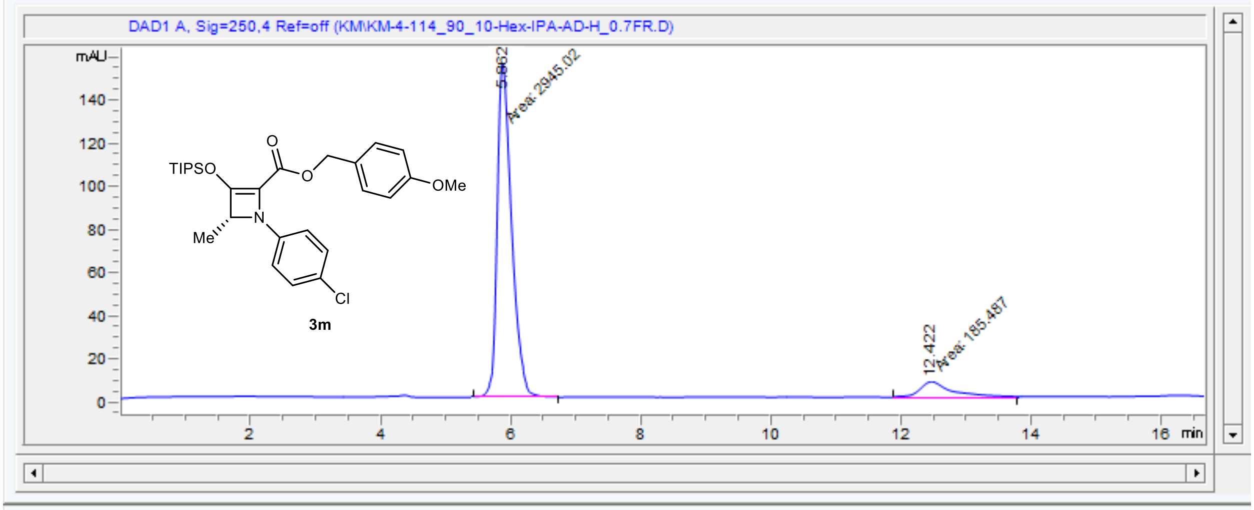

#	Time	Area	Height	Width	Area%	Symmetry
1	5.862	2945	154.2	0.3183	94.180	0.575
2	12.422	185.5	8.7	0.4089	5.820	0.623

Supplementary Figure 152. HPLC trace for 4-methoxybenzyl (*R*)-1-(4-chlorophenyl)-4-methyl-3-[(triisopropylsilyl)oxy]-1,4-dihydroazete-2-carboxylate (3m)

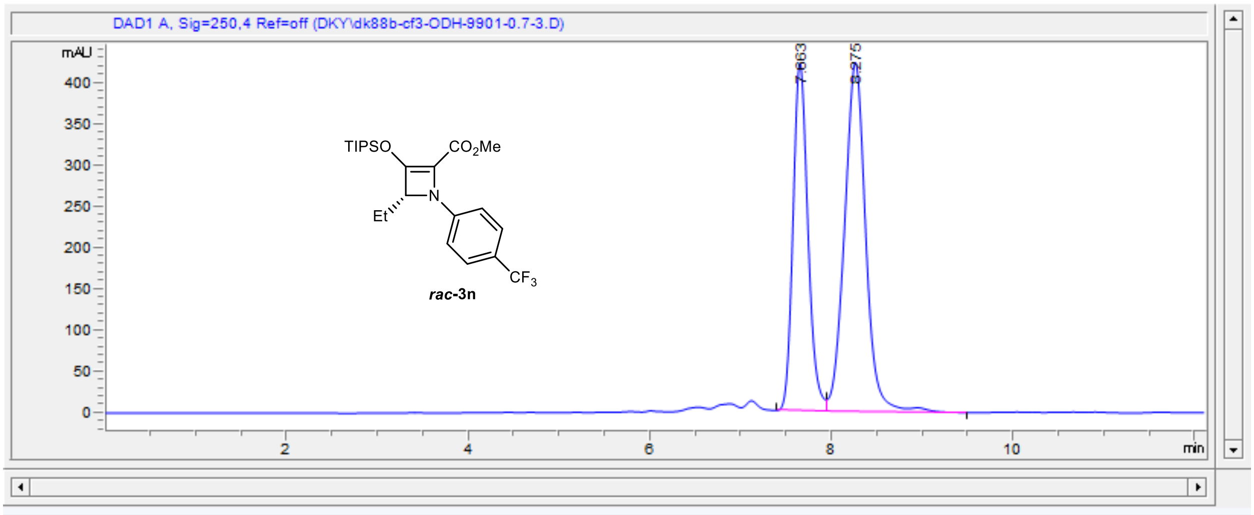

#	Time	Area	Height	Width	Area%	Symmetry
1	7.663	6406.8	422.3	0.1773	48.172	0.819
2	8.275	6893.1	423.4	0.2446	51.828	0.992

Supplementary Figure 153. HPLC trace for methyl 4-ethyl-1-[4-(trifluoromethyl)phenyl]-3-[(triisopropylsilyl)oxy]-1,4-dihydroazete-2-carboxylate (*rac*-3n)

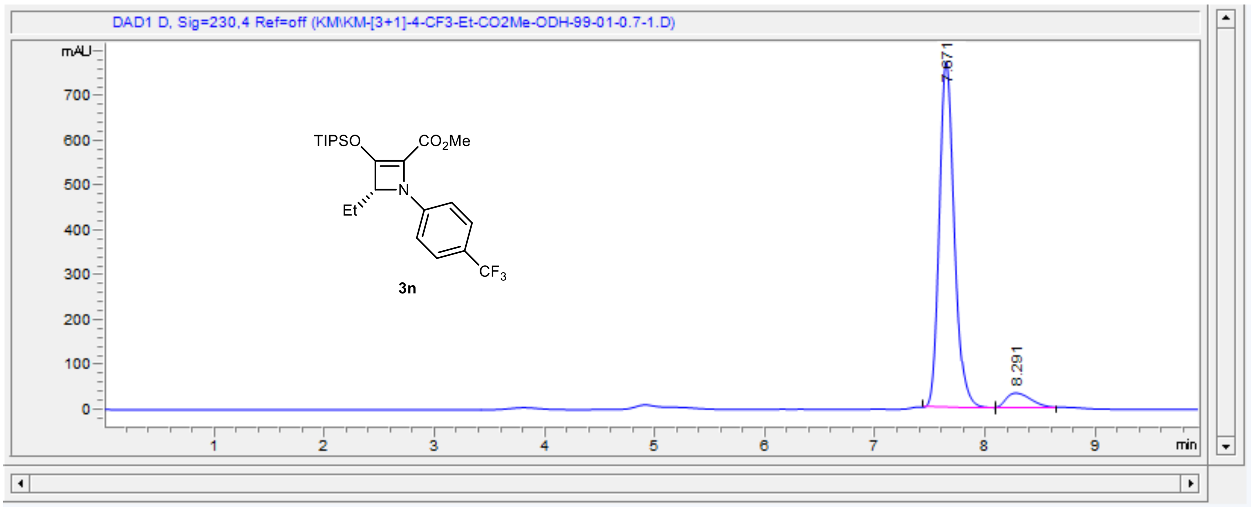

#	Time	Area	Height	Width	Area%	Symmetry
1	7.671	6171.1	778.8	0.1321	95.570	0.72
2	8.291	286.3	35.7	0.0912	4.430	0.77

Supplementary Figure 154. HPLC trace for methyl (*R*)-4-ethyl-1-[4-(trifluoromethyl)phenyl]-3-[(triisopropylsilyl)oxy]-1,4-dihydroazete-2-carboxylate (3n)

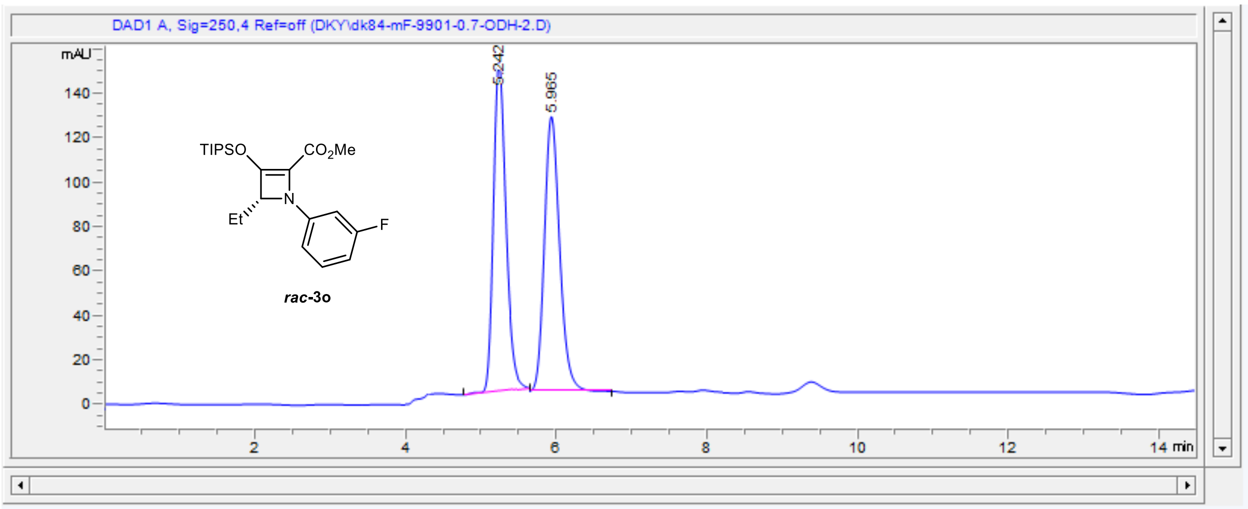

#	Time	Area	Height	Width	Area%	Symmetry
1	5.242	1742.5	147.5	0.1799	50.031	0.736
2	5.965	1740.3	123.2	0.2168	49.969	0.793

Supplementary Figure 155. HPLC trace for methyl 1-(3-fluorophenyl)-4-ethyl-3-[(triisopropylsilyl)oxy]-1,4-dihydroazete-2-carboxylate (*rac*-3o)

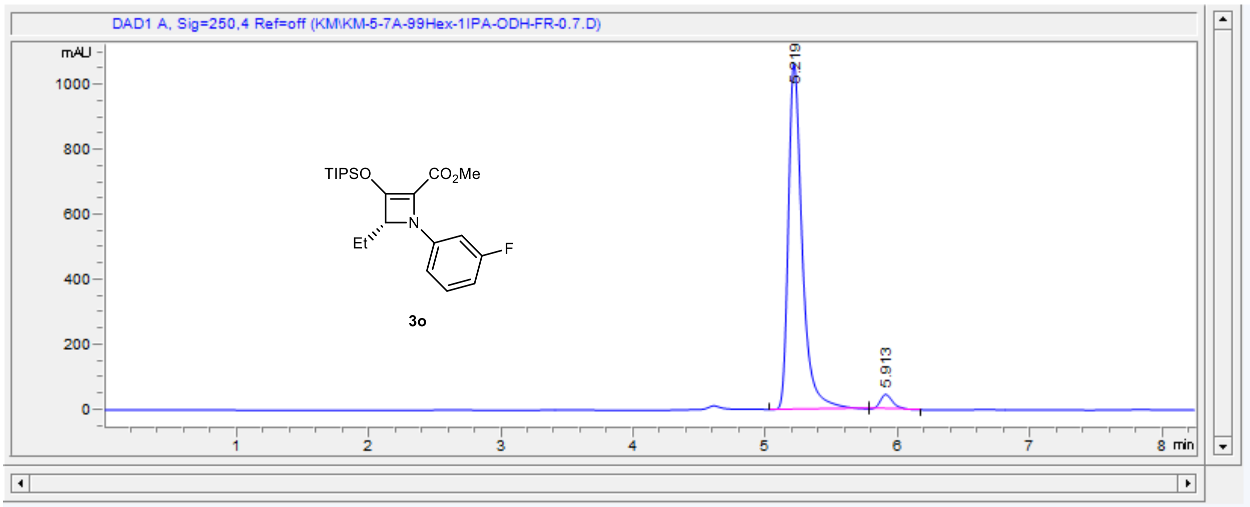

#	Time	Area	Height	Width	Area%	Symmetry
1	5.219	7796.4	1070	0.109	95.743	0.633
2	5.913	346.7	45	0.102	4.257	0.704

Supplementary Figure 156. HPLC trace for methyl (*R*)-1-(3-fluorophenyl)-4-ethyl-3-[(triisopropylsilyl)-oxy]-1,4-dihydroazete-2-carboxylate (3o)

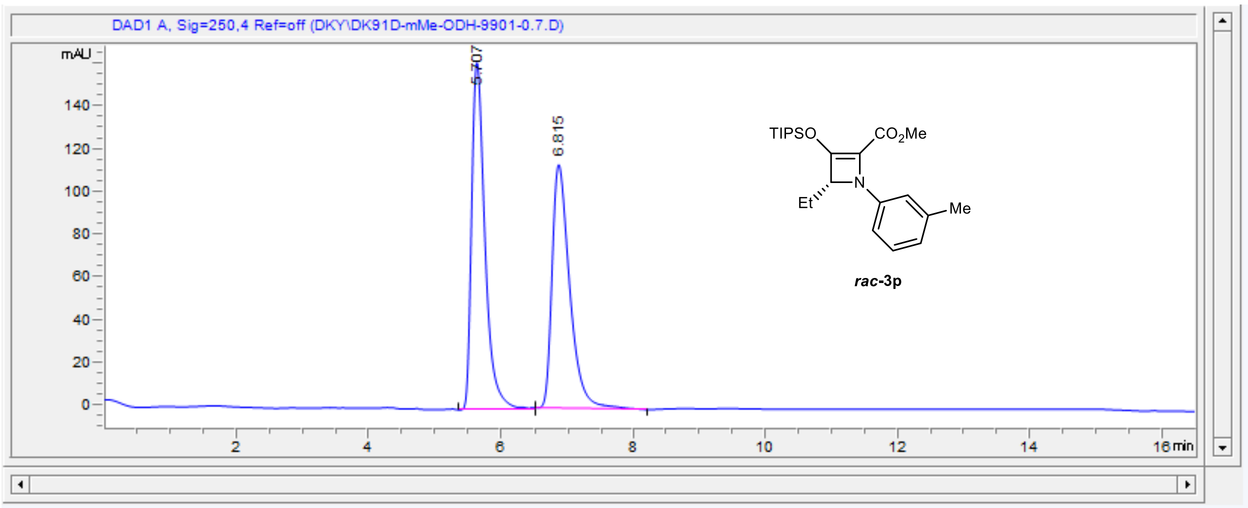

#	Time	Area	Height	Width	Area%	Symmetry
1	5.707	2131.3	161.4	0.1998	50.033	0.715
2	6.815	2128.5	114.4	0.281	49.967	0.557

Supplementary Figure 157. HPLC trace for methyl 4-ethyl-1-(3-methylphenyl)-3-[(triisopropylsilyl)oxy]-1,4-dihydroazete-2-carboxylate (*rac*-3p)

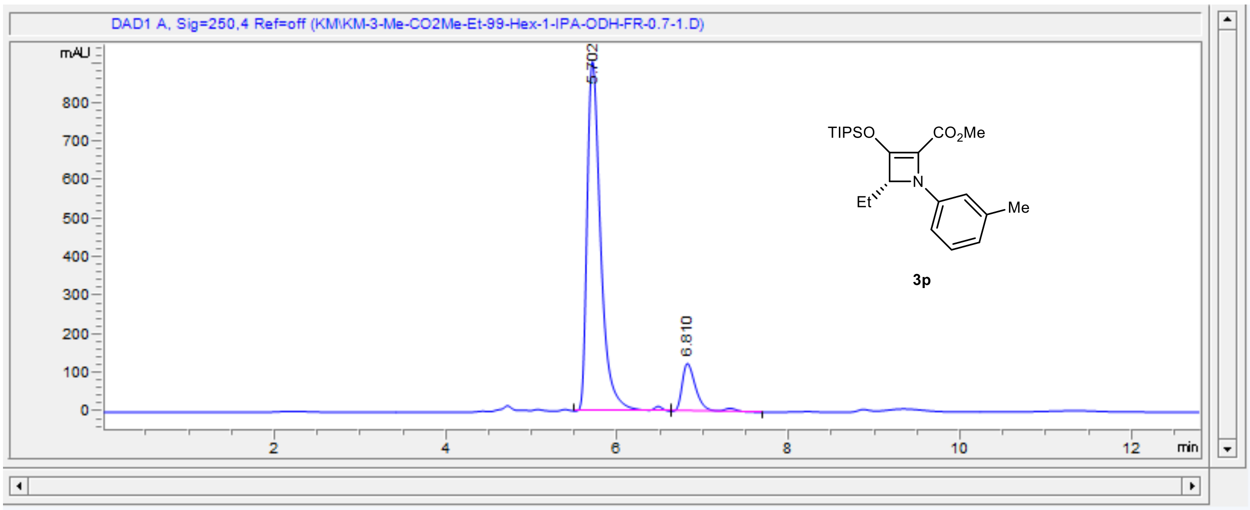

#	Time	Area	Height	Width	Area%	Symmetry
1	5.702	10010.3	904.6	0.1683	87.002	0.597
2	6.81	1442.9	124	0.1648	12.998	0.606

Supplementary Figure 158. HPLC trace for methyl (*R*)-4-ethyl-1-(3-methylphenyl)-3-[(triisopropylsilyl)-oxy]-1,4-dihydroazete-2-carboxylate (3p)

HPLC traces of racemic and chiral ring opened products (selected examples)

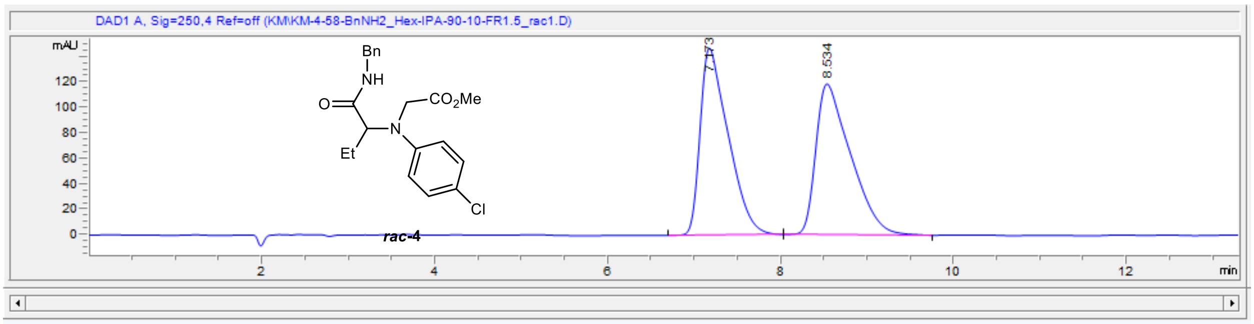

#	Time	Area	Height	Width	Area%	Symmetry
1	7.173	3296.9	146.6	0.3203	49.953	0.469
2	8.534	3303.1	118.3	0.3998	50.047	0.449

Supplementary Figure 159. HPLC trace for methyl *N*-[1-(benzylamino)-1-oxobutan-2-yl]-*N*-(4-chloro-phenyl)glycinate (*rac*-4)

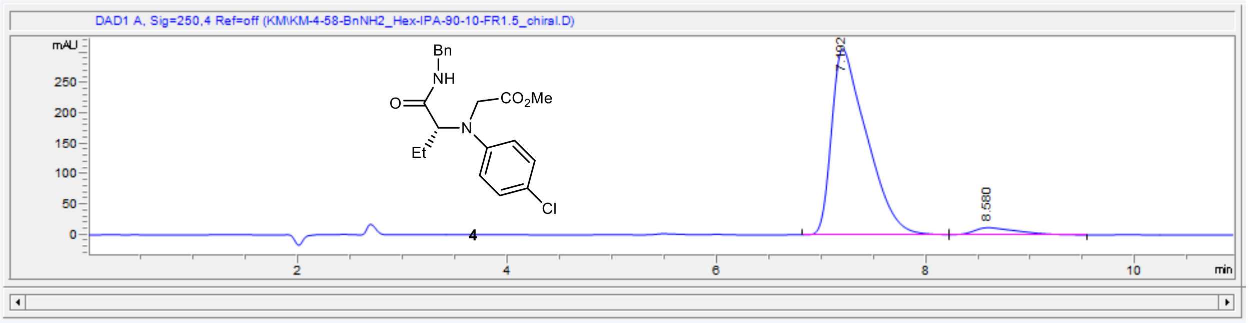

#	Time	Area	Height	Width	Area%	Symmetry
1	7.192	7139.9	307.2	0.3292	95.235	0.441
2	8.58	333.7	11.7	0.4028	4.765	0.443

Supplementary Figure 160. HPLC trace for methyl (*R*)-*N*-[1-(benzylamino)-1-oxobutan-2-yl]-*N*-(4-chlo-rophenyl)glycinate (4)

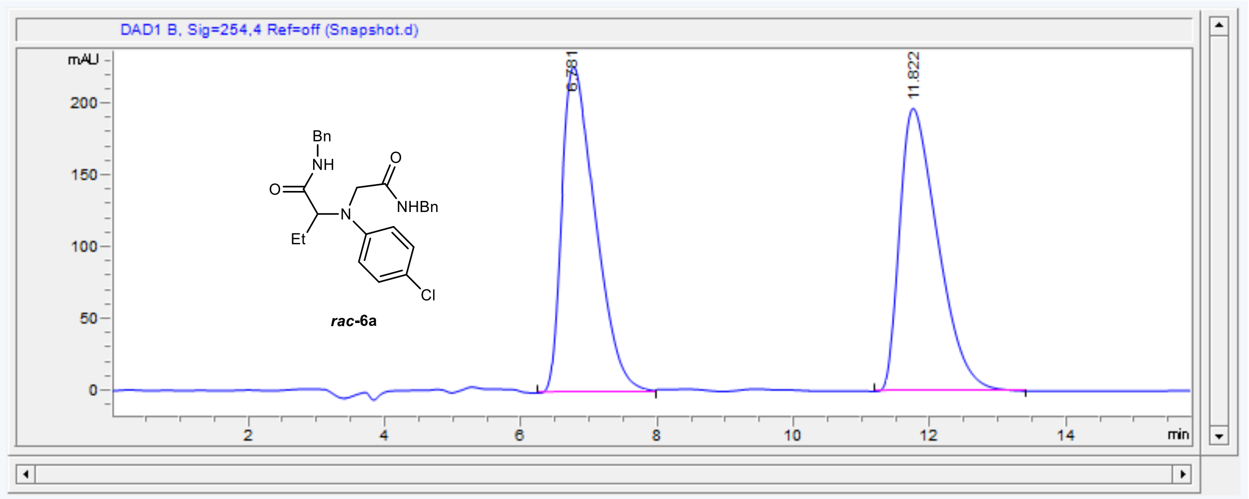

#	Time	Area	Height	Width	Area%	Symmetry
1	6.781	7831.2	224.6	0.5387	50.473	0.505
2	11.822	7684.4	196.4	0.5984	49.527	0.52

Supplementary Figure 161. HPLC trace for *N*-benzyl-2-([2-(benzylamino)-2-oxoethyl](4-chlorophenyl)-amino)butanamide (*rac*-6a)

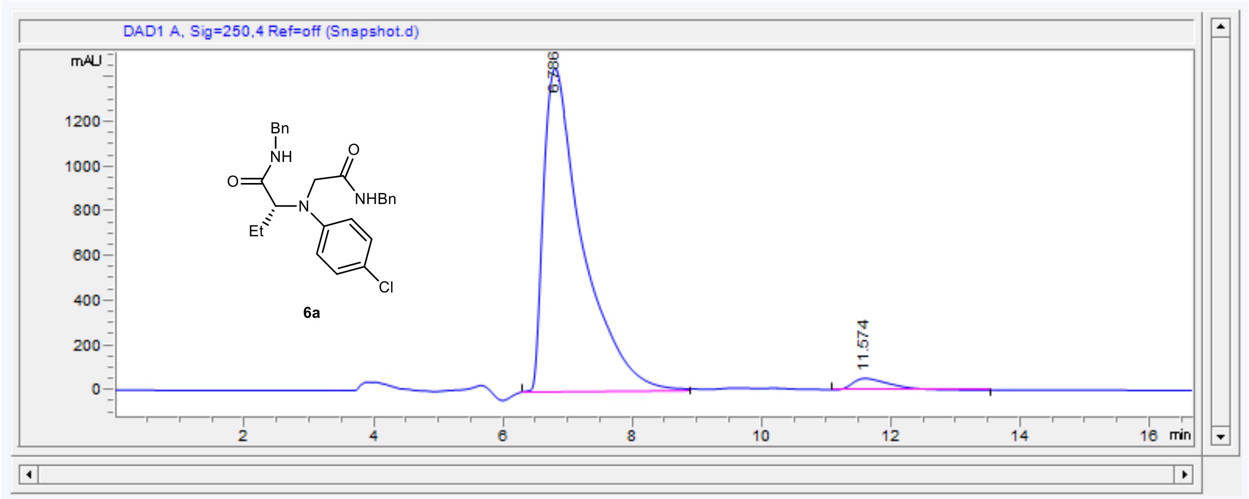

#	Time	Area	Height	Width	Area%	Symmetry
1	6.786	60480.2	1443.7	0.6982	94.899	0.399
2	11.574	3129.1	51.1	0.6224	5.101	0.439

Supplementary Figure 162. HPLC trace for (*R*)-*N*-benzyl-2-([2-(benzylamino)-2-oxoethyl](4-chloro-phenyl)amino)butanamide (6a)

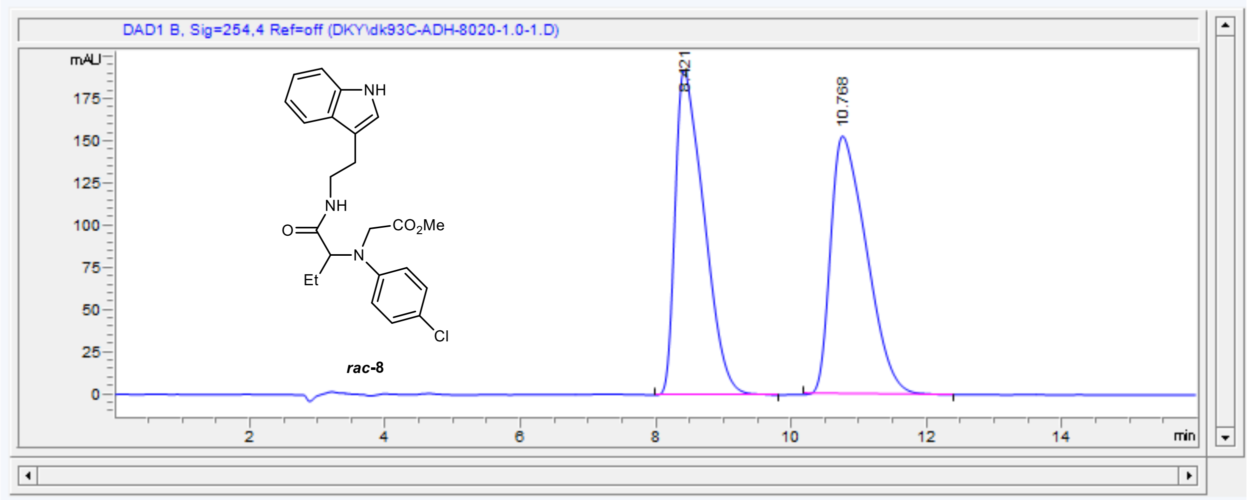

#	Time	Area	Height	Width	Area%	Symmetry
1	8.421	5730.3	193.8	0.4849	50.138	0.427
2	10.768	5698.8	154	0.5997	49.862	0.479

Supplementary Figure 163. HPLC trace for methyl *N*-(1-([2-(1*H*-indol-3-yl)ethyl]amino)-1-oxobutan-2-yl)-*N*-(4-chlorophenyl)glycinate (*rac*-8)

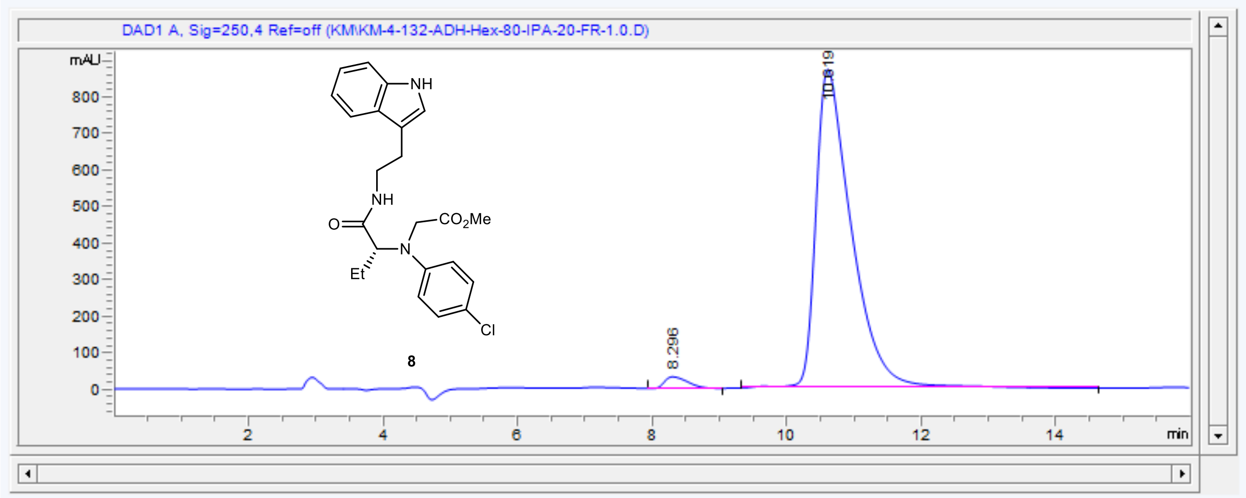

#	Time	Area	Height	Width	Area%	Symmetry
1	8.296	1765.8	42.7	0.3008	5.078	0.499
2	10.619	32371.3	874.2	0.5387	94.922	0.47

Supplementary Figure 164. HPLC trace for methyl (*R*)-*N*-(1-([2-(1*H*-indol-3-yl)ethyl]amino)-1-oxobutan-2-yl)-*N*-(4-chlorophenyl)glycinate (8)

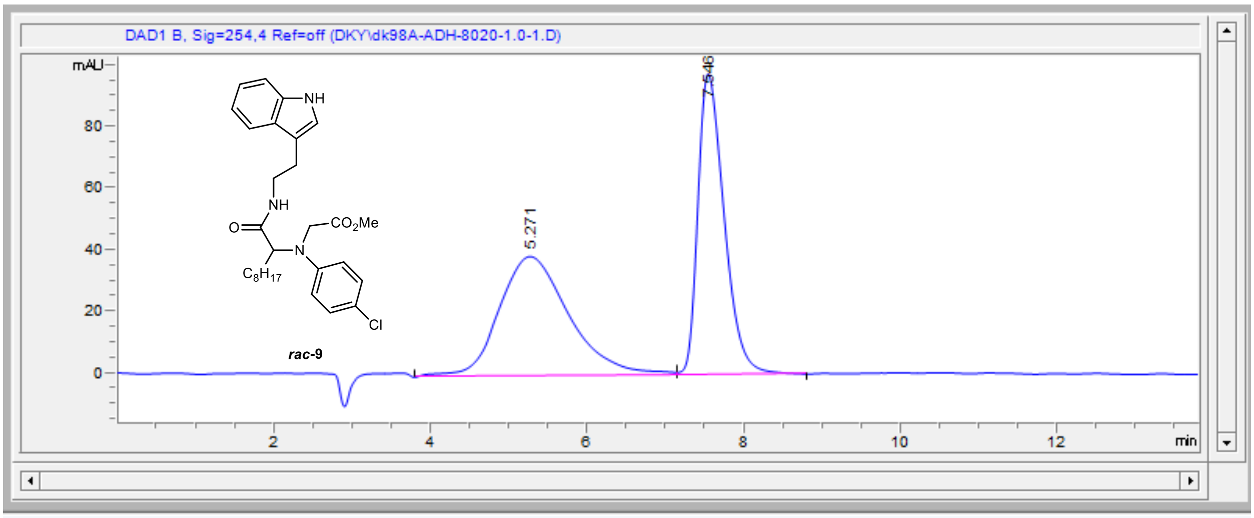

#	Time	Area	Height	Width	Area%	Symmetry
1	5.271	2443.8	38.9	0.9485	51.673	0.77
2	7.546	2285.6	97.7	0.3628	48.327	0.596

Supplementary Figure 165. HPLC trace for methyl *N*-(1-[(2-(1*H*-indol-3-yl)ethyl]amino)-1-oxodecan-2-yl)-*N*-(4-chlorophenyl)glycinate (*rac*-9)

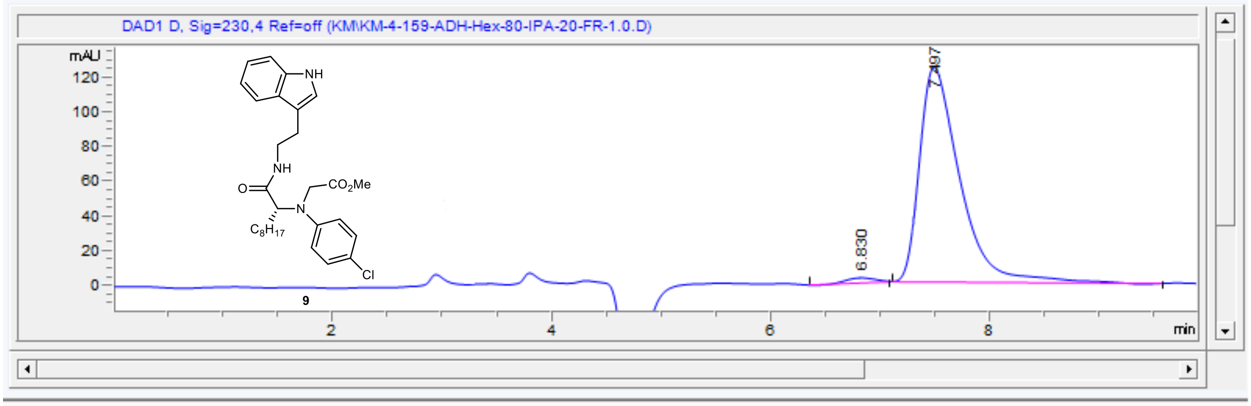

#	Time	Area	Height	Width	Area%	Symmetry
1	6.83	47.3	4	0.4086	1.423	1.334
2	7.497	3347.7	125.8	0.4434	98.577	0.522

Supplementary Figure 166. HPLC trace for methyl (*R*)-*N*-(1-[(2-(1*H*-indol-3-yl)ethyl]amino)-1-oxodecan-2-yl)-*N*-(4-chlorophenyl)glycinate (9)

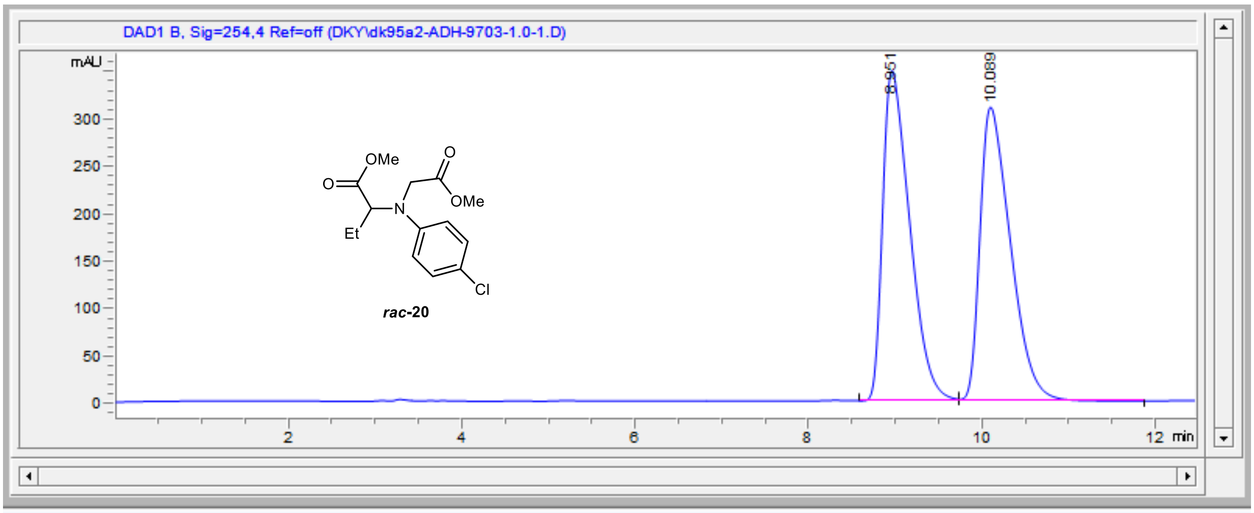

#	Time	Area	Height	Width	Area%	Symmetry
1	8.951	7537.5	348.1	0.3386	49.930	0.49
2	10.089	7558.5	309.7	0.3828	50.070	0.501

Supplementary Figure 167. HPLC trace for methyl 2-[(4-chlorophenyl)(2-methoxy-2-oxoethyl)-amino]butanoate (*rac*-20)

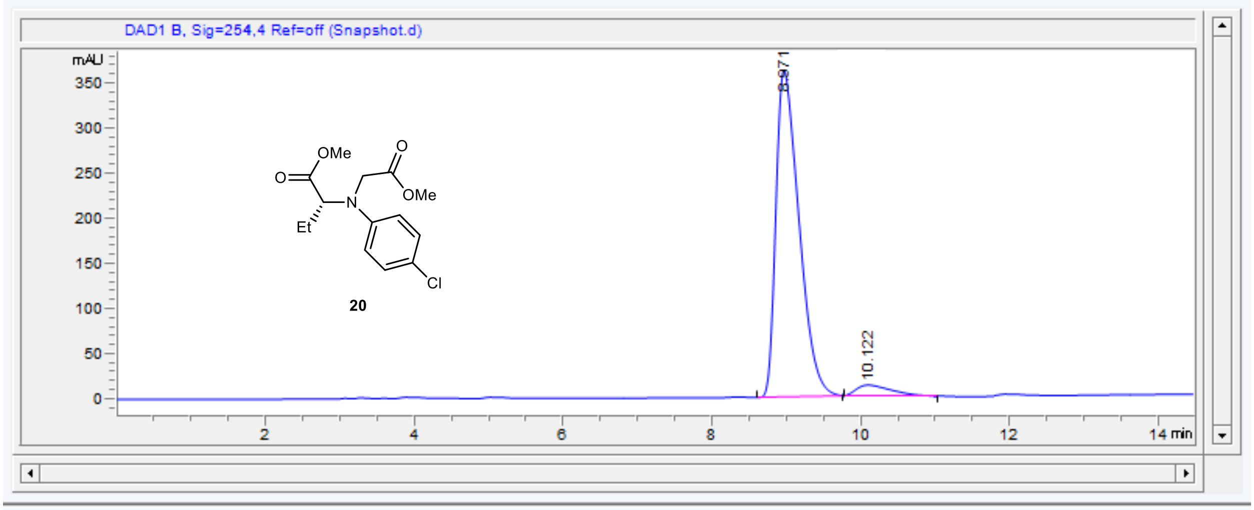

#	Time	Area	Height	Width	Area%	Symmetry
1	8.971	7898.1	364.5	0.3408	94.943	0.508
2	10.122	415.2	16.5	0.3504	5.057	0.514

Supplementary Figure 168. HPLC trace for methyl (*R*)-2-[(4-chlorophenyl)(2-methoxy-2-oxoethyl)-amino]butanoate (20)

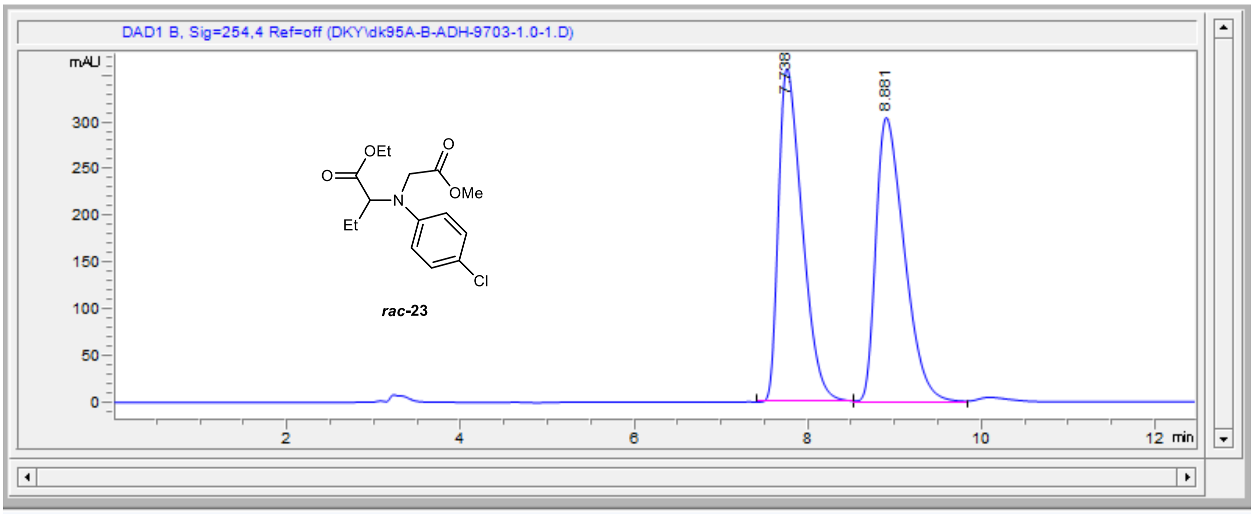

#	Time	Area	Height	Width	Area%	Symmetry
1	7.738	6773.1	355.6	0.2942	49.457	0.526
2	8.881	6921.8	303.5	0.3801	50.543	0.56

Supplementary Figure 169. HPLC trace for ethyl 2-[(4-chlorophenyl)(2-methoxy-2-oxoethyl)amino]-butanoate (*rac*-23)

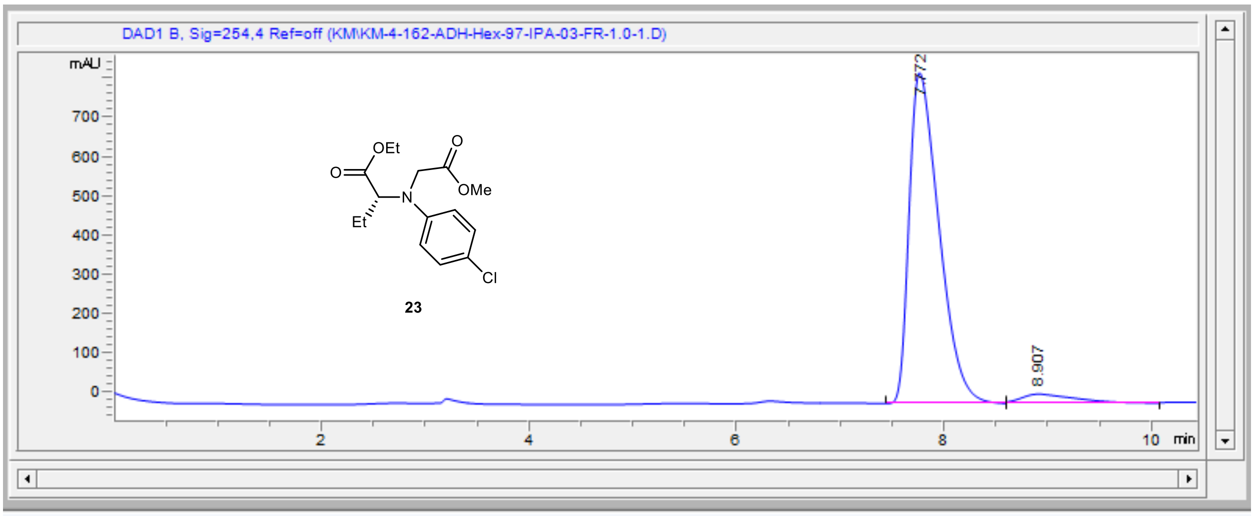

#	Time	Area	Height	Width	Area%	Symmetry
1	7.772	16986.3	846.2	0.3122	95.223	0.504
2	8.907	849.4	31.3	0.3605	4.777	0.525

Supplementary Figure 170. HPLC trace for ethyl (*R*)-2-[(4-chlorophenyl)(2-methoxy-2-oxoethyl)amino]-butanoate (23)

Supplementary References

1 Dong, K., Marichev, K. O., Xu, X. & Doyle, M. P. High stereocontrol in the preparation of silyl-protected γ-substituted enoldiazoacetates. *SynLett* **30,** 1457−1461 (2019).

2 Deng, Y., Massey, L. A., Zavalij, P. & Doyle, M. P. Catalytic asymmetric [3+1]-cycloaddition reaction of ylides with electrophilic metallo-enolcarbene intermediates. *Angew. Chem. Int. Ed.* **56**, 7479–7483 (2017).

3 Nolin, K. A., Ahn, R. W., Kobayashi, Y., Kennedy-Smith, J. J. & Toste, F. D. Enantioselective reduction of ketones and imines catalyzed by (CN-Box)Re^{V}–oxo complexes. *Chem. − Eur. J.* **16**, 9555−9562 (2010).

4 Qiao, J.-B., Zhao, Y.-M. & Gu, P. Asymmetric intramolecular desymmetrization of *meso*-α,α'-diazido alcohols with aryldiazoacetates: assembly of chiral C_3 fragments with three continuous stereocenters. *Org. Lett.* **18**, 1984−1987 (2016).

5 CrysAlisPro 1.171.38.41, Rigaku Oxford Diffraction (2015).

6 SCALE3 ABSPACK − An Oxford Diffraction program (1.0.4, gui:1.0.3), Oxford Diffraction Ltd. (2005).

7 Dolomanov, O. V., Bourhis, L. J., Gildea, R. J., Howard, J. A. K. & Puschmann, H. OLEX2: A complete structure solution, refinement and analysis program. *J. Appl. Cryst.* **42**, 339−341 (2009).

8 Sheldrick, G. M. SHELXT – Integrated space-group and crystal-structure determination. *Acta Cryst.* **A71**, 3−8 (2015).

9 Sheldrick, G. M. A short history of SHELX. *Acta Cryst.* **A64**, 112−122 (2008).
